# Bifunctional Azido(thio)ureas from an *O*-Protected 2-Amino-2-deoxy-d-glucopyranose: Synthesis and Structural Analyses

**DOI:** 10.3390/molecules29235687

**Published:** 2024-11-30

**Authors:** Concepción Sosa-Gil, Esther Matamoros, Pedro Cintas, Juan C. Palacios

**Affiliations:** 1 Departamento de Química Orgánica e Inorgánica, Facultad de Ciencias, and Instituto del Agua, Cambio Climático y Sostenibilidad (IACYS)-Unidad de Química Verde y Desarrollo Sostenible, Universidad de Extremadura, 06006 Badajoz, Spain; csosagil@hotmail.com (C.S.-G.); pecintas@unex.es (P.C.); 2 Departamento de Química Orgánica, Universidad de Málaga, Campus Teatinos s/n, 29071 Málaga, Spain; 3 Instituto de Investigación Biomédica de Málaga y Plataforma en Nanomedicina—IBIMA, Plataforma Bionand, Parque Tecnológico de Andalucía, 29590 Málaga, Spain

**Keywords:** azido(thio)ureas, amino sugars, atropisomers, conformational analysis, carbohydrate synthesis, computational chemistry

## Abstract

This publication reports a facile and convenient preparation of tri-*O*-acetyl-glucopyranoses, derived from the corresponding 2-deoxyaminosugar, where the vicinal anomeric and C2 positions are decorated by azido and (thio)ureido groups, respectively. This double functionalization leads to an inherently chiral core incorporating the versatile azido and (thio)ureido linkages prone to further manipulation. The latter also provides a structural element for hydrogen-bonded donor-acceptor (HB-DA) sites, which are of immense value in organocatalytic pursuits. A computation-aided conformational analysis unveils the landscape of available conformers and their relative stability. *N*-aryl (thio)ureas bearing substituents at ortho positions exist as mixtures of *M*- and *P*-atropisomeric conformers.

## 1. Introduction

The preparation and importance of ureido derivatives dates back to the dawn of organic synthesis, and interest has grown at a fast pace over the past century, which can be exemplified by numerous applications. Thus, the urea function itself can form inclusion complexes with long chain alkanes and some functionalized molecules [1,2]. In such compounds, the urea molecules are bound together by intramolecular hydrogen bonds (HBs), leading to helical networks in which the host compounds can be oriented and accommodated. Urea structures also function as strong HB donors and acceptors [3] and have been widely employed for obtaining supramolecular architectures [4,5]. Substituted ureas and *bis*-ureas also form gels with organic liquids and water [6,7,8,9,10,11,12,13,14,15], and in some cases, the resulting organo/hydro-gels are thermally reversible and require very little concentration [16]. The scientific literature also describes pseudodisaccharides *O*-protected by acyl groups and linked by the thiourea function [17,18,19], while, surprisingly, the corresponding disaccharide-type derivatives linked by ureido groups are less known. Some ureas derived from carbohydrates exhibit interesting biological properties such as the SF-1993 [20] and CV-1 [21,22] antibiotics and the family of glycocinnamoyl espermidines [23,24]. Moreover, glycosylureas are inhibitors of α-glycosidase [25] and *N*-nitrosoureas, such as streptozotocin [26,27] and chlorozotocin [28], and possess antitumor activity [29]. These substances induce DNA crosslinking after its in vivo degradation. The isocyanate generated by this mechanism does not seem to be directly involved in the antitumor effect, but it does react with biogenic amines and proteins. Other side effects include the inhibition of RNA synthesis, DNA polymerase, and other enzymes involved in DNA repair, all accounting for the toxicity of nitrosoureas [30].

Methods aimed at synthesizing ureidosugars are usually limited to a few protocols, involving the condensation of monosaccharides with ureas to give symmetrical glycosylureas [31] and ureidodisaccharides [32,33], the reaction of glycosylamines [34,35], amino sugars [36,37,38,39,40] with isocyanates or their synthetic equivalents such as carbamates [41,42,43], the addition of water to diglycosylcarbodiimides [44,45], as well as by-products in various transformations of glycosyl isocyanates [43,46], each possessing inherent pros and cons.

**Chart 1:**
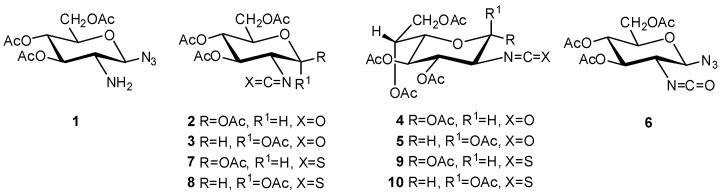
Structures of azido precursor **1** and iso(thio)cyanates **2**–**10** used throughout this work.

It is likely that the synthetic protocol that still remains versatile enough to access a broad range of structurally diverse glycoconjugates and pseudo-oligosaccharides is the reaction of *O*-protected amino sugars with either isocyanates or isothiocyanates. Therefore, in this work, we describe the preparation of a series of ureas and thioureas linked to the sugar fragment through non-anomeric carbon atoms, starting from d-glucosamine as the available precursor. In order to increase the functionality relative to previous work, we thought of the labile azido group at the anomeric carbon as tri-*O*-acetyl-2-amino-2-deoxy-β-d-glucopyranosyl azide (**1**) can readily be obtained [47]. Organic azides can undergo a variety of synthetically useful transformations, particularly reducing into amine group [48,49,50] or the unique azide-alkyne cycloaddition [51,52,53,54]. On the other hand, sugar iso(thio)cyanates have long been known in carbohydrate chemistry, with the first example at the non-anomeric position being reported by Jochims and Seeliger [55] as early as 1965 using a phosgene stream. Clearly, the use of neat phosgene is to be highly discouraged in contemporary synthesis. A safer and less hazardous surrogate is a 1.93 *M* solution of phosgene in toluene, which was applied by Nowick et al. [56] to the preparation of isocyanates derived from amino acid esters. We have employed this in previous work in the synthesis of isocyanates from amino sugars (**2**–**5**) [57]. A third method, which is safe enough, involves triphosgene, Cl_3_C-OCOO-CCl_3_, a synthetic equivalent of phosgene, under heterogeneous conditions, similar to those of the Schotten–Baumann synthesis [58]. This way, isocyanate **6** has been obtained from **1** (Chart 1). In contrast, liquid thiophosgene, even if highly toxic, can easily be manipulated under heterogeneous biphasic conditions and removed by distillation. The method has been applied to the preparation of isothiocyanates **7–10** [17,18,19,55].

## 2. Results and Discussion

### 2.1. Syntheses and Structural Characterization

**Scheme 1:**
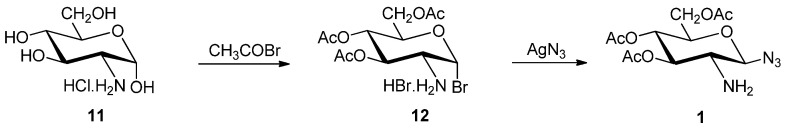
Two-step preparation of 3,4,6-Tri-*O*-acetyl-2-amino-2-deoxy-β-d-glucopyranosyl azide.

3,4,6-Tri-*O*-acetyl-2-amino-2-deoxy-β-d-glucopyranosyl azide (**1**) is an ideal starting material for the preparation of chiral derivatives functionalized at C1 and C2 pyranose positions. Its synthesis, according to an old protocol [47], involves the treatment of d-glucosamine hydrochloride (**11**) with acetyl bromide and the subsequent treatment of the resulting α-configured glucopyranosyl bromide hydrobromide (**12**) with silver azide, which follows an S_N_2 mechanism and proceeds with the reversal of the anomeric configuration, thus forming the β-glycosyl azide (Scheme 1).

The structure of **1** was confirmed by coincidence of its physical data with those described [47] and was supported by analytical and spectroscopic data. Thus, the IR spectrum shows both asymmetric and symmetric stretching vibrations of the NH_2_ group at 3380 and 3326 cm^−1^, respectively, together with an intense stretching band of the azido group at 2112 cm^−1^. The ^1^H NMR spectrum shows three signals for acetate methyl groups at ~2.1 ppm, while the anomeric proton (4.54 ppm) and the H-2 proton (2.83 ppm) lie in a shielding zone and are shifted upfield due to the low electronegativity of the vicinal azido and amino groups. The high coupling constants *J*_1,2_ = *J*_2,3_ = *J*_3,4_ = *J*_4,5_ (=9.5 Hz) are consistent with a d-*gluco* pyranoid ring in ^4^*C*_1_ conformation and β-anomeric configuration.

**Scheme 2:**
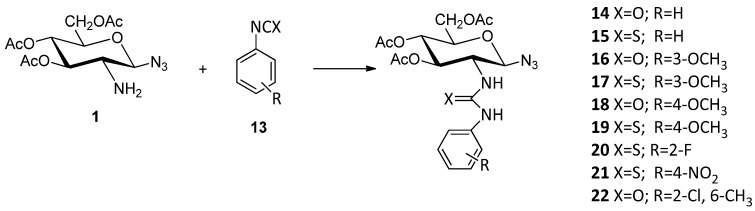
Direct preparation of ureido or thioureido glucopyranosyl azides.

The reaction of **1** with equimolar amounts of an aryl isocyanate or aryl isothiocyanate (**13**) in benzene or CH_2_Cl_2_ at room temperature led to the ureido and thioureido derivatives **14**–**22** (Scheme 2), which often crystallized spontaneously and could be isolated in moderate to high yields (45–97%), although their syntheses have not been optimized (Table S1). Again, all spectroscopic data fully support such structures. IR spectra show absorptions at ~3550–3250 cm^−1^, ~2100 cm^−1^, ~1750 cm^−1^, ~1250 cm^−1^, and ~1650 cm^−1^, accounting for stretching vibrations of the multiple functional groups present, namely NH, N=N=N, C=O (ester and amide), and C-O-C bonds [59]. Most protons and carbon atoms of compounds **14**–**22** show similar chemical shifts. In the ^1^H NMR spectra, downfield resonances correspond in all cases to the NH protons of either ureido or thioureido linkages between 8.2 and 7.0 ppm and which are close to or overlapping the typical aromatic protons (~7.5–6.5 ppm). Signals at the upper field, in the interval between 5.2 and 3.7 ppm, belong to the aminosugar moiety and appear in the following order: H-3 > H-4 > H-1 > H-6 > H-6′ > H-2 > H-5 (Table 1). When comparing the signals of H-2 of **14**–**22** with those of the starting product **1**, a strong deshielding is generally observed (Δδ ~1.0 ppm for the ureido derivatives and Δδ ~2.1 ppm for the thioureido ones) as a consequence of the transformation of the NH_2_ group into the (thio)urea functionalities (Table 1). The remaining signals can easily be attributed to the aromatic protons of the starting isocyanate or isothiocyanate (Table S2). For *meta*- and *para*-OMe-substituted compounds, the methyl protons resonate at ~3.8 ppm. Unambiguous assignments could be established through double-resonance experiments, 2D correlations, and D_2_O isotopic exchange, with the latter proving that the singlet signal between 7.0 and 8.0 ppm corresponds to the NH group directly bound to the aromatic ring, whereas the doublet at ~6.0 ppm corresponds to the other NH linked to the sugar.

Likewise, ^13^C chemical shits (Table 2) could be assigned through polarization transfer (DEPT) and heteronuclear ^1^H-^13^C correlation spectra. As expected, the most deshielded signal at ~180 ppm can be attributed to the C=S bond of thioureas, while the carbonyl group of ureido derivatives appear at ~150 ppm. The carbonyls of the acetates groups are located between them at ~170 ppm, while the signals of their methyl groups invariably appear at ~21 ppm. The carbon signals of the pyranose ring generally follow a diagnostic sequence as follows: C-1 > C-5 > C-3 > C-4 > C-6 > C-2. Due to the poorly electron-withdrawing character of the azido group, the chemical shift in the anomeric carbon lies below 90 ppm. For substituted aryl rings, the methoxy groups of **16**–**19** appear at ~55 ppm and the methyl signal of **22** at ~18 ppm.

**Scheme 3:**
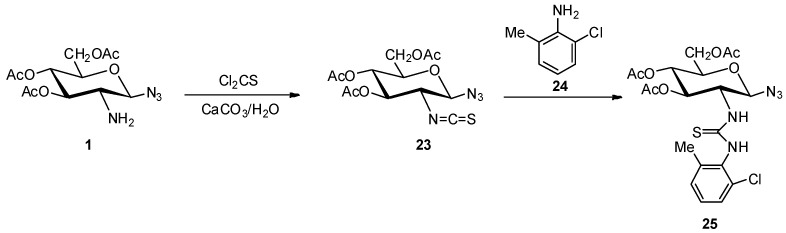
General route towards 2-isothiocyanato-β-d-glucopyranosyl azide (**23**) and coupling with anilines.

A vigorously stirred biphasic reaction of thiophosgene and a chloroform solution of **1** in the presence of a saturated aqueous solution of CaCO_3_ made it possible to obtain 3,4,6-tri-*O*-acetyl-2-deoxy-2-isothiocyanato-β-d-glucopyranosyl azide (**23**) in a 58% yield (Scheme 3). The IR spectrum of the latter shows strong absorptions at 2120 and 2077 cm^−1^, typical of stretching vibrations of the N=N=N and N=C=S heterocumulenes, respectively. The isothiocyanato group causes a strong deshielding of the H-2 proton (~0.9 ppm) in the ^1^H NMR spectrum, while the carbon signal resonates at 142 ppm.

The possibility of condensing **23** with substituted anilines provides a straightforward route to thioureas. As shown in Scheme 3, the use of 2-chloro-6-methylaniline (**24**) led to the corresponding thiourea **25**, precipitated from the reaction mixture as an orange solid after 72 h. The low isolated yield (22%) is most likely due to the steric hindrance caused by two substituents at the *ortho* positions. Although its spectroscopic data are similar to those outlined above for other thioureas, the interpretation of NMR spectra recorded at room temperature is challenging. The spectra are poorly resolved, and some signals are absent relative to similar structures, such as **22**, for instance (Figure S44, Supplementary Materials). As we will see later, this behavior evidences the existence of a slow equilibrium between different conformers. Table 1 and Table 2 collect the most salient spectroscopic parameters of **25**. The ^13^C NMR spectrum could be resolved at 223 K, the temperature at which the signals split into those of the individual conformers.

### 2.2. Conformational Analysis of Urea/Thiourea Derivatives

All the new compounds show large coupling constants, *J*_2,3_ = *J*_3,4_ = *J*_4,5_ (>8.5 Hz), in agreement with a d-*gluco*-configured pyranoid ring in a ^4^*C*_1_ conformation (Table 3) and β-configuration at the anomeric position, the latter being witnessed by the large coupling constant *J*_1,2_ (~9.5 Hz).

**Figure 1:**
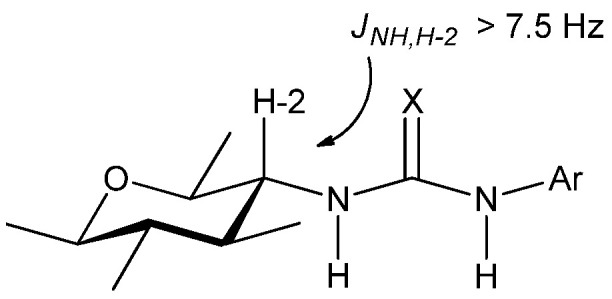
Experimental relative disposition in solution of urea/tiourea and sugar ring fragments.

Furthermore, the high value measured for *J*_NH,H-2_ > 7.5 Hz evidences an antiperiplanar relationship between the H-2 and NH protons. As a result, the half-plane containing the urea/thiourea group is arranged approximately perpendicular to the plane containing the pyranoid ring (Figure 1).

**Scheme 4:**
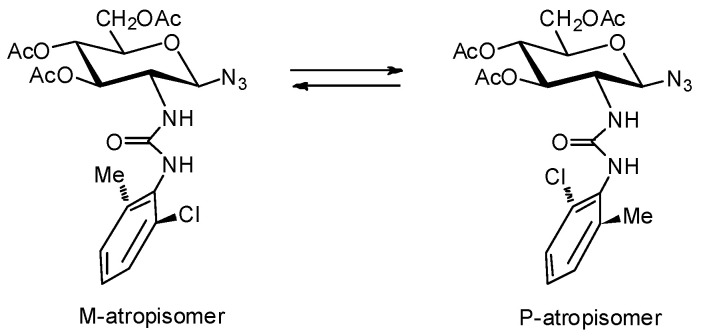
Atropisomeric conformers derived from *o,o’*-disubstituted *N*-aryl ureas.

As mentioned above, some ^1^H NMR spectra of these (thio)ureido derivatives show broad signals with poor resolution. A preliminary surmise points to the average spectra resulting from rotamers that interconvert slowly at room temperature. The origin of such a restricted rotation should reasonably be ascribed to the presence of amide or thioamide bonds. In addition, compounds **22** and **25** could exhibit double configurational restrictions arising from both the urea/thiourea linkages and the bulky groups located at the *ortho* positions [60,61] (Scheme 4).

Variable-temperature experiments were carried out to verify this working hypothesis. Thus, when decreasing the temperature, the proton signals of compound **22** broadened and ultimately split into two rounded peaks, thus indicating the coexistence of two rotamers at least. Something similar happened when recording the spectra of thiourea **25** at different temperatures. As the temperature increased, the wide signals observed at room temperature evolved to sharp and well-defined peaks, while when decreasing the temperature to 223 K, double signal sets could be detected. The coalescence temperature (T_c_) was determined to be 270 K. These experiments allowed us to quantify the energy barrier to rotation. At the coalescence temperature, the rate constant between two equilibrium states equally populated and uncoupled is given by the following expression [62,63]:

*k* = πΔν/(2)^½^ (1)

where Δν represents the frequency difference between the analogous signals, corresponding to both rotamers. This expression is not completely accurate when the equilibrium takes place between unequally populated states, yet it can be used to obtain an estimated value of the interconversion barrier because the error is usually small. On the other hand, according to the Theory of the Transition State, the variation in the rate constant of an elementary reaction step with temperature is given by Equation (2) [64]:

*k* = (k_B_T_c_/h)exp − (ΔG^‡^/RT_c_) (2)

where R, k_B,_ and h represent the ideal gas, Boltzmann, and Plank constants, respectively.

From Equations (1) and (2), the barrier (ΔG^‡^) can be deduced by equaling them in their logarithmic form as follows:

ln[πΔν/(2)^½^] = ln (k_B_T_c_/h) − (ΔG^‡^/RT_c_) (3)

and rearranging the terms as follows:

ΔG^‡^/RT_c_ = ln[k_B_T_c_(2)^½^/πhΔν] (4)

resulting in an expression used for calculating the rotational barrier:

ΔG^‡^ = RT_c_ln[k_B_T_c_(2)^½^/πhΔν] (5)

By substituting the constants in the latter, Equation (6), which has been employed in our calculations, results in

ΔG^‡^ (cal/mol) = 1.987T_c_ [22.62 + ln(T_c_/Δν)] (6)

For compound **25**, taking into account its coalescence temperature and the chemical shift difference Δν (in frequency units), the activation free energy (ΔG^‡^) corresponds to 11.2 kcal/mol. This rotational barrier is consistent with the existence of a slow equilibrium at room temperature, which probably corresponds to the interconversion of rotamers of the thioamide bond.

### 2.3. Theoretical Calculations

To clarify further and rationalize the above assumptions, a series of DFT-based calculations [65,66,67,68,69,70] have been performed on the stability of the different conformations adopted by ureas and thioureas in solution. Modeling was carried out with the Gaussian package [71] using the hybrid density functional M0-62X [72] in combination with the 6-311G(d,p) [73,74] and the def2-TZVP valence-triple-ζ [75] basis sets, both in the gas phase and when simulating the presence of chloroform (the SMD method) [76], the solvent in which NMR spectra were recorded.

**Figure 2:**
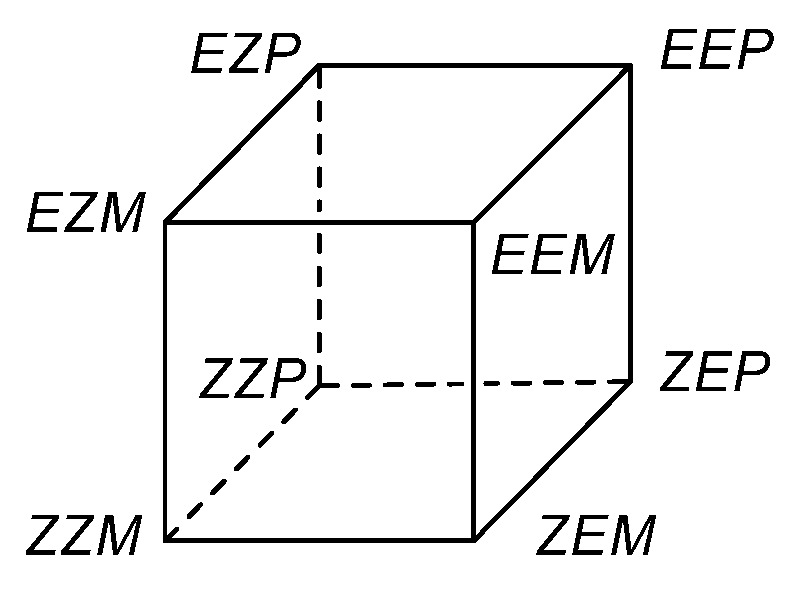
Conformational space for (thio)ureido derivatives showing their potential interconversions.

Since the NH and the H-2 signals always show an antiperiplanar disposition (*J*_NH,H-2_ > 7.5 Hz), in line with other (thio)ureido derivatives from sugars [60,61], the possible conformers are reduced to four for **14**–**21**. Compounds **22** and **25**, however, owing to the restricted rotation caused by *ortho* substituents, could also occur as a mixture of *M* and *P* atropisomers, thereby doubling the number of potential conformers (Figure 2).

**Scheme 5:**
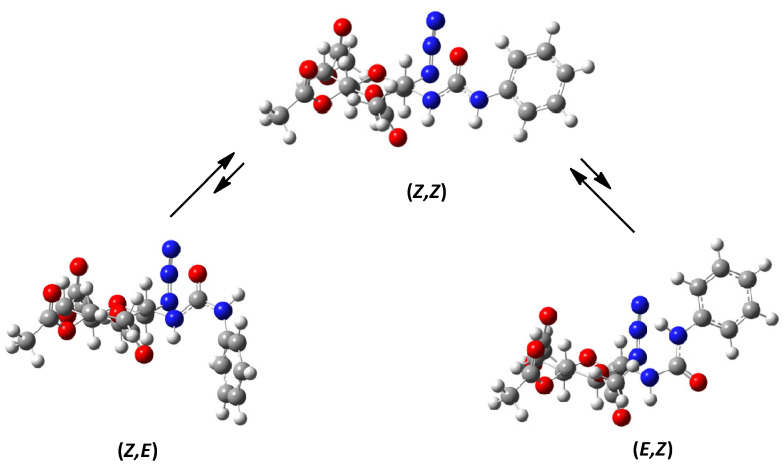
Optimized structures for the conformational rotamers of azido urea **14** (color code: carbon, gray; hydrogen, white; oxygen, red and nitrogen, blue).

Table 4 shows the energies calculated for the different conformations, (*Z*,*Z*), (*Z*,*E*), and (*E*,*Z*), of urea **14** and Scheme 5 for their optimized structures. The most stable rotamer, both in the gas phase and in CHCl_3,_ is the (*Z*,*Z*) structure, which should be equilibrated in solution with the (*Z*,*E*) rotamer.

**Scheme 6:**
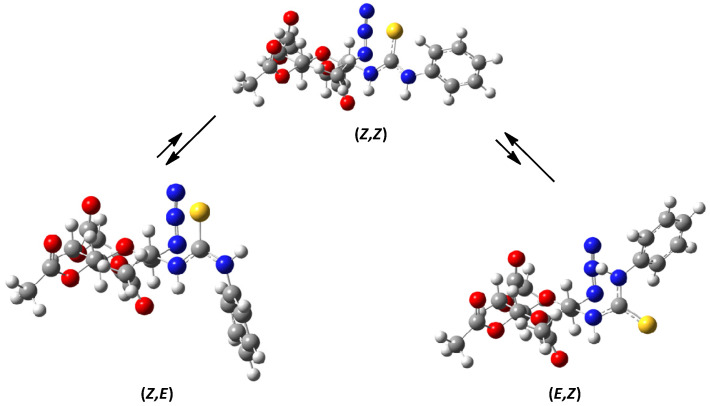
Optimized structures for the conformational rotamers of azido thiourea **15** (color code: carbon, gray; hydrogen, white; oxygen, red; nitrogen, blue and sulfur, yellow).

An analogous computation has been carried out for the conformational space of azido thiourea **15**, whose data and optimized structures are shown in Table 4 and Scheme 6, respectively. Conversely, the (*Z*,*E*) conformer of **15** now possesses the greatest stability in both the gas phase and in solution, a fact that deviates from the stability order obtained for the conformers of **14**. Clearly, the larger volume of the thiocarbonyl group C=S relative to that of C=O is behind this change.

**Figure 3:**
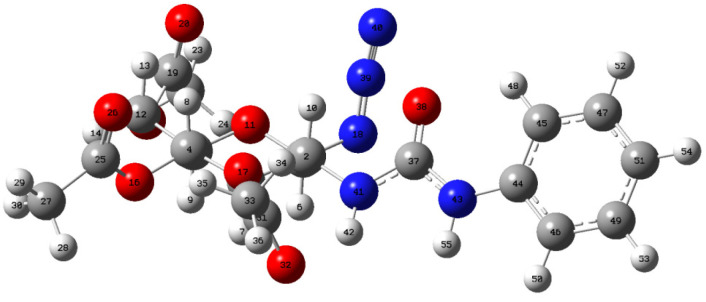
Atom numbering for the calculated structure of **14** (*Z,Z* conformer) (color code: carbon, gray; hydrogen, white; oxygen, red and nitrogen, blue).

**Figure 4:**
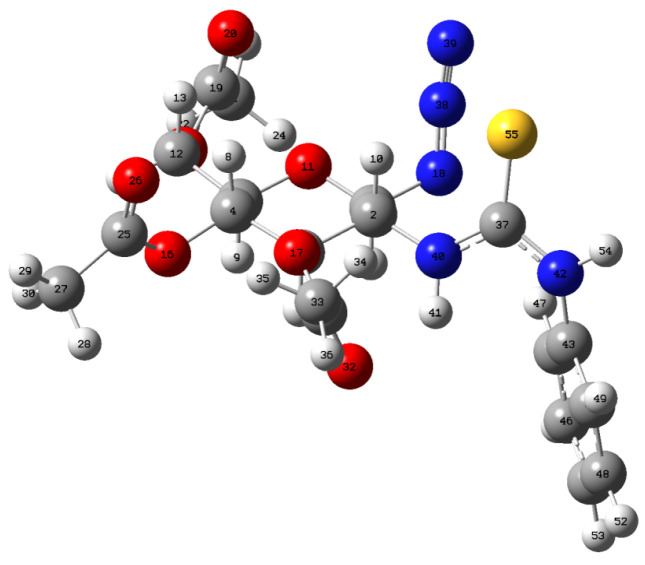
Atom numbering for the calculated structure of **15** (*Z,E* conformer) (color code: carbon, gray; hydrogen, white; oxygen, red; nitrogen, blue and sulfur, yellow).

Table 5 and Table 6 depict a selection of bond distances, virtual angles, and significant dihedral angles for the optimized structures of the (*Z*,*Z*), (*Z*,*E*), and (*E*,*Z*) conformers of **14** and **15**. For the sake of clarity, Figure 3 and Figure 4 show the numbering codes to define the bond lengths and angles. In all cases, the pyranoid ring retains the ^4^*C*_1_ conformation, which minimizes the steric interactions of the ring substituents. Interestingly, the linear azide group adopts a quasi-axial arrangement and is parallel to the C=O/C=S groups in the (*Z*,*Z*) and (*Z*,*E*) conformers. The dihedral angles between N39-N18 and C37-O38/C37-S55 are close to 0°.

It is noteworthy that the distance between O38 and H48 at the aromatic ring of **14** is significantly lower than the sum of the van der Waals radii of both atoms (2.19–2.21 < r_vw_^H^ + r_vw_^O^ = 1.17 + 1.52 = 2.69 Å) [77], indicating a putative interaction that contributes to the coplanarity of the benzene ring with the flat urea system, as reflected by the low dihedral angle involving the C=O bond (C37-O38) and the C44-C45 bond of the aromatic ring (~5°). Thiourea **15**, however, lacks such a coplanarity (~40°); the aromatic ring rotates to accommodate the bulky sulfur atom, and the distance between the latter and H47 at the aromatic ring is very close to the sum of the van der Waals radii (2.78–2.82~r_vw_^H^ + r_vw_^S^ = 1.17 + 1.8 = 2.97 Å).

On the other hand, the spatial orientation between the proton at C-2 of the pyranose and the NH group is close to an antiperiplanar arrangement. They form dihedral angles of ~140–160º in the (*Z*,*Z*), (*Z*,*E*), and (*E*,*Z*) conformers of **14** and **15**. This geometry can also be inferred from experimental NMR data, i.e., the high values of *J*_NH,H-2_ (~8–9 Hz), which indicate an antiperiplanar relationship between both protons. Moreover, the downfield shifts shown by the H-2 of ureas (Δδ ~1 ppm) and thioureas (Δδ ~2 ppm) with respect to that observed for the H-2 signal of **1** agree with an arrangement in which the C=O or C=S bonds lie parallel to the C2-H bond. Overall, the conformational arrangement involving such bonds should generate, at least in part, a strong deshielding with anisotropic effects experienced by H-2 in particular (Figure 1).

**Figure 5:**
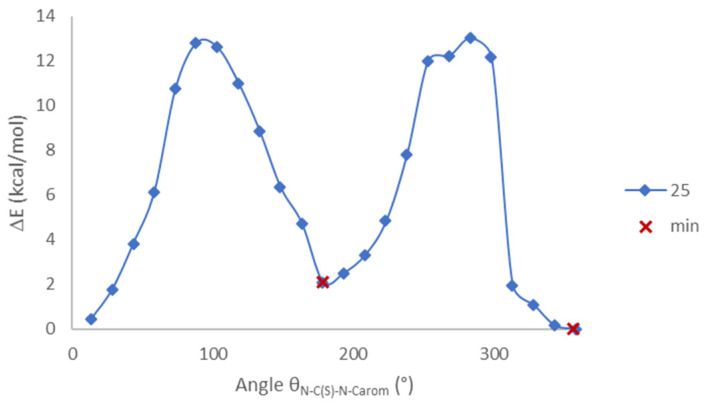
Conformational profiles of the aryl moiety of **25** at the M06-2X/6-311G(d,p) level in the gas phase.

A conformational analysis of the aryl moiety of **25** has been achieved, and the energy landscape of the arrangements generated around both N-C(S) bonds calculated, i.e., by rotating the dihedral angle θ_N-C(S)-N-Carom_ from 0° to 360° with a step size of 15° each. Again, the computational analysis was performed using the aforementioned 6-311G(d,p) and def2-TZVP valence-triple-ζ basis sets, with all geometries optimized in the gas phase at the M06-2X level of theory without any geometrical restriction. Bulk solvation has been reproduced with the SMD method as well. The M06-2X/def2-TZVP combination has been reported to provide suitable geometry optimization in terms of cost and accuracy for carbohydrate derivatives [78,79,80]. Such results are shown in Figure 5 and Table 7 and Table 8.

**Figure 6:**
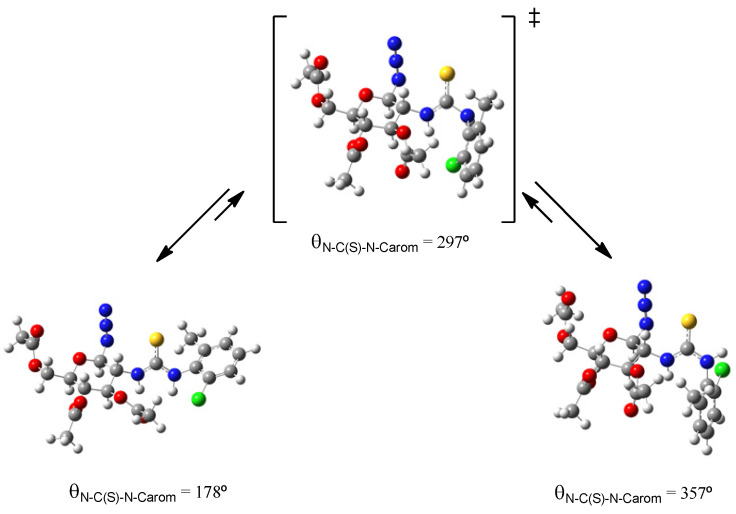
Optimized structures for the two conformational minima of **25** and the transition structure (‡) at the M06-2X/6-311G(d,p) level.

Results are similar at both levels of calculation. The most stable conformations found correspond to the *ZZ* and *ZE* arrangements (entries 1 and 2, respectively) and the calculated barrier to interconversion between them is ~10–12 kcal/mol (Table 9, Figure 6), a value very close to that found experimentally using dynamic NMR.

**Chart 2:**
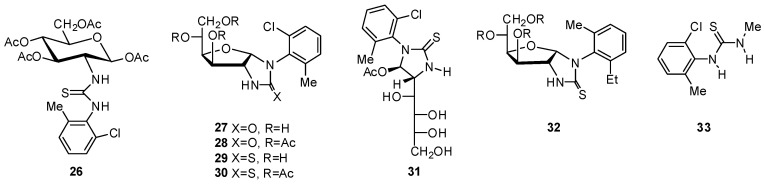
Acyclic and cyclic atropisomeric (thio)ureido derivatives.

Furthermore, this value is similar to that reported for thioureido derivative **26** (~14 kcal/mol), having a close structural resemblance to **25**, which was assigned to the interconversion between *M* and *P* rotamers [60]. However, the cyclic (thio)ureas **27**–**31** showed higher barriers to this rotation, >22 kcal/mol, as measured by dynamic NMR and/or estimated by theoretical calculations (Chart 2). Accordingly, both rotamers are configurationally stable and could be isolated [60,61].

**Scheme 7:**
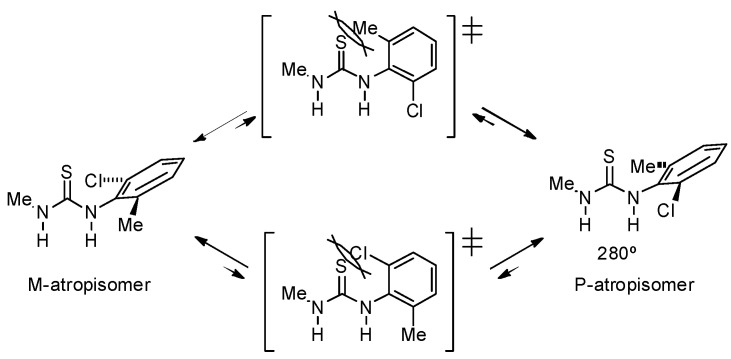
Conformational profile of the *M*/*P* interconversion for thiourea **33**.

**Figure 7:**
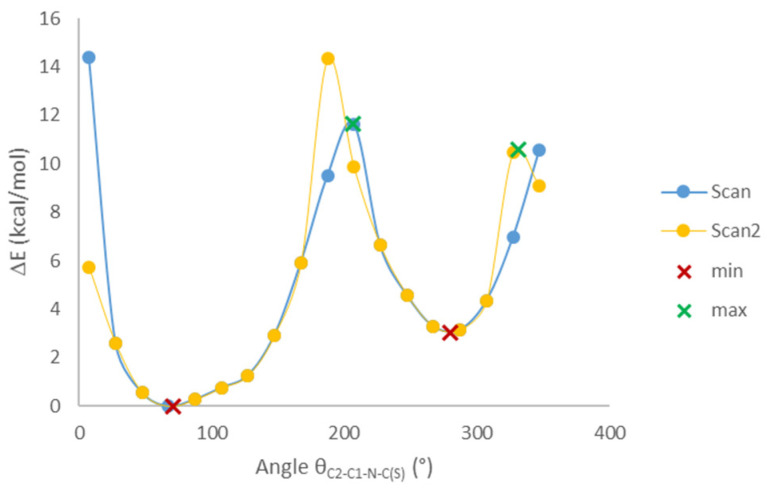
Conformational profiles for the M/P interconversion of **33** at the M06-2X/6-311G(d,p) level in the gas phase.

The gap found in such barriers could be reasonably ascribed to the greater flexibility of the acyclic thiourea system. To verify the point, we calculated the rotational profile around the angle θ_C2-C1-N-C(S)_. A simpler molecular model (**33**) was employed to avoid the conformational complications caused by the sugar moiety and to reduce the computational cost. The expected landscape is shown in Scheme 7, which was carried out from an angle of 280° with a step size of 20° until completing 360°, first clockwise and then counterclockwise. Data for all minima and maxima found are collected in Table 10 and depicted in Figure 7. The determination of the stabilities of the *M* and *P* rotamers and the corresponding transition states would allow us to estimate the barrier to rotation.

**Figure 8:**
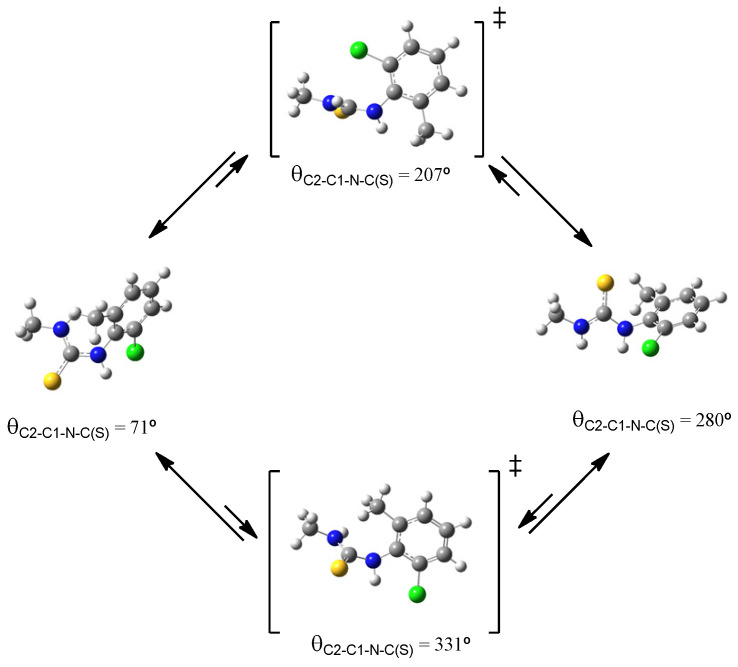
Optimized structures for the stationary points related to the rotational evolution in compound **33** and the transition structure (‡).

Both curves are practically coincidental and show that the rotation barrier takes a value of ~13.5 kcal/mol, similar to that determined for **25** and **26**. Figure 8 shows the structures of the corresponding maxima and minima. It is worth pointing out that the initial *ZZP*-rotamer does not transform into the *ZZM*-rotamer, but into the *ZEP*; that is, the same transformation as for compound **25**, as shown in Figure 5. Notably, the saddle points do not correspond to those represented in Scheme 7. It is evident that this transformation is energetically more favorable than atropisomerization, in which the half-planes defined by the thiourea linkage and the aromatic ring become coplanar, while either the chlorine atom or the methyl group interact with a bulky sulfur atom.

### 2.4. Unprotected Ureas

**Scheme 8:**
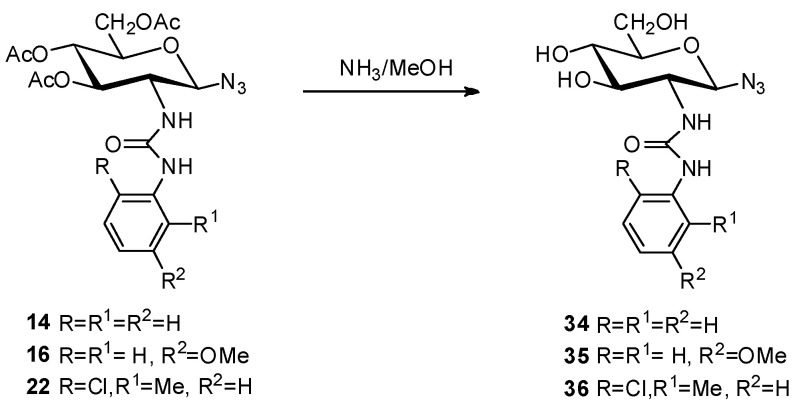
Deacetylation of glucosyl azidoureas.

Selective *O*-deacetylation reactions were attempted using the conventional and mild conditions provided by a saturated solution of ammonia in methanol at room temperature. Compounds **14**, **16**, and **22** could be successfully deacetylated (Scheme 8) without affecting other functional groups, leading to the corresponding unprotected derivatives **34**–**36**, respectively, in high yields (Table S3). In stark contrast, the deacetylation protocol failed with thioureas, giving rise to messy reactions. As portrayed by **15**, proton NMR monitoring shows a complex mixture, for which the multiple signals most likely result from the decomposition of the starting material.

This transformation can easily be monitored by TLC, and, after completion, the isolation protocol essentially involves filtration and further purification by recrystallization from ethanol. High-resolution mass spectra (HRMS, CI mode) allowed us to determine the molecular weight and compositions of **34**–**36** (Figures S75–S77). The peak [M+Na]^+^ was detected in the case of **34** at *m*/*z* 346.1122, consistent with the formula C_13_H_17_O_5_N_5_Na and for **35** at *m*/*z* 376.1228, which agrees with C_14_H_19_O_6_N_5_Na. For urea **36**, the [M+Na]^+^ peaks corresponding to the ^35^Cl and ^37^Cl isotopes could be detected, with an approximate intensity ratio 3:1, with the former being detected at *m*/*z* 394.0889 with composition C_14_H_18_O_5_N_5_ClNa.

All spectroscopic data support unequivocally the structures of such compounds as well. The absence of acetate absorptions in the IR spectra at ~1730 cm^−1^ evidence a complete deacetylation reaction. The typical broad bands of the OH groups and the NH peak appear between 3500 and 3100 cm^−1^, whereas the alcohol C-O bond lies at 1080 cm^−1^ (Figure S13). The intense signal of the azido group is preserved at ~2120 cm^−1,^ and that of the urea carbonyl at ~1620 cm^−1^. Well-resolved ^1^H NMR spectra could be obtained in all cases, thus facilitating assignment (Table 11) and being corroborated by double-resonance experiments and D_2_O-induced isotopic exchange conducted on **34** (Figure S47). As expected, the most deshielded signal corresponds to the NH group linked directly to the aromatic ring (~8.50 ppm), while the NH bound to the pyranoid ring appears as a doublet at ~6.2 ppm. The anomeric proton resonates at ~4.5 ppm, and the remaining ring protons now follow the shift order H-6 > H-2, H-6′ > H-4, H-5 > H-3. The high coupling constants *J*_2,3_ = *J*_3,4_ = *J*_4,5_ (~9.5 Hz) indicate that the pyranoid ring maintains a ^4^*C*_1_ conformation with d-*gluco* configuration, and the high value of *J*_1,2_ (~9.0 Hz) evidences that the equatorial (β) orientation of the azido group remains unaffected. ^13^C NMR data are gathered in Table 12. Together with the absence of resonances for the acetate groups, the urea carbonyl signal is observed at ~160 ppm (Figure S72). The HMQC correlation spectrum enables the assignment of the pyranoid carbons following the order C-1 > C-4 > C-5 > C-3 > C-6 > C-2 (Table 11).

## 3. Conclusions

The present study repurposes the use and some applications of a per-*O*-acetylated-2-amino-2-deoxy-glucopyranosyl azide (**1**), described more than seven decades ago. This chiral synthon can be leveraged to obtain a series of new ureas and thioureas by reaction with aryl isocyanates and isothiocyanates, which can exist as mixtures of conformers in solution, while preserving the pyranoid conformation and the anomeric configuration. The new sugar isothiocyanate 3,4,6-tri-*O*-acetyl-2-deoxy-2-isothiocyanate-β-d-glucopyranosyl azide (**23**) has been obtained by a reaction of (**1**) with thiophosgene. Conformational analysis inferred from NMR data and theoretical calculations evidence a greater stability of the (*Z,Z*) conformer for ureas and the (*Z,E*) conformer for the corresponding thioureido derivatives, a fact attributed to the changes induced by carbonyl or thiocarbonyl groups. *N*-aryl (thio)ureas featuring *ortho*-disubstitution can also exist as atropisomeric conformers due to an additional bond rotation, for which the low energy barrier could be determined, as illustrated by the thioureido derivative **25** obtained by a reaction of **23** with 2-chloro-6-methylaniline. Azidoureas can easily be deacetylated with ammonia in methanol, affording derivatives with free OH groups and unaltered functionality, a process that failed for thioureas, nevertheless. In summary, the new family of carbohydrate (thio)ureas possessing the azido group at the anomeric position offers new synthetic avenues to be explored. Such manipulations range from expected click-type reactions, already developed for *N*-acetyl d-glucosamino azides, to the preparation of glycoconjugates and glycomimetics tailored for specific applications [49,81,82,83].

## 4. Experimental

### 4.1. General Methods

All reagents and solvents were obtained from commercial suppliers and used without further purification. Melting points were determined on Gallenkamp MPA (York, UK) and Electrothermal IA 9000 (York, UK) apparatuses and are uncorrected. Optical rotations were determined on a Perkin-Elmer 241 polarimeter (Waltham, MA, USA) at 22 ± 2 °C, with sodium (D line, λ = 589 nm) and mercury beams (λ = 578, 546, 436 nm). IR spectra were recorded in the range of 4000–600 cm^−1^ on an FT-IR Thermo spectrophotometer (Waltham, MA, USA). Solid samples were recorded on KBr (Merck (Darmstadt, Germany)) pellets. NMR spectra were measured on Bruker 400 and 500 AC/PC instruments (Karlsruhe, Germany) in DMSO-*d*_6_ or CDCl_3_. Structural elucidation was facilitated through (a) distortionless enhancement by polarization transfer (DEPT), (b) 2D correlation spectroscopy (COSY), (c) heteronuclear multiple-quantum correlation (HMQC), (d) heteronuclear multiple bond correlation (HMBC), (e) isotope exchange with deuterium oxide, and (f) variable-temperature experiments. All *J* values are given in hertz. Microanalyses were determined on a Leco^®^ CHNS-932 analyzer (St. Joseph, MI, USA). High-resolution mass spectra (HRMS) were obtained using electrospray ionization (ESI) techniques with a 6520 Accurate-Mass Q-TOF LC/MS system from Agilent Technologies (Santa Clara, CA, USA) at the Servicio de Apoyo a la Investigación (SAIUEX) from the University of Extremadura.

### 4.2. Computational Details

The computational DFT study was carried out using the hybrid functional M06-2X [72] in conjunction with 6-311G(d,p) and def2-TZVP valence-triple-ζ basis sets [73,74,75], as implemented in the Gaussian09 package [71]. In all cases, frequency analyses were carried out to confirm the existence of true stationary points on the potential energy surface. All thermal corrections were calculated at the standard values of 1 atm at 298.15 K. Solvent effects were modeled through density-based, self-consistent reaction field (SCRF) theory of bulk electrostatics, i.e., the solvation model based on density (SMD) [76]. This solvation method accounts for long-range electrostatic polarization (bulk solvent) together with short-range effects due to cavitation, dispersion, and solvent structural effects.

### 4.3. Synthetic Procedures

*3,4,6-Tri-O-acetyl-2-amino-2-deoxy-β-d-glucopyranosyl azide* (**1**) **[47]**. To a solution of **12** (2.25 g, 6.1 mmol) in chloroform (20 mL) a suspension of silver azide in chloroform (from 1.0 g of sodium azide) was added. The mixture was vigorously refluxed, filtered, and washed with chloroform. The filtrate was evaporated to dryness and the crude product (1.6 g, 95%) was crystallized from ethanol, affording yellowish needles (1.1 g, 60%), which had m.p. 120–121 °C. IR (KBr) ν_max_ 3380, 3326 (NH_2_), 2112 (N_3_), 1748 (acetate), 1237 (C-O-C), 1040 (C-O), 905 cm^−1^. ^1^H NMR (400 MHz, CDCl_3_): δ (ppm) 5.03 (t, 3H, H-3, *J* 15.2 Hz, *J* 7.6 Hz), 4.97 (t, 3H, H-4), 4.54 (d, 1H, H-1, *J* 7.2 Hz), 4.30 (dd, 1H, H-6, *J* 10.0 Hz, *J* 4.0 Hz), 4.14 (dd, 1H, H-6a, *J* 9.6 Hz, *J* 1.6 Hz), 3.80–3.77 (m, 1H, H-5), 2.83 (t, 1H, H-2, *J* 14.8 Hz, *J* 7.2 Hz), 2.10 (s, 3H, OAc), 2.08 (s, 3H, OAc), 2.03 (s,3H, OAc), 1.50 (bs, 2H, NH). ^13^C{^1^H} NMR (100 MHz, CDCl_3_): δ (ppm) 170.6 (2CO), 169.6 (CO), 91.8 (C-1), 75.1 (C-5), 73.9 (C-3), 68.2 (C-4), 61.9 (C-6), 55.8 (C-2), 20.7, 20.6, and 20.5 (OAc).

*3,4,6-Tri-O-acetyl-2-deoxy-2-(3-phenylureido)-β-d-glucopyranosyl azide* (**14**). Phenyl isocyanate (0.16 mL, 1.51 mmol) was added under constant stirring to a cooled (ice bath) solution of **1** (0.50 g, 1.51 mmol) in methylene chloride (4 mL). Immediately, a white crystalline solid separated (0.24 g, 97%), which had m.p. 170–171 °C. [α]_D_ −11.9°; [α]_578_ −11.9°; [α]_546_ −12.6°; [α]_436_ −21.9° (*c* 0.5, chloroform). IR (KBr) $\bar{\nu }$_max_ 3349, 3285, 2117, 1752, 1652, 1598, 1566, 1225, 1062, 1039, 735, 666 cm^−1^. ^1^H NMR (500 MHz, CDCl_3_): δ (ppm) 7.44 (s, 1H, NH-Ph), 7.27 (m, 4H, arom.), 7.06 (t, 1H, arom., 13.5 Hz, *J* 6.5 Hz), 5.70 (d, 1H, NH, *J* 8.5 Hz), 5.29 (t, 1H, H-3, *J* 9.5 Hz), 5.09 (t, 1H, H-4), 4.78 (d, 1H, H-1, *J* 9.5 Hz), 4.26 (dd, 1H, H-6, *J* 12.0 Hz, *J* 5.0 Hz), 4.16 (dd, 1H, H-6′, *J* 2.0 Hz), 3.82 (c, 1H, H-2, *J* 19.0 Hz), 3.77 (m, 1H, H-5), 2.10 (s, 3H, OAc), 2.04 (s, 3H, OAc), 2.02 (s, 3H, OAc). ^13^C{^1^H} NMR (125 MHz, CDCl_3_): δ (ppm) 170.1, 170.7, 169.3 (CO, acetate), 155.6 (CO, urea), 138.0, 129.3 (2C), 124.2, 121.1 (2C) (arom.), 89.0 (C1), 73.8 (C5), 72.5 (C3), 68.3 (C4), 62.0 (C6), 54.9 (C2), 20.7, (OAc), 20.6 (OAc). Anal. Calculated for C_19_H_23_N_5_O_8_: C, 50.78; H, 5.16; N, 15.58. Found: C, 50.71; H, 5.08; N 15.74.

*3,4,6-Tri-O-acetyl-2-deoxy-2-(3-phenylthioureido)-β-d-glucopyranosyl azide* (**15**). Phenyl isothiocyanate (0.180 mL, 1.51 mmol) was added under constant stirring to a cooled (ice bath) solution of **1** (0.50 g, 1.51 mmol) in methylene chloride (4 mL). Immediately, a white crystalline solid separated (0.20 g, 45%), which showed m.p. 149–150 °C. [α]_D_ −44.9°; [α]_578_ −46.8°; [α]_546_ −51.7°; [α]_436_ −83.0° (*c* 0.5, chloroform). IR (KBr) $\bar{\nu }$_max_ 3337, 3297, 3064, 2112, 1750, 1729, 1541, 1238, 1100, 1086, 1040, 951 cm^−1^. ^1^H NMR (500 MHz, CDCl_3_): δ (ppm) 8.15 (s, 1H, NH), 7.45 (t, 2H, arom, *J* 7.5 Hz), 7.35 (m, 1H, arom.), 7.21 (d, 2H, arom.), 5.99 (d, 1H, NH, *J* 9.0 Hz), 5.18 (m, 2H, H-3, and H-4), 4.93 (bs, 1H, H-2), 4.60 (bs, 1H, H-1), 4.25 (dd, 1H, H-6, *J* 12.5 Hz, 5.0 Hz), 4.16 (dd, 1H, H-6′, *J* 2.5 Hz), 3.73 (m, 1H, H-5), 2.10 (s, 3H, OAc), 2.09 (s, 3H, OAc), 2.01 (s, 3H, OAc). ^13^C{^1^H} NMR (125 MHz, CDCl_3_): δ (ppm) 181.8 (CS), 171.1 (CO), 170.6 (CO), 169.1 (CO), 135.5, 130.2 (2C), 127.8, 125.6 (2C) (arom), 88.5 (C1), 74.1 (C5), 72.6 (C3), 67.7 (C4), 61.7 (C6), 58.6 (C2), 20.7 (OAc), 20.6 (OAc), 20.5 (OAc). ^1^H NMR (400 MHz, DMSO-*d*_6_): δ (ppm) 9.82 (s, 1H, NH), 7.66 (d, 1H, NH, *J* 7.6 Hz), 7.36 (t, 2H, arom., *J* 7.6 Hz), 7.27 (d, 2H, arom., *J* 8.0 Hz), 7.17 (t, 1H, arom.), 5.30 (bs, 1H, H-3), 4.95 (t, 3H, H-1, H-2, H-4), 4.21 (dd, 1H, H-6, *J* 12.4 Hz, *J* 4.8 Hz), 4.10 (dd, 1H, H-6′, *J* 2.0 Hz), 3.97 (m, 1H, H-5), 2.04 (s, 3H, OAc), 1.99 (s, 3H, OAc), 1.97 (s, 3H, OAc). ^13^C{^1^H} NMR (100 MHz, DMSO-*d*_6_): δ (ppm) 181.1 (CS), 170.0 (CO), 169.8 (CO), 169.2 (CO), 138.1, 128.8 (2C), 128.2, 123.7 (2C) (arom.), 87.4 (C1), 72.7 (C5), 72.6 (C3), 67.9 (C4), 61.5 (C6), 57.6 (C2), 20.4 (OAc), 20.4 (OAc), 20.3 (OAc). Anal. Calculated for C_19_H_23_N_5_O_7_S: C, 49.03; H, 4.98; N, 15.05; S, 6.89. Found: C, 49.32; H, 5.02; N, 15.15; S, 7.04.

*3,4,6-Tri-O-acetyl-2-deoxy-2-[3-(3-methoxyphenyl)ureido]-β-d-glucopyranosyl azide* (**16**). 3-Methoxyphenyl isocyanate (0.14 mL, 1.1 mmol) was added under constant stirring to a cooled (ice bath) solution of **1** (0.34 g, 1.0 mmol) in methylene chloride (4 mL). The mixture was evaporated, and the residue was treated with ethyl ether and crystallized from benzene, affording a white solid (0.26 g, 52%) that had m.p. 159–160 °C. [α]_D_ −30.5°; [α]_578_ −31.5°; [α]_546_ −35.4°; [α]_436_ −52.4° (*c* 0.5, chloroform). IR (KBr) $\bar{\nu }$_max_ 3322, 3203, 3093, 2121, 1754, 1649, 1610, 1585, 1288, 1223, 1090, 1032 cm^−1^. ^1^H NMR (500 MHz, CDCl_3_): δ (ppm) 7.30 (s, 1H, arom.), 7.21 (t, 1H, arom., *J* 8.5 Hz), 6.98 (t, 1H, NH, *J* 2.5 Hz), 6.83 (dd, 1H, arom., *J* 7.5 Hz, *J* 1.0 Hz), 6.65 (dd, 1H, arom.), 5.54 (d, 1H, NH, *J* 9.0 Hz), 5.30 (t, 1H, H-3, *J* 9.5 Hz), 5.10 (t, 1H, H-4), 4.81 (d, 1H, H-1, *J* 9.5 Hz), 4.28 (dd, 1H, H-6, *J* 12.5 Hz, *J* 5.5 Hz), 4.18 (dd, 1H, H-6′, *J* 2.5 Hz), 3.85 (t, 1H, H-2, *J* 9.0 Hz), 3.81 (m, 1H, H-5), 3.78 (s, 3H, CH_3_), 2.12 (s, 3H, OAc), 2.06 (s, 3H, OAc), 2.04 (s, 3H, OAc). ^13^C{^1^H} NMR (125 MHz, CDCl_3_): δ (ppm) 171.1 (CO), 170.7 (CO), 169.3 (CO), 160.4 (CO), 155.3 (1C), 139.3 (1C), 130.0 (1C), 113.2 (1C), 109.7 (1C), 107.0 (1C) (arom.), 89.0 (C1), 73.8 (C3), 72.6 (C5), 68.3 (C4), 62.0 (C6), 55.2 (C2), 54.8 (CH_3_), 20.7 (OAc), 20.7 (OAc), 20.5 (OAc). Anal. Calculated for C_20_H_25_N_5_ O_9_: C, 50.10; H, 5.26; N, 14.61. Found: C, 49.90; H, 5.25; N, 14.57.

*3,4,6-Tri-O-acetyl-2-deoxy-2-[3-(3-methoxyphenyl)thioureido]-β-d-glucopyranosyl azide* (**17**). 3-Methoxyphenyl isothiocyanate (0.17 mL, 1.2 mmol) was added under constant stirring to a cooled (ice bath) solution of **1** (0.34 g, 1.0 mmol) in methylene chloride (4 mL). The mixture was evaporated, and the residue was treated with ethyl ether and crystallized from benzene, yielding a white solid (0.32 g, 64%) that showed m.p. 108–109 °C. [α]_D_ −30.5°; [α]_578_ −31.5°; [α]_546_ −35.4°; [α]_436_ −45.5° (*c* 0.5, chloroform). IR (KBr) $\bar{\nu }$_max_ 3325, 3154, 3019, 1752, 1732, 1601, 1549, 1233, 1037 cm^−1^. ^1^H NMR (400 MHz, CDCl_3_): δ (ppm) 8.09 (s, 1H, NH), 7.34 (t, 1H, arom., *J* 8.4 Hz), 6.87 (d, 1H, arom., *J* 7.2 Hz), 6.77 (s, 2H, arom.), 6.05 (d, 1H, NH, *J* 9.2 Hz), 5.19 (m, 2H, H-3, and H-4), 4.98 (bs, 1H, H-2), 4.60 (bs, 1H, H-1), 4.26 (dd, 1H, H-6, *J* 12.4 Hz, *J* 4.8 Hz), 4.16 (dd, 1H, H-6′, *J* 2.4 Hz), 3.83 (s, 3H, CH_3_), 3.74 (m, 1H, H-5), 2.11 (s, 3H, OAc), 2.10 (s, 3H, OAc), 2.02 (s, 3H, OAc). ^13^C{^1^H} NMR (100 MHz, CDCl_3_): δ (ppm) 181.5 (C=S), 171.0 (CO), 170.7 (CO), 169.1 (CO), 160.9 (1C), 131.0 (1C), 128.3 (1C), 117.3 (2C), 113.6 (1C), 111.0 (C1) (arom.), 74.0 (C5), 72.6 (C3), 67.7 (C4), 61.7 (C6), 58.6 (C2), 55.5 (CH_3_), 20.7 (OAc), 20.70 (OAc), 20.51 (OAc). Anal. Calculated for C_20_H_25_N_5_ O_8_S: C, 48.48; H, 5.09; N, 14.13; S, 6.47. Found: C, 48.24; H, 5.21; N, 14.11; S, 6.23.

*3,4,6-Tri-O-acetyl-2-deoxy-2-[3-(4-methoxyphenyl)ureido]-β-d-glucopyranosyl azide* (**18**). 4-Methoxyphenyl isocyanate (0.14 mL, 1.2 mmol) was added under constant stirring to a cooled (ice bath) solution of **1** (0.34 g, 1.0 mmol) in methylene chloride (4 mL). A solid precipitated, which was filtered and crystallized subsequently from benzene, affording a white solid (0.22 g, 64%) that showed m.p. 139–140 °C. [α]_D_ −42.7°; [α]_578_ −43.4°; [α]_546_ −49.6°; [α]_436_ −80.3° (*c* 0.6, chloroform);. IR (KBr) $\bar{\nu }$_max_ 3636, 3570, 3343, 3184, 2119, 1732, 1549, 1515, 1227, 1103, 1086 cm^−1^. ^1^H NMR (500 MHz, CDCl_3_): δ (ppm) 7.87 (s, 1H, NH), 7.13 (d, 2H, arom., *J* 9.0 Hz), 6.96 (d, 2H, arom.), 5.74 (d, 1H, NH, *J* 7.5 Hz), 5.18 (t, 1H, H-4, *J* 9.0 Hz), 5.10 (bs, 1H, H-3), 4.93 (bs, 1H, H-2), 4.54 (bs, 1H, H-1), 4.25 (dd, 1H, H-6, *J* 12.5 Hz, *J* 4.5 Hz), 4.16 (dd, 1H, H-6′, *J* 2.5 Hz), 3.83 (s, 3H, CH_3_), 3.72 (m, 1H, H-5), 2.10 (s, 6H, OAc), 2.01 (s, 3H, OAc). ^13^C{^1^H} NMR (125 MHz, CDCl_3_): δ (ppm) 182.3 (CO), 171.0 (CO), 170.6 (CO), 169.0 (CO), 159.4 (1C), 128.0 (2C), 115.3 (2C) (arom.), 88.5 (C1), 74.1 (C5), 72.6 (C3), 67.7 (C4), 61.7 (C6), 58.6 (C2), 55.5 (CH_3_), 20.7 (OAc), 20.7 (OAc), 20.5 (OAc). Anal. Calculated for C_20_H_25_N_5_ O_9_: C, 50.10; H, 5.26; N, 14.61. Found: C, 49.89; H, 5.36; N, 14.57.

*3,4,6-Tri-O-acetyl-2-deoxy-2-[3-(4-methoxyphenyl)tioureido]-β-d-glucopyranosyl azide* (**19**). 4-Methoxyphenyl isothiocyanate (0.14 mL, 1.0 mmol) was added under vigorous stirring to a cooled (ice bath) solution of **1** (0.34 g, 1.0 mmol) in methylene chloride (4 mL). The mixture was evaporated, and the residue was treated with ethyl ether. A solid (0.32 g, 64%) was obtained, which crystallized from benzene and had m.p. 142–143 °C. [α]_D_ −45.4°; [α]_578_ −43.4°; [α]_546_ −49.6°; [α]_436_ −80.3° (*c* 0.5, chloroform). IR (KBr) $\bar{\nu }$_max_ 3636, 3380, 3344, 3186, 2112, 1748, 1548, 1238, 1104, 1040 cm^−1^. ^1^H NMR (400 MHz, CDCl_3_): δ (ppm) 7.90 (s, 1H, NH), 7.13 (d, 2H, arom., *J* 8.8 Hz), 6.95 (d, 2H, arom.), 5.75 (d, 1H, NH, *J* 7.6 Hz), 5.18 (t, 1H, H-3, *J* 9.6 Hz), 5.10 (bs, 1H, H-4), 4.93 (bs, 1H, H-2), 4.55 (bs, 1H, H-1), 4.25 (dd, 1H, H-6, *J* 12.4 Hz, *J* 4.8 Hz), 4.16 (dd, 1H, H-6′, *J* 2.4 Hz), 3.83 (s, 3H, CH_3_), 3.71 (m, 1H, H-5), 2.10 (s, 6H, OAc), 2.01 (s, 3H, OAc). ^13^C{^1^H} NMR (100 MHz, CDCl_3_): δ (ppm) 182.2 (CS), 171.1 (CO), 170.7 (CO), 169.1 (CO), 159.4 (1C), 128.3 (1C), 128.1 (2C), 115.3 (2C) (arom.), 88.5 (C1), 74.1 (C5), 72.6 (C3), 67.6 (C4), 61.7 (C6), 58.6 (C2), 55.5 (CH_3_), 20.8 (OAc), 20.7 (OAc), 20.5 (OAc). Anal. Calculated for C_20_H_25_N_5_ O_8_S: C, 48.48; H, 5.09; N, 14.13; S, 6.47. Found: C, 48.13; H, 5.15; N, 14.11; S, 6.27.

*3,4,6-Tri-O-acetyl-2-deoxy-2-[3-(2-fluorophenyl)tioureido]-β-d-glucopyranosyl azide* (**20**). 2-Fluorophenyl isothiocyanate (0.13 mL, 1.1 mmol) was added under constant stirring to a cooled (ice bath) solution of **1** (0.50 g, 1.5 mmol) in methylene chloride (4 mL). Immediately, a white crystalline solid precipitated (0.31 g, 69%), which showed m.p. 132–133 °C. [α]_D_ −53.1°; [α]_578_ −55.4°; [α]_546_ −63.5°; [α]_436_ −114.6° (*c* 0.5, chloroform). IR (KBr) $\bar{\nu }$_max_ 3331, 3068, 2962, 2929, 2112, 1751, 1729, 1544, 1241, 1100, 1085, 1040 cm^−1^. ^1^H NMR (500 MHz, CDCl_3_): δ (ppm) 7.96 (s, 1H, NH), 7.50 (bs, 1H, arom.), 7.31 (m, 1H, arom.), 7.19 (m, 2H, arom.), 6.18 (bs, 1H, NH), 5.17 (m, 2H, H-3, and H-4), 4.86 (bs, 1H, H-2), 4.66 (bs, 1H, H-1), 4.26 (dd, 1H, H-6, *J* 12.5 Hz, *J* 5.0 Hz), 4.17 (dd, 1H, H-6′, *J* 2.0 Hz), 3.77 (m, 1H, H-5), 2.10 (s, 3H, OAc), 2.07 (s, 3H, OAc), 2.01 (s, 3H, OAc). ^13^C{^1^H} NMR (125 MHz, CDCl_3_): δ (ppm) 181.6 (CS), 171.6 (CO), 170.7 (CO), 169.2 (CO), 144.6 (1C), 143.2 (1C), 125.2 (2C), 123.1 (2C) (arom.), 88.6 (C1), 74.1 (C5), 72.8 (C3), 67.6 (C4), 61.7 (C6), 58.7 (C2), 20.8 (OAc), 20.7 (OAc), 20.5 (OAc). Anal. Calculated for C_19_H_22_N_5_FO_7_S: C, 47.20; H, 4.59; N, 14.49; S, 6.63. Found: C, 47.29; H, 4.73; N, 14.42; S, 6.76.

*3,4,6-Tri-O-acetyl-2-deoxy-2-[3-(4-nitrophenyl)thioureido]-β-d-glucopyranosyl azide* (**21**). 4-Nitrophenyl isothiocyanate (0.18 g, 1.0 mmol) was added under vigorous stirring to a cooled (ice bath) solution of **1** (0.33 g, 1.0 mmol) in methylene chloride (4 mL). The mixture was evaporated and the residue was treated with ethyl ether and crystallized from benzene, yielding a yellow solid (0.32 g, 64%) that had m.p. 100–101 °C. [α]_578_ −14.7°; [α]_546_ −12.8°; [α]_436_ −47.6° (*c* 0.5, chloroform). IR (KBr) $\bar{\nu }$_max_ 3331, 3073, 2119, 1750, 1598, 1539, 1234, 1111, 1046 cm^−1^; ^1^H NMR (500 MHz, CDCl_3_): δ (ppm) 8.61 (s, 1H, NH), 8.21 (d, 2H, arom., *J* 9.5 Hz), 7.57 (d, 2H, arom.), 6.64 (d, 1H, NH, *J* 8.5 Hz), 5.22 (m, 2H, H-3, and H-4), 4.75 (bs, 1H, H-2), 4.31 (dd, 1H, H-6, *J* 12.5 Hz, *J* 4.5 Hz), 4.19 (dd, 1H, H-6′, *J* 2.5 Hz), 3.82 (m, 1H, H-5), 2.12 (s, 3H, OAc), 2.10 (s, 3H, OAc), 2.06 (s, 3H, OAc). ^13^C{^1^H} NMR (125 MHz, CDCl_3_): δ (ppm) 182.5 (CS), 171.4 (CO), 170.7 (CO), 169.1 (CO), 127.7 (1C), 125.0 (1C), 116.8 (2C), 1116.7 (2C) (arom.), 88.6 (C1), 74.0 (C5), 72.5 (C3), 67.8 (C4), 61.7 (C6), 58.7 (C2), 20.7 (OAc), 20.6 (OAc), 20.5 (OAc). HRMS (*m*/*z*) [M+H]^+^: calculated for C_19_H_22_N_6_O_9_S, 510.1050, found 510.1067.

*3,4,6-Tri-O-acetyl-2-deoxy-2-[3-(2-chloro-6-methylphenyl)ureido]-β-d-glucopyranosyl azide* (**22**). 2-Chloro-6-methylphenyl isocyanate (0.07 mL, 0.5 mmol) was added under constant stirring to a cooled (ice bath) solution of **1** (0.16 g, 0.5 mmol) in methylene chloride (4 mL). The mixture was stirred for 24 h, after which a solid (0.13 g, 54%) was obtained, which crystallized from benzene and showed m.p. 220–221 °C. [α]_589_ −21.8°; [α]_578_ −23.0°; [α]_546_ −26.5°; [α]_436_ −44.3° (*c* 0.5, chloroform). IR (KBr) $\bar{\nu }$_max_ 3301, 2120, 1753, 1646, 1597, 1233, 1070, 1036 cm^−1^. ^1^H NMR (500 MHz, DMSO-*d*_6_): δ (ppm) 7.97 (s, 1H, NH), 7.31 (d, 1H, arom., *J* 8.0 Hz), 7.20 (d, 1H, arom., *J* 6.5 Hz), 7.15 (t, 1H, arom.), 6.38 (bs, 1H, NH), 5.23 (t, 1H, H-3, *J* 9.5 Hz), 4.94 (d, 1H, H1, *J* 9.0 Hz), 4.89 (t, 1H, H-4), 4.18 (dd, 1H, H-6, *J* 12.5 Hz, *J* 5.0 Hz), 4.08 (dd, 1H, H-6′, *J* 2.0 Hz), 4.00 (m, 1H, H-5), 3.80 (q, 1H, H-2, *J* 19.5 Hz, *J* 10.0 Hz), 2.18 (s, 3H, CH_3_), 2.03 (s, 3H, OAc), 1.98 (s, 3H, OAc), 1.96 (s, 3H, OAc). ^1^H NMR (400 MHz, CDCl_3_): δ (ppm) 7.31 (m, 1H, arom.), 7.17 (m, 1H, arom.), 6.24 (bs, 1H, NH), 5.26 (t, 1H, H-3, *J* 9.6 Hz), 5.07 (t, 1H, H-4), 4.81 (d, 1H, NH, *J* 8.8 Hz), 4.75 (bs, 1H, H-1), 4.26 (dd, 1H, H-6, *J* 12.4 Hz, *J* 4.8 Hz), 4.15 (dd, 1H, H-6′, *J* 2.0 Hz), 3.75 (m, 2H, H-2, and H-5), 2.31 (s, 3H, CH_3_), 2.10 (s, 3H, OAc), 2.04 (s, 3H, OAc), 2.01 (s, 3H, OAc). ^13^C{^1^H} NMR (100 MHz, DMSO-*d*_6_): δ (ppm) 170.0 (CO), 169.5 (CO), 169.2 (CO), 154.7 (CO), 138.7 (1C), 137.2 (1C), 134.1, 128.9 (2C), 126.7 (2C), 88.0 (C1), 72.6 (C5), 72.3 (C3), 68.3 (C4), 61.7 (C6), 53.4 (C2), 20.4 (OAc), 20.3 (2OAc), 18.1 (CH_3_). Anal. Calculated for C_20_H_24_ClN_5_ O_8_: C, 48.25; H, 4.86; N, 14.07; Cl, 7.12. Found: C, 48.10; H, 4.66; N, 14.17.

*3,4,6-Tri-O-acetyl-2-deoxy-2-isothiocianato-β-d-glucopyranosyl azide* (**23**). To a vigorously stirred mixture of **1** (0.22 g, 0.67 mmol) in chloroform (5 mL), calcium carbonate (0.17 g, 0.68 mmol) and water (2 mL) was added thiophosgene (0.06 mL, 0.78 mmol). Stirring was maintained at room temperature for 48 h. After this time, the mixture was filtered and extracted with chloroform. The filtrate was washed with water. The organic phase was dried over calcium chloride and evaporated to dryness, yielding an orange residue from which a solid separates when treating with ethyl ether (0.15 g, 58%). Crystallized from benzene it showed m.p. 88–89 °C. [α]_589_ 14.3°; [α]_578_ 15.0°; [α]_546_ 17.9°; [α]_436_ 33.9° (*c* 0.5, chloroform). IR (KBr) $\bar{\nu }$_max_ 2120, 2077, 1750, 1739, 1239, 1218, 1062, 1033 cm^−1^. ^1^H NMR (400 MHz, CDCl_3_): δ (ppm) 5.20 (t, 1H, H-3, *J* 10.0 Hz), 4.99 (t, 1H, H-4), 4.75 (d, 1H, H-1, *J* 8.8 Hz), 4.28 (dd,1H, H-6, *J* 12.8 Hz, *J* 4.8 Hz), 4.13 (dd, 1H, H-6′, *J* 2.4 Hz), 3.80 (m, 1H, H-5), 3.74 (dd, 1H, H-2, *J* 10.4 Hz, *J* 9.2 Hz), 2.10 (s, 3H, OAc), 2.09 (s, 3H, OAc), 2.03 (s, 3H, OAc). ^13^C{^1^H} NMR (100 MHz, CDCl_3_): δ (ppm) 170.5 (CO), 169.7 (CO), 169.4 (CO), 141.9 (CS), 88.5 (C1), 74.1 (C5), 72.6 (C3), 67.4 (C4), 61.4 (C6), 60.2 (C2), 20.7 (OAc), 20.6 (OAc), 20.5 (OAc). Anal. Calculated for C_13_H_16_N_4_ O_7_S: C, 41.93; H, 4.33; N, 15.05; S, 8.61. Found: C, 42.01; H, 4.01; N, 15.00; S, 8.41.

*3,4,6-Tri-O-acetyl-2-deoxy-2-[3-(2-chloro-6-methylphenyl)thioureido]-β-d-glucopyranosyl azide* (**25**). 2-Chloro-6-methylaniline (0.03 mL, 0.27 mmol) was added under constant stirring to a cooled (ice bath) solution of **23** (0.10 g, 0.27 mmol) in methylene chloride (4 mL). The mixture was kept at room temperature for 72 h, after which it was evaporated to dryness. When the resulting orange residue was treated with ethyl ether/petroleum ether, a solid (0.031 g, 22%) was obtained, which crystallized from benzene and had m.p. 164–165 °C. IR (KBr) $\bar{\nu }$_max_ 3331, 2118, 1750, 1730, 1241, 1104, 1088, 1053 cm^−1^. ^1^H NMR (500 MHz, CDCl_3_, 330 K): δ (ppm) 7.51 (bs, 1H, NH), 7.35 (d, 1H, arom., *J* 7.5 Hz), 7.23 (m, 2H, arom.), 5.51 (bs, 1H, NH), 5.12 (d, 1H, H-1, *J* 4.0 Hz), 4.76 (bs, 1H, H-3), 4.59 (bs, 1H, H-4), 4.24 (dd, 1H, H-6, *J* 12.5 Hz, *J* 5.0 Hz), 4.17 (dd, 1H, H-6′, *J* 2.5 Hz), 3.73 (bs, 1H, H-5), 2.31 (s, 3H, CH_3_), 2.08 (s, 3H, OAc), 2.05 (s, 3H, OAc), 1.98 (s, 3H, OAc). ^13^C{^1^H} NMR (100 MHz, CDCl_3_, 223 K): δ (ppm) 180.7 (CS), 171.3 (CO), 171.2 (CO), 169.3 (CO), 139.4 (1C), 133.6 (1C), 130.3 (1C), 130.0 (1C), 129.9 (1C), 127.9 (1C) (arom.), 88.2 (C1), 73.3 (C5), 71.3 (C3), 66.2 (C6), 70.0 (C4), 57.9 (C2), 21.0 (OAc), 20.9 (OAc), 18.3 (OAc). HRMS (*m*/*z*) [M+Na]^+^: calculated for C_20_H_24_O_7_N_5_ClNaS 536.0965, found 536.0977.

*Deacetylation reaction. General procedure.* To a solution of the per-*O*-acetyl ureido derivative (0.25 mmol) in methanol (4 mL) was added a saturated solution of ammonia in methanol. The reaction was monitored by TLC (silica gel plate) using benzene/methanol (9:1) as eluent.

*2-Deoxy-2-(3-phenylureido)-β-d-glucopyranosyl azide* (**34**). Starting from **14** and according to the general procedure, the reaction mixture was evaporated to dryness and the crude product (81%) was crystallized from ethanol and had m.p. 205–206 °C. IR (KBr) $\bar{\nu }$_max_ 3445, 3351, 3276, 2120, 1619, 1596, 1562, 1237, 1084, 1029 cm^−1^. ^1^H NMR (500 MHz, DMSO-*d_6_*): δ (ppm) 8.52 (s, 1H, NH), 7.40 (d, 2H, arom., *J* 8.0 Hz), 7.22 (t, 2H, arom., *J* 7.5 Hz), 6.90 (t, 1H, arom.), 6.12 (d, 1H, NH, *J* 9.0 Hz), 5.15 (d, 2H, OH-3, and OH-4, *J* 5.5 Hz), 4.67 (t, 1H, OH-6, *J* 6.0 Hz), 4.51 (d, 1H, H-1, *J* 9.0 Hz), 3.72 (ddd, 1H, H-6, *J* 12.5 Hz, *J* 5.5 Hz), 3.50 (ddd, 1H, H-6′), 3.45 (c, 1H, H-2), 3.30 (m, 2H, H-4, and H-5), 3.15 (td, 1H, H-3, *J* 9.5 Hz, *J* 5.5 Hz). ^13^C{^1^H} NMR (125 MHz, DMSO-*d_6_*): δ (ppm) 155.0 (CO), 140.3, 128.6 (2C), 121.0, 117.6 (2C) (arom.), 88.9 (C1), 79.1 and 74.1 (C4, C5), 70.2 (C3), 60.7 (C6), 55.3 (C2). HRMS (*m*/*z*) [M+Na]^+^: calculated for C_13_H_17_O_5_N_5_Na 346.1114, found 346.1122.

*2-Deoxy-2-[3-(4-methoxyphenyl)ureido]-β-d-glucopyranosyl azide* (**35**). Starting from **15** and in agreement with the general procedure, the title compound crystallized from the reaction mixture. It was filtered, washed with ethyl ether (23%), recrystallized from ethanol, and showed m.p. 214–215 °C. IR (KBr) $\bar{\nu }$_max_ 3470, 3305, 3108, 2110, 1641, 1597, 1561, 1488, 1240, 1090, 1054 cm^−1^. ^1^H NMR (400 MHz, DMSO-*d_6_*): δ (ppm) 8.55 (s, 1H, NH), 7.13 (m, 2H, arom.), 6.88 (d, 1H, arom., *J* 7.6 Hz), 6.48 (dd, 1H, arom., *J* 8.0 Hz, *J* 2.4 Hz), 6.11 (d, 1H, NH, *J* 8.8 Hz), 5.16 (m, 2H, OH-3, and OH-4), 4.69 (t, 1H, OH-6, *J* 5.6 Hz), 4.51 (d, 1H, H-1, *J* 9.0 Hz), 3.72 (m, 1H, H-6), 3.70 (s, 3H, CH_3_), 3.47 (m, 2H, H-2, and H-6′), 3.30 (m, 2H, H-4, and H-5), 3.14 (td, 1H, H-3, *J* 8.8 Hz, *J* 5.2 Hz). ^13^C{^1^H} NMR (100 MHz, DMSO-*d_6_*): δ (ppm) 159.5 (CO), 154.9 (1C), 141.5 (1C), 129.3 (1C), 110.0 (1C), 106.4 (1C), 103.5 (1C) (arom.), 88.8 (C1), 79.1 (C4 ó C5), 74.1 (C4 ó C5), 70.2 (C3), 60.7 (C6), 55.3 (C2), 54.8 (CH_3_). HRMS (*m*/*z*) [M+Na]^+^: calculated for C_14_H_19_O_6_N_5_Na 373.1217, found 373.1228.

*2-Deoxy-2-[3-(2-chloro-6-methylphenyl)ureido]-β-d-glucopyranosyl azide* (**36**). Starting from **22** and in agreement with the general procedure, the title compound crystallized from the reaction mixture. It was filtered, washed with ethyl ether (79%), recrystallized from ethanol, and showed m.p. 206–207 °C. IR (KBr) $\bar{\nu }$_max_ 3300, 2227, 2121, 1638, 1567, 1468, 1455, 1248, 1073, 1035 cm^−1^. ^1^H NMR (400 MHz, DMSO-*d*_6_): δ (ppm) 7.81 (s, 1H, NH), 7.29 (d, 1H, arom., *J* 8.0 Hz), 7.18 (d, 1H, arom., *J* 7.2 Hz), 7.13 (t, 1H, arom.), 6.31 (d, 1H, NH, *J* 8.4 Hz), 5.13 (d, 1H, OH, *J* 5.6 Hz), 5.09 (d, 1H, OH), 4.67 (t, 1H, OH-6, *J* 5.6 Hz), 4.46 (d, 1H, H-1, *J* 8.8 Hz), 3.72 (dd, 1H, H-6, *J* 10.4 Hz, *J* 5.6 Hz), 3.46 (m, 2H, H-6′, and H-2), 3.30 (m, 2H, H-4, and H-5), 3.13 (td, 1H, H-3, *J* 8.8 Hz, *J* 5.2 Hz). ^13^C{^1^H} NMR (100 MHz, DMSO-*d*_6_): δ (ppm) 155.4 (CO), 138.5 (1C), 134.6 (1C), 131.5 (1C), 128.9 (1C), 126.7 (1C), 126.6 (1C) (arom.), 89.2 (C1), 79.2 and 74.1 (C4, C5), 70.4 (C3), 60.8 (C6), 55.8 (C2), 18.4 (CH_3_). HRMS (*m*/*z*) [M+Na]^+^: calculated for C_14_H_18_O_5_N_5_ClNa 394.0885, found 394.0889.

## Figures and Tables

**Table 1:** ^1^H NMR data (δ, ppm) for the aminosugar moiety of **1**, **14**–**23**, and **25**.

Compound	NHAr	NH	H-1	H-2	H-3	H-4	H-5	H-6	H-6′
**1** ^b^		1.50 s	4.54 d	2.83 t	5.03 t	4.97 t	3.78 ddd	4.30 dd	4.14 dd
**14** ^a^	7.44 s	5.70 d	4.78 d	3.82 q	5.29 t	5.09 t	3.76 m	4.26 dd	4.15 dd
**15** ^a^	8.15 s	5.99 d	4.60 bs	4.93 bs	5.18 m		3.73 m	4.25 dd	4.16 dd
**16** ^a^	6.98 t	5.54 d	4.81 d	3.85 t	5.30 t	5.10 t	3.81 m	4.28 dd	4.18 dd
**17** ^b^	8.09 s	6.05 d	4.60 bs	4.98 m	5.19 m		3.74 m	4.26 dd	4.16 dd
**18** ^a^	7.87 s	5.74 d	4.54 bs	4.93 bs	5.10 bs	5.18 t	3.72 m	4.25 dd	4.15 dd
**19** ^b^	7.90 s	5.75 d	4.55 bs	4.93 bs	5.18 t	5.10 bs	3.71 m	4.25 dd	4.16 dd
**20** ^a^	7.96 s	6.18 bs	4.66 bs	4.86 bs	5.17 m		3.77 m	4.26 dd	4.17 dd
**21** ^a^	8.61 s	6.64 dd	4.75 bs		5.22 m		3.82 m	4.31 dd	4.19 dd
**22** ^c^	7.97 s	6.38 bs	4.94 d	3.80 q	5.23 t	4.89 t	4.00 m	4.18 dd	4.08 dd
**23** ^b^			4.75 d	3.74 d	5.20 t	5.00 t	3.80 m	4.28 dd	4.13 dd
**25** ^a,d^	7.51 s	5.51 bs	5.12 d	---	4.76 bs	4.59 bs	3.73 bs	4.24 dd	4.17 dd

^a^ In CDCl_3_ at 500 MHz. ^b^ In CDCl_3_ at 400 MHz. ^c^ In DMSO-*d_6_* at 500 MHz. ^d^ At 330 K.

**Table 2:** ^13^C NMR data (δ, ppm) of **1**, **14**–**23** and **25**.

Compound	CO ^d^	CS	C-1	C-2	C-3	C-4	C-5	C-6
**1** ^b^	170.6	---	91.8	55.8	73.9	68.2	75.1	61.9
**14** ^a^	155.6	---	89.0	54.9	72.6	68.3	73.8	62.0
**15** ^a^	---	181.2	88.5	58.6	72.6	67.7	74.1	61.7
**16** ^a^	155.3	---	89.0	54.8	72.6	68.3	73.8	62.0
**17** ^b^	---	181.5	88.9	58.6	72.6	67.7	74.0	62.0
**18** ^a^	159.4	---	88.5	58.6	72.7	67.7	74.1	61.7
**19** ^b^	---	182.2	88.5	58.6	72.6	67.7	74.1	61.7
**20** ^a^	---	181.6	88.6	58.8	72.9	67.6	74.1	61.7
**21** ^a^	---	182.5	88.6	58.7	72.5	67.8	74.0	61.7
**22** ^c^	154.7	---	88.0	53.4	72.3	68.3	72.6	62.0
**23** ^b^	---	141.9	88.5	60.2	72.6	67.4	74.1	61.4
**25** ^b,e^	---	180.70	88.2	57.9	71.3	61.0	73.3	67.2

^a^ In CDCl_3_ at 125 MHz. ^b^ In CDCl_3_ at 100 MHz. ^c^ In DMSO-*d_6_* at 100 MHz. ^d^ Urea. ^e^ At 223 K.

**Table 3:** NMR coupling constants (Hz) for **1** and **14**–**22**.

Compound	*J*_NH,H-2_	*J*_1,2_	*J*_2,3_	*J*_3,4_	*J*_4,5_	*J*_5,6_	*J*_5,6′_	*J*_6,6′_
**1** ^a^	---	9.0	9.5	9.5	9.5	5.0	2.0	12.0
**14** ^a^	8.5	9.5	9.5	9.5	10.0	5.0	1.5	12.0
**15** ^a^	9.0	---	---	---	9.0	5.0	2.5	12.5
**16** ^a^	9.0	9.5	9.5	9.5	10.0	5.5	2.5	12.5
**17** ^b^	11.5	---	---	---	9.0	6.0	3.0	15.5
**18** ^a^	7.5	---	---	9.0	10.0	4.5	2.5	12.5
**19** ^b^	7.6	---	---	9.6	9.6	4.8	2.4	12.4
**20** ^a^	---	---	---	---	9.5	5.0	2.0	12.5
**21** ^a^	---	---	---	---	9.5	4.5	2.5	12.5
**22** ^c^	---	9.5	10.0	9.5	10.0	5.0	2.5	12.5

^a^ In CDCl_3_ at 500 MHz. ^b^ In CDCl_3_ at 400 MHz. ^c^ In DMSO-*d_6_* at 500 MHz.

**Table 4:** Relative electronic and Gibbs free energies calculated for conformers of compounds **14** and **15** ^a^.

	14				15			
Conformer	ΔE ^d^	ΔG ^d^	ΔE ^e^	ΔG ^e^	ΔE ^d^	ΔG ^d^	ΔE ^e^	ΔG ^e^
*Z*,*Z* ^b^	0.0	0.0	0.0	0.0	3.9	3.2	3.0	2.8
*Z*,*E* ^b^	0.6	0.1	1.2	3.2	0.0	0.0	0.0	0.0
*E*,*Z* ^b^	3.8	4.7	3.3	5.9	5.6	5.4	3.5	3.3
*Z*,*Z* ^c^	0.0	0.0	0.0	0.0	3.9	2.5	2.3	2.1
*Z*,*E* ^c^	0.6	1.0	1.1	0.9	0.0	0.0	0.0	0.0
*E*,*Z* ^c^	4.3	4.8	3.4	3.7	6.4	5.1	4.4	3.9

^a^ In kcal/mol. ^b^ M062X/6-311G(d,p). ^c^ M062X/def2-TZVP. ^d^ In the gas phase. ^e^ In CHCl_3_.

**Table 5:** Selected bond distances (Å) and dihedral angles (°) for conformers of **14**.

	(*Z*,*Z*) ^a^	(*Z*,*E*) ^a^	(*E*,*Z*) ^a^	(*Z*,*Z*) ^b^	(*Z*,*E*) ^b^	(*E*,*Z*) ^b^
O38—H48	2.19	---	2.20	2.21	---	2.20
N39—N18—C37—O38	−8.59	−7.67	---	-8.23	−7.58	---
O38—C37—C44—C45	4.70	---	9.36	5.25	---	9.36
O38—C37—N43—C44	−0.74	−179.55	6.43	−1.40	173.76	−2.75
H42—N41—C2—H10	133.32	137.16	164.24	136.67	139.35	162.07

^a^ At the M062X/6-311G(d,p) level. ^b^ At the M062X/def2-TZVP level.

**Table 6:** Selected bond distances (Å) and dihedral angles (°) for conformers of **15**.

	(*Z*,*Z*) ^a^	(*Z*,*E*) ^a^	(*E*,*Z*) ^a^	(*Z*,*Z*) ^b^	(*Z*,*E*) ^b^	(*E*,*Z*) ^b^
S55—H47	2.82	---	2.96	2.78	---	2.86
N38—N18—C37—S55	−8.14	−1.81	---	-7.08	-1.06	---
S55—C37—C43—C44	40.64	---	53.47	39.98	---	50.74
S55—C37—N42—C43	−1.17	−159.95	13.86	0.31	−164.02	16.66
H41—N40—C2—H10	145.29	159.38	160.72	147.66	162.65	160.44

^a^ At the M062X/6-311G(d,p) level. ^b^ At the M062X/def2-TZVP level.

**Table 7:** Relative energy minima (in kcal/mol) and their dihedral angles (in °) found for **25** ^a^.

		Gas Phase				Chloroform			
Entry		ΔE ^b^	ΔG ^b^	θ_N-C(S)-N-Carom_	θ_N-C(S)-N-C2_	ΔE ^c^	ΔG ^c^	θ_N-C(S)-N-Carom_	θ_N-C(S)-N-C2_
1	min 1	2.1	0.0	178.4	161.0	2.9	1.9	187.7	176.5
2	min 2	0.0	−0.1	357.1	176.2	0.0	0.0	355.8	176.9

^a^ At the M062X/6-311G(d,p). ^b^ In the gas phase. ^c^ In CHCl_3_.

**Table 8:** Relative energy minima (in kcal/mol) and their dihedral angles (in °) found for **25** ^a^.

		Gas Phase				Chloroform			
Entry		ΔE ^b^	ΔG ^b^	θ_N-C(S)-N-Carom_	θ_N-C(S)-N-C2_	ΔE ^c^	ΔG ^c^	θ_N-C(S)-N-Carom_	θ_N-C(S)-N-C2_
1	min 1	0.6	−1.0	180.8	164.2	1.5	1.6	187.0	176.3
2	min 2	0.0	0.0	358.1	176.7	0.0	0.0	356.7	177.7

^a^ At the M062X/def2-TZVP level. ^b^ In the gas phase. ^c^ In CHCl_3_.

**Table 9:** Relative energy maxima (in kcal/mol) and their dihedral angles (in °) found for **25** ^a^.

	∆E	∆G	θ_N-C(S)-N-Carom_	θ_N-C(S)-N-C2_	ṽ ^‡ b^
max	11.2	12.2	296.9	165.2	−32.49

^a^ At the M062X/6-311G(d,p) level in the gas phase. ^b^ Imaginary frequencies for the transition structure (‡) in cm^−1^.

**Table 10:** Stationary points for the rotational interconversion of thiourea **33** ^a^.

		Gas Phase				Chloroform			
Entry		ΔE ^b^	ΔG ^b^	θ_C2-C1-N-C(S)_ ^c^	ṽ ^‡ d^	ΔE ^b^	ΔG ^b^	θ_C2-C1-N-C(S)_ ^c^	ṽ ^‡ d^
1	min 1	0.00	0.00	70.90		0.00	0.00	74.95	
2	min 2	3.05	3.85	279.66		2.38	1.98	273.72	
3	max 1	11.65	13.64	206.91	−66.19	12.38	13.78	205.32	−69.18
4	max 2	10.61	13.46	331.35	−73.53	11.88	14.29	331.15	−70.93

^a^ At the M062X/6-311G(d,p) in the gas phase. ^b^ In kcal/mol. ^c^ In degrees. ^d^ Imaginary frequencies for the transition structure (‡) in cm^−1^.

**Table 11:** ^1^H NMR data (δ, ppm) of **34**–**36** ^a^.

Compound	NHAr	NH	H-1	H-2	H-3	H-4/H-5	H-6	H-6′
**34** ^b^	8.52 s	6.12 d	4.51 d	3.45 c	3.15 td	3.30 m	3.72 dd	3.50 dd
**35** ^a^	8.55 s	6.11 d	4.51 d	3.47 m	3.14 td	3.30 m	3.72 m	3.47 m
**36** ^a^	7.81 s	6.31 d	4.46 d	3.46 m	3.13 td	3.30 m	3.72 dd	3.46 m

^a^ In DMSO-*d_6_* at 400 MHz. ^b^ In DMSO-*d_6_* at 500 MHz.

**Table 12:** ^13^C NMR data (δ, ppm) of **34**–**36** ^a^.

Compound	CO	C1	C2	C3	C4	C5	C6	CH_3_
**34** ^b^	155.0	88.9	55.3	70.2	79.2	74.1	60.8	---
**35** ^a^	159.6	88.8	55.3	70.2	79.2	74.1	60.7	54.8
**36** ^a^	155.4	89.2	55.8	70.4	79.3	74.1	60.8	18.4

^a^ In DMSO-*d_6_* at 100 MHz. ^b^ In DMSO-*d_6_* at 125 MHz.

## Data Availability

All data can be obtained from the authors upon reasonable request. This manuscript is part of Ph.D. theses, by one of us (C.S.-G.) under a Creative Commons Licence, which are available at https://dehesa.unex.es/handle/10662/6467, from institutional repositories. (Accessed on 4 November 2024).

## Supplementary Materials

The following supporting information can be downloaded at: https://www.mdpi.com/article/10.3390/molecules29235687/s1, FTIR and NMR spectra, and computational data mentioned through the main text.
