# Supplementary material for: Bifunctional Azido(thio)ureas from an O-Protected 2-Amino-2-deoxy-d-glucopyranose: Synthesis and Structural Analyses
Source: Molecules. 2024 Nov 30;29(23):5687. doi: 10.3390/molecules29235687 (PMC11643871; doi:10.3390/molecules29235687)
Supplement: Supplementary file 1 [file molecules-29-05687-s001.zip › molecules-3335596-supplementary.pdf]

Supplementary Materials

Bifunctional azido(thio)ureas from an *O*-protected 2-amino-2-deoxy-D-glucopyranose: synthesis and structural analyses

Concepción Sosa-Gil,[a] Esther Matamoros,[a,b,c,]* Pedro Cintas[a] and Juan C. Palacios.[a,]*

[a]*Departamento de Química Orgánica e Inorgánica, Facultad de Ciencias, and Instituto del Agua, Cambio Climático y Sostenibilidad (IACYS), Universidad de Extremadura, 06006 Badajoz, Spain*

[b]*Departamento de Química Orgánica, Universidad de Málaga, Campus Teatinos s/n, 29071 Málaga, Spain.*

[c]*Instituto de Investigación Biomédica de Málaga y Plataforma en Nanomedicina – IBIMA, Plataforma Bionand, Parque Tecnológico de Andalucía, 29590 Málaga, Spain*

E-mail: esthermc@unex.es; palacios@unex.es

Summary

Pages	Supplementary information
2	Tables
3	IR spectra (Fig. S1-S15)
11	NMR spectra (Fig. S16-S72)
40	Mass spectra (Fig. S73-S77)
43	Cartesian coordinates and calculated energies at the M06-2X/6-311G(d,p) and M06-2X/def2-TZVP level in gas phase and $CHCl_3$ (SMD Model)

Table S1. Yield (%) and IR spectroscopic data (cm^{-1}) of **14**-**22**

Comp.	Yield	$\bar{\upsilon}_{NH}$	$\bar{\upsilon}_{N=N=N}$	$\bar{\upsilon}_{C=O}$[a]	$\bar{\upsilon}_{C=O}$[b]	δ_{NH}[c]	$\bar{\upsilon}_{C\text{-}O\text{-}C}$[a]	$\bar{\upsilon}_{C\text{-}O}$
**14**	97	3349/3285	2117	1751	1652	1566	1225	1062
**15**	45	3337/3297	2112	1750	1598	1541	1238	1086
**16**	52	3322	2121	1754	1649	1585	1223	1090
**17**	65	3325/3154	2118	1752	1601	1549	1233	1081
**18**	64	3343/3184	2119	1732	1611	1549	1228	1085
**19**	64	3345/3182	2120	1731	1612	1550	1227	1086
**20**	69	3331	2113	1752	1597	1544	1241	1086
**21**	64	3331	2119	1750	1598	1569	1234	1087
**22**	54	3301	2118	1750	1729	1540	1241	1088

[a]Acetate. [b]Urea amide I band. [c]Urea amide II band.

Table S2. ^{1}H NMR data (δ, ppm) of **14**-**22**

Comp.	Ar-1	Ar-2	Ar-3	Ar-4	Ar-5
**14**[a]			7.27 m/7.06m		
**15**[a]	7.21 d	7.45 t	7.35 m	7.45 t	7.21 d
**16**[a]	7.30 s	6.83 dd	7.21 t	6.65 dd	---
**17**[b]	6.77 m	6.87 d	7.34 t	6.77 m	---
**18**[a]	7.13 d	6.96 d	6.96 d	7.13 d	---
**19**[b]	7.13 d	6.95 d	6.95 d	7.13 d	---
**20**[a]	7.30 sa	7.31 m	7.19 m		---
**21**[a]	8.21 d	7.57 d	7.57 d	8.21 d	---
**22**[c]	7.31 d	7.20 d	7.15 t		---

[a]In $CDCl_3$ at 500 MHz. [b]In $CDCl_3$ at 400 MHz. [c]In DMSO-d_6 at 500 MHz.

Table S3. Yield (%) and IR spectroscopic data of **34**-**36**

Urea	Yield	$\bar{\upsilon}_{OH}$	$\bar{\upsilon}_{NH}$	$\bar{\upsilon}_{N=N=N}$	$\bar{\upsilon}_{C=O}$[a]	$\bar{\upsilon}_{C=O}$[b]	δ_{NH}[c]	$\bar{\upsilon}_{C\text{-}O}$
**34**	40	3276	3351	2120	1619	1596	1561	1084
**35**	23	3304	3470	2110	1641	1598	1561	1090
**36**	79	3299	3299	2121	1638	1657	---	1073

[a]Acetate. [b]Urea I amide band. [c] Urea amide II band.

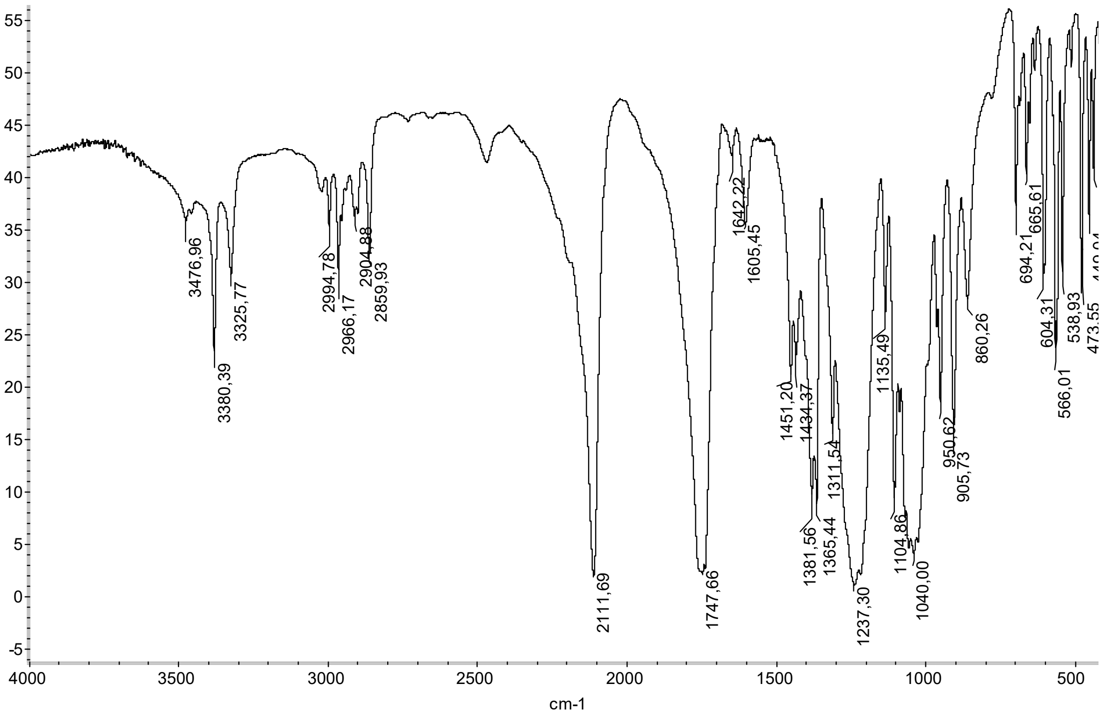

Figure S1 FT-IR spectrum of 3,4,6-tri-O-acetyl-2-amino-2-deoxy-β-D-glucopyranosyl azide (**1**).

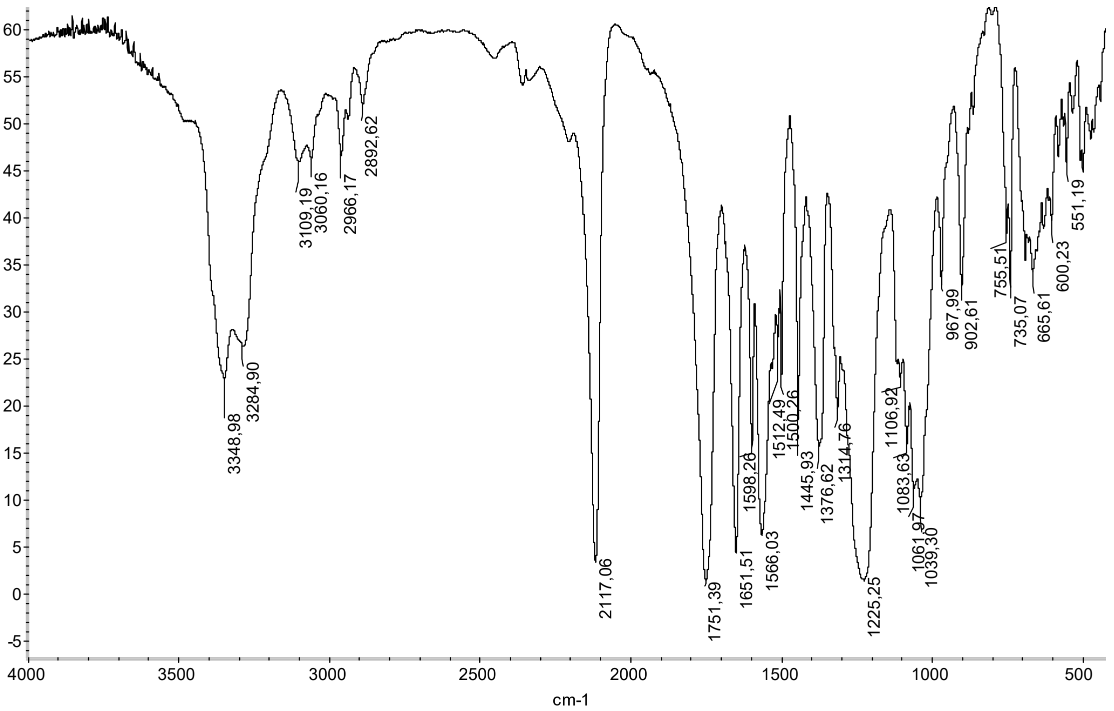

Figure S2 FT-IR spectrum of 3,4,6-tri-*O*-acetyl-2-deoxy-2-(3-phenylureido)-β-D-glucopyranosyl azide (**14**).

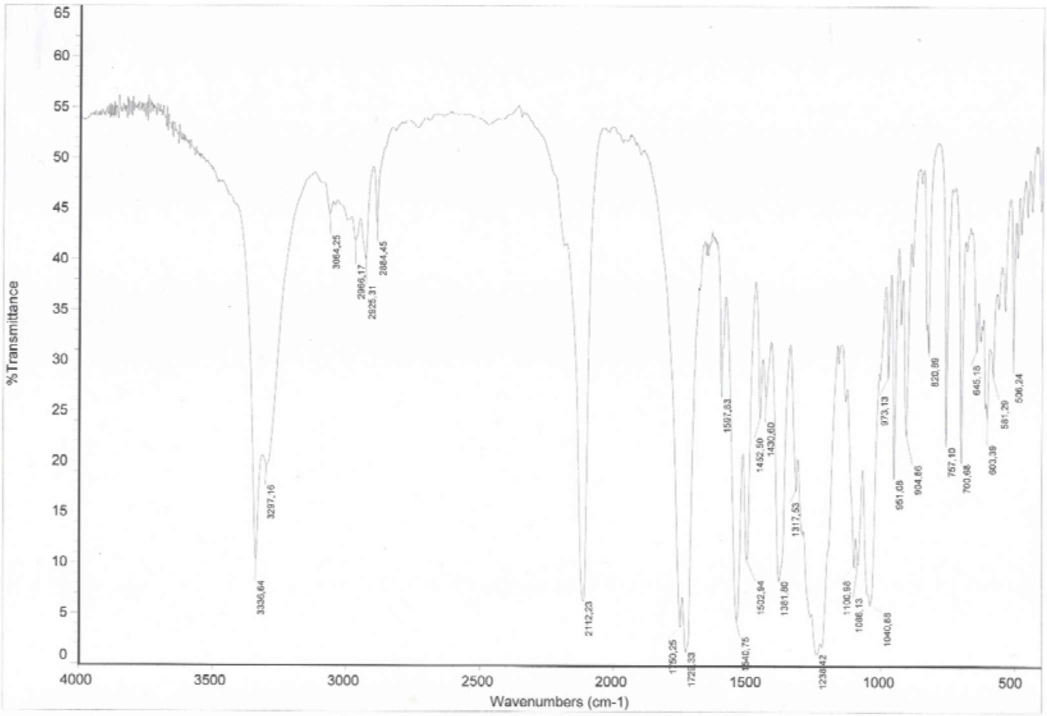

Figure S3 FT-IR spectrum of 3,4,6-tri-*O*-acetyl-2-deoxy-2-(3-phenylthioureido)-β-D-glucopyranosyl azide (**15**).

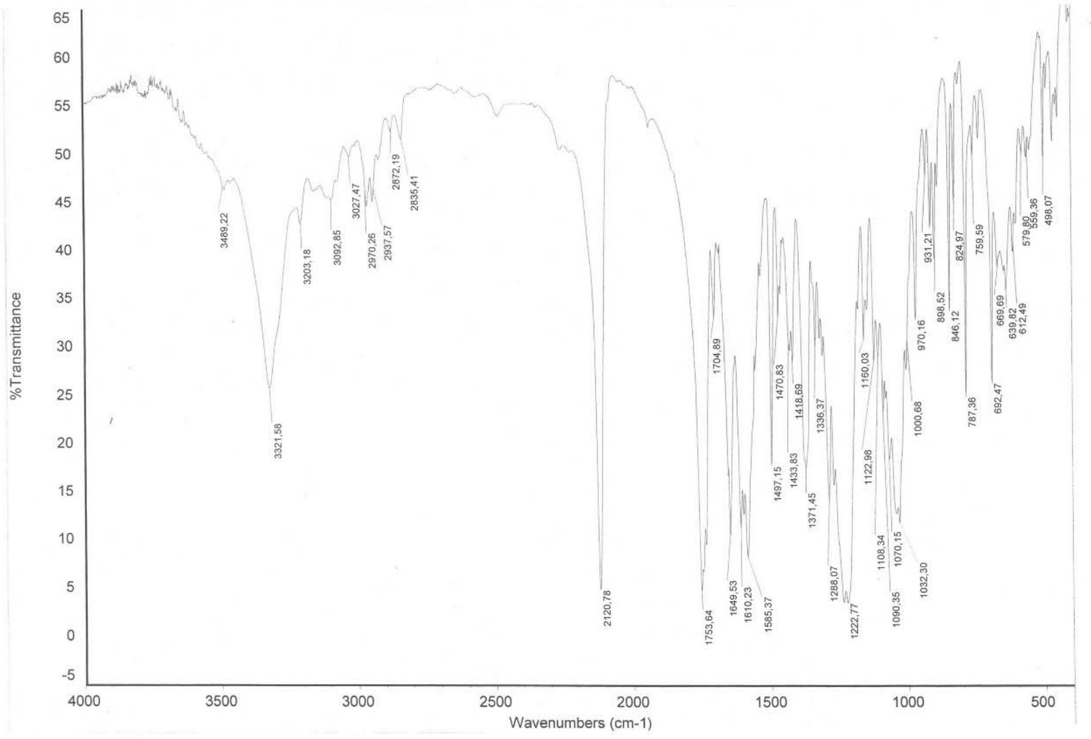

Figure S4 FT-IR spectrum of 3,4,6-tri-*O*-acetyl-2-deoxy-2-[3-(3-methoxyphenyl)ureido]-β-D-glucopyranosyl azide (**16**).

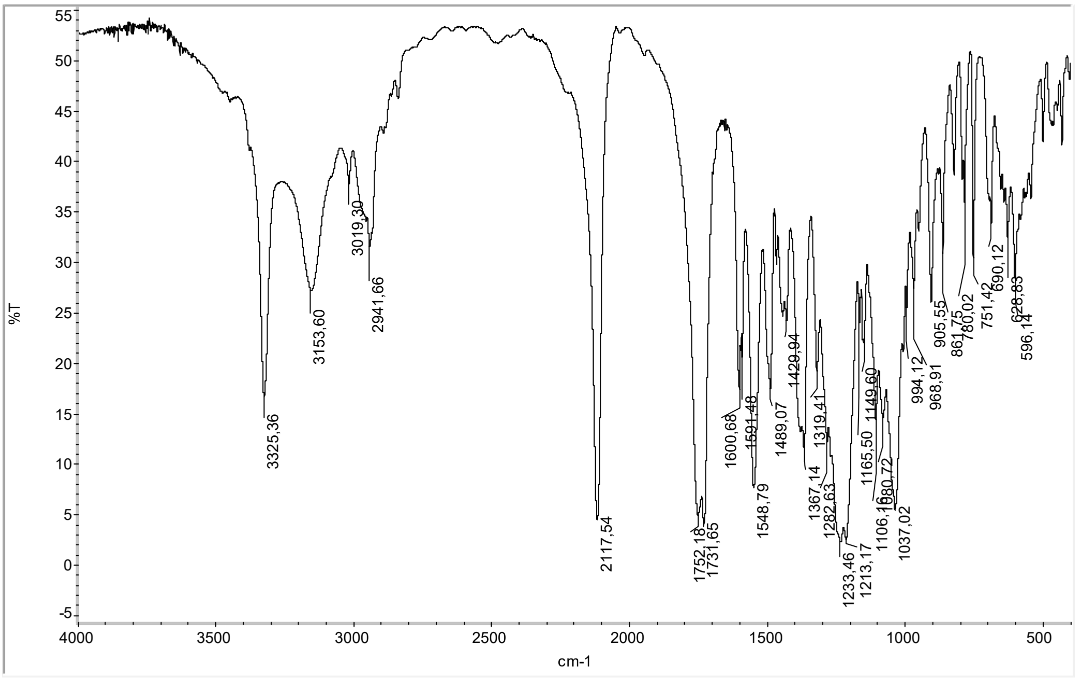

Figure S5 FT-IR spectrum of 3,4,6-tri-*O*-acetyl-2-deoxy-2-[3-(3-methoxyphenyl)thioureido]-β-D-glucopyranosyl azide (**17**).

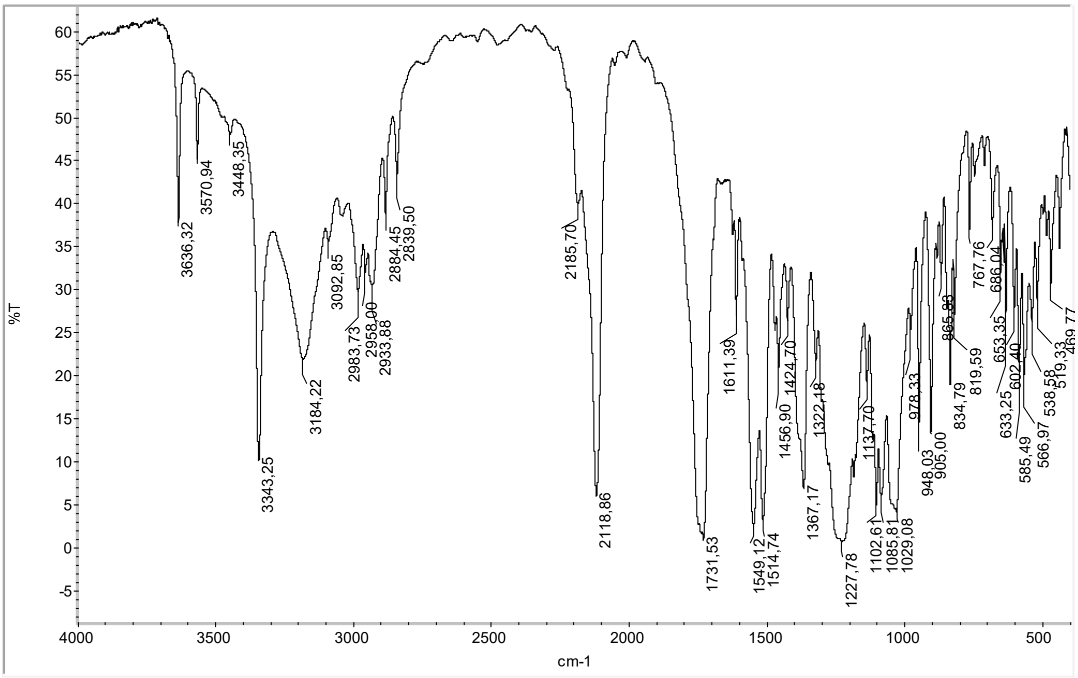

Figure S6 FT-IR spectrum of 3,4,6-tri-*O*-acetyl-2-deoxy-2-[3-(4-methoxyphenyl)ureido]-β-D-glucopyranosyl azide (**18**).

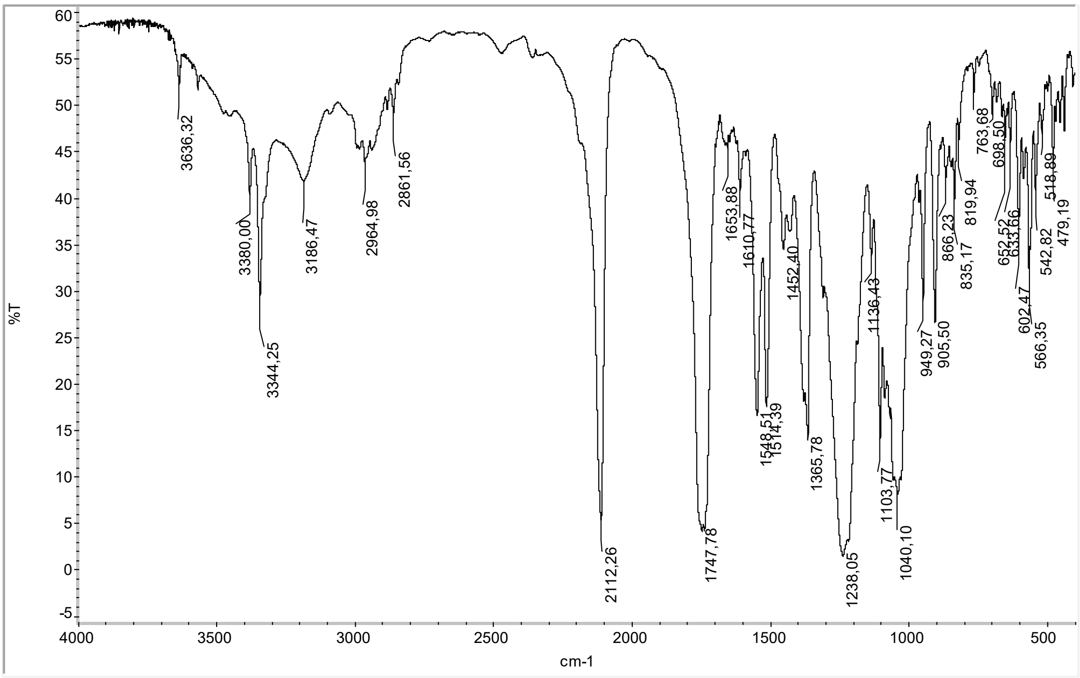

Figure S7 FT-IR spectrum of 3,4,6-tri-*O*-acetyl-2-deoxy-2-[3-(4-methoxyphenyl)thioureido]-β-D-glucopyranosyl azide (**19**).

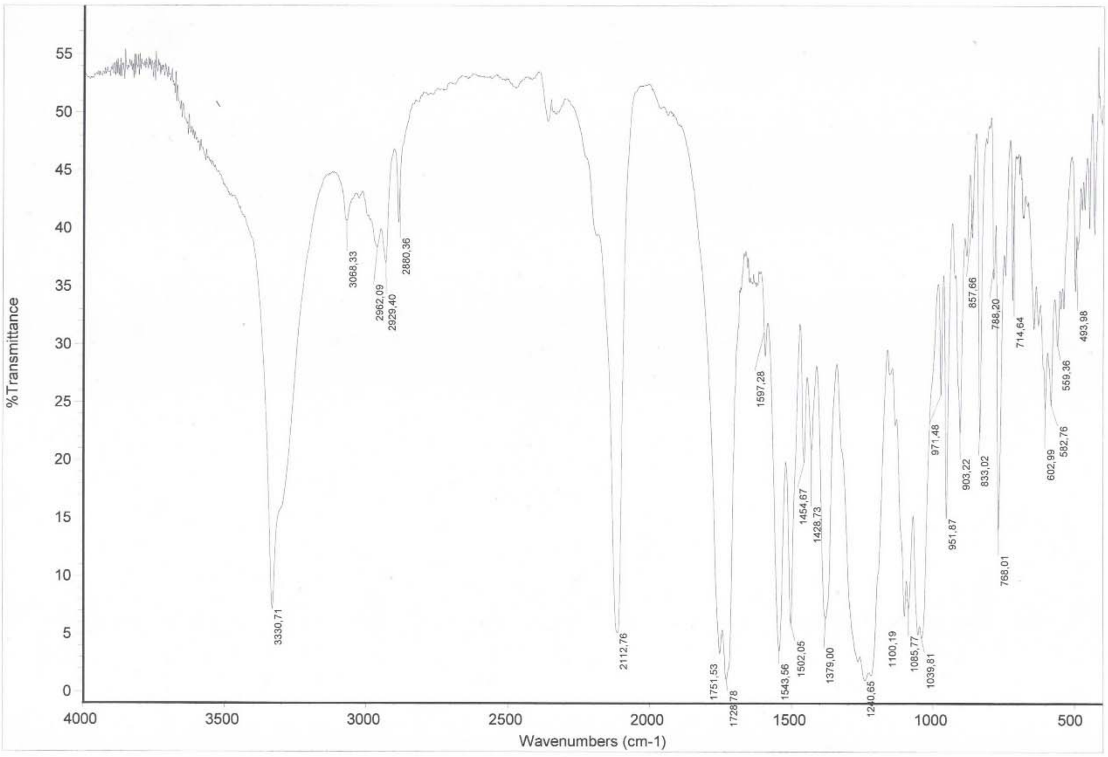

Figure S8 FT-IR spectrum of 3,4,6-tri-*O*-acetyl-2-deoxy-2-[3-(2-fluorophenyl)thioureido]-β-D-glucopyranosyl azide (**20**).

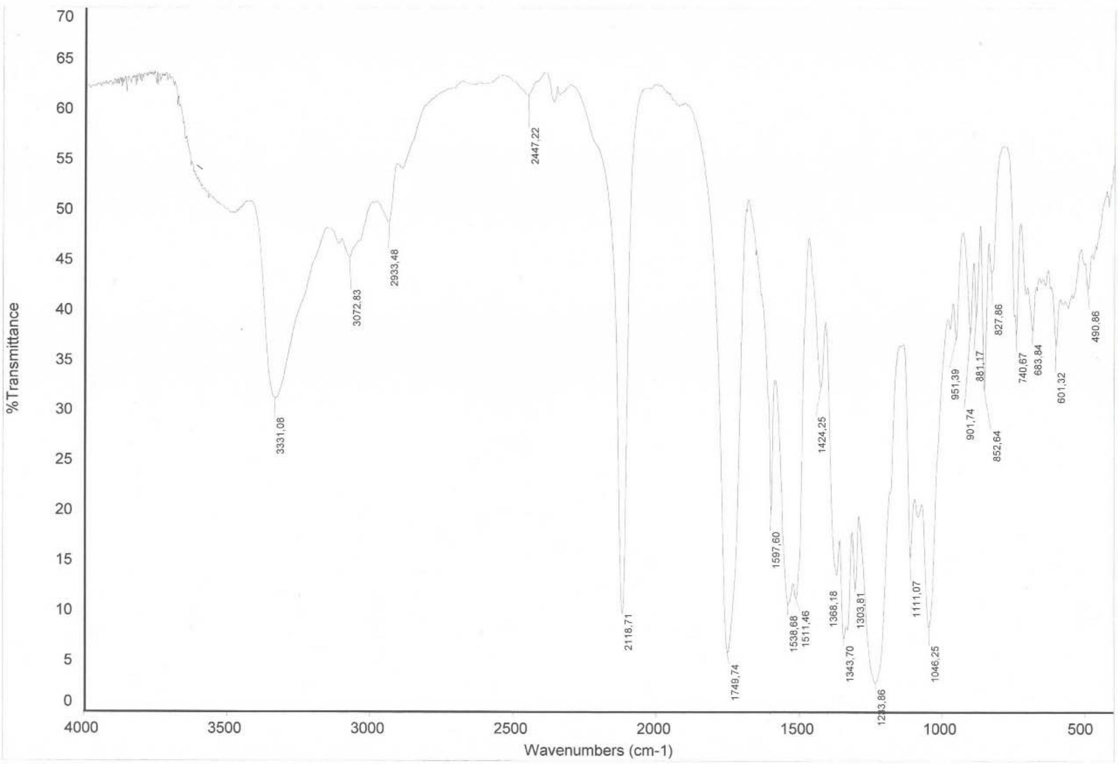

Figure S9 FT-IR spectrum of 3,4,6-tri-*O*-acetyl-2-deoxy-2-[3-(4-nitrophenyl)thioureido]-β-D-glucopyranosyl azide (**21**).

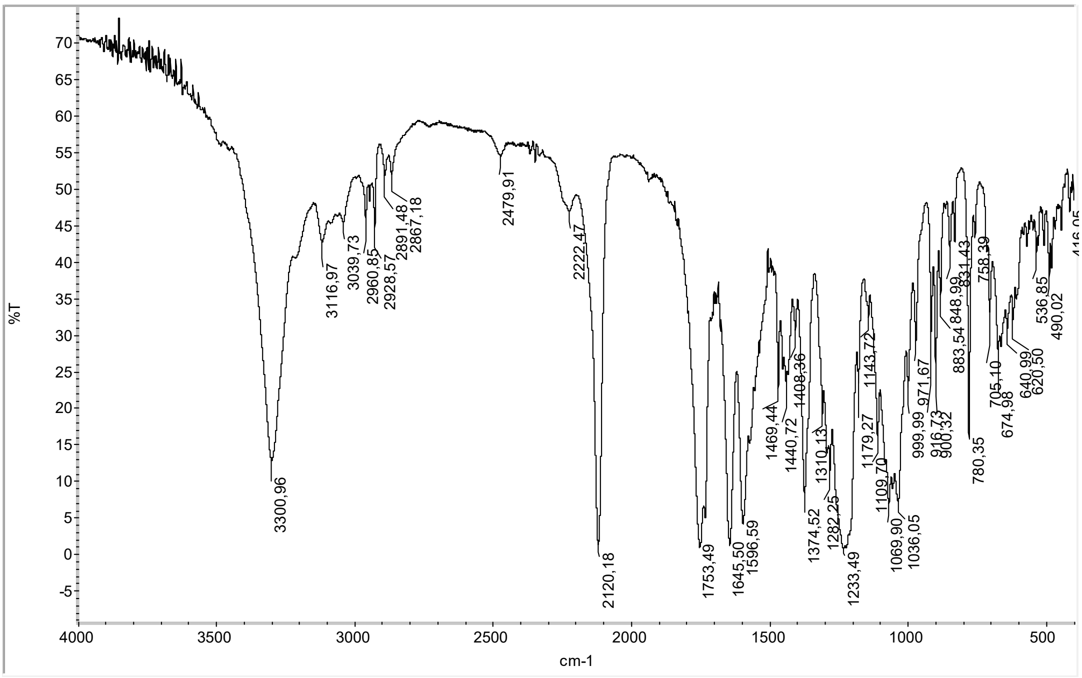

Figure S10 FT-IR spectrum of 3,4,6-tri-*O*-acetyl-2-deoxy-2-[3-(2-chloro-6-methylphenyl)ureido]-β-D-glucopyranosyl azide (**22**).

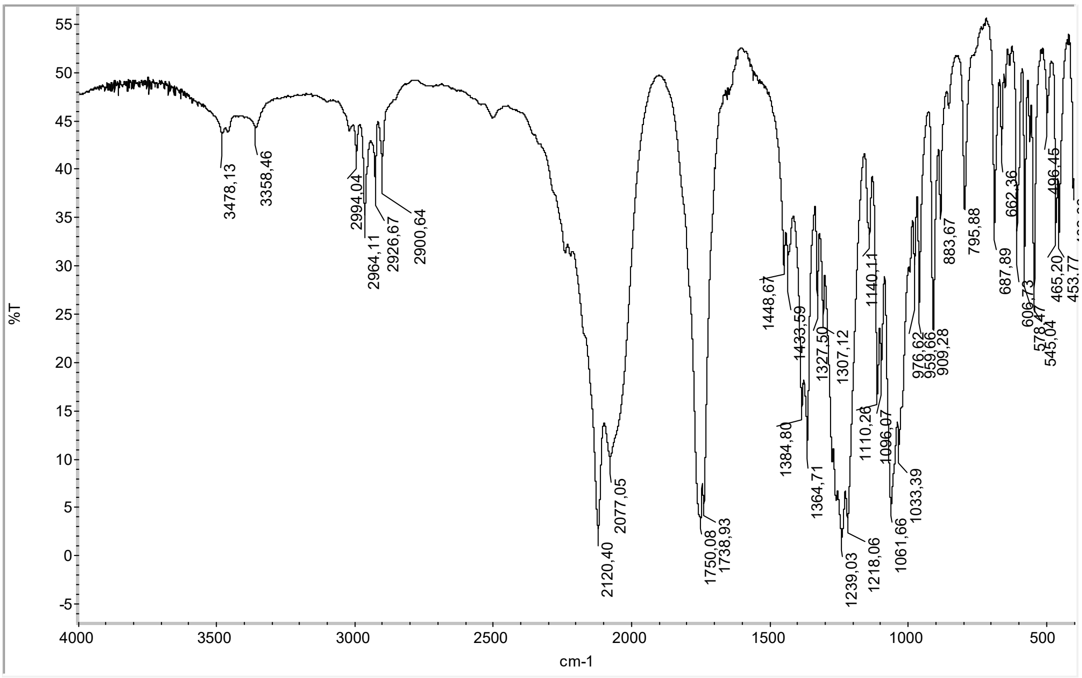

Figure S11 FT-IR spectrum of 3,4,6-tri-*O*-acetyl-2-deoxy-2-isothiocianato-β-D-glucopyranosyl azide (**23**).

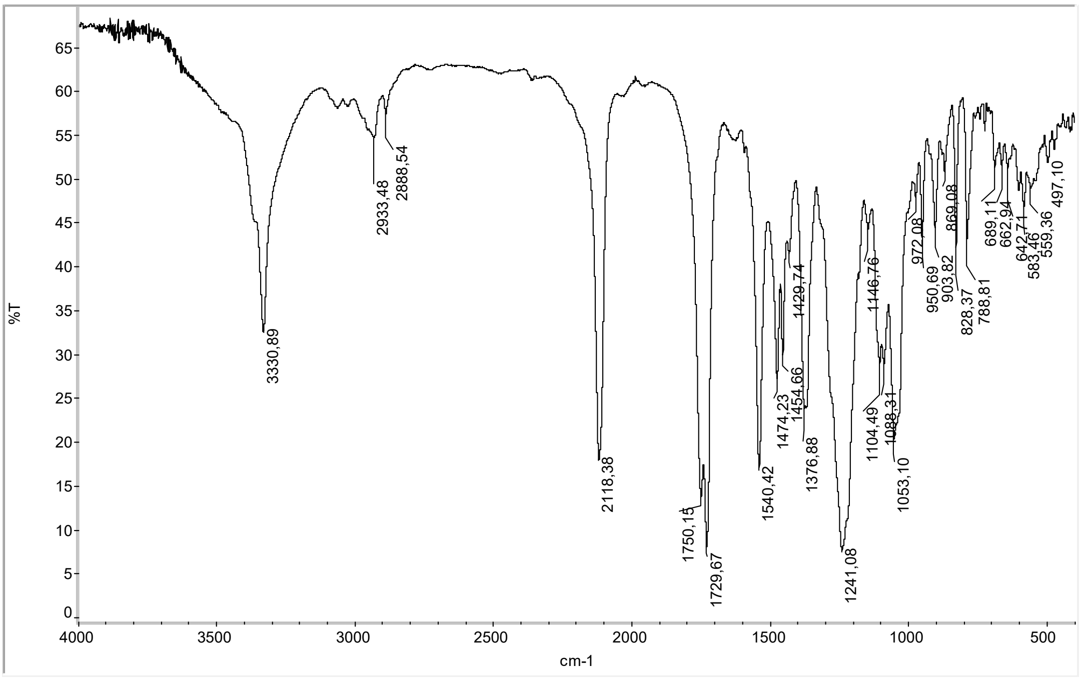

Figure S12 FT-IR spectrum of 3,4,6-tri-*O*-acetyl-2-deoxy-2-[3-(2-chloro-6-methylphenyl)thioureido]-β-D-glucopyranosyl azide (**25**).

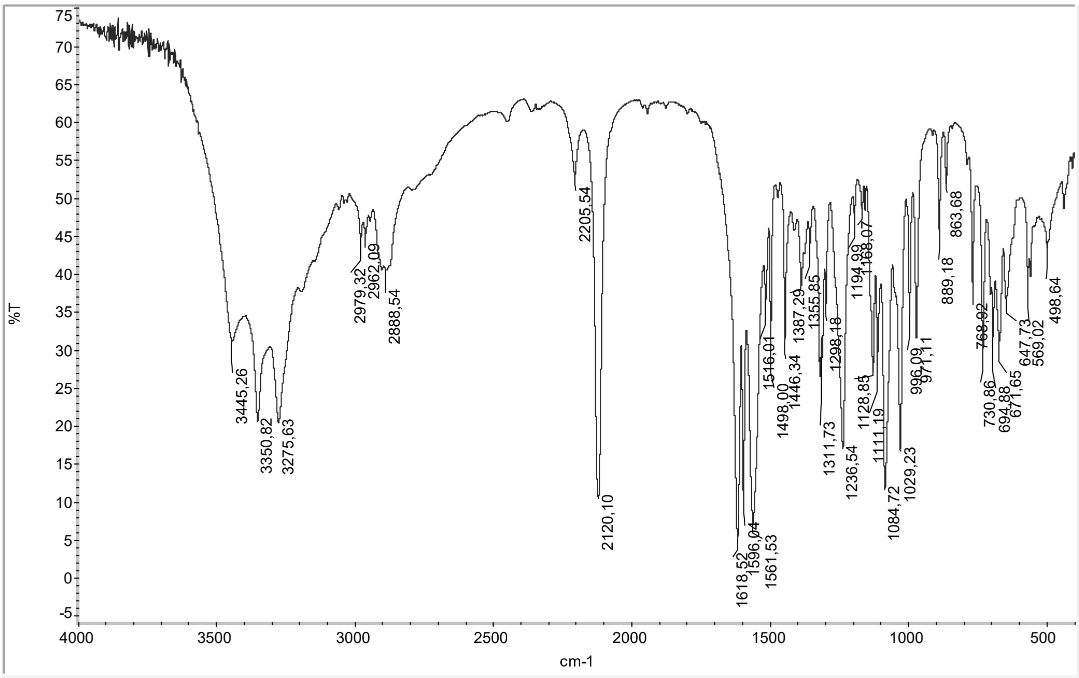

Figure S13 FT-IR spectrum of 2-deoxy-2-(3-phenylureido)-β-D-glucopyranosyl azide (**34**).

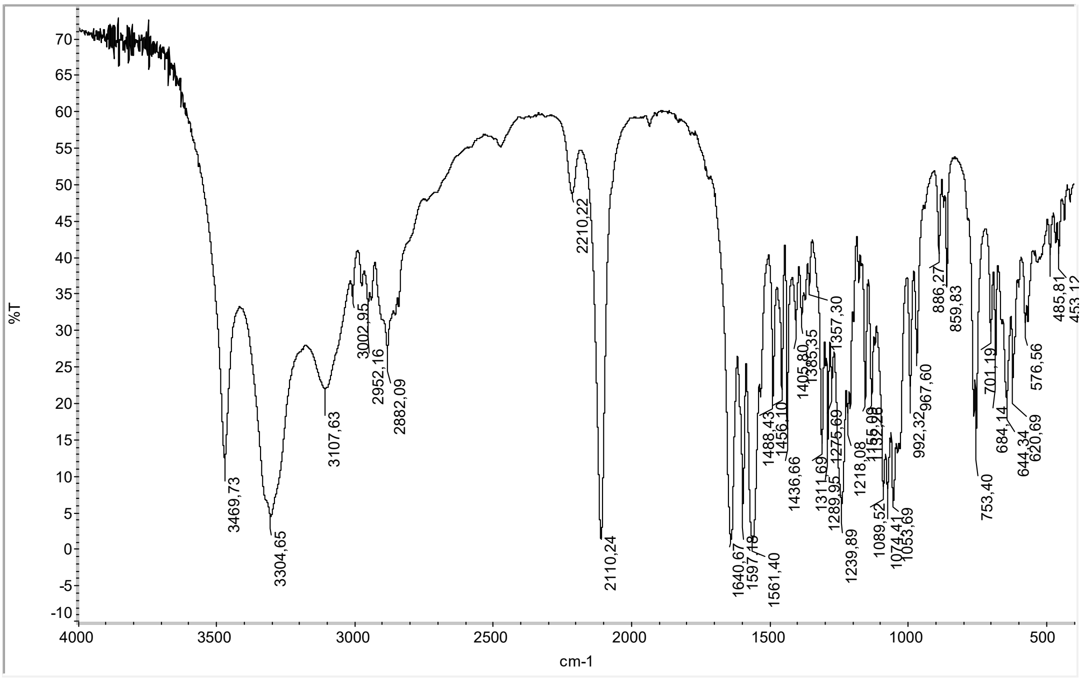

Figure S14 FT-IR spectrum of 2-deoxy-2-[3-(4-methoxyphenyl)ureido]-β-D-glucopyranosyl azide (**35**).

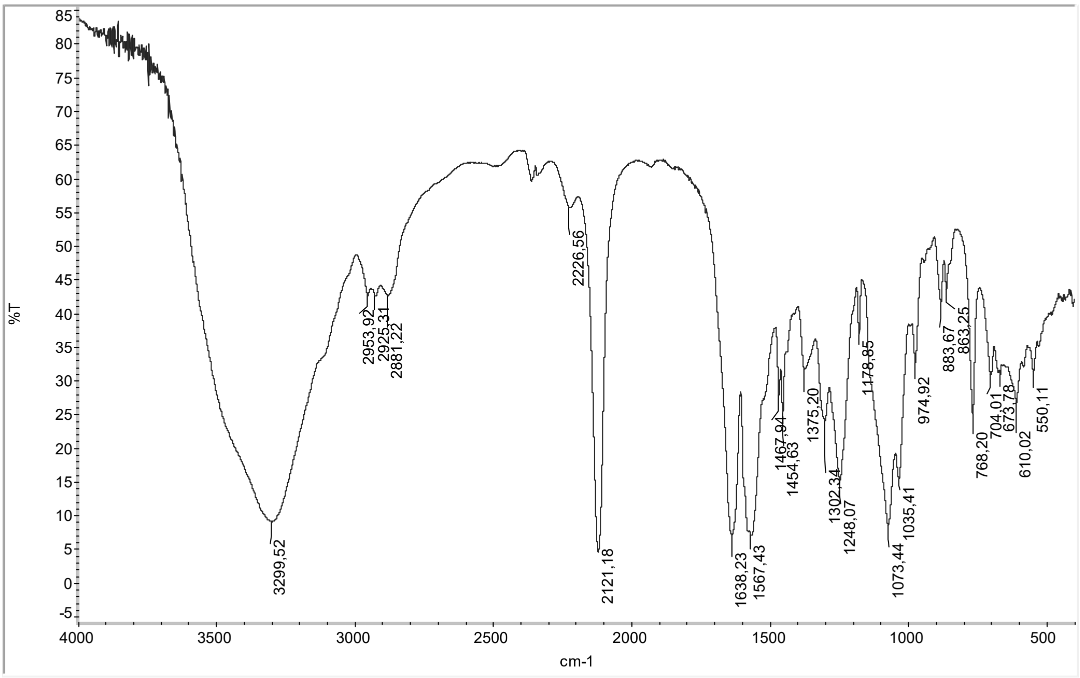

Figure S15 FT-IR spectrum of 2-deoxy-2-[3-(2-chloro-6-methylphenyl)ureido]-β-D-glucopyranosyl azide (**36**).

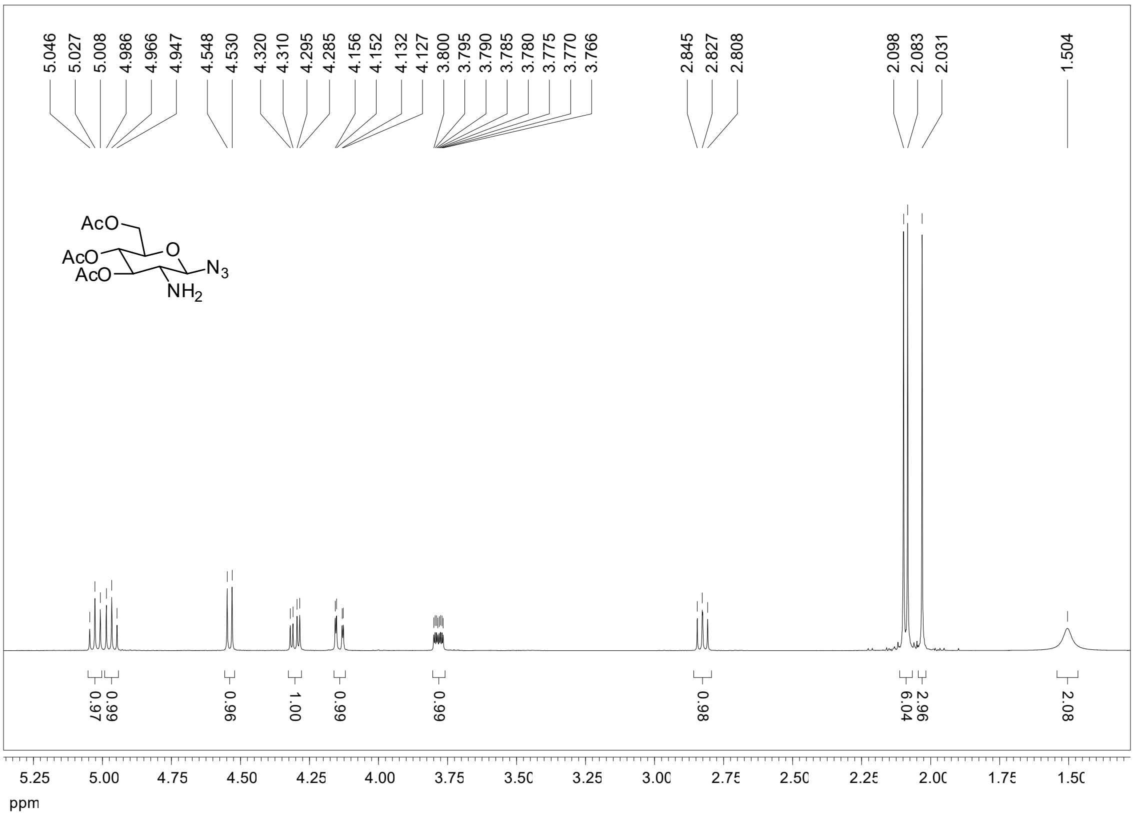

Figure S16 ^{1}H NMR spectrum of 3,4,6-tri-O-acetyl-2-amino-2-deoxy-β-D-glucopyranosyl azide (**1**) recorded in $CDCl_3$.

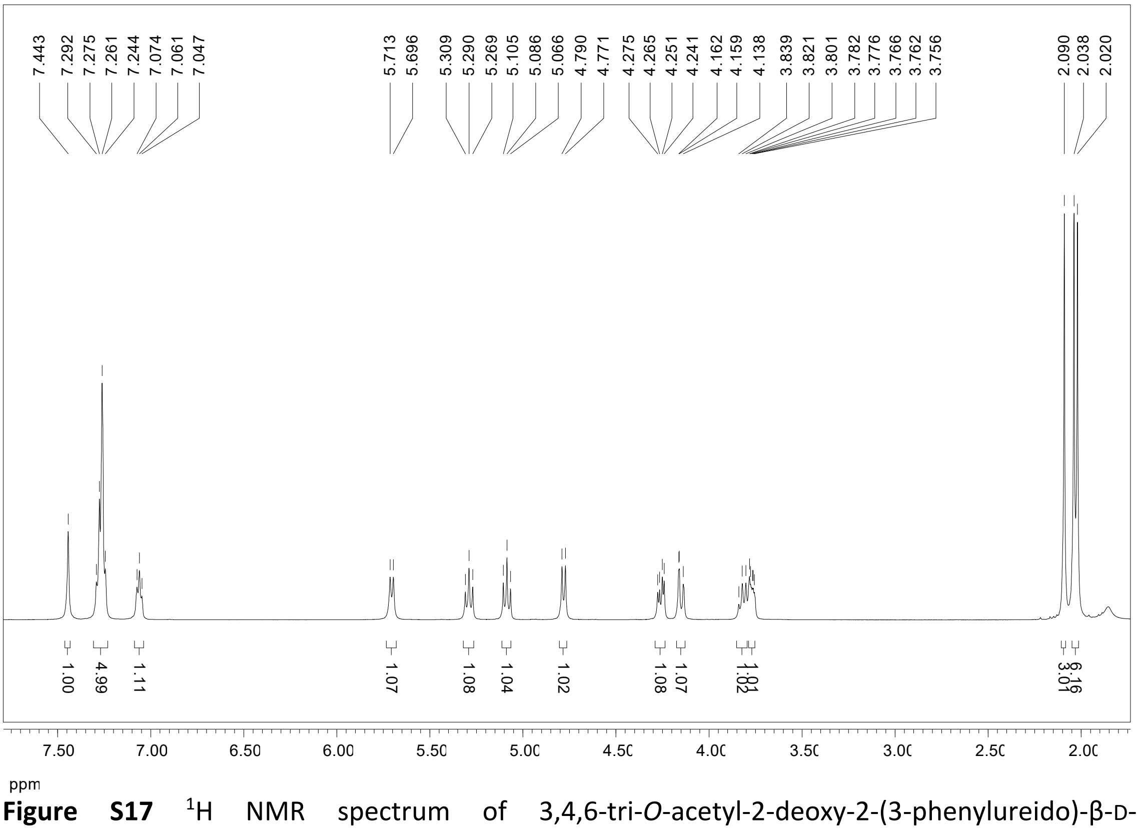

Figure S17 ^{1}H NMR spectrum of 3,4,6-tri-*O*-acetyl-2-deoxy-2-(3-phenylureido)-β-D-glucopyranosyl azide (**14**) recorded in $CDCl_3$.

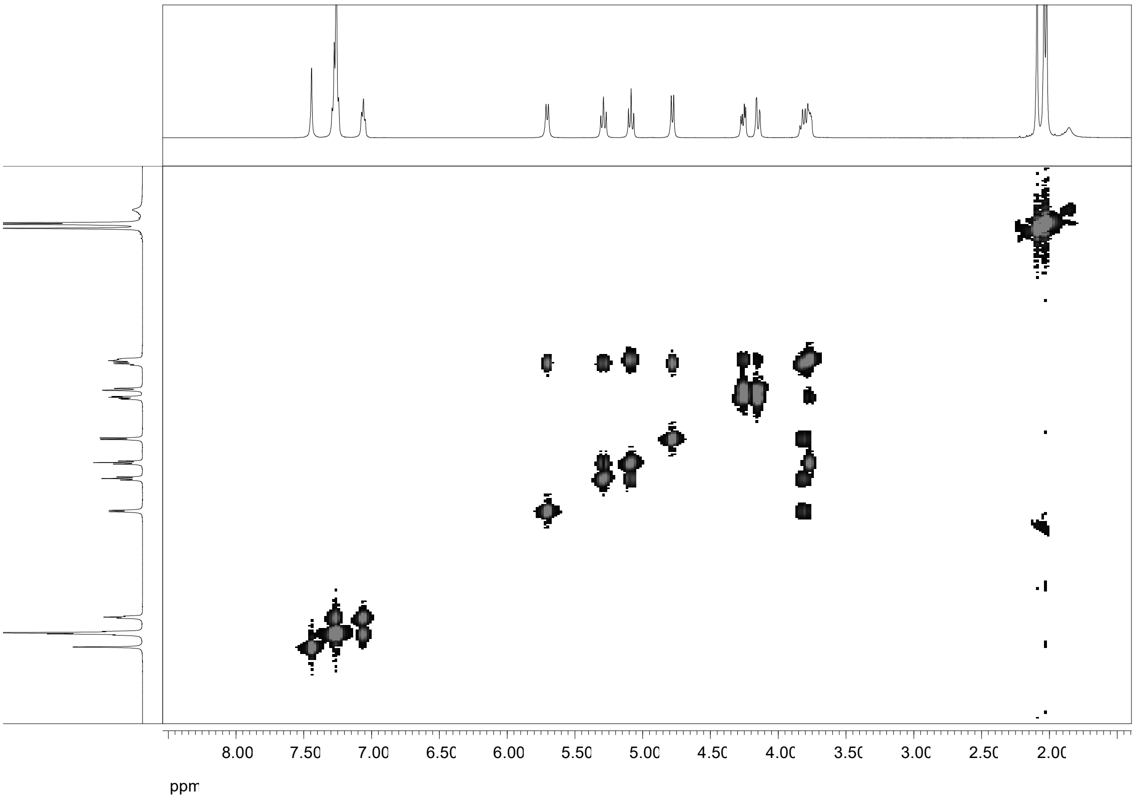

Figure S18 COSY spectrum of 3,4,6-tri-*O*-acetyl-2-deoxy-2-(3-phenylureido)-β-D-glucopyranosyl azide (**14**) recorded in $CDCl_3$.

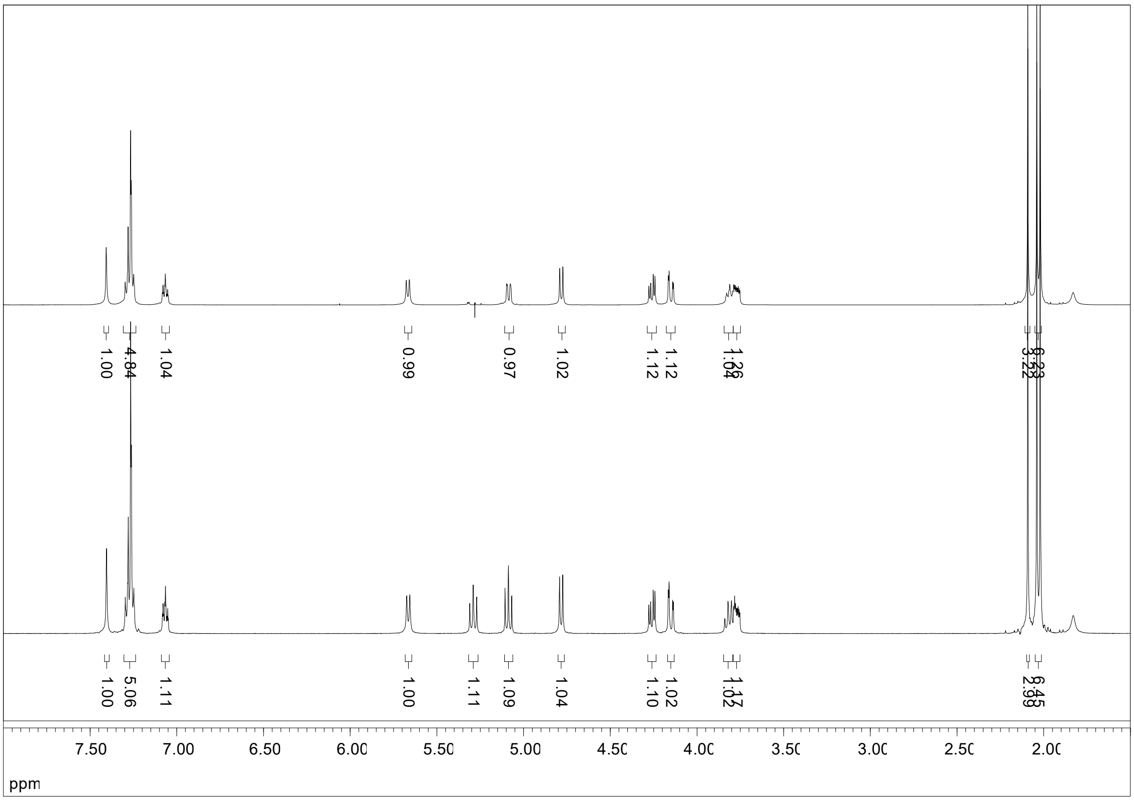

Figure S19 H-3 irradiation of 3,4,6-tri-*O*-acetyl-2-deoxy-2-(3-phenylureido)-β-D-glucopyranosyl azide (**14**) recorded in $CDCl_3$.

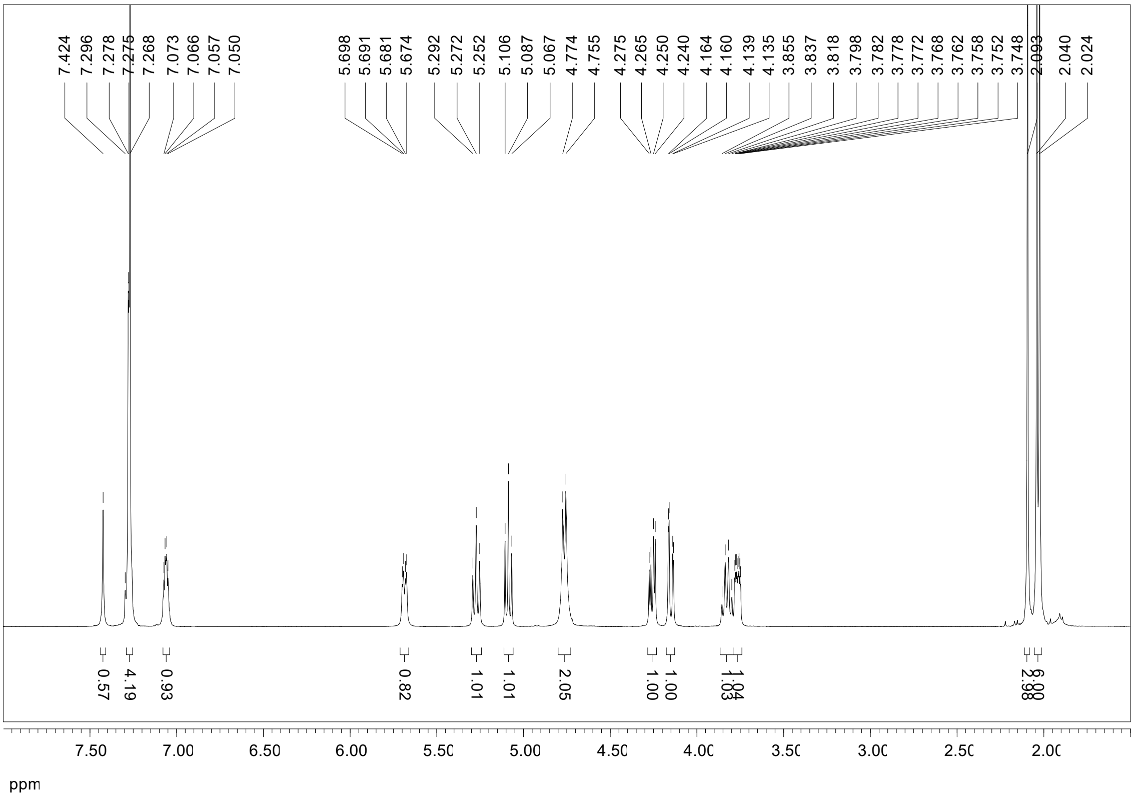

Figure S20 ^{1}H NMR spectrum of 3,4,6-tri-*O*-acetyl-2-deoxy-2-(3-phenylureido)-β-D-glucopyranosyl azide (**14**) recorded with isotopic exchange in D_2O.

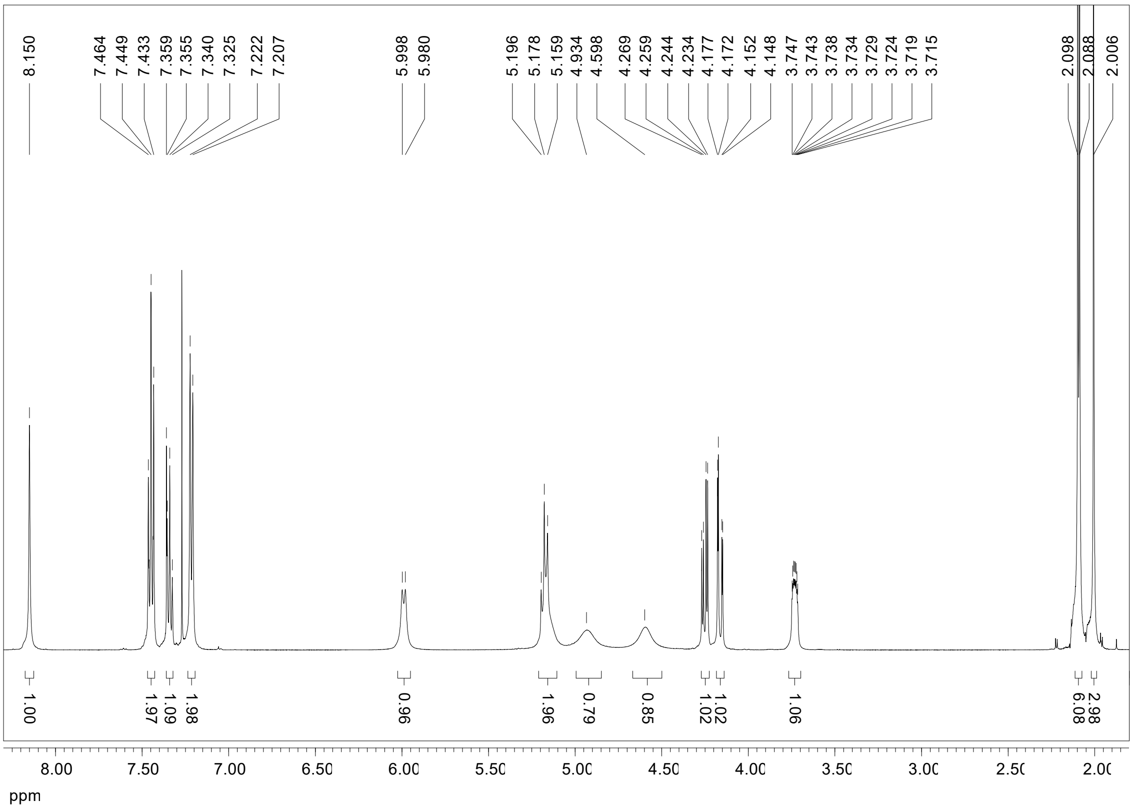

Figure S21 ^{1}H NMR spectrum of 3,4,6-tri-*O*-acetyl-2-deoxy-2-(3-phenylthioureido)-β-D-glucopyranosyl azide (**15**) recorded in $CDCl_3$.

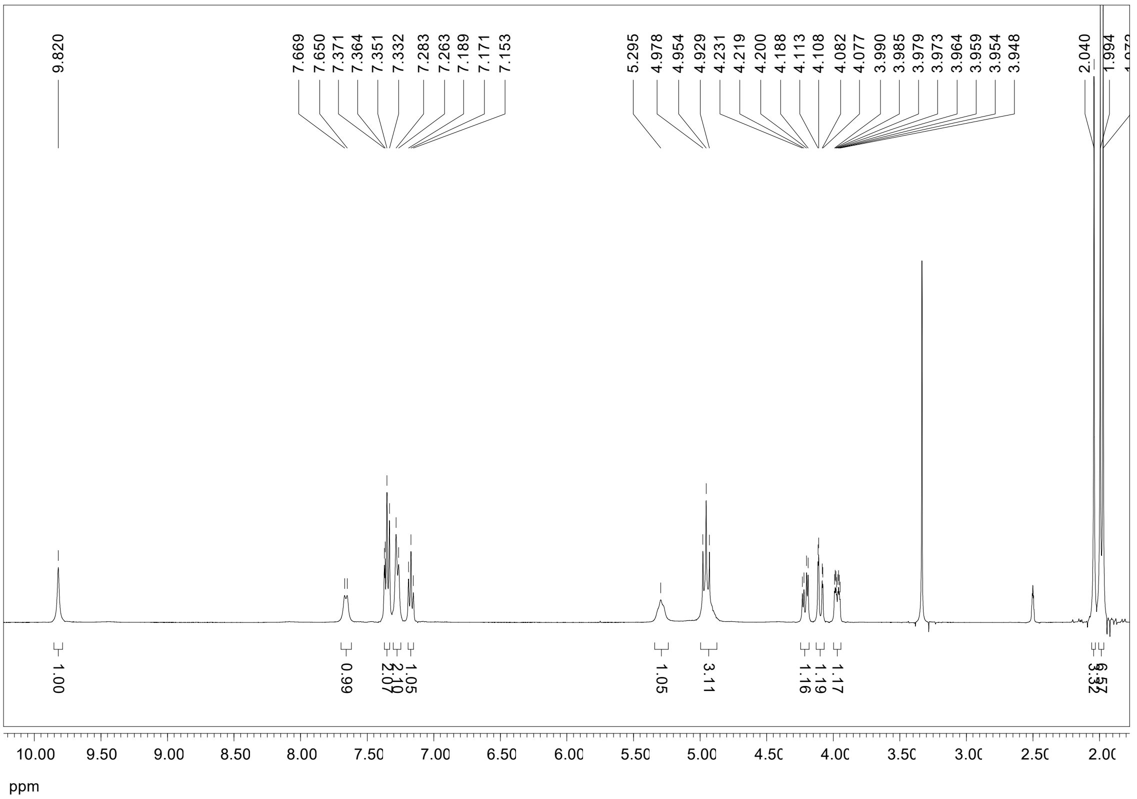

Figure S22 ^{1}H NMR spectrum of 3,4,6-tri-*O*-acetyl-2-deoxy-2-(3-phenylthioureido)-β-D-glucopyranosyl azide (**15**) recorded in DMSO-d_6.

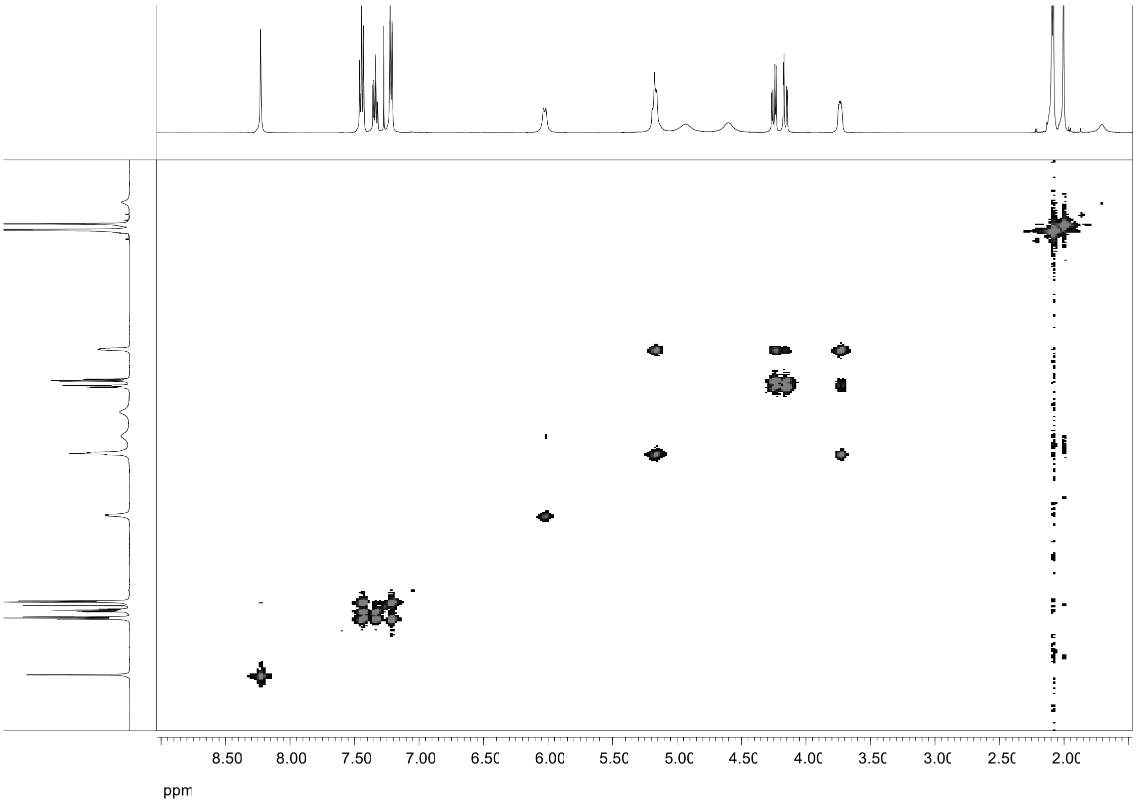

Figure S23 COSY spectrum of 3,4,6-tri-*O*-acetyl-2-deoxy-2-(3-phenylthioureido)-β-D-glucopyranosyl azide (**15**) recorded in CDCl$_3$.

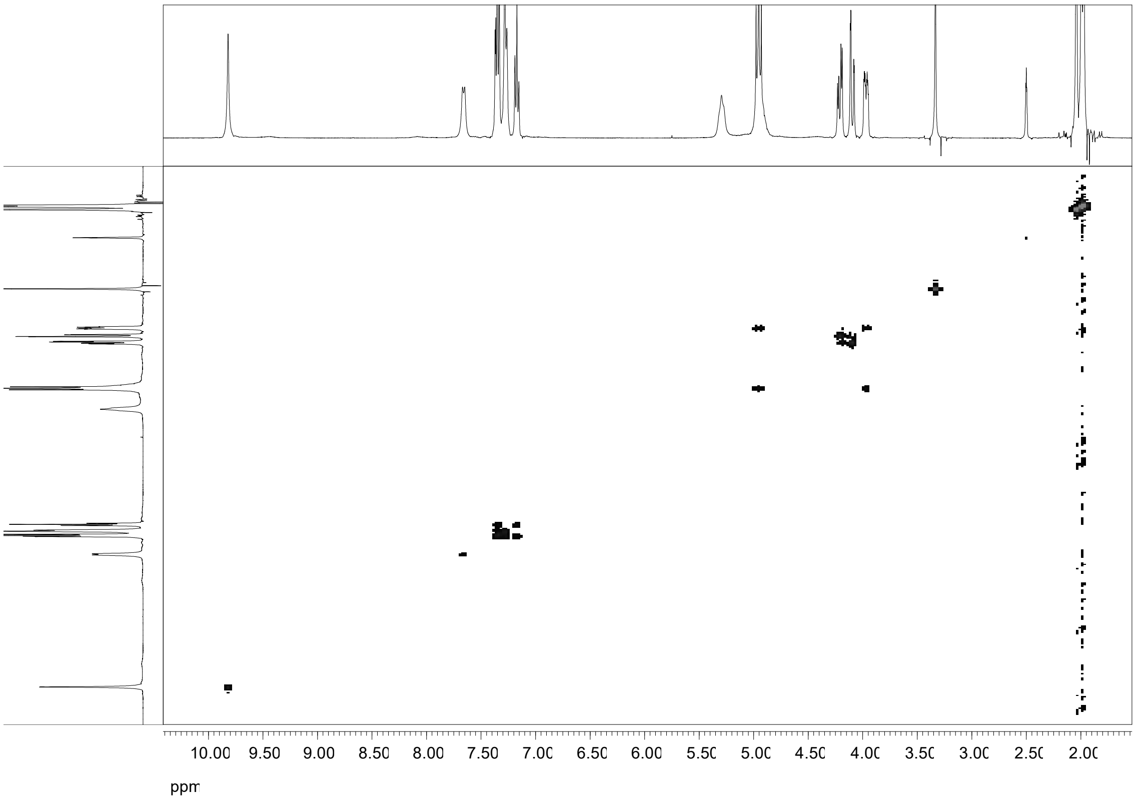

Figure S24 COSY spectrum of 3,4,6-tri-*O*-acetyl-2-deoxy-2-(3-phenylthioureido)-β-D-glucopyranosyl azide (**15**) recorded in DMSO-d_6.

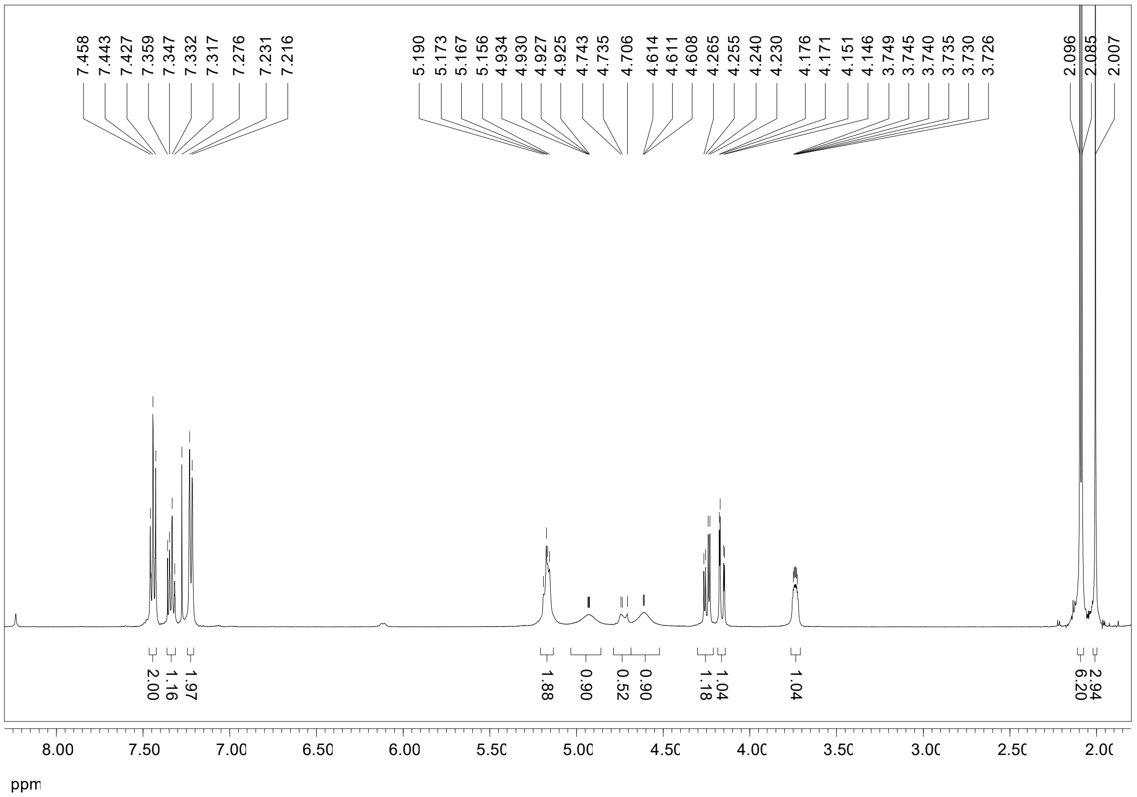

Figure S25 ^{1}H NMR spectrum of 3,4,6-tri-*O*-acetyl-2-deoxy-2-(3-phenylthioureido)-β-D-glucopyranosyl azide (**15**) recorded with isotopic exchange in D_2O.

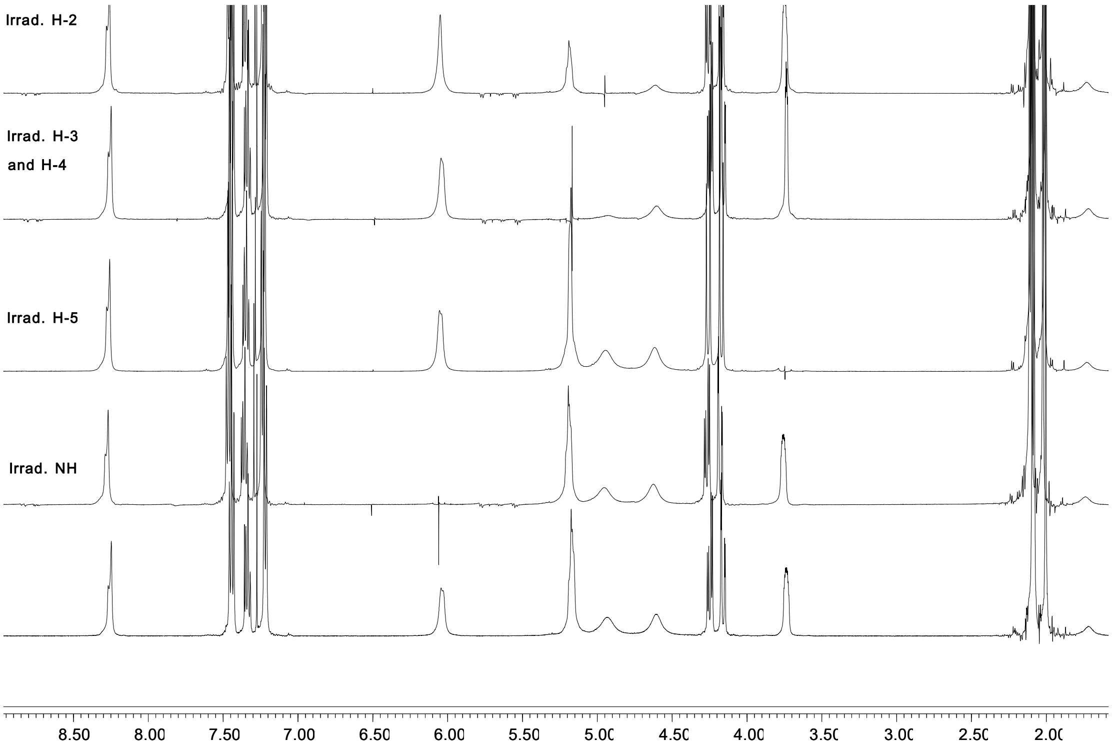

Figure S26 Selective proton irradiations of 3,4,6-tri-*O*-acetyl-2-deoxy-2-(3-phenylthioureido)-β-D-glucopyranosyl azide (**15**) recorded in $CDCl_3$.

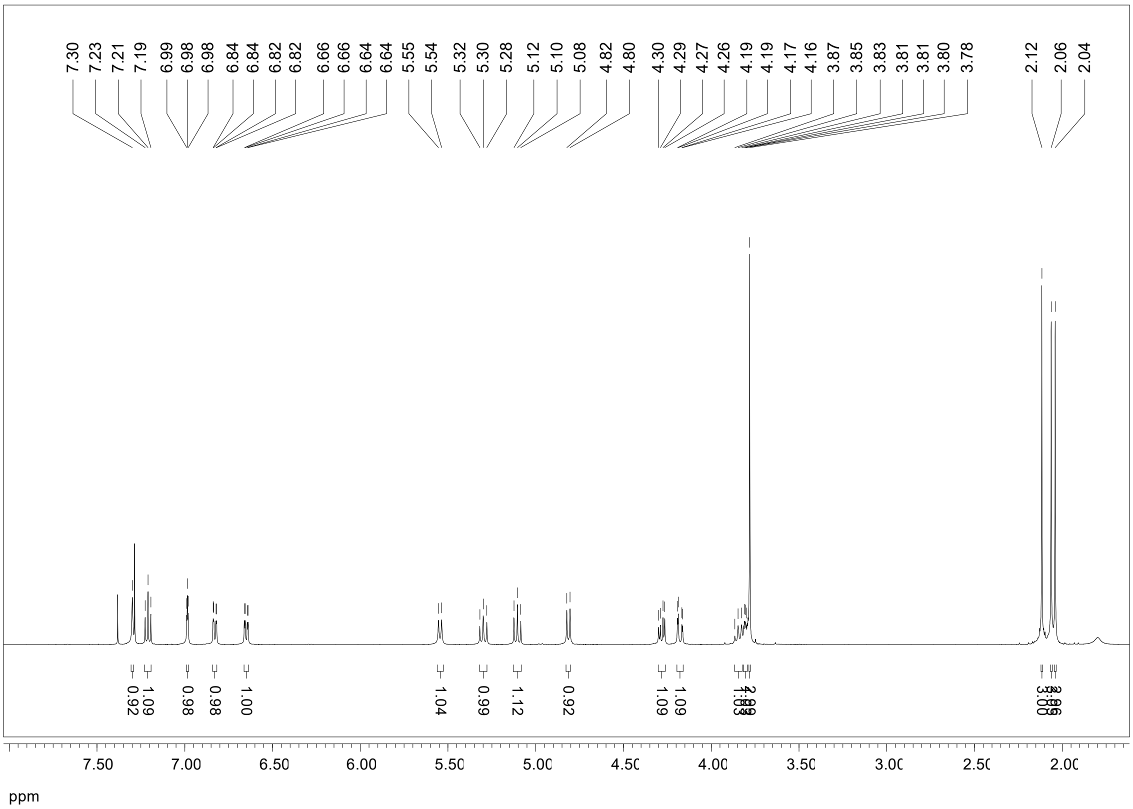

Figure S27 ^{1}H NMR spectrum of 3,4,6-tri-*O*-acetyl-2-deoxy-2-[3-(3-methoxyphenyl)ureido]-β-D-glucopyranosyl azide (**16**) recorded in $CDCl_3$.

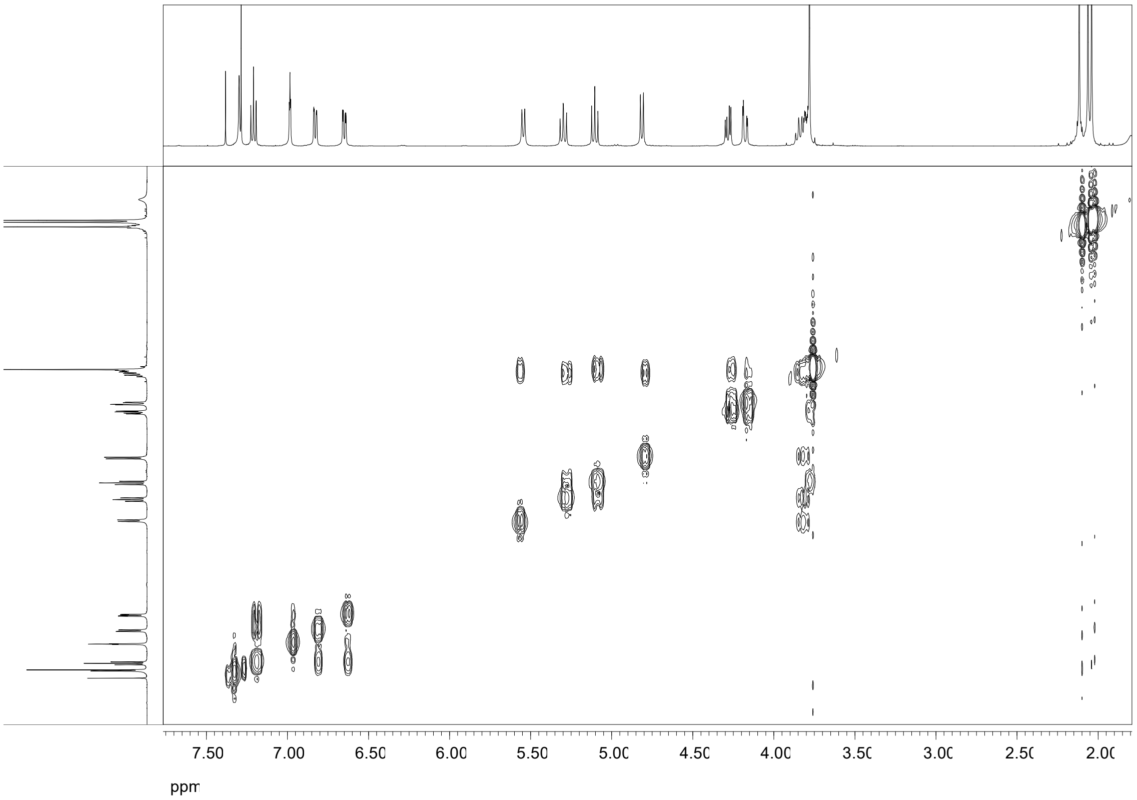

Figure S28 COSY spectrum of 3,4,6-tri-*O*-acetyl-2-deoxy-2-[3-(3-methoxyphenyl)ureido]-β-D-glucopyranosyl azide (**16**) recorded in $CDCl_3$.

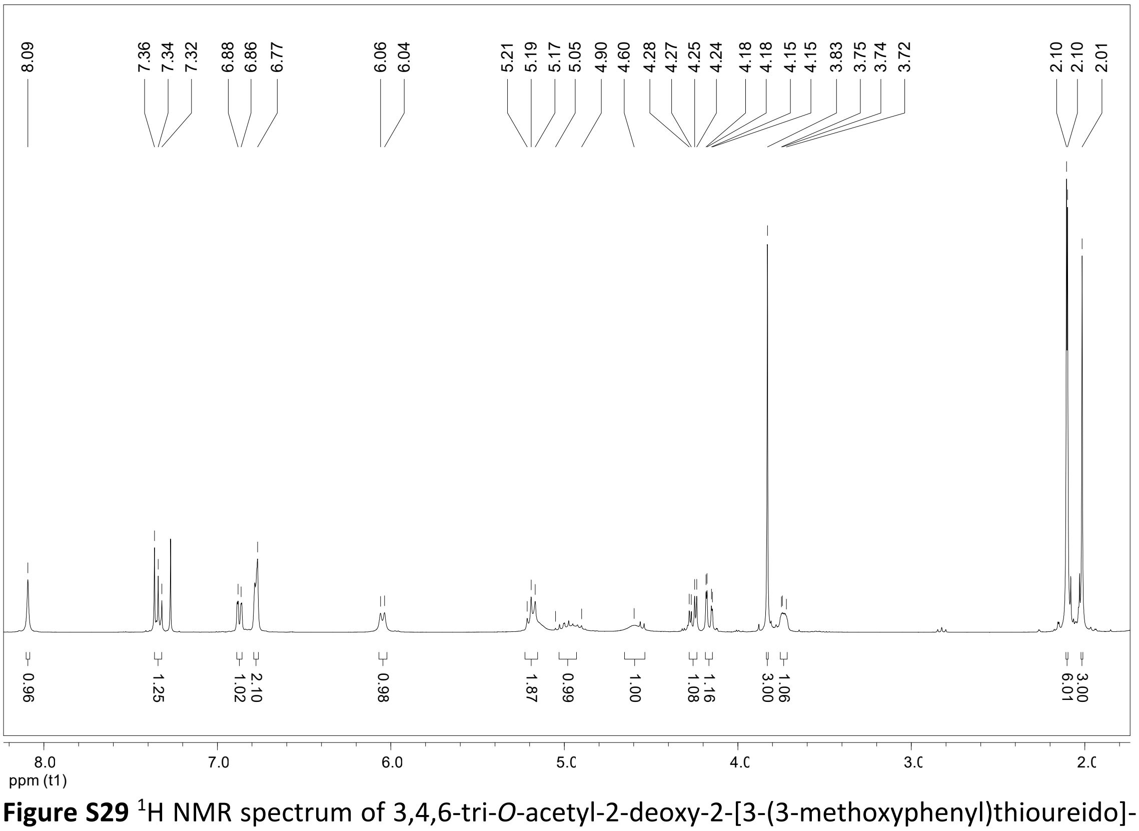

Figure S29 ^{1}H NMR spectrum of 3,4,6-tri-*O*-acetyl-2-deoxy-2-[3-(3-methoxyphenyl)thioureido]-β-D-glucopyranosyl azide (**17**) recorded in $CDCl_3$.

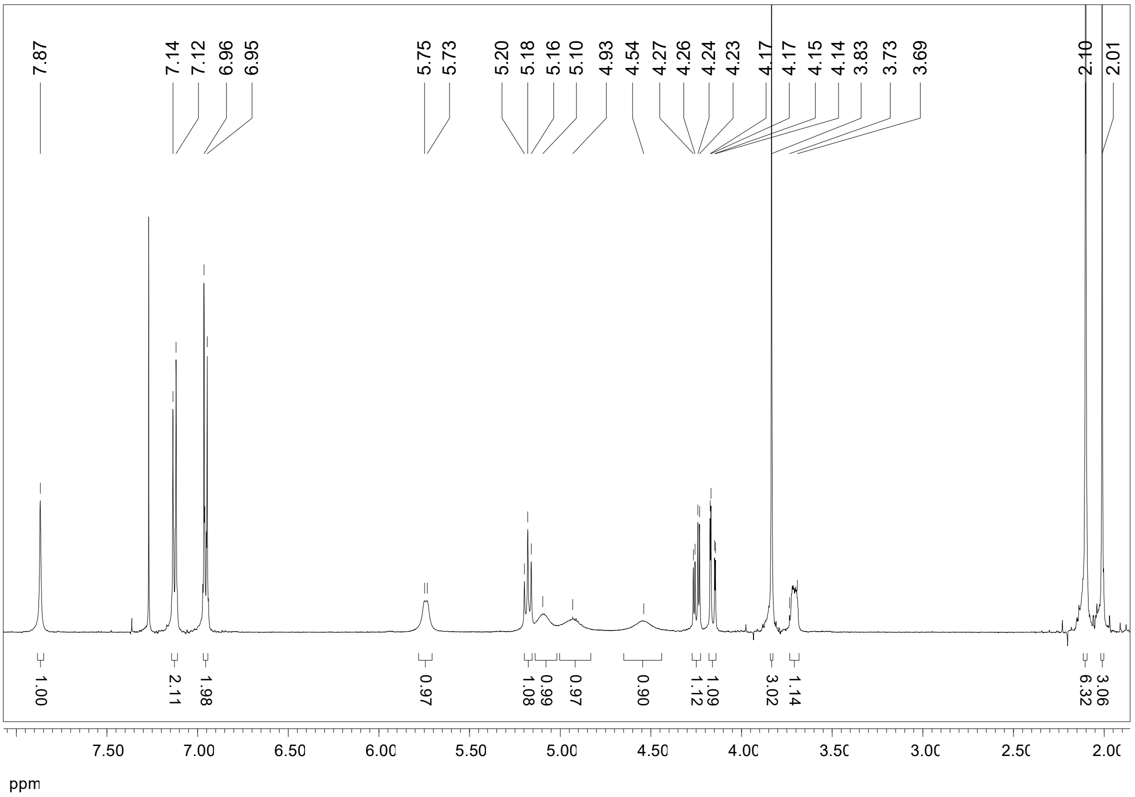

Figure S30 ¹H NMR spectrum of 3,4,6-tri-*O*-acetyl-2-deoxy-2-[3-(4-methoxyphenyl)ureido]-β-D-glucopyranosyl azide (**18**) recorded in $CDCl_3$.

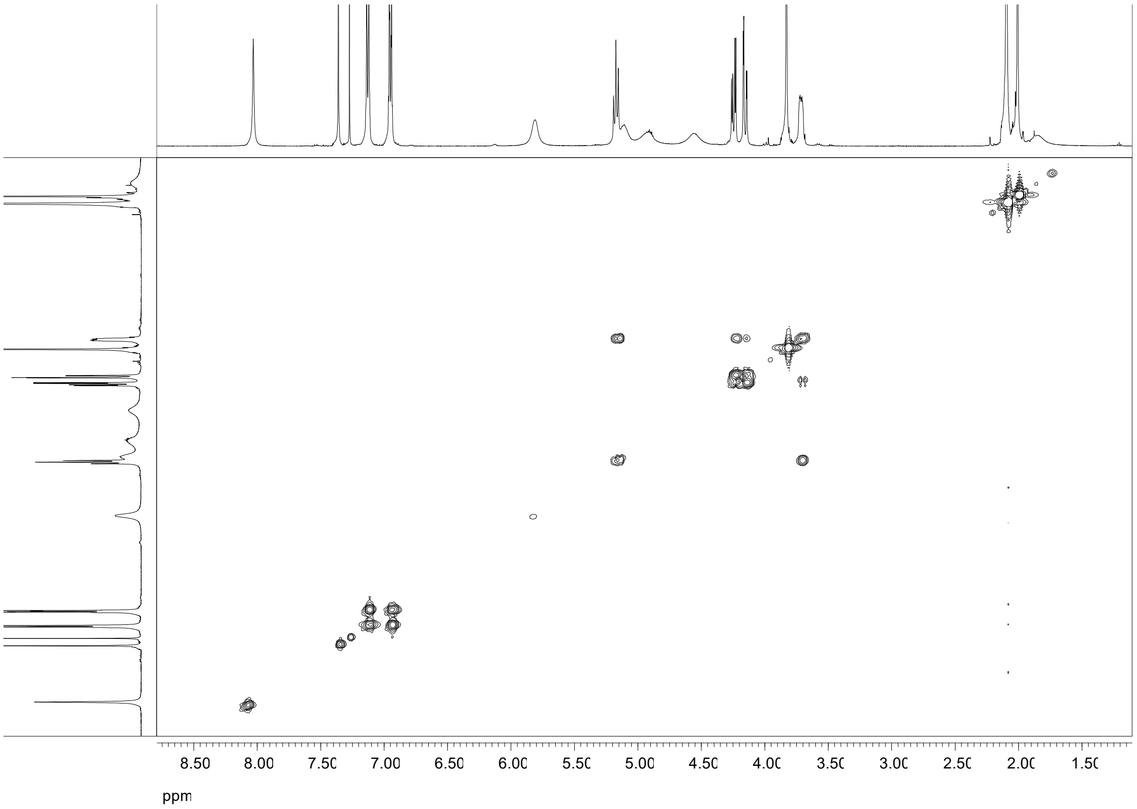

Figure S31 COSY spectrum of 3,4,6-tri-*O*-acetyl-2-deoxy-2-[3-(4-methoxyphenyl)ureido]-β-D-glucopyranosyl azide (**18**) recorded in $CDCl_3$.

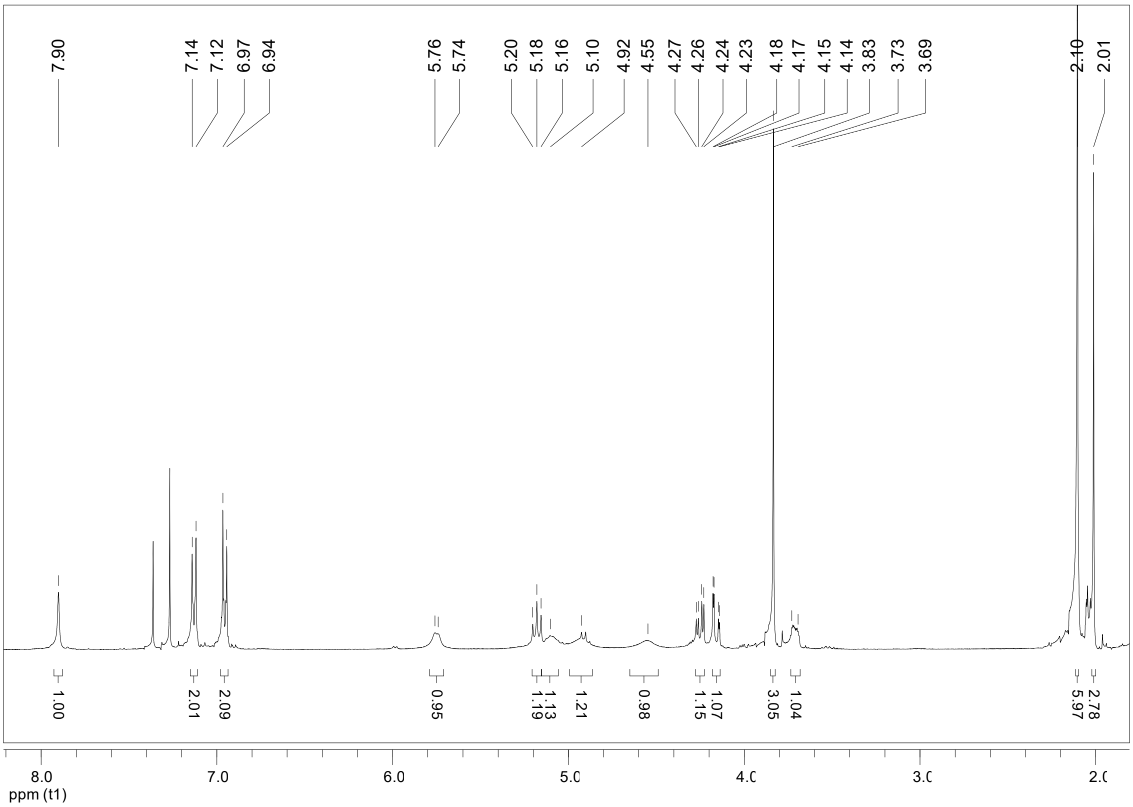

Figure S32 ^{1}H NMR spectrum of 3,4,6-tri-*O*-acetyl-2-deoxy-2-[3-(4-methoxyphenyl)thioureido]-β-D-glucopyranosyl azide (**19**) recorded in $CDCl_3$.

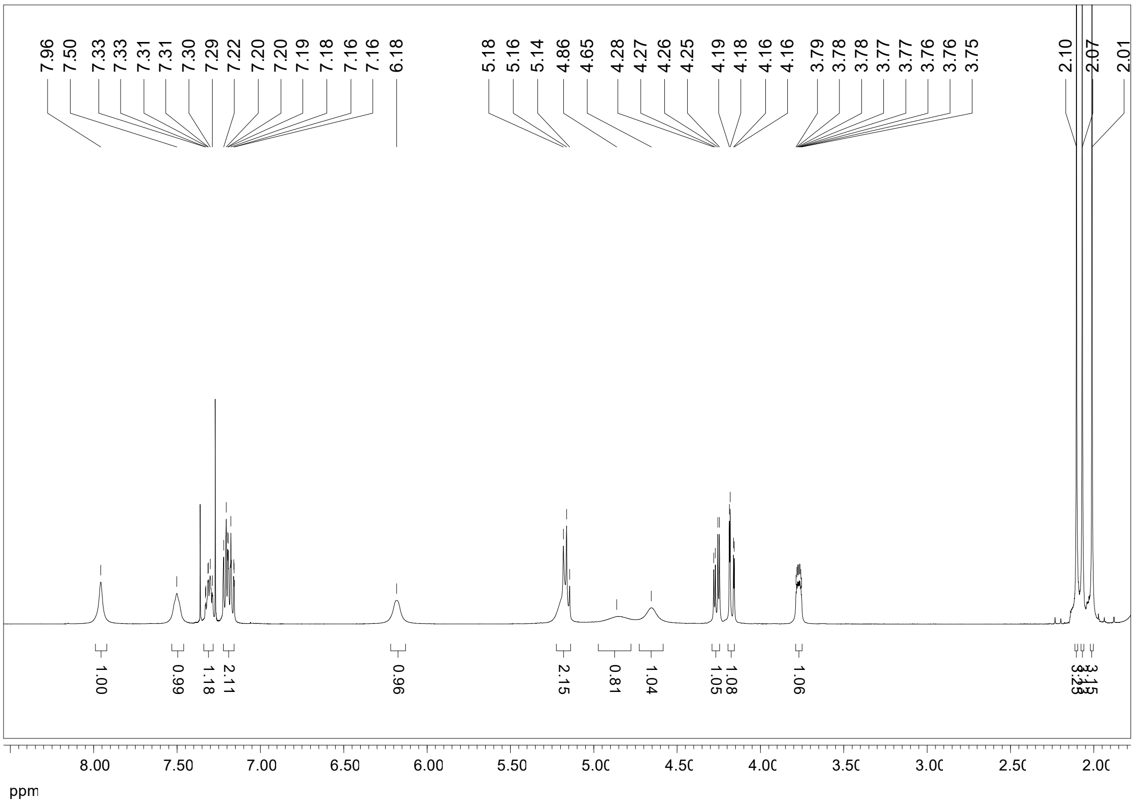

Figure S33 ^{1}H NMR spectrum of 3,4,6-tri-*O*-acetyl-2-deoxy-2-[3-(2-fluorophenyl)thioureido]-β-D-glucopyranosyl azide (**20**) recorded in $CDCl_3$.

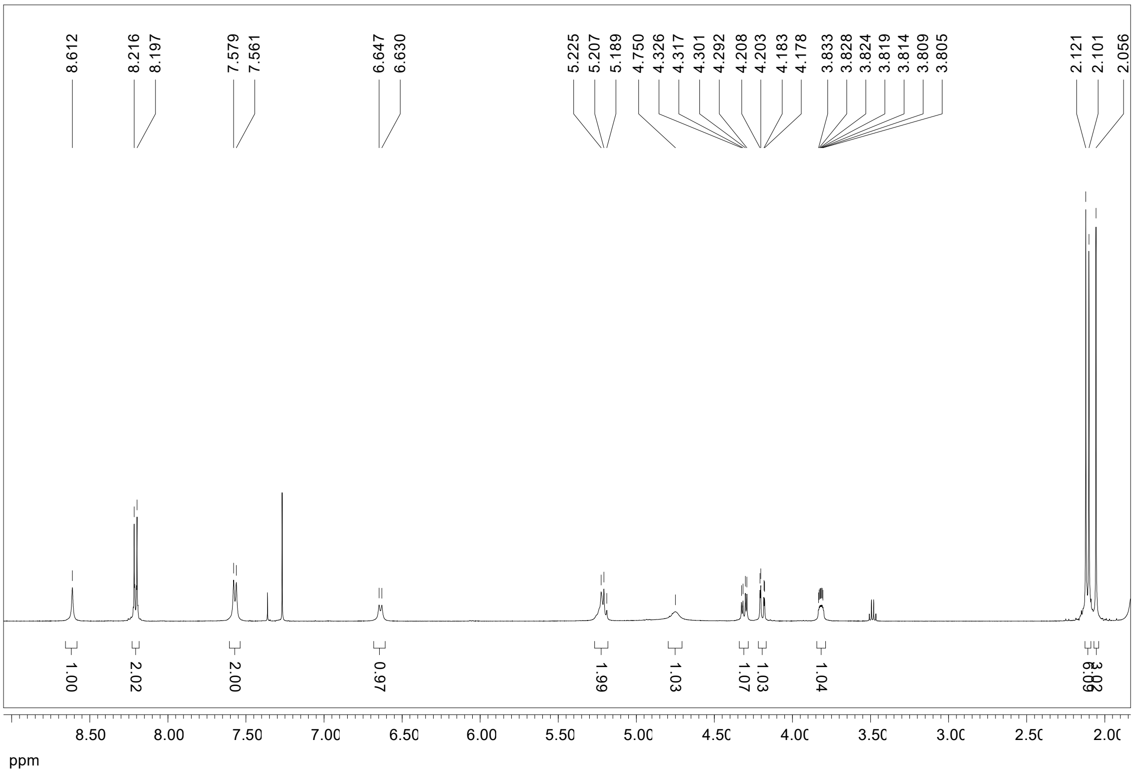

Figure S34 ^{1}H NMR spectrum of 3,4,6-tri-*O*-acetyl-2-deoxy-2-[3-(4-nitrophenyl)thioureido]-β-D-glucopyranosyl azide (**21**) recorded in $CDCl_3$.

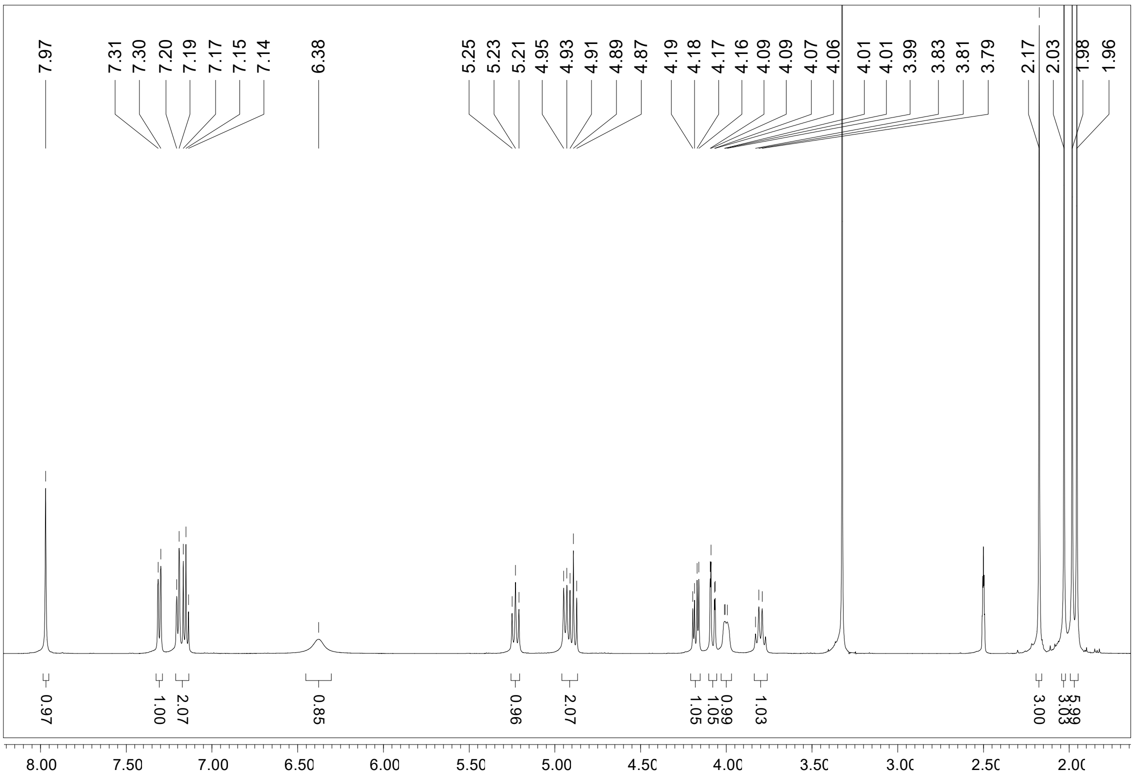

Figure S35 ^{1}H NMR spectrum of 3,4,6-tri-*O*-acetyl-2-deoxy-2-[3-(2-chloro-6-methylphenyl)ureido]-β-D-glucopyranosyl azide (**22**) recorded in DMSO-d_6.

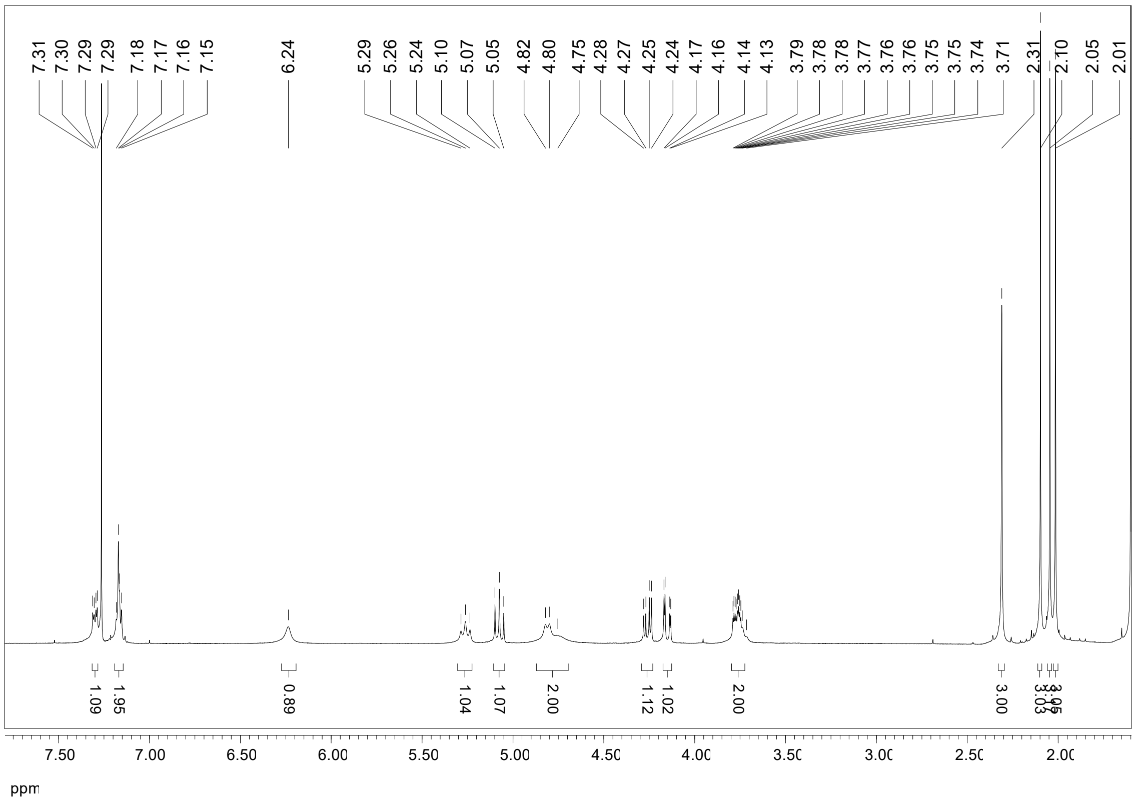

Figure S36 ^{1}H NMR spectrum of 3,4,6-tri-*O*-acetyl-2-deoxy-2-[3-(2-chloro-6-methylphenyl)ureido]-β-D-glucopyranosyl azide (**22**) recorded in $CDCl_3$.

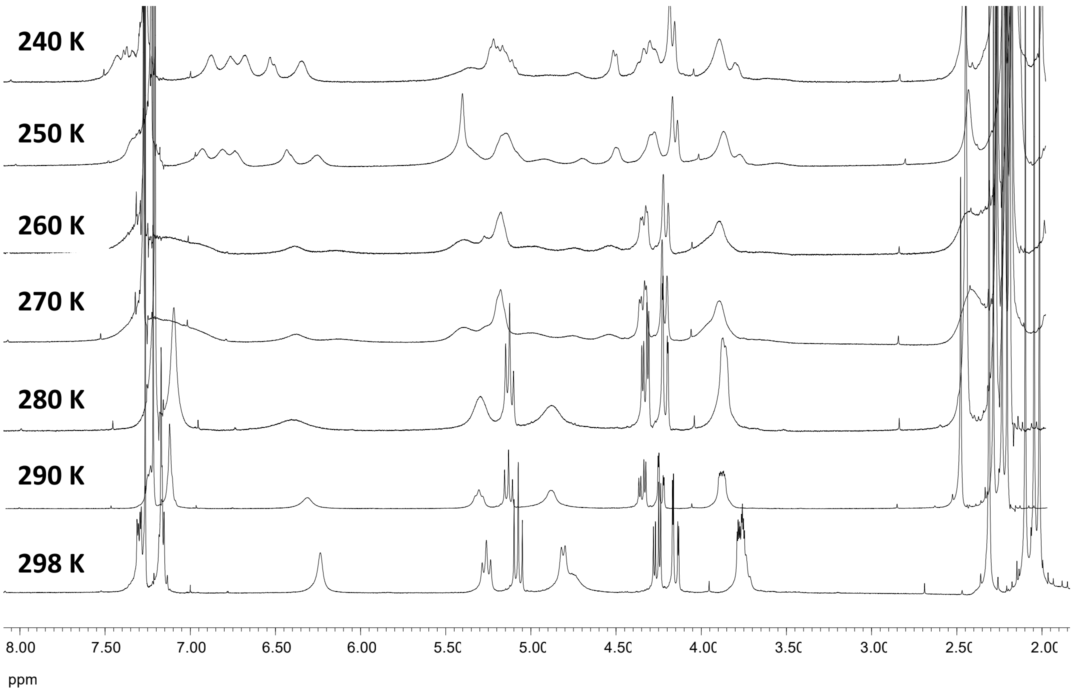

Figure S37 Variable-temperature ^{1}H NMR spectra of 3,4,6-tri-*O*-acetyl-2-deoxy-2-[3-(2-chloro-6-methylphenyl)ureido]-β-D-glucopyranosyl azide (**22**) recorded in $CDCl_3$.

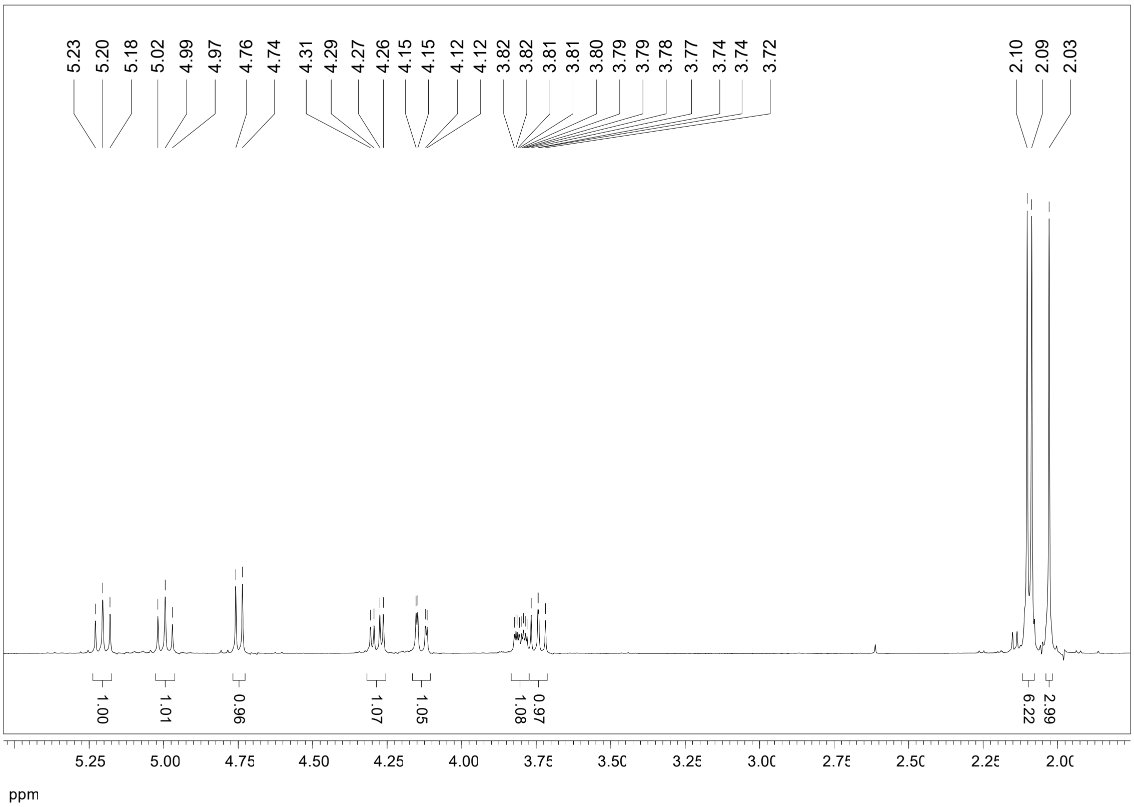

Figure S38 ^{1}H NMR spectrum of 3,4,6-tri-*O*-acetyl-2-deoxy-2-isothiocianato-β-D-glucopyranosyl azide (**23**) recorded in $CDCl_3$..

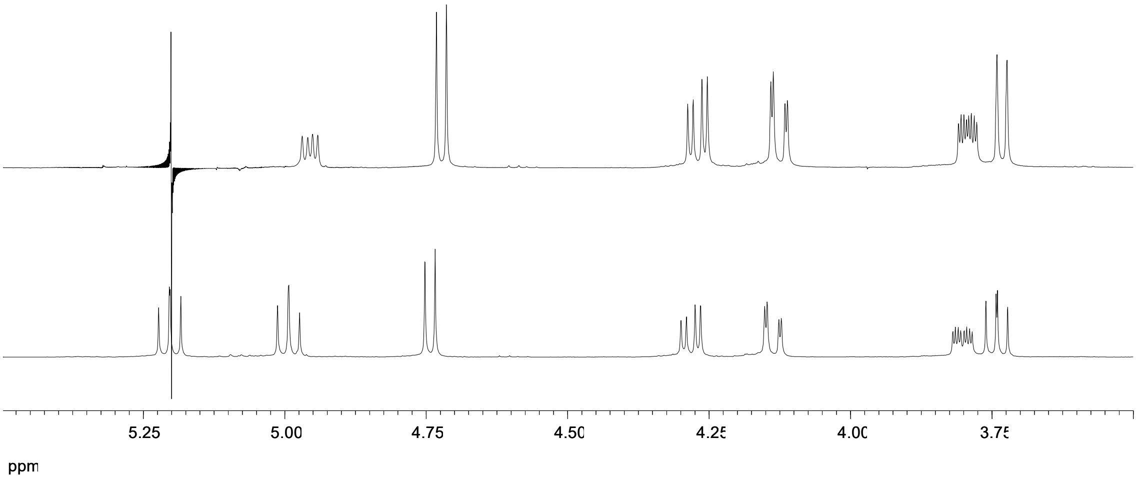

Figure S39 H-3 irradiation of 3,4,6-tri-*O*-acetyl-2-deoxy-2-isothiocianato-β-D-glucopyranosyl azide (**23**) recorded in $CDCl_3$..

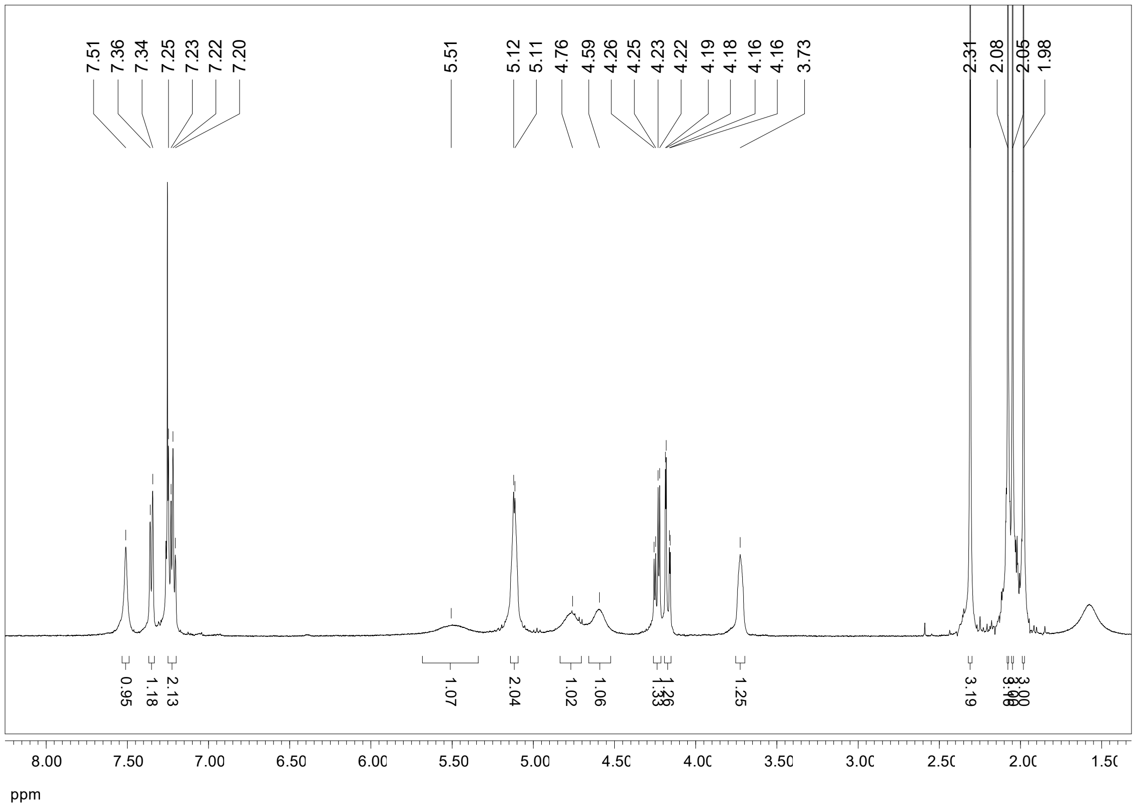

Figure S40 ^{1}H NMR spectrum of 3,4,6-tri-*O*-acetyl-2-deoxy-2-[3-(2-chloro-6-methylphenyl)thioureido]-β-D-glucopyranosyl azide (**25**) recorded in $CDCl_3$ at 330K.

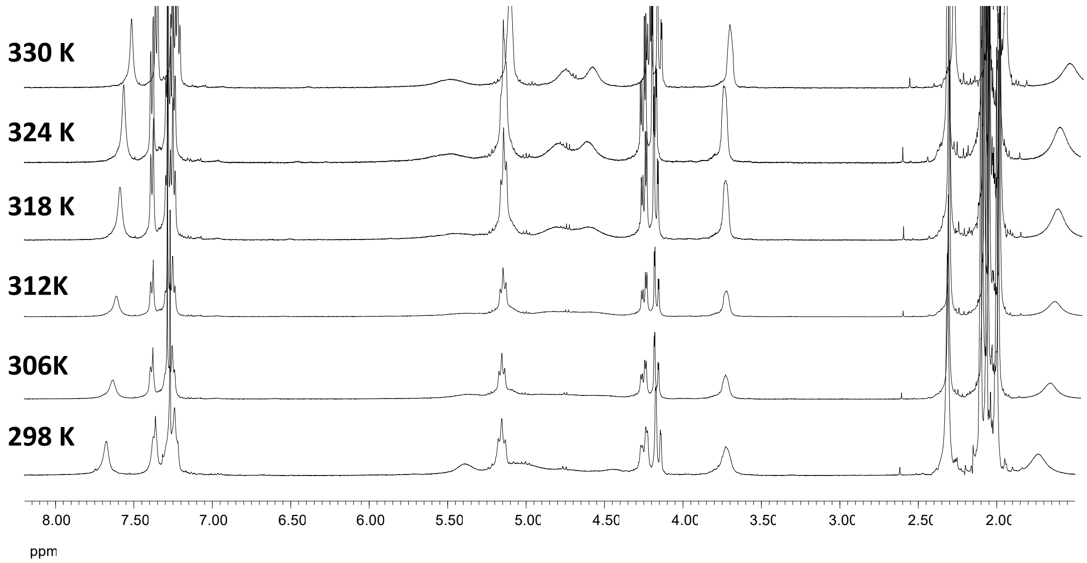

Figure S41 Variable-temperature ^{1}H NMR spectra of 3,4,6-tri-*O*-acetyl-2-deoxy-2-[3-(2-chloro-6-methylphenyl)thioureido]-β-D-glucopyranosyl azide (**25**) recorded in $CDCl_3$.

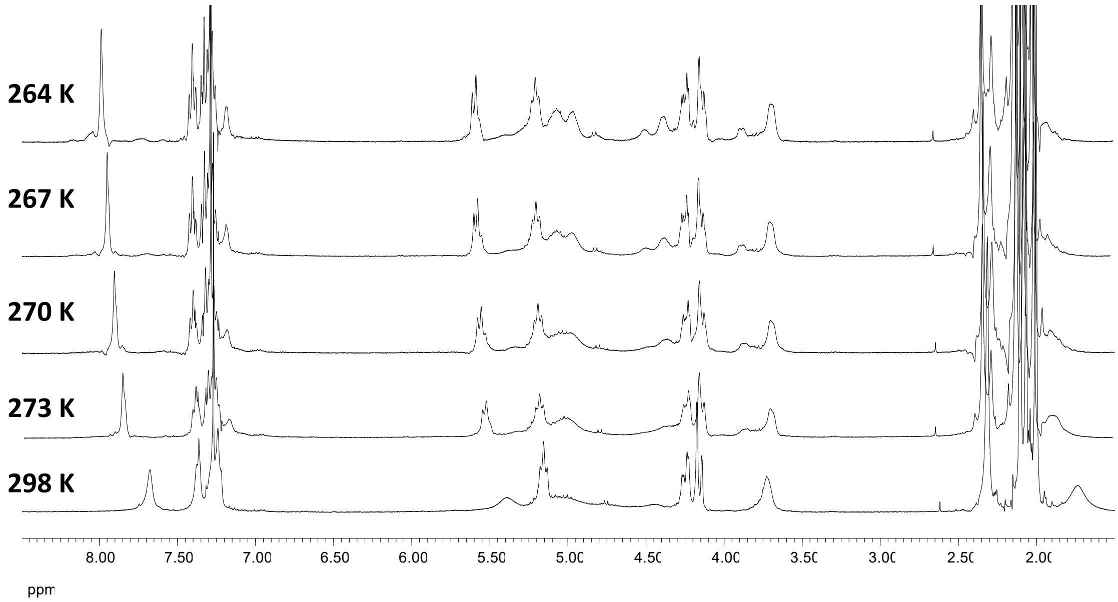

Figure S42 Variable-temperature ^{1}H NMR spectra of 3,4,6-tri-*O*-acetyl-2-deoxy-2-[3-(2-chloro-6-methylphenyl)thioureido]-β-D-glucopyranosyl azide (**25**) recorded in $CDCl_3$.

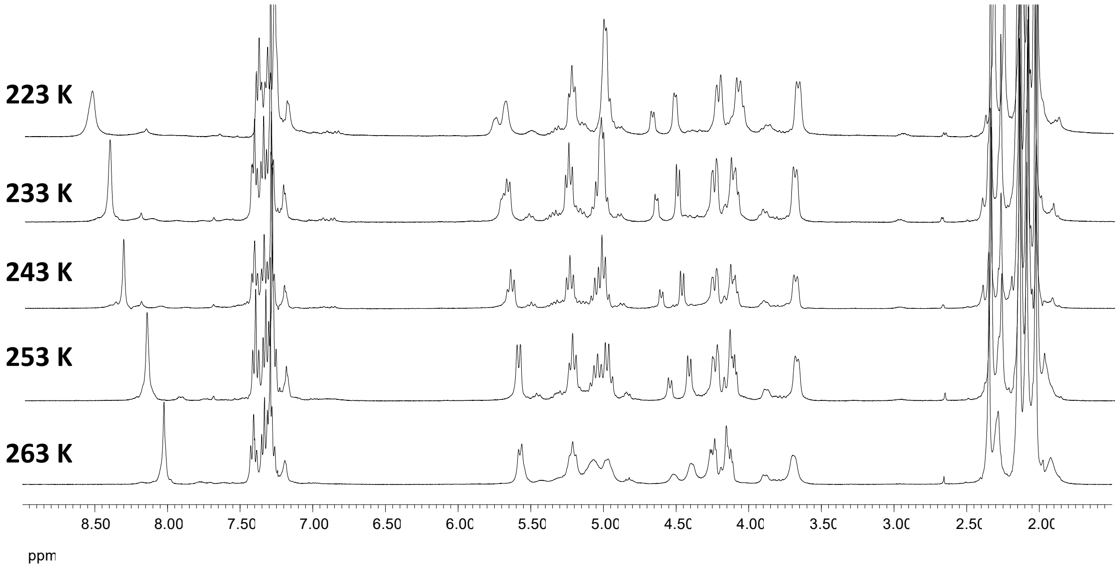

Figure S43 Variable-temperature ^{1}H NMR spectra of 3,4,6-tri-*O*-acetyl-2-deoxy-2-[3-(2-chloro-6-methylphenyl)thioureido]-β-D-glucopyranosyl azide (**25**) recorded in $CDCl_3$.

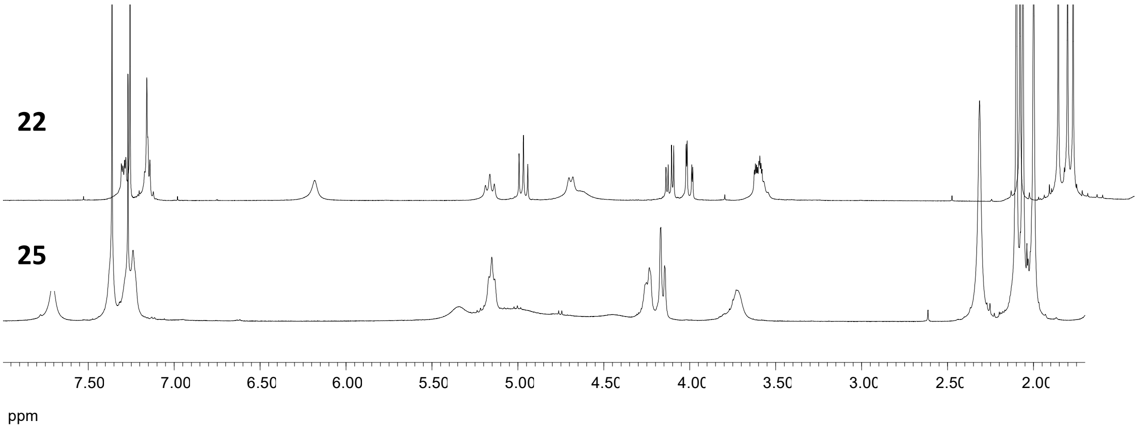

Figure S44 ^{1}H NMR spectrum of 3,4,6-tri-*O*-acetyl-2-deoxy-2-[3-(2-chloro-6-methylphenyl)ureido]-β-D-glucopyranosyl azide (**22**) and 3,4,6-tri-*O*-acetyl-2-deoxy-2-[3-(2-chloro-6-methylphenyl)thioureido]-β-D-glucopyranosyl azide (**25**) recorded in $CDCl_3$ at 330K.

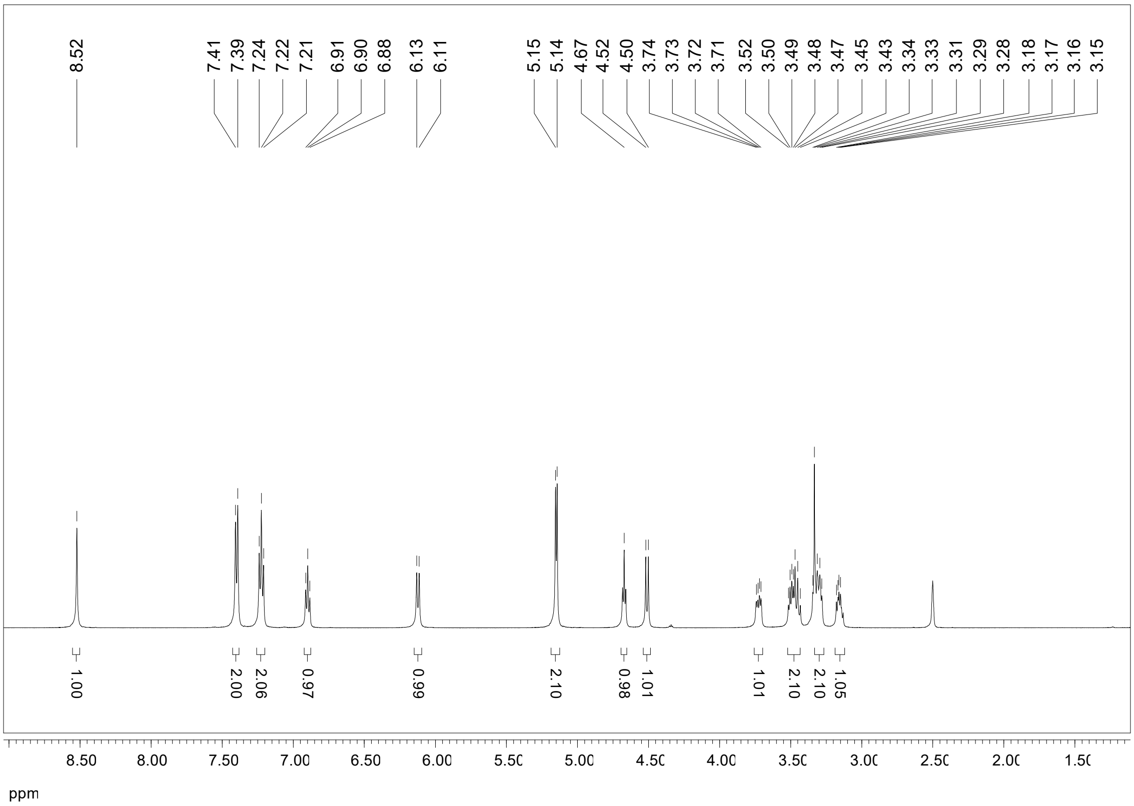

Figure S45 ^{1}H NMR spectrum of 2-deoxy-2-(3-phenylureido)-β-D-glucopyranosyl azide (**34**) recorded in DMSO-d_6.

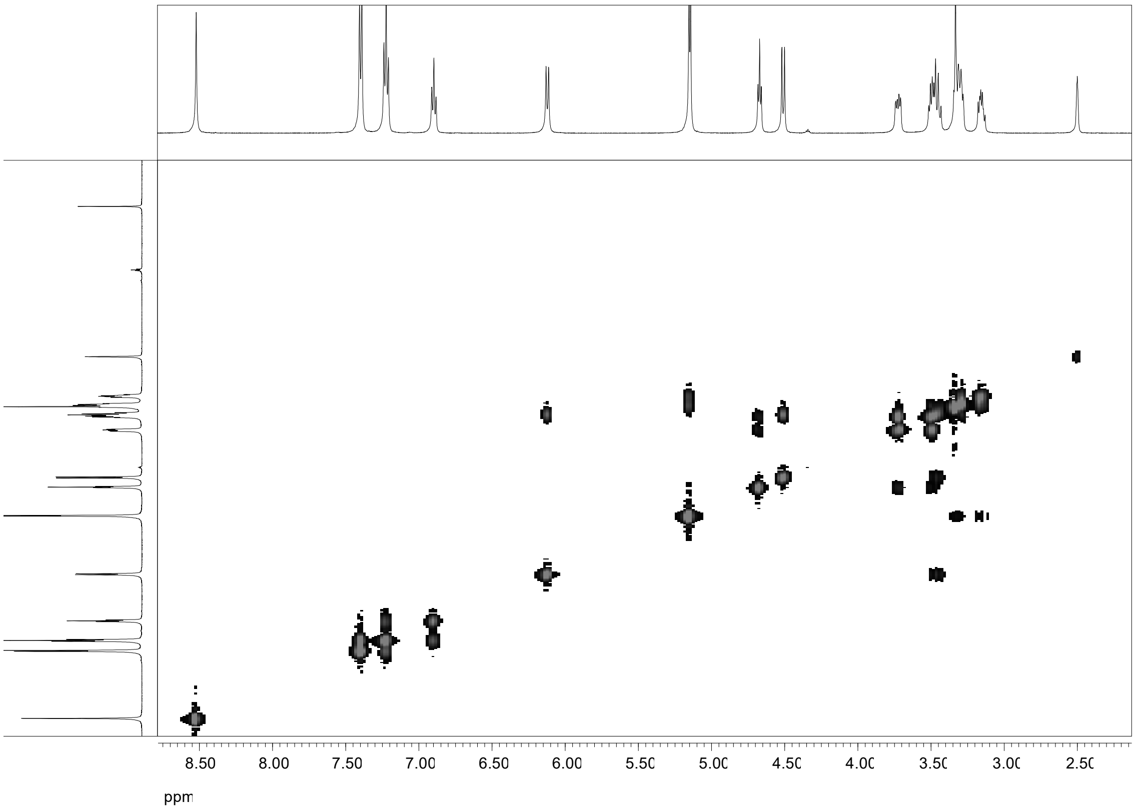

Figure S46 COSY spectrum 2-deoxy-2-(3-phenylureido)-β-D-glucopyranosyl azide (**34**) recorded in DMSO-d_6.

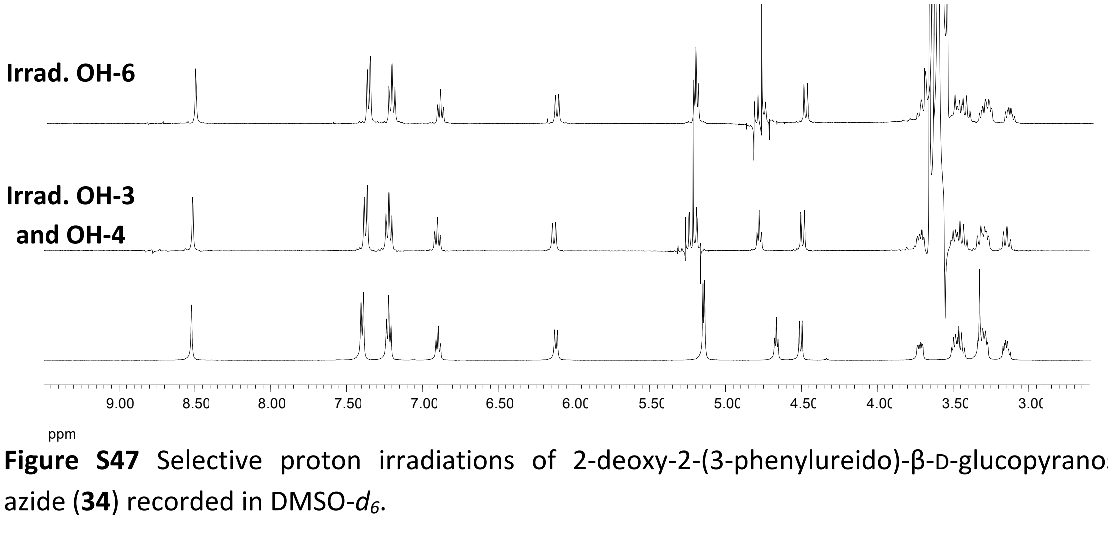

Figure S47 Selective proton irradiations of 2-deoxy-2-(3-phenylureido)-β-D-glucopyranosyl azide (**34**) recorded in DMSO-d_6.

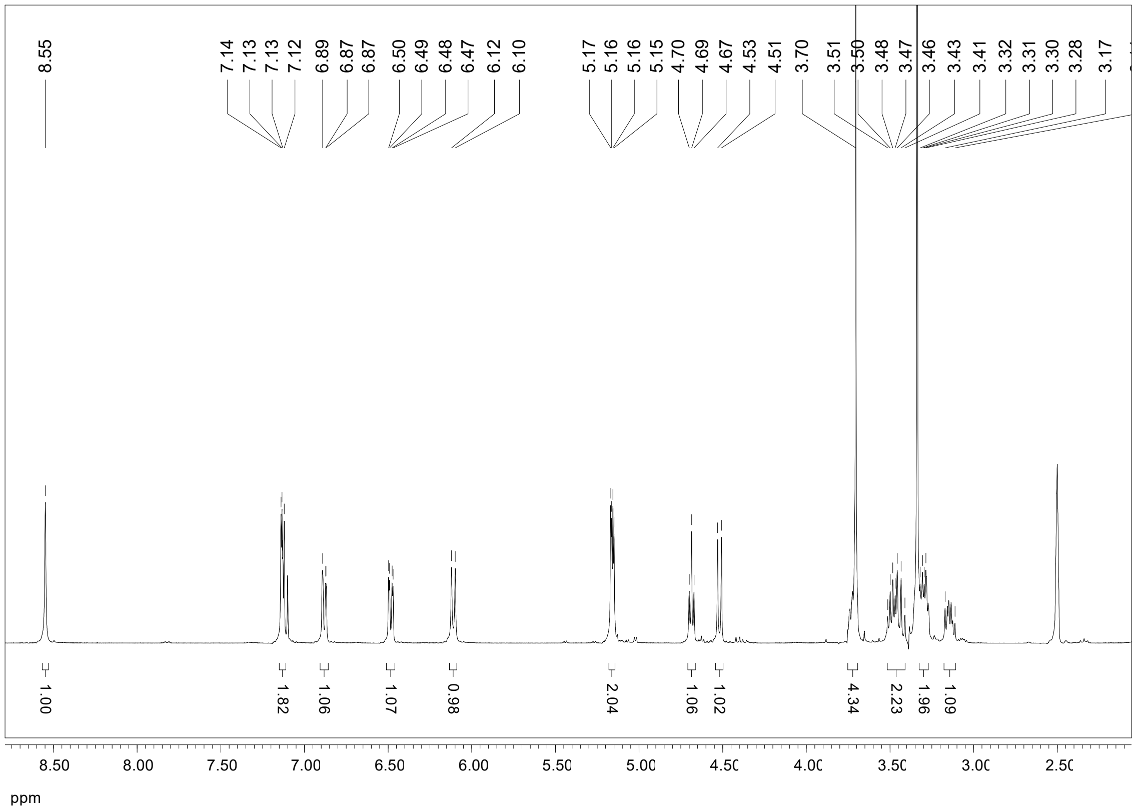

Figure S48 ^{1}H NMR spectrum of 2-deoxy-2-[3-(4-methoxyphenyl)ureido]-β-D-glucopyranosyl azide (**35**) recorded in DMSO-d_6.

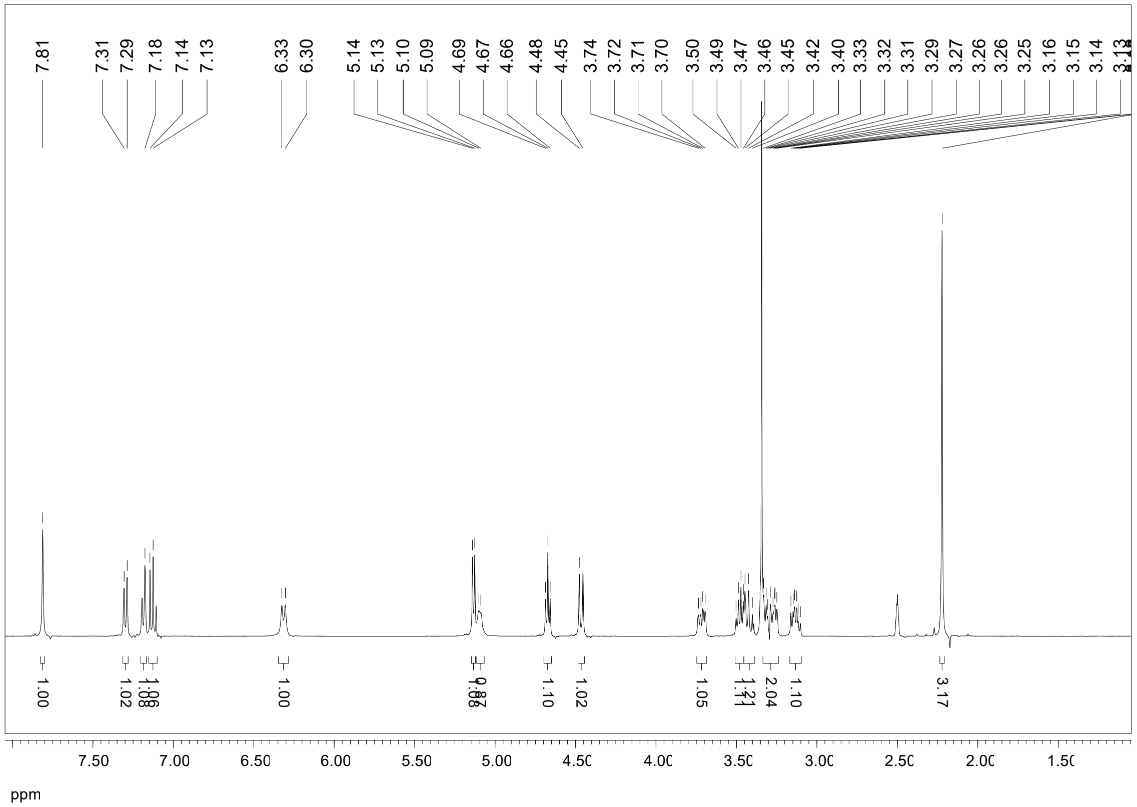

Figure S49 ^{1}H NMR spectrum of 2-deoxy-2-[3-(2-chloro-6-methylphenyl)ureido]-β-D-glucopyranosyl azide (**36**) recorded in DMSO-d_6.

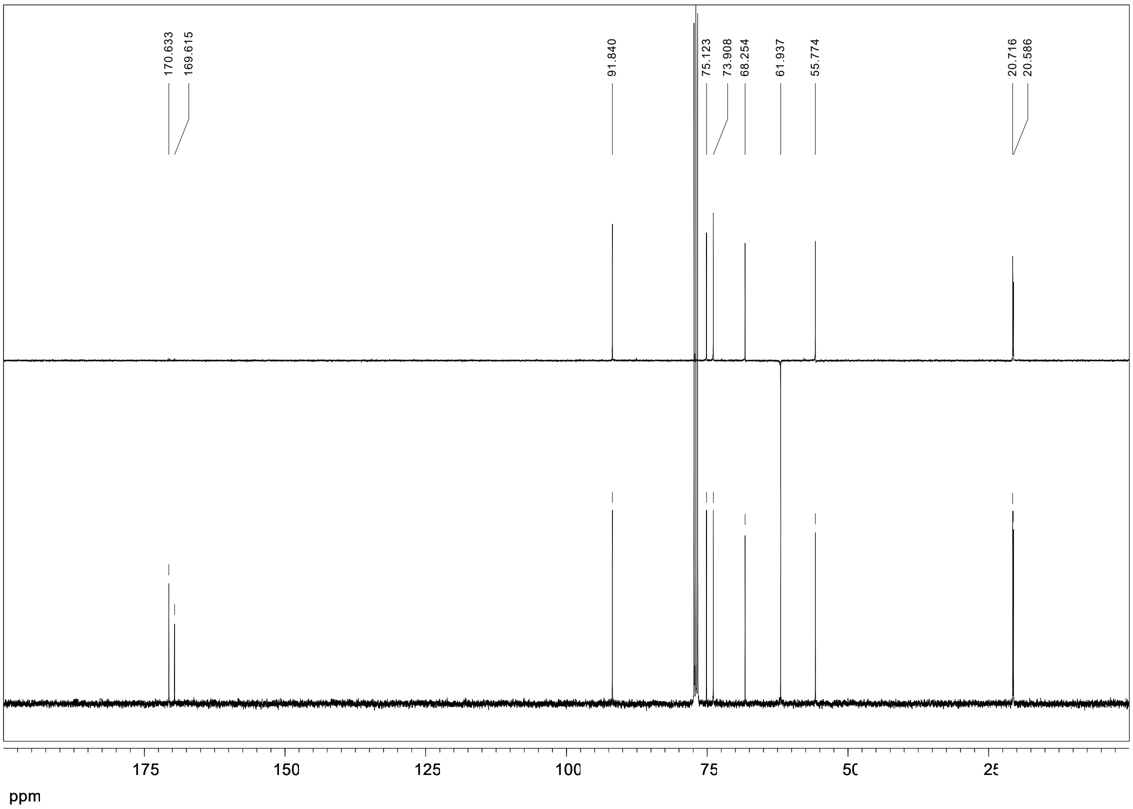

Figure S50 $^{13}C\{^{1}H\}$ NMR and DEPT spectra of 3,4,6-tri-O-acetyl-2-amino-2-deoxy-β-D-glucopyranosyl azide (**1**) recorded in $CDCl_3$.

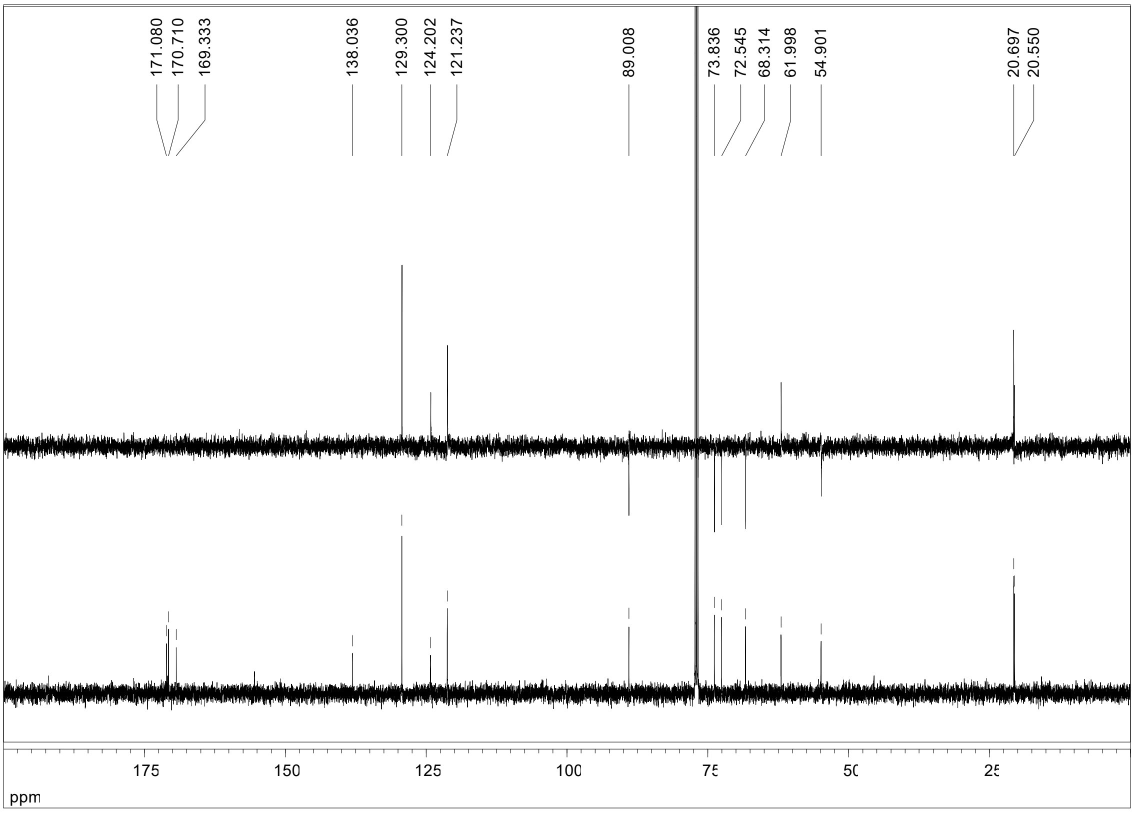

Figure S51 $^{13}C\{^{1}H\}$ NMR and DEPT spectra of 3,4,6-tri-*O*-acetyl-2-deoxy-2-(3-phenylureido)-β-D-glucopyranosyl azide (**14**) recorded in $CDCl_3$.

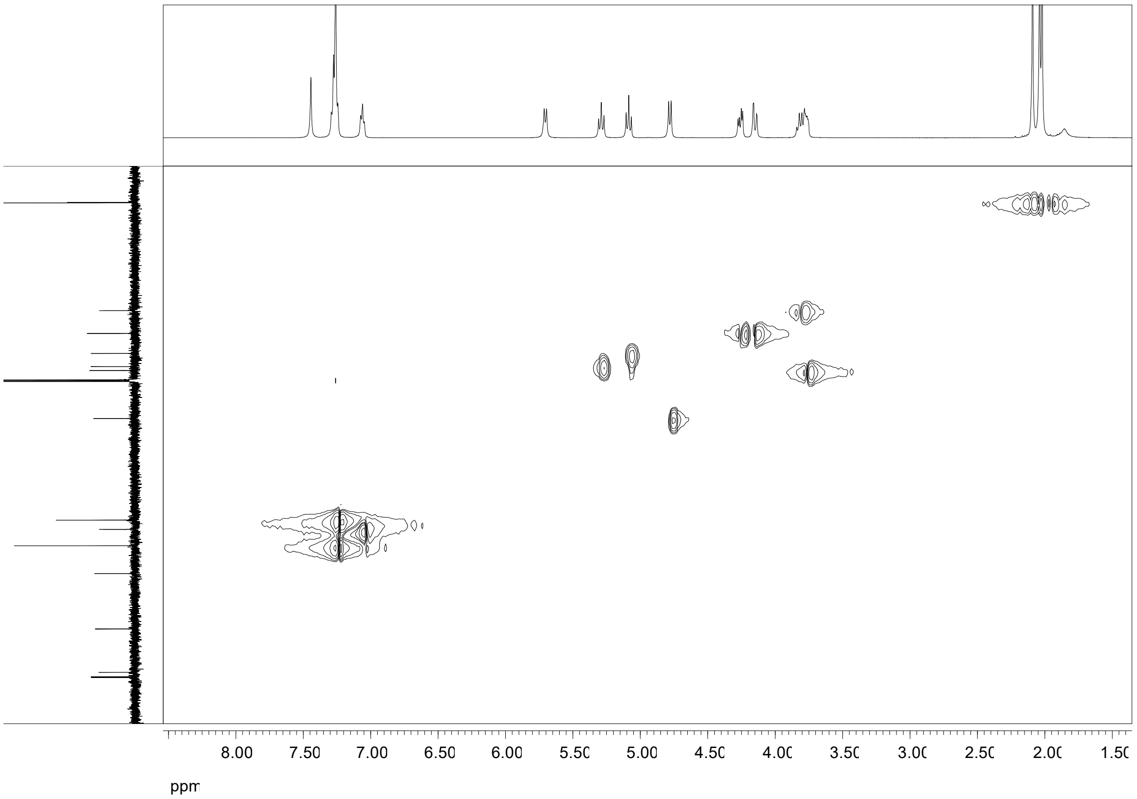

Figure S52 HMQC spectrum of 3,4,6-tri-*O*-acetyl-2-deoxy-2-(3-phenylureido)-β-D-glucopyranosyl azide (**14**) recorded in DCl3.

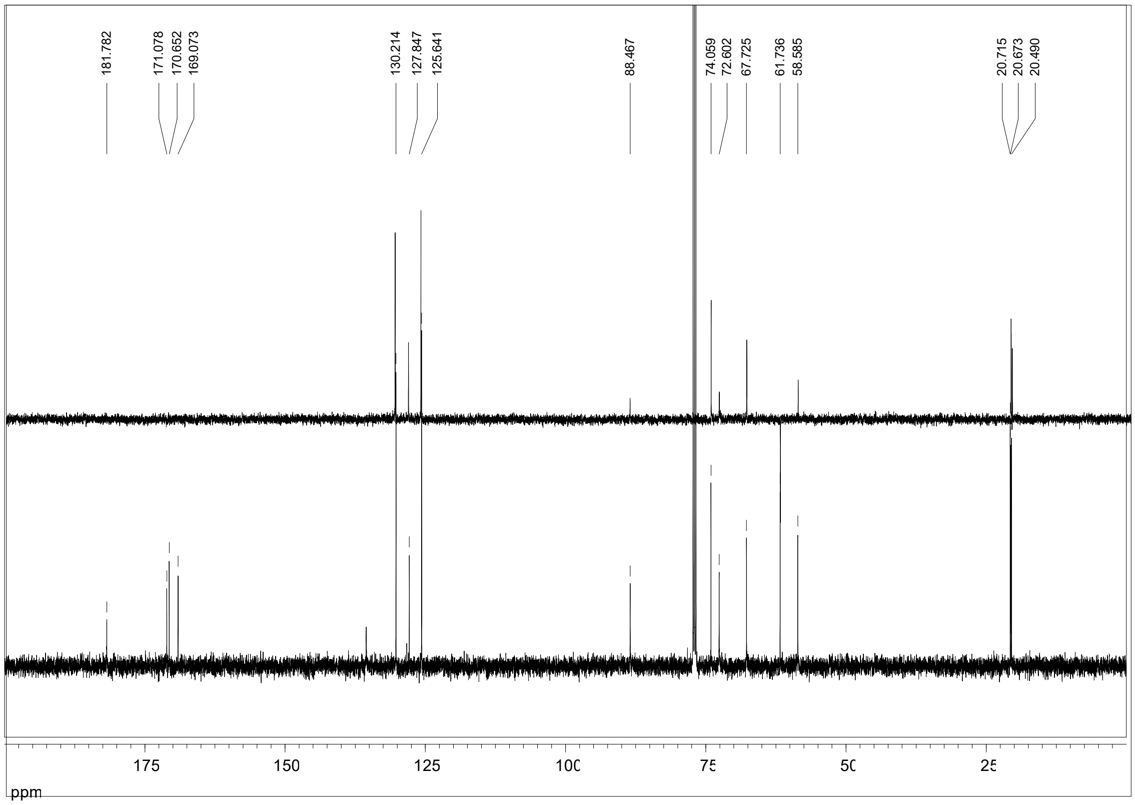

Figure S53 $^{13}C\{^{1}H\}$ NMR and DEPT spectra of 3,4,6-tri-*O*-acetyl-2-deoxy-2-(3-phenylthioureido)-β-D-glucopyranosyl azide (**15**) recorded in $CDCl_3$.

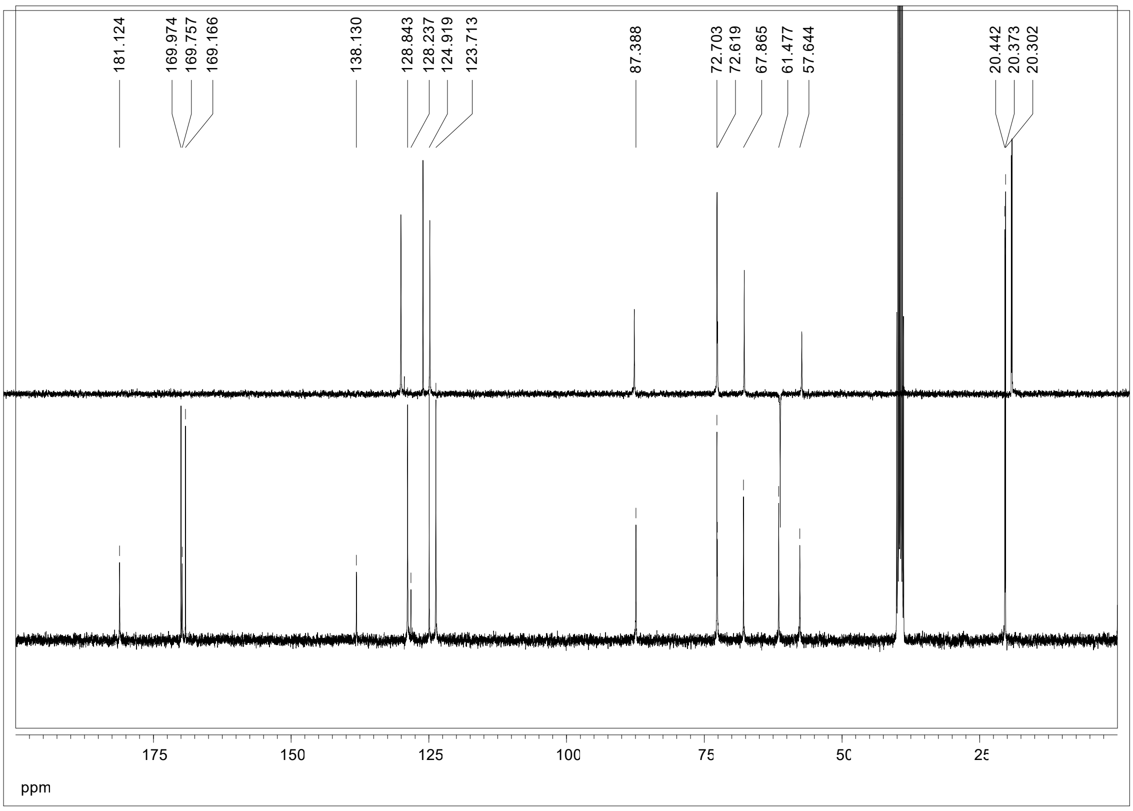

Figure S54 $^{13}C\{^{1}H\}$ NMR and DEPT spectra of 3,4,6-tri-*O*-acetyl-2-deoxy-2-(3-phenylthioureido)-β-D-glucopyranosyl azide (**15**) recorded in DMSO-d_6.

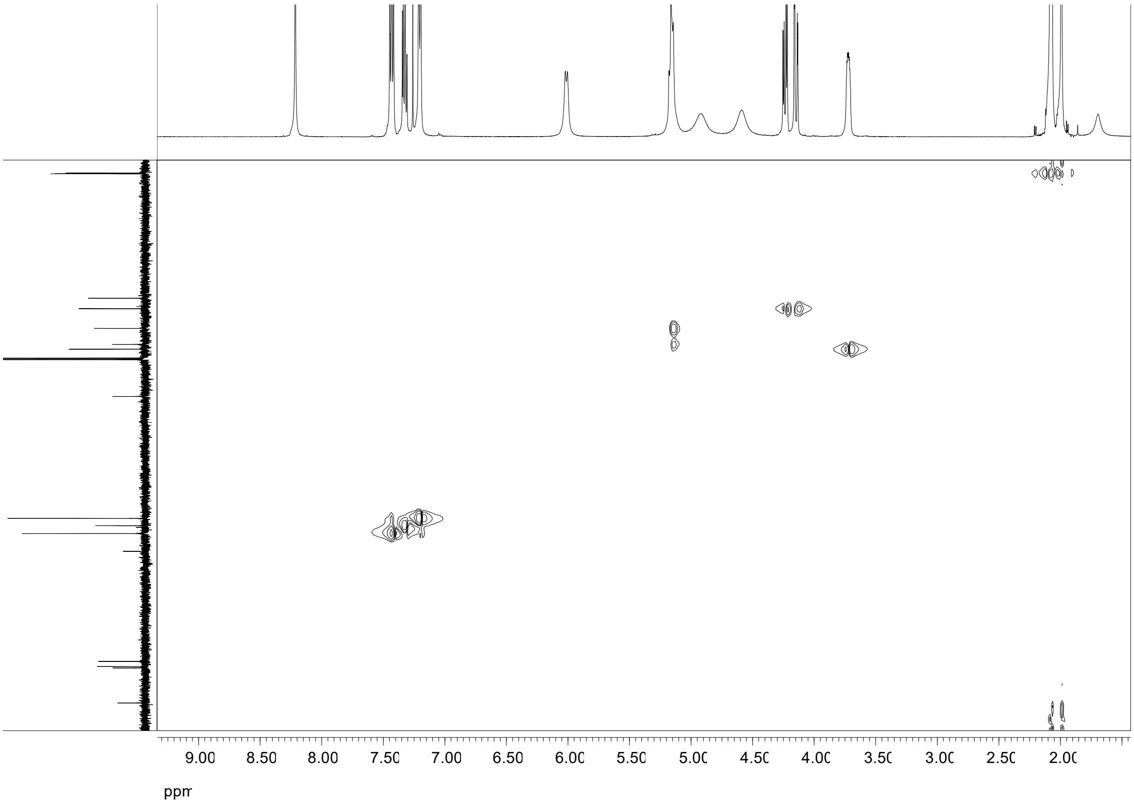

Figure S55 HMQC spectrum of 3,4,6-tri-*O*-acetyl-2-deoxy-2-(3-phenylthioureido)-β-D-glucopyranosyl azide (**15**) recorded in $CDCl_3$.

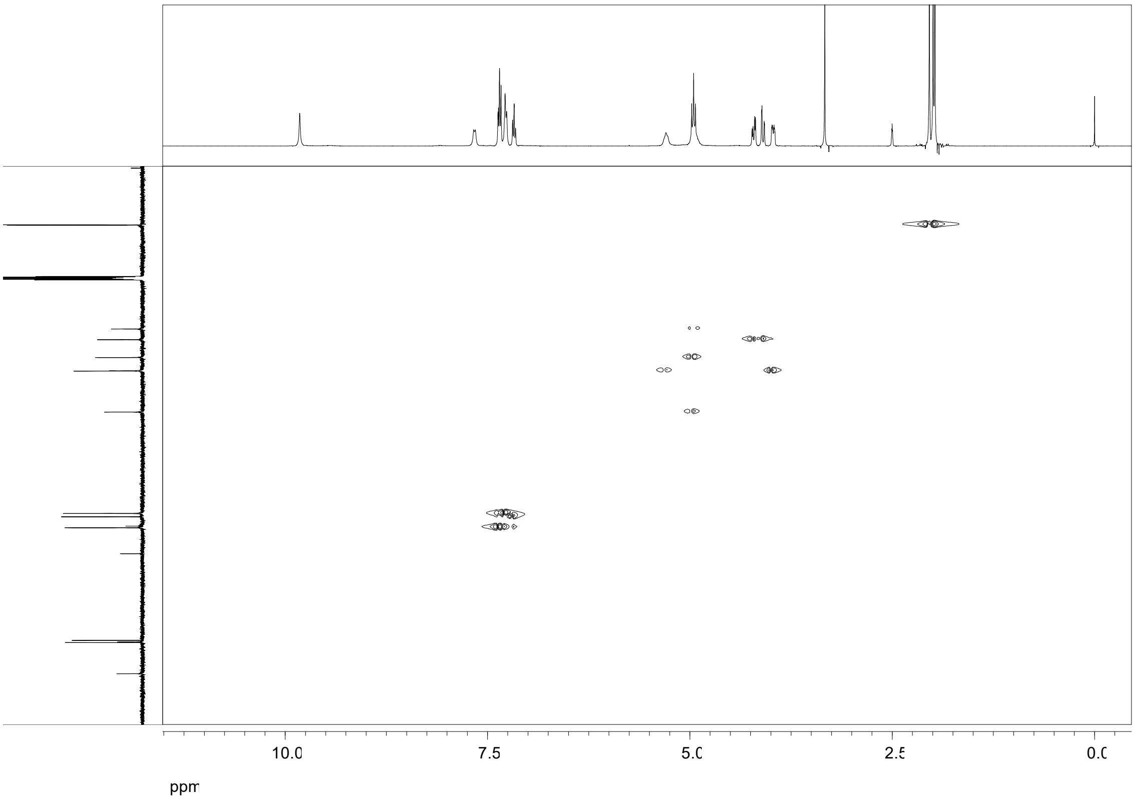

Figure S56 HMQC spectrum of 3,4,6-tri-*O*-acetyl-2-deoxy-2-(3-phenylthioureido)-β-D-glucopyranosyl azide (**15**) recorded in DMSO-*d6*.

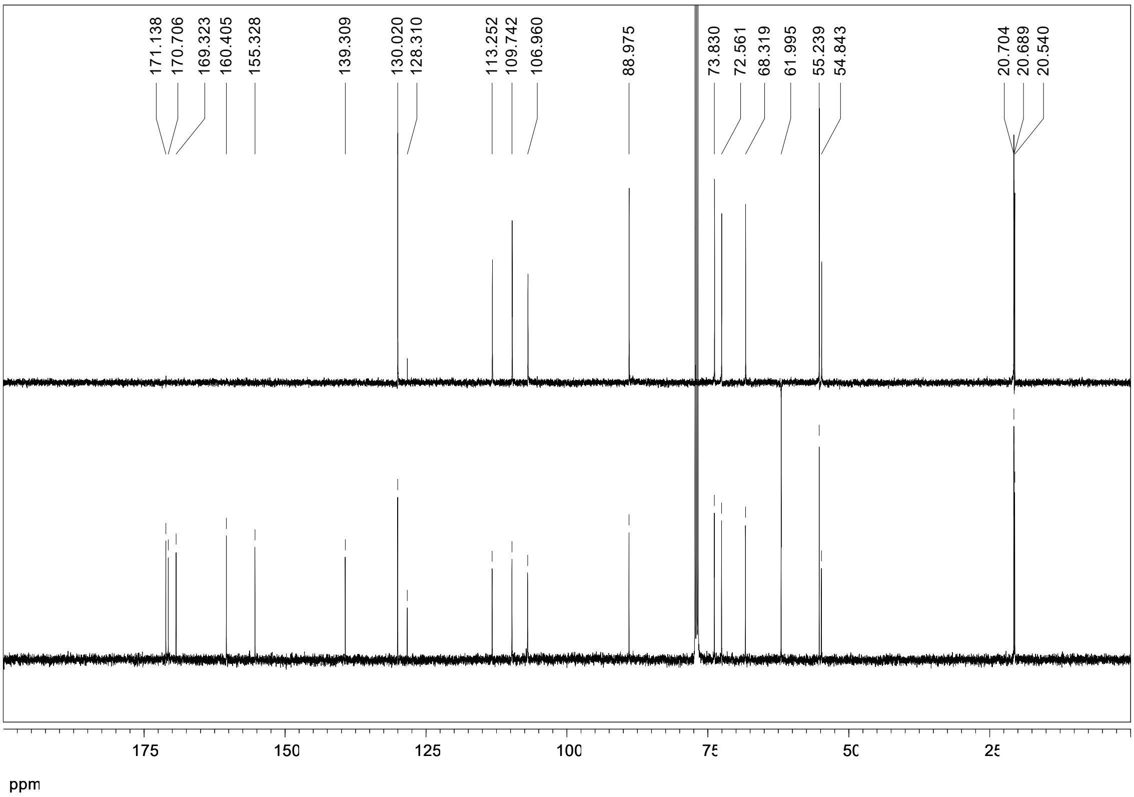

Figure S57 $^{13}C\{^{1}H\}$ NMR and DEPT spectra of 3,4,6-tri-*O*-acetyl-2-deoxy-2-[3-(3-methoxyphenyl)ureido]-β-D-glucopyranosyl azide (**16**) recorded in $CDCl_3$.

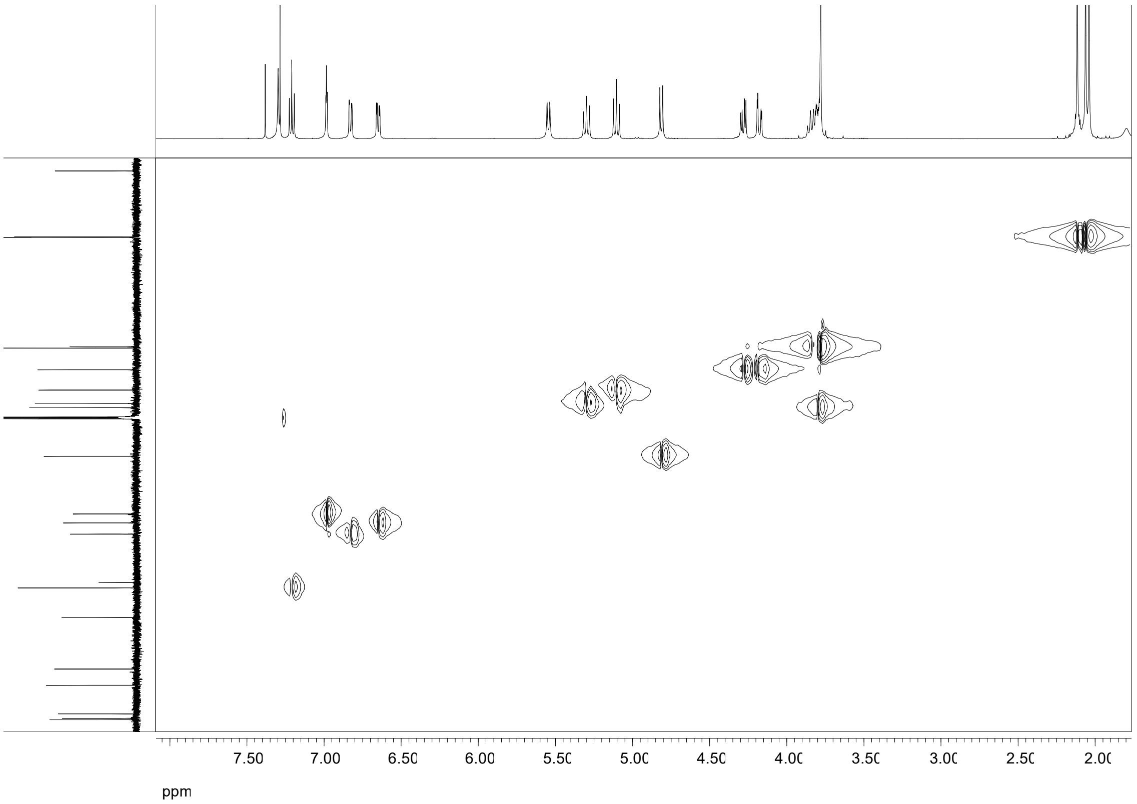

Figure S58 HMQC spectrum of 3,4,6-tri-*O*-acetyl-2-deoxy-2-[3-(3-methoxyphenyl)ureido]-β-D-glucopyranosyl azide (**16**) recorded in $CDCl_3$.

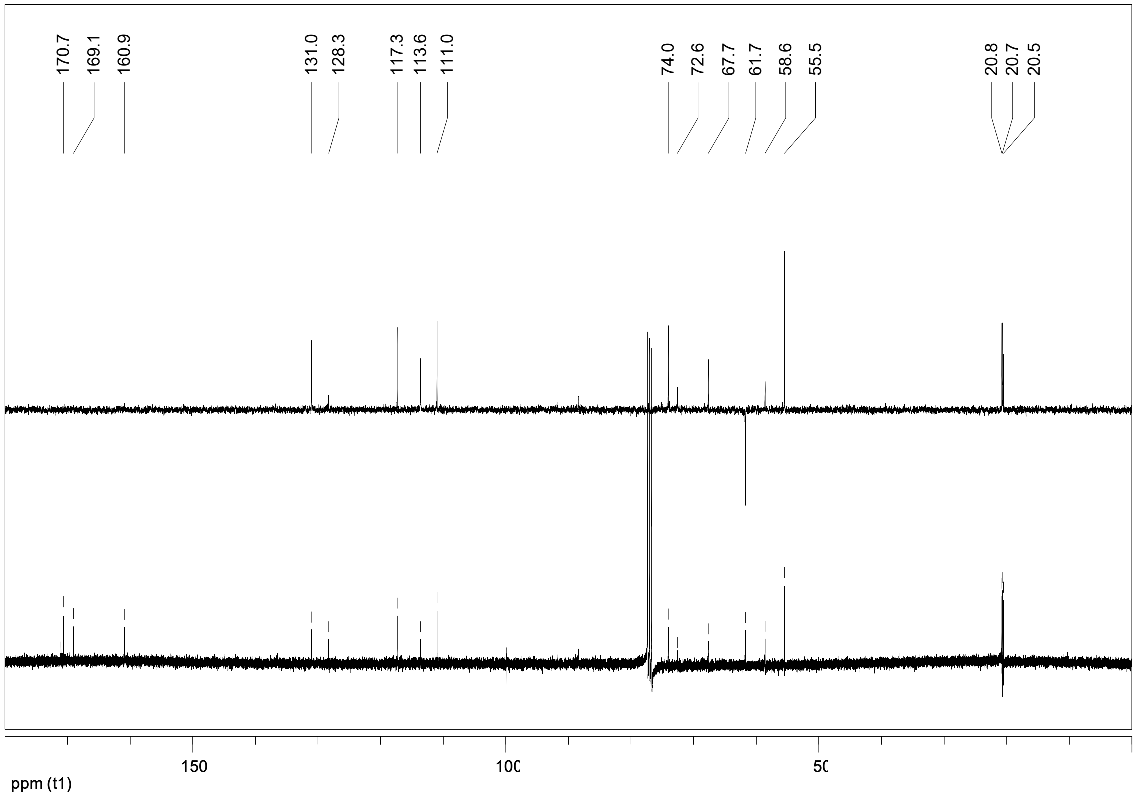

Figure S59 $^{13}C\{^{1}H\}$ NMR and DEPT spectra of 3,4,6-tri-*O*-acetyl-2-deoxy-2-[3-(3-methoxyphenyl)thioureido]-β-D-glucopyranosyl azide (**17**) recorded in $CDCl_3$.

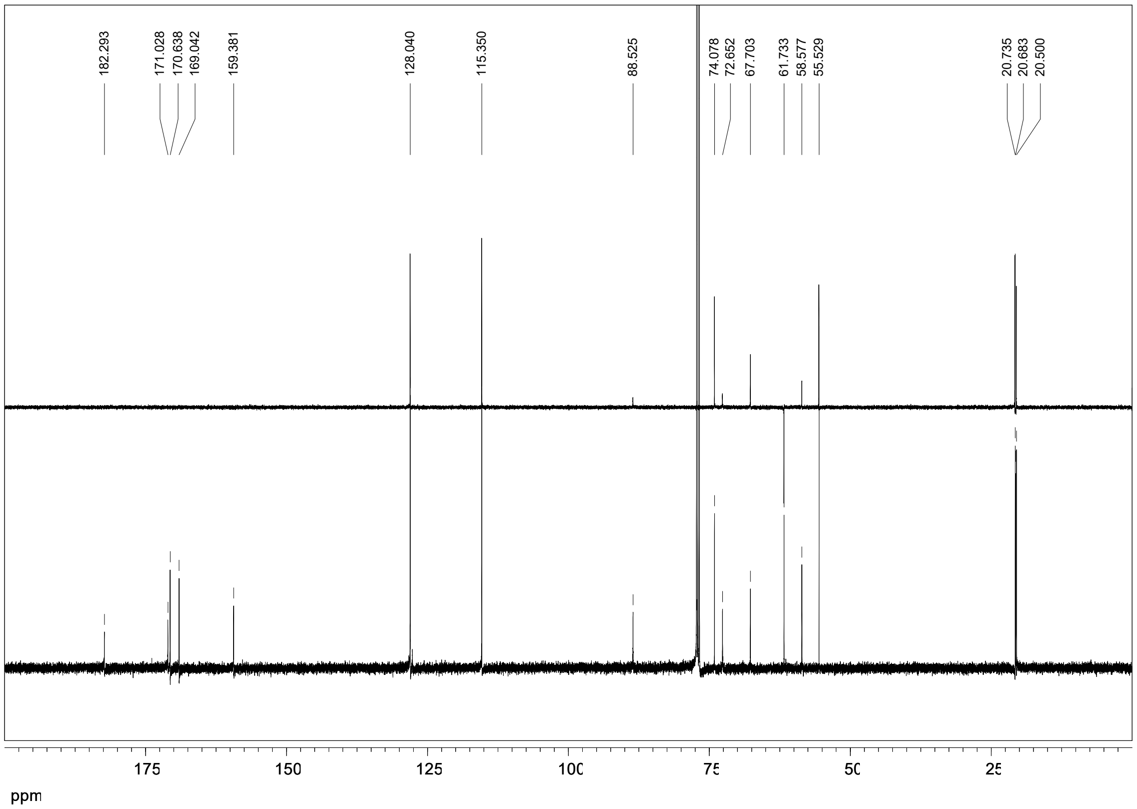

Figure S60 $^{13}C\{^{1}H\}$ NMR and DEPT spectra of 3,4,6-tri-*O*-acetyl-2-deoxy-2-[3-(4-methoxyphenyl)ureido]-β-D-glucopyranosyl azide (**18**) recorded in $CDCl_3$.

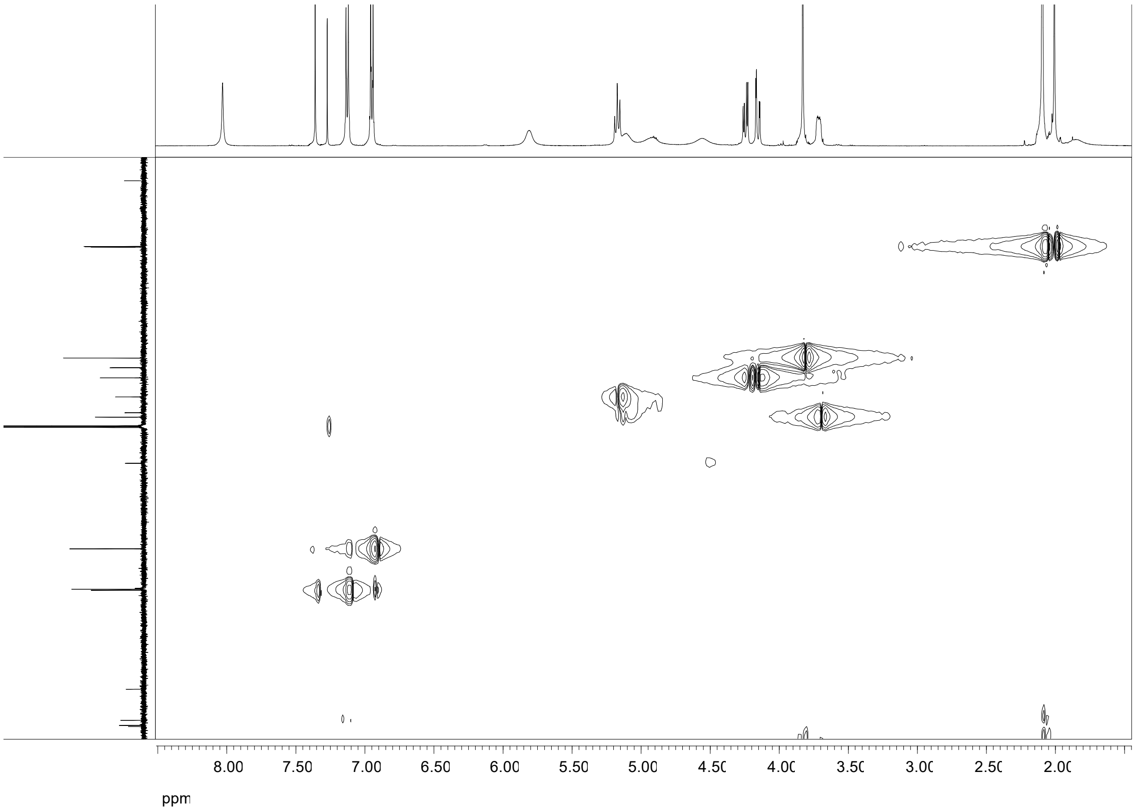

Figure S61 HMQC spectrum of 3,4,6-tri-*O*-acetyl-2-deoxy-2-[3-(4-methoxyphenyl)ureido]-β-D-glucopyranosyl azide (**18**) recorded in $CDCl_3$.

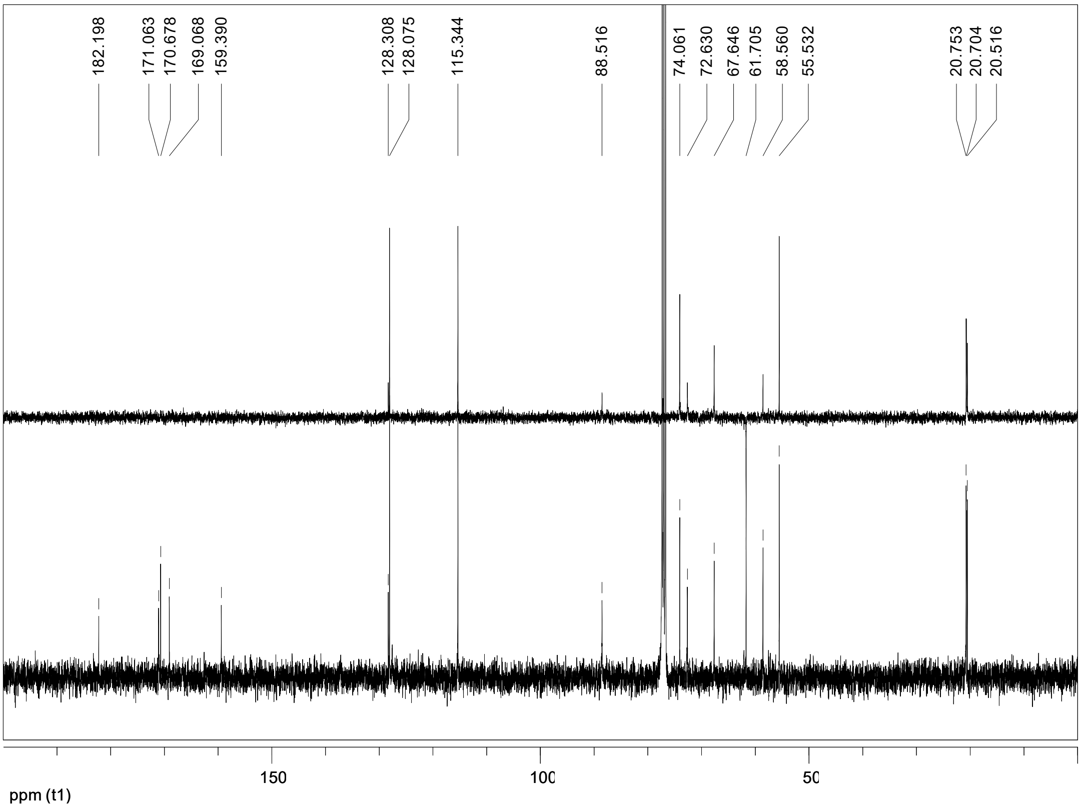

Figure S62 $^{13}C\{^{1}H\}$ NMR and DEPT spectra of 3,4,6-tri-*O*-acetyl-2-deoxy-2-[3-(4-methoxyphenyl)thioureido]-β-D-glucopyranosyl azide (**19**) recorded in $CDCl_3$.

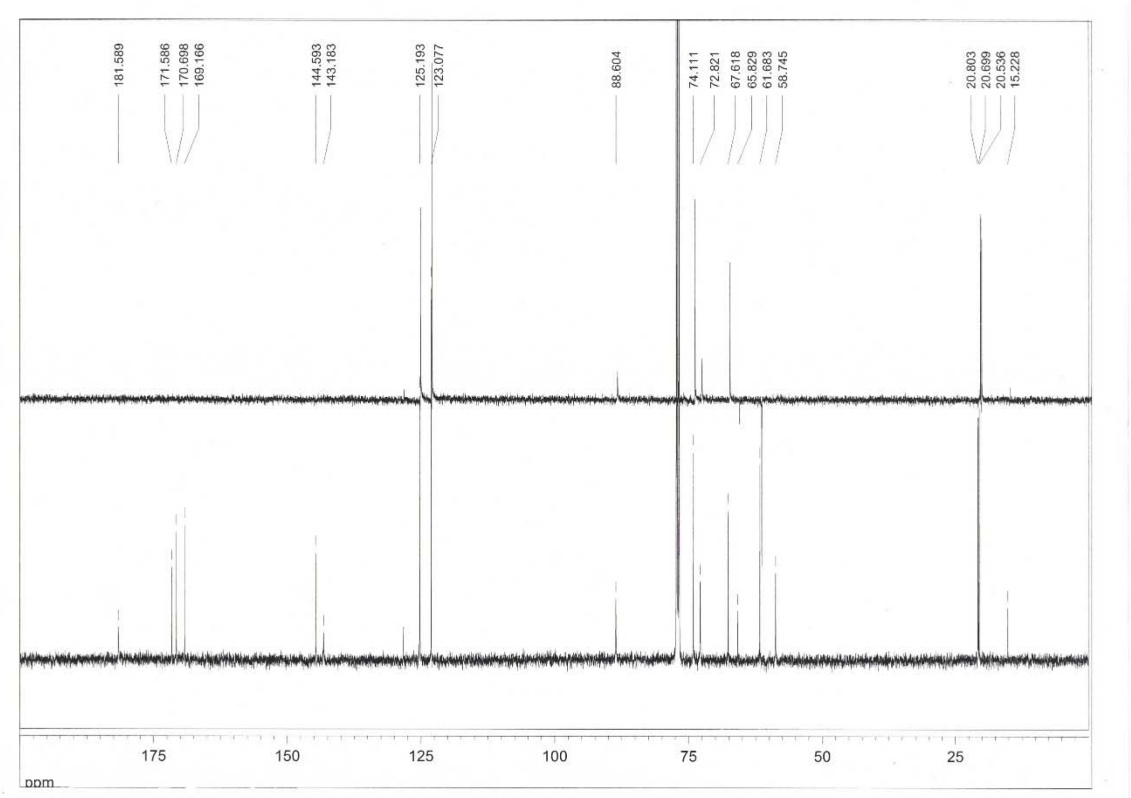

Figure S63 $^{13}C\{^{1}H\}$ NMR and DEPT spectra of 3,4,6-tri-*O*-acetyl-2-deoxy-2-[3-(2-fluorophenyl)thioureido]-β-D-glucopyranosyl azide (**20**) recorded in $CDCl_3$.

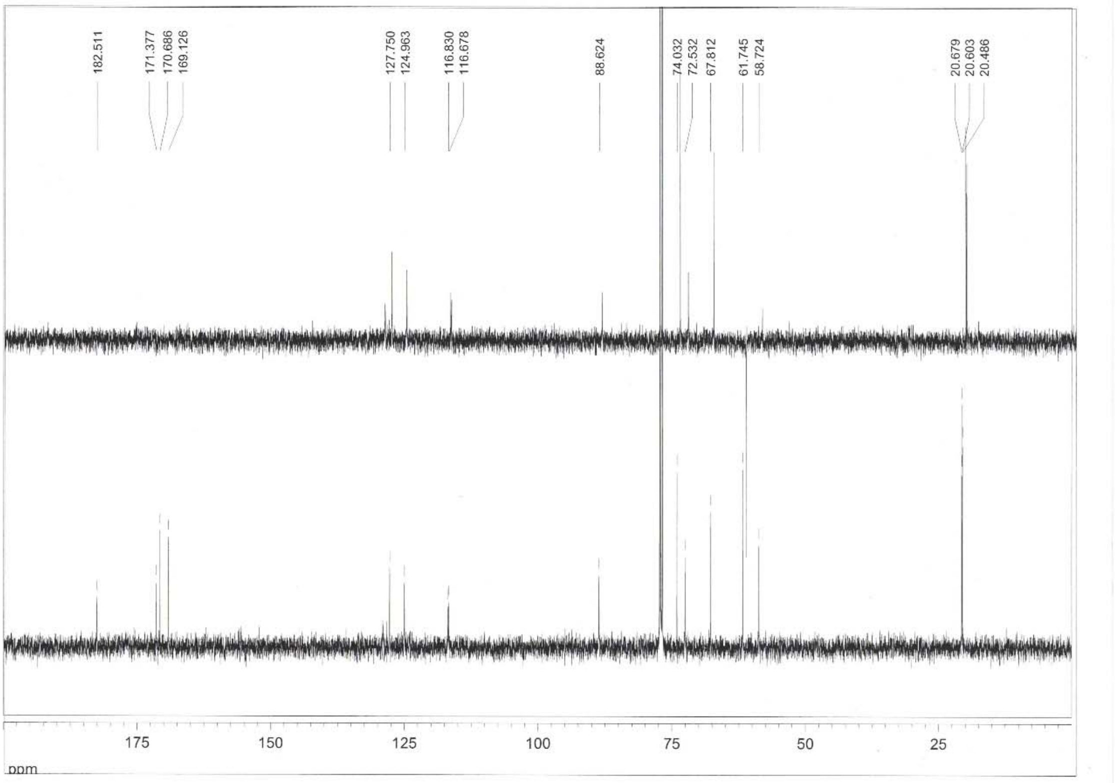

Figure S64 $^{13}C\{^{1}H\}$ NMR and DEPT spectra of 3,4,6-tri-*O*-acetyl-2-deoxy-2-[3-(4-nitrophenyl)thioureido]-β-D-glucopyranosyl azide (**21**) recorded in $CDCl_3$.

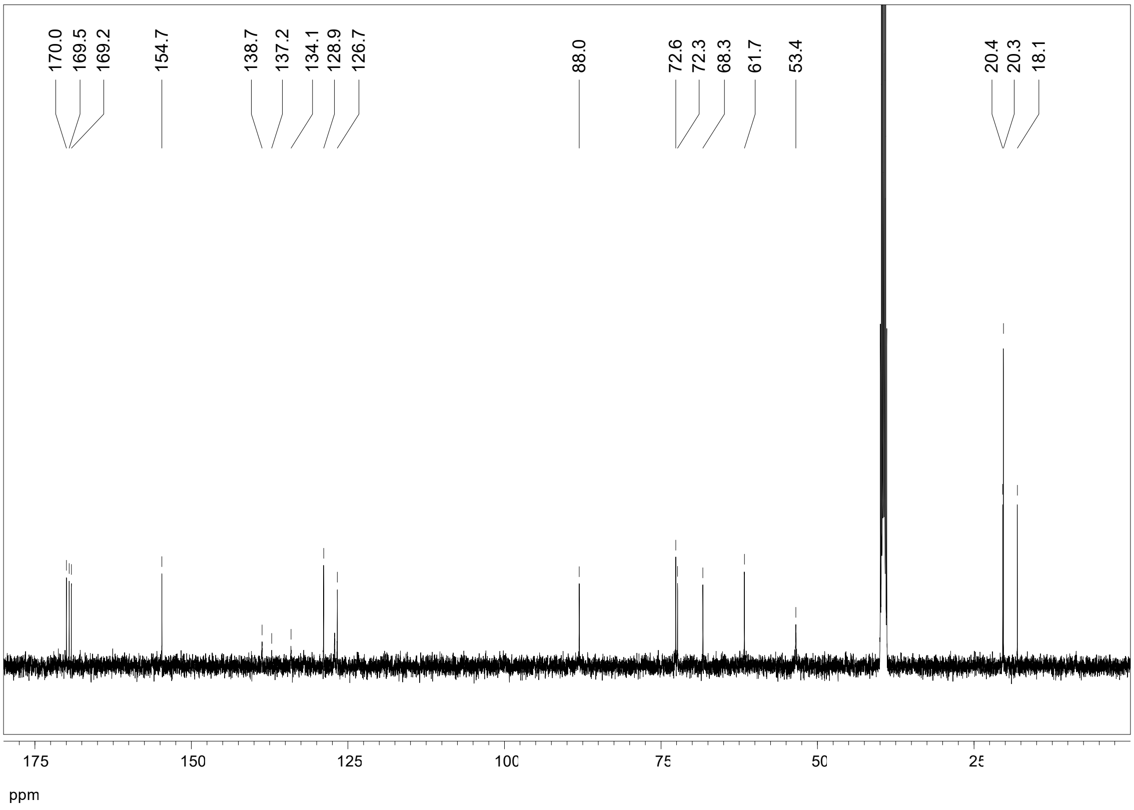

Figure S65 $^{13}C\{^{1}H\}$ NMR spectra of 3,4,6-tri-*O*-acetyl-2-deoxy-2-[3-(2-chloro-6-methylphenyl)ureido]-β-D-glucopyranosyl azide (**22**) recorded in DMSO-d_6.

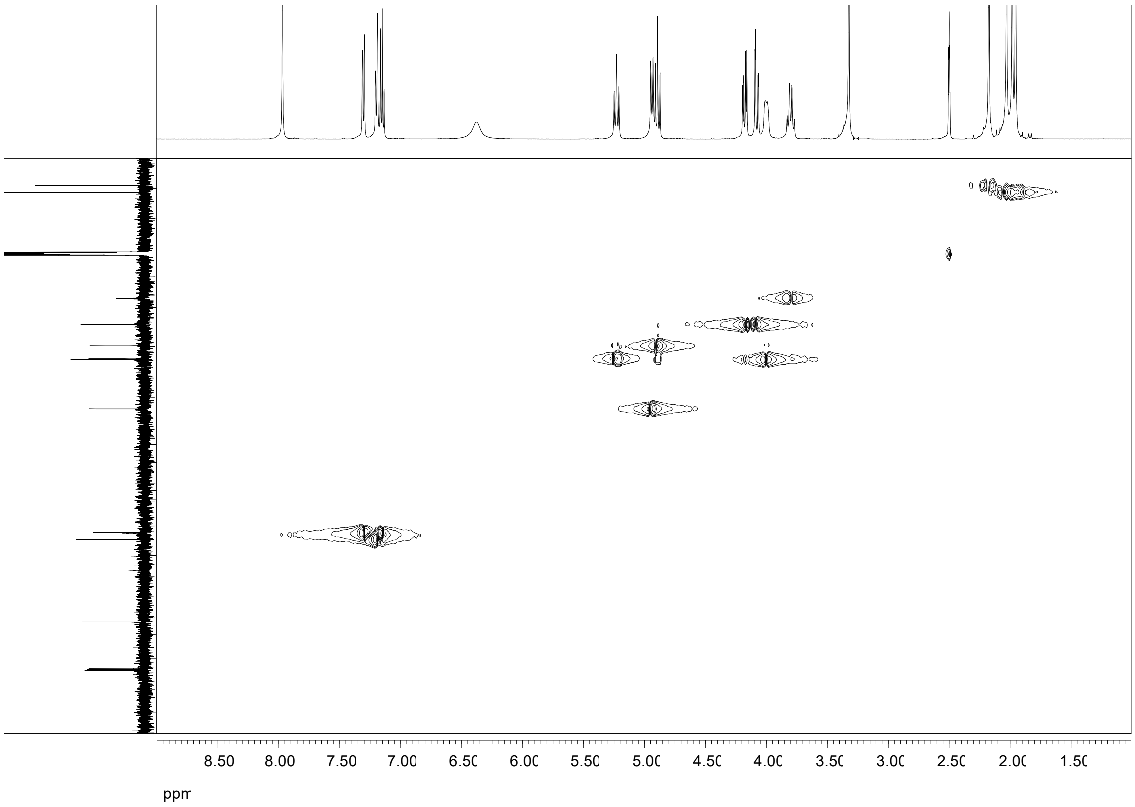

Figure S66 HMQC spectrum of 3,4,6-tri-*O*-acetyl-2-deoxy-2-[3-(2-chloro-6-methylphenyl)ureido]-β-D-glucopyranosyl azide (**22**) recorded in DMSO-d_6.

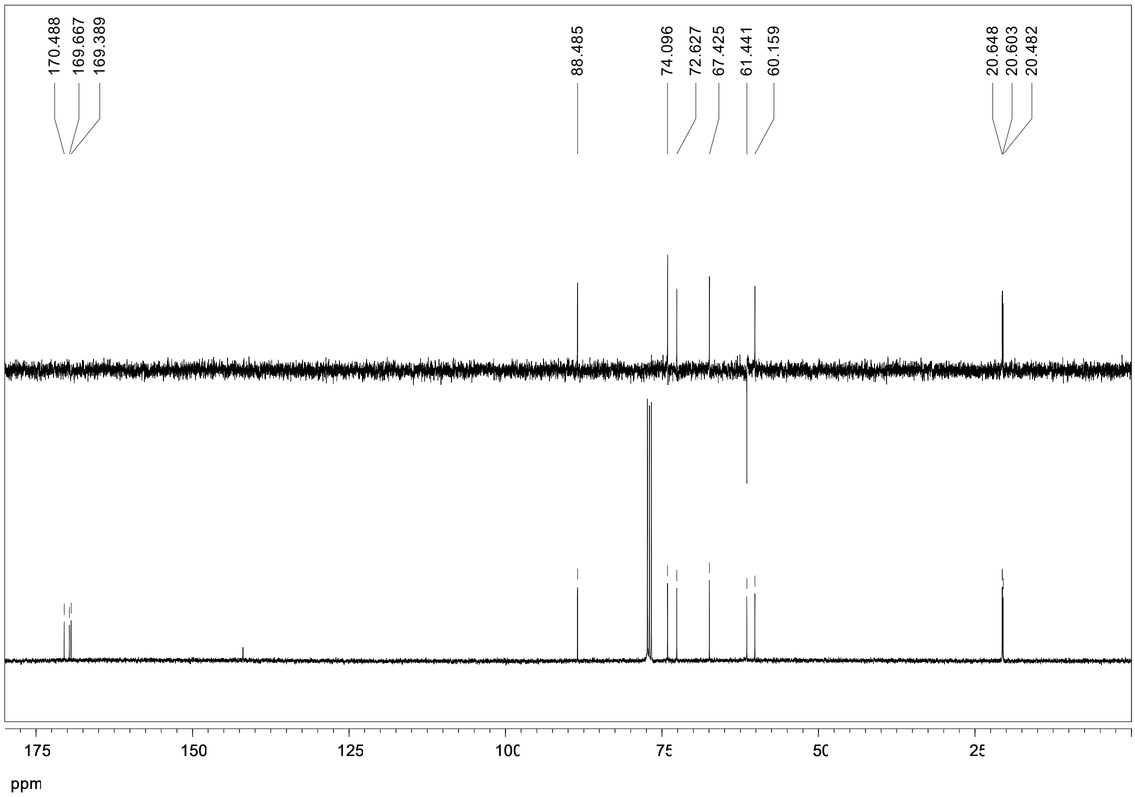

Figure S67 ^{13}C{^{1}H} NMR and DEPT spectra of 3,4,6-tri-*O*-acetyl-2-deoxy-2-isothiocianato-β-D-glucopyranosyl azide (**23**) recorded in $CDCl_3$.

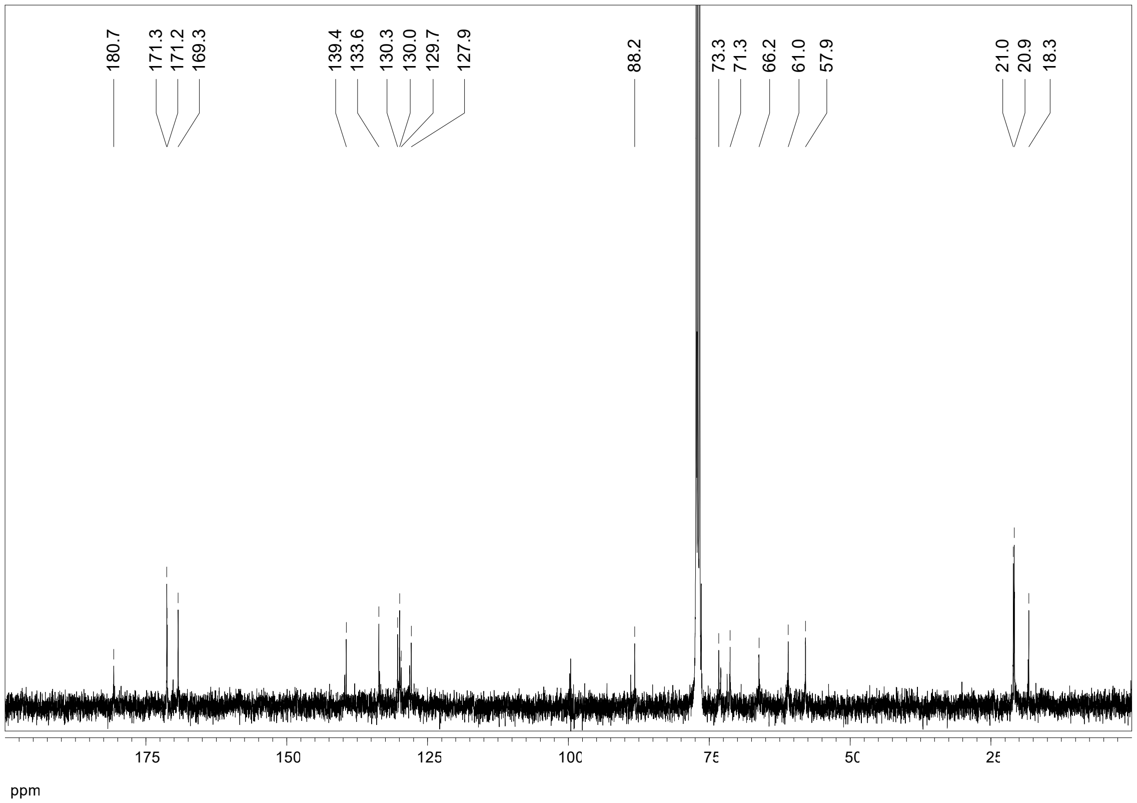

Figure S68 $^{13}C\{^1H\}$ NMR spectrum of 3,4,6-tri-*O*-acetyl-2-deoxy-2-[3-(2-chloro-6-methylphenyl)thioureido]-β-D-glucopyranosyl azide (**25**) recorded in $CDCl_3$ at 223K.

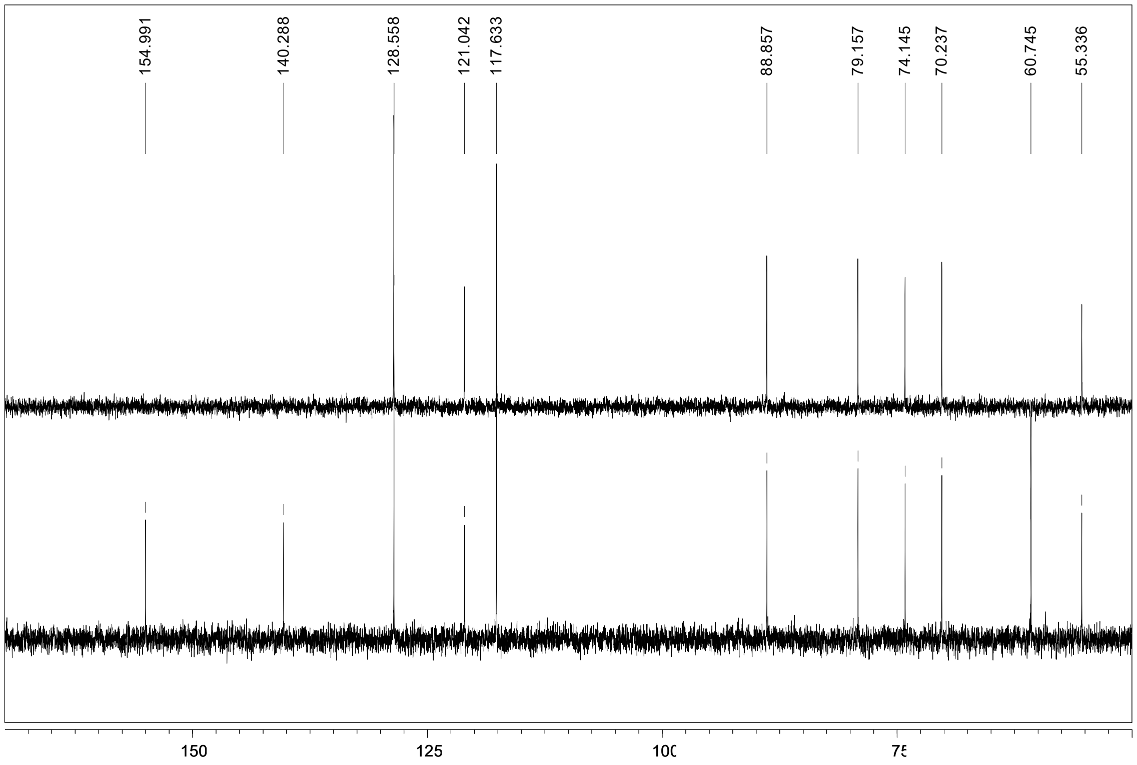

Figure S69 $^{13}C\{^1H\}$ NMR and DEPT spectra of 2-deoxy-2-(3-phenylureido)-β-D-glucopyranosyl azide (**34**) recorded in DMSO-d_6.

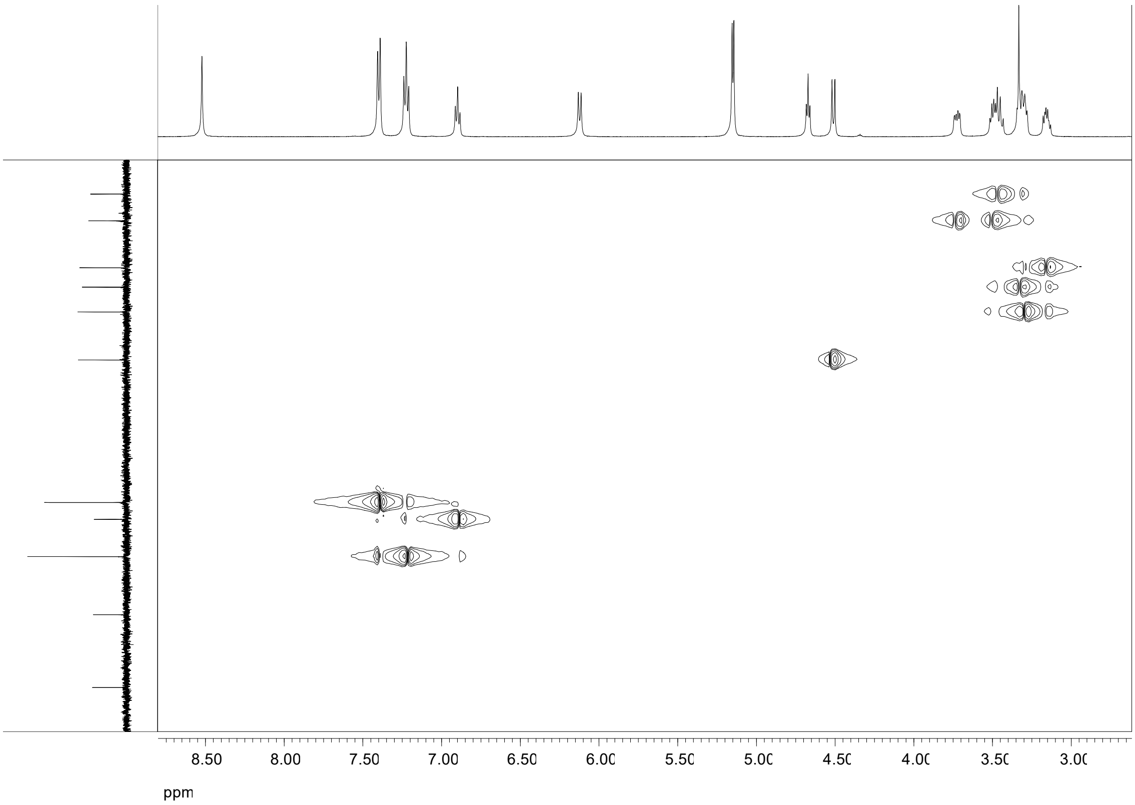

Figure S70 HMQC spectrum of 2-deoxy-2-(3-phenylureido)-β-D-glucopyranosyl azide (**34**) recorded in DMSO-d_6.

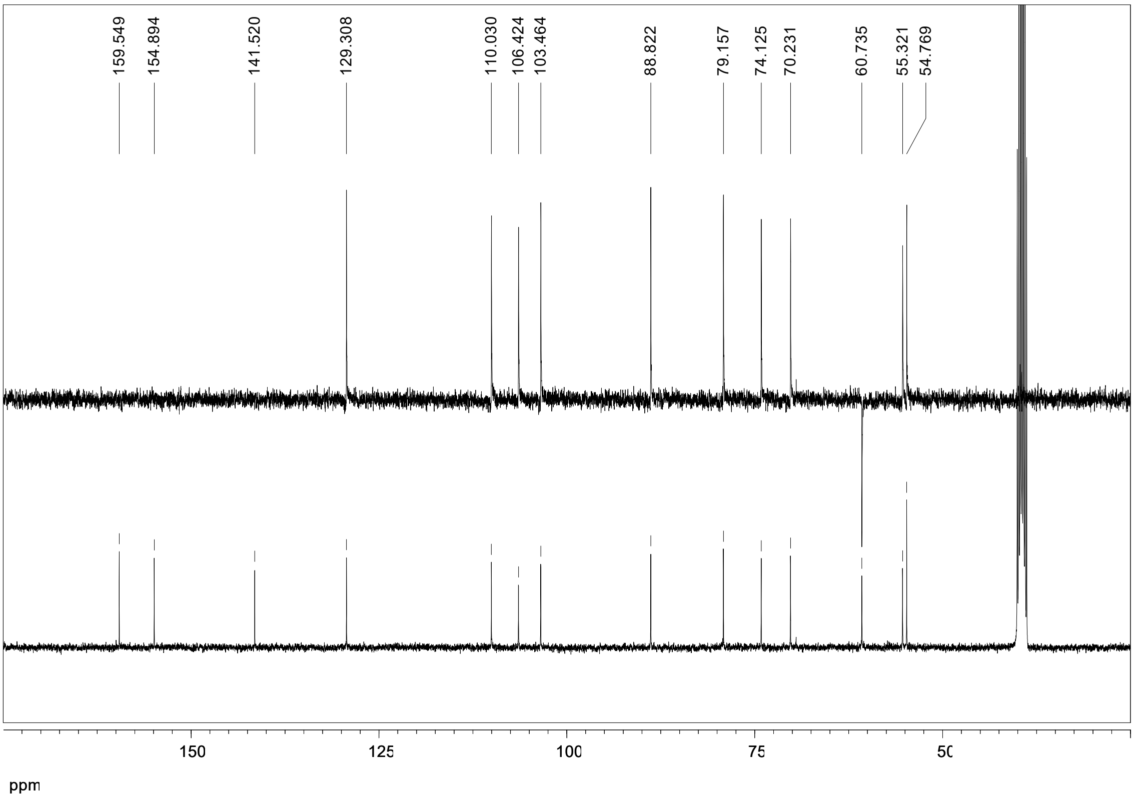

Figure S71 $^{13}C\{^{1}H\}$ NMR and DEPT spectra of 2-deoxy-2-[3-(4-methoxyphenyl)ureido]-β-D-glucopyranosyl azide (**35**) recorded in DMSO-d_6.

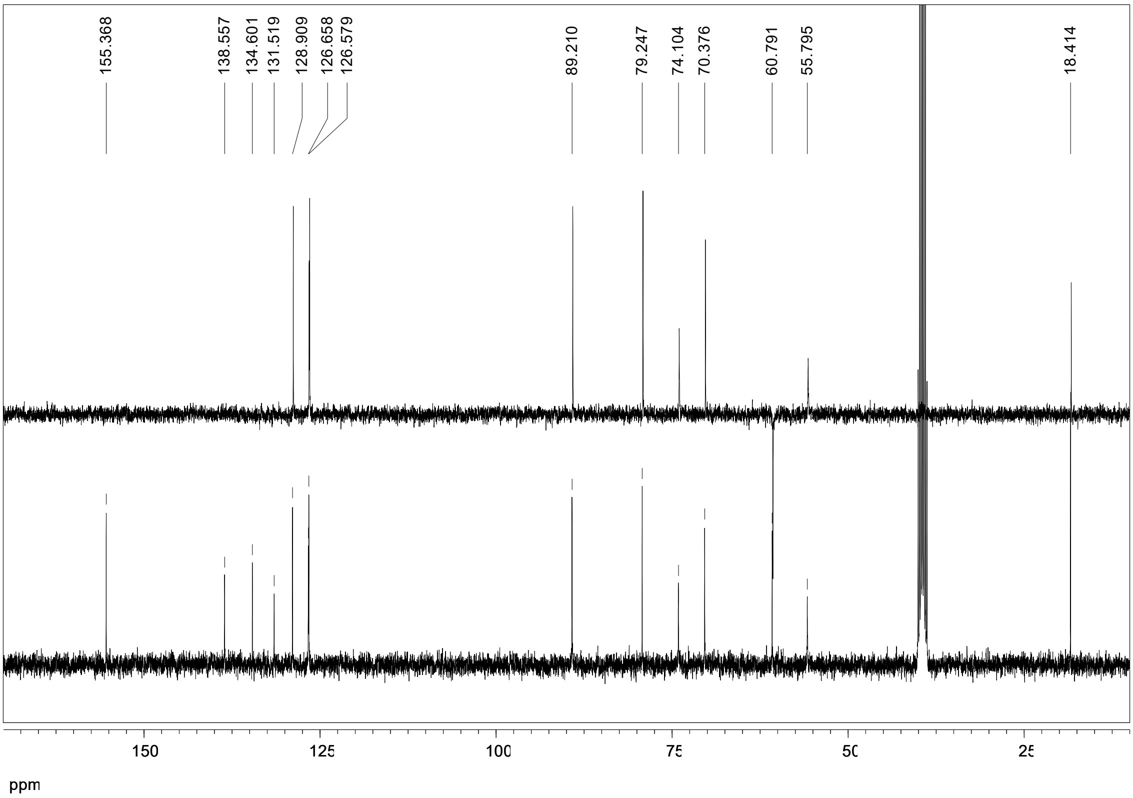

Figure S72 $^{13}C\{^{1}H\}$ NMR and DEPT spectra of 2-deoxy-2-[3-(2-chloro-6-methylphenyl)ureido]-β-D-glucopyranosyl azide (**36**) recorded in DMSO-d_6.

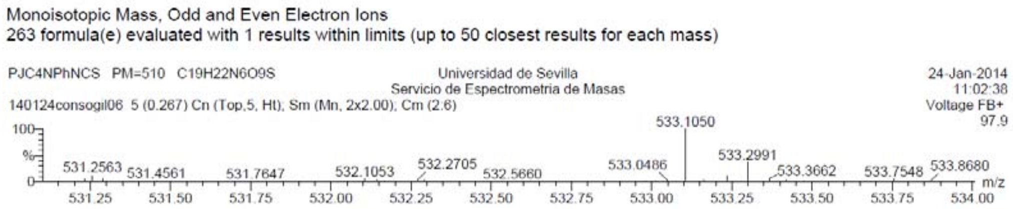

Minimum:	80.00				-100.0		
Maximum:	100.00		200.0	10.0	100.0		
Mass	RA	Calc. Mass	mDa	PPM	DBE	Score	Formula
533.1050	100.00	533.1067	-1.7	-3.1	11.5	1	C19 H22 N6 O9 Na S

Figure S73 Mass spectrum of 3,4,6-tri-*O*-acetyl-2-deoxy-2-[3-(4-nitrophenyl)thioureido]-β-D-glucopyranosyl azide (**21**).

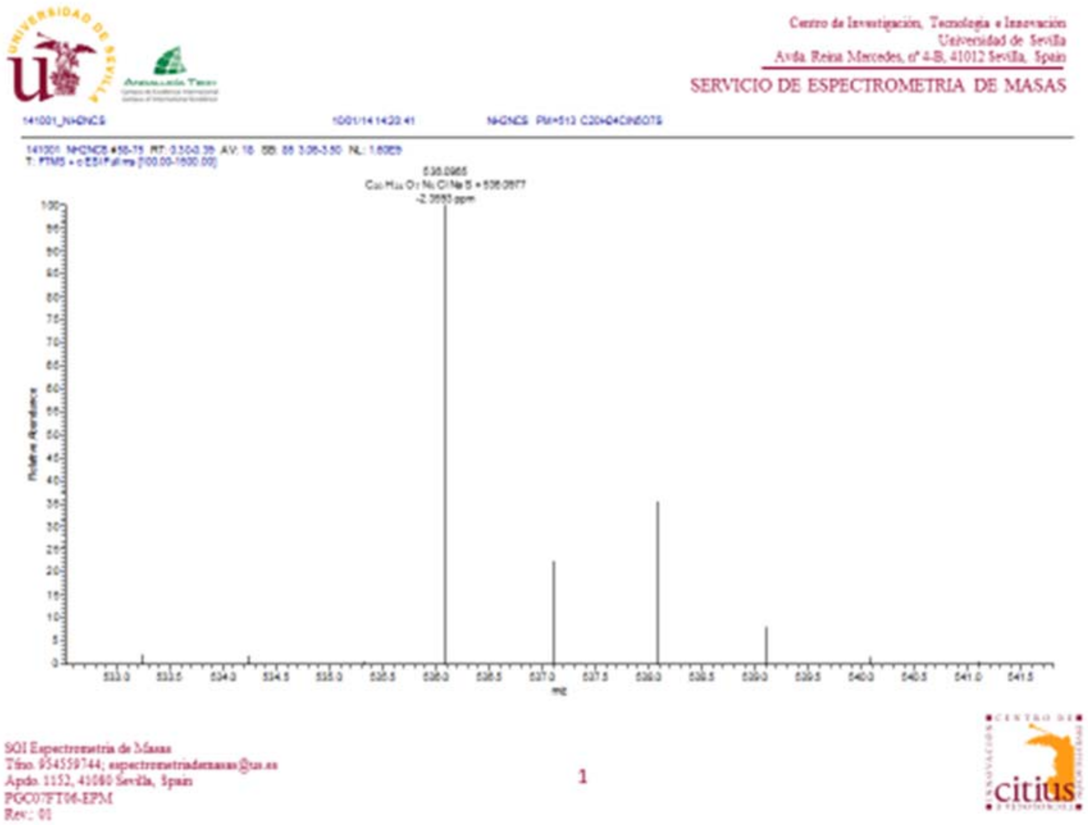

Figure S74 Mass spectrum of 3,4,6-tri-*O*-acetyl-2-deoxy-2-[3-(2-chloro-6-methylphenyl)thioureido]-β-D-glucopyranosyl azide (**25**).

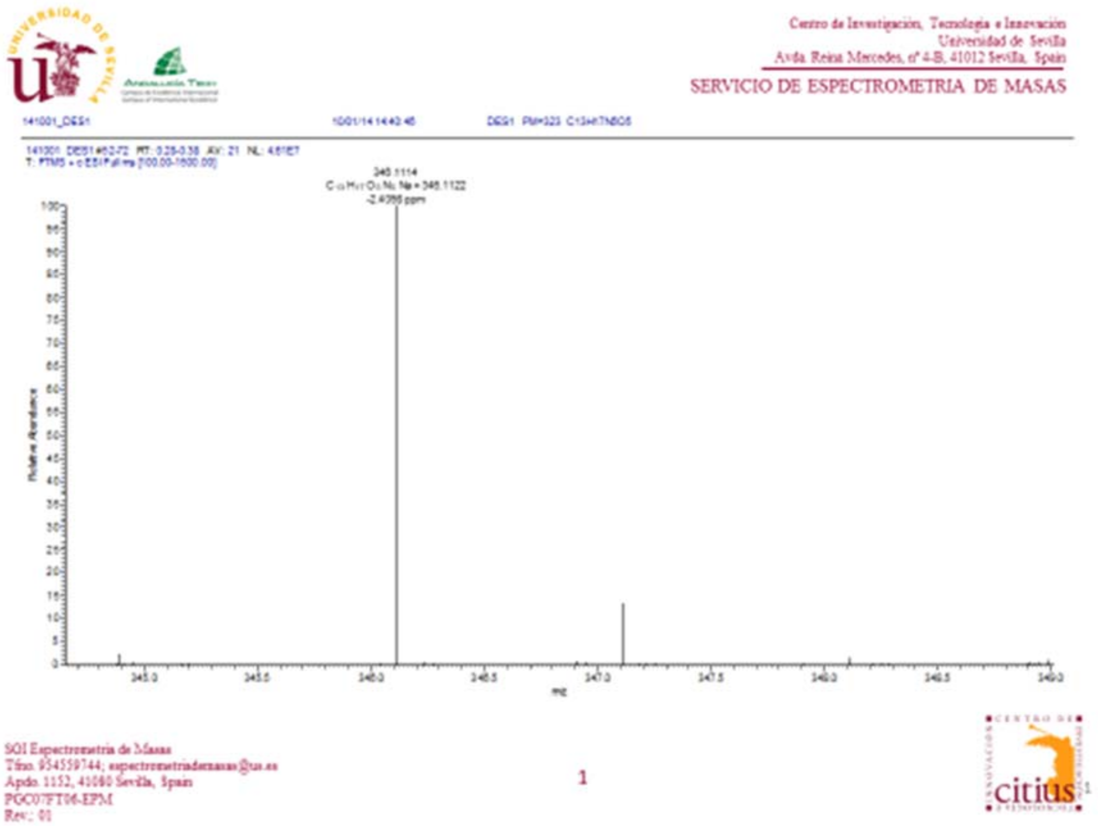

Figure S75 Mass spectrum of 2-deoxy-2-(3-phenylureido)-β-D-glucopyranosyl azide (**34**).

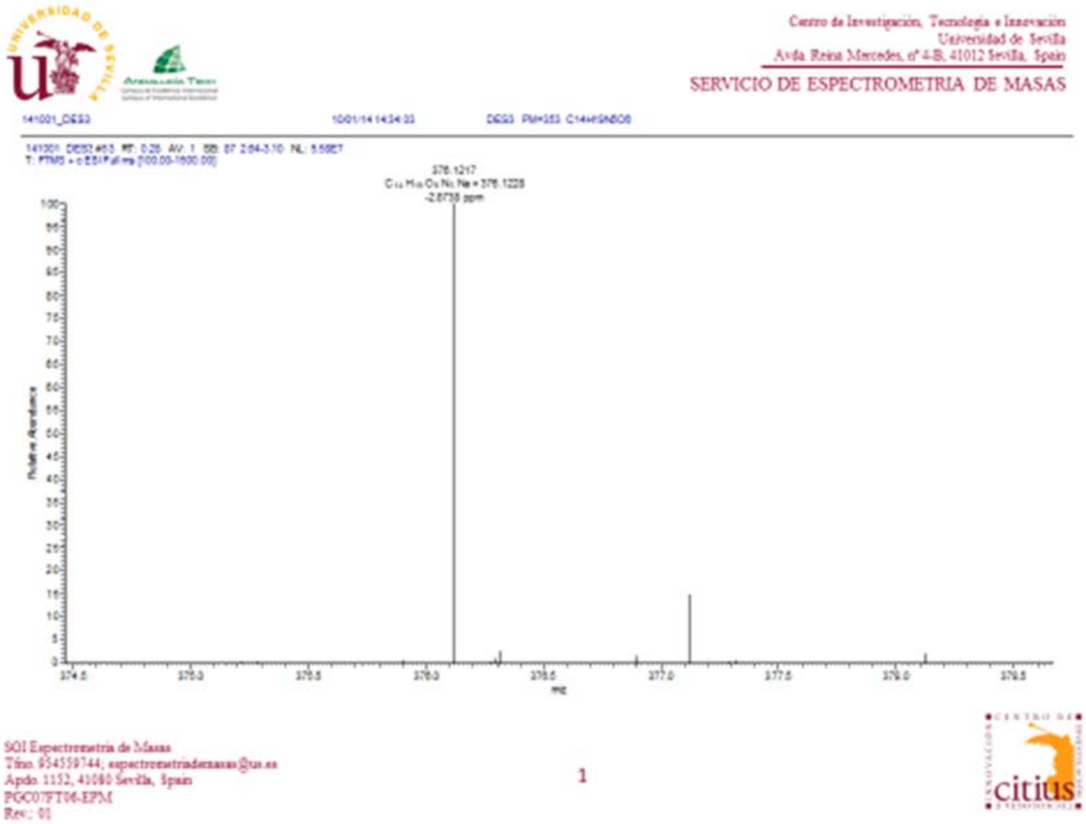

Figure S76 Mass spectrum of 2-deoxy-2-[3-(4-methoxyphenyl)ureido]-β-D-glucopyranosyl azide (**35**).

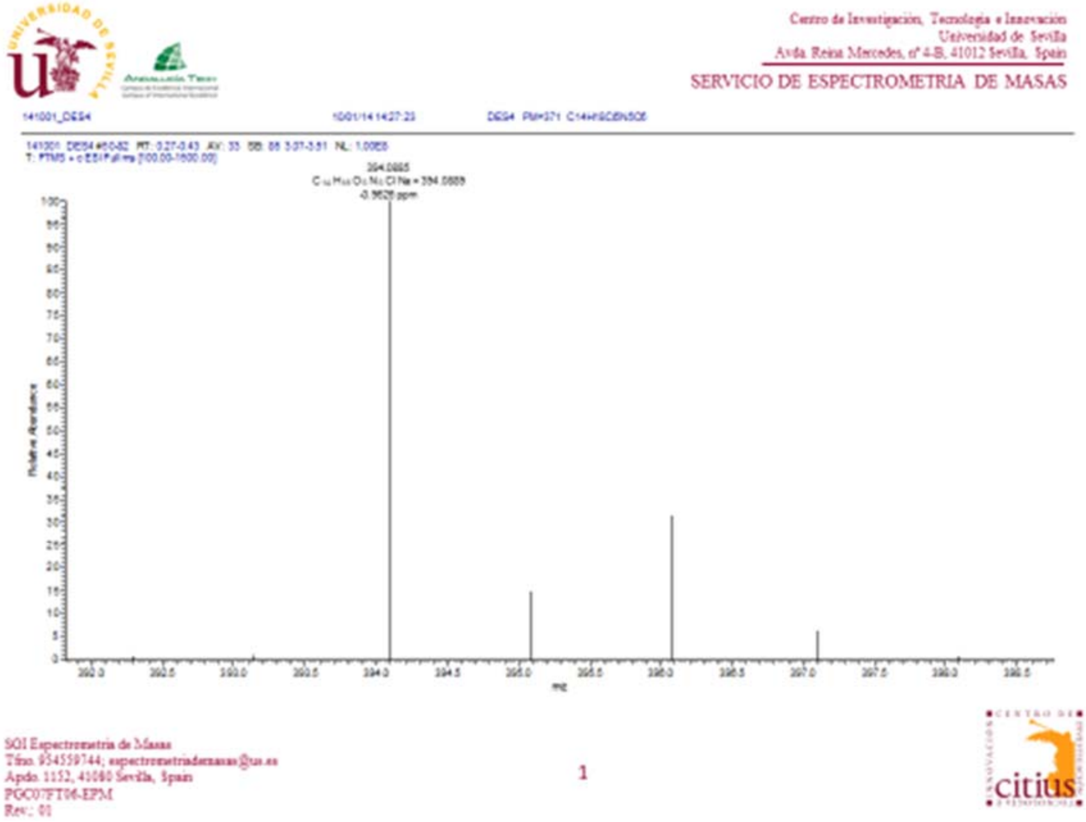

Figure S77 Mass spectrum of 2-deoxy-2-[3-(2-chloro-6-methylphenyl)ureido]-β-D-glucopyranosyl azide (**36**).

Cartesian coordinates and calculated energies at the M06-2X/6-311G(d,p) and M06-2X/def2-TZVP level in gas phase and $CHCl_3$ (SMD Model).

Structure 14 (Z,Z) (M06-2X/6-311G(d,p), gas phase)

Energy (Hartrees): = -1613.2317991
No imaginary frequencies

Standard orientation:

Center Number	Atomic Number	Atomic Type	Coordinates (Angstroms) X	Y	Z
1	6	0	0.698151	-1.244239	-0.802029
2	6	0	-0.004172	-0.032270	-0.159418
3	6	0	0.862919	1.206789	-0.360160
4	6	0	2.285272	0.970296	0.120049
5	6	0	2.842188	-0.300385	-0.517632
6	1	0	0.772015	-1.093151	-1.885934
7	1	0	0.887353	1.484217	-1.420136
8	1	0	2.302970	0.900250	1.210657
9	1	0	2.914145	-0.157038	-1.606628
10	1	0	-0.110200	-0.230701	0.913279
11	8	0	1.986443	-1.386424	-0.233317
12	6	0	4.211238	-0.653311	0.027612
13	1	0	4.148402	-0.851802	1.098931
14	1	0	4.913194	0.155602	-0.172516
15	8	0	4.683624	-1.807404	-0.655392
16	8	0	3.100149	2.060938	-0.310460
17	8	0	0.357754	2.312590	0.391928
18	7	0	-0.031296	-2.481742	-0.640707
19	6	0	4.575238	-2.993924	-0.001851
20	8	0	4.276076	-3.094761	1.149989
21	6	0	4.884666	-4.128101	-0.935776
22	1	0	5.791410	-3.914219	-1.500726
23	1	0	4.987811	-5.047260	-0.365653
24	1	0	4.059983	-4.223701	-1.644694
25	6	0	3.386955	3.037705	0.592586
26	8	0	3.028843	3.015245	1.731450
27	6	0	4.209701	4.114216	-0.056156
28	1	0	3.661551	4.533065	-0.901020
29	1	0	4.429043	4.886766	0.675628
30	1	0	5.134678	3.684688	-0.443039
31	6	0	-0.471354	3.181151	-0.214446
32	8	0	-0.891696	3.030256	-1.332187
33	6	0	-0.792012	4.327592	0.696861
34	1	0	-1.420777	3.960168	1.510182
35	1	0	0.125689	4.714377	1.140022
36	1	0	-1.319466	5.097477	0.140587
37	6	0	-2.421215	-0.333412	-0.082340
38	8	0	-2.328898	-1.053107	0.889135
39	7	0	-0.121279	-2.851700	0.542271
40	7	0	-0.253707	-3.294790	1.558692
41	7	0	-1.314173	0.181287	-0.728828
42	1	0	-1.421015	0.978299	-1.341422
43	7	0	-3.607568	0.060562	-0.660552
44	6	0	-4.913601	-0.287903	-0.269421
45	6	0	-5.206722	-1.017535	0.885860
46	6	0	-5.958022	0.144556	-1.093034
47	6	0	-6.533635	-1.299924	1.190908
48	1	0	-4.406188	-1.359500	1.522382
49	6	0	-7.275077	-0.145603	-0.772623
50	1	0	-5.731480	0.711020	-1.990926
51	6	0	-7.573320	-0.872027	0.374975
52	1	0	-6.750910	-1.867797	2.087988
53	1	0	-8.069003	0.197792	-1.425024
54	1	0	-8.601059	-1.101617	0.627148
55	1	0	-3.543547	0.587067	-1.518163

Structure 14 (Z,E) (M06-2X/6-311G(d,p), gas phase)

Energy (Hartrees): = -1613.2307815
No imaginary frequencies

Standard orientation:

Center Number	Atomic Number	Atomic Type	Coordinates (Angstroms) X	Y	Z
1	6	0	0.822801	-1.412991	-0.372813
2	6	0	-0.084034	-0.463041	0.433664
3	6	0	0.198064	0.969713	-0.007300
4	6	0	1.679694	1.289984	0.096944
5	6	0	2.486311	0.237865	-0.661155
6	1	0	0.568648	-1.346411	-1.438018
7	1	0	-0.126032	1.117538	-1.043603
8	1	0	1.980327	1.333241	1.146842
9	1	0	2.235319	0.294497	-1.731466
10	1	0	0.166381	-0.573722	1.494562
11	8	0	2.174339	-1.046756	-0.164593
12	6	0	3.979592	0.431838	-0.492536
13	1	0	4.257019	0.324791	0.557524
14	1	0	4.275658	1.409775	-0.870607
15	8	0	4.648675	-0.549254	-1.275723
16	8	0	1.919704	2.550302	-0.529593
17	8	0	-0.489217	1.903206	0.825613
18	7	0	0.649949	-2.806763	-0.029407
19	6	0	5.128369	-1.635827	-0.616078
20	8	0	5.182899	-1.722144	0.574026
21	6	0	5.564323	-2.685992	-1.596708
22	1	0	6.139230	-2.235099	-2.404769
23	1	0	6.144688	-3.444679	-1.078845
24	1	0	4.670032	-3.138795	-2.029587
25	6	0	2.040068	3.645313	0.268517
26	8	0	2.006977	3.605454	1.461401
27	6	0	2.228647	4.876845	-0.571004
28	1	0	1.397205	4.974976	-1.269755
29	1	0	2.288120	5.746594	0.077421
30	1	0	3.143674	4.780616	-1.157220
31	6	0	-1.685967	2.365343	0.409158
32	8	0	-2.233879	1.983914	-0.589513
33	6	0	-2.213011	3.393026	1.365953
34	1	0	-2.416320	2.908769	2.322660
35	1	0	-1.454121	4.156267	1.540457
36	1	0	-3.124209	3.827321	0.964166
37	6	0	-2.107922	-1.587754	1.164542
38	8	0	-1.524058	-2.121125	2.085825
39	7	0	1.000935	-3.077051	1.132146
40	7	0	1.299525	-3.445428	2.142988
41	7	0	-1.480688	-0.776754	0.250858
42	1	0	-2.032552	-0.183286	-0.355076
43	7	0	-3.467799	-1.754598	0.986454
44	6	0	-4.293330	-1.218159	-0.027257
45	6	0	-3.964235	-1.374040	-1.375028
46	6	0	-5.471935	-0.558847	0.321656
47	6	0	-4.791079	-0.842534	-2.356050
48	1	0	-3.069900	-1.924881	-1.640961
49	6	0	-6.308712	-0.057278	-0.666662
50	1	0	-5.720309	-0.439326	1.370149
51	6	0	-5.966526	-0.186335	-2.008006
52	1	0	-4.524366	-0.961517	-3.399206
53	1	0	-7.224142	0.449343	-0.385554
54	1	0	-6.613911	0.217360	-2.776375
55	1	0	-3.904117	-2.220775	1.767429

Structure 14 (E,Z) (M06-2X/6-311G(d,p), gas phase)

Energy (Hartrees): = -1613.2257103
No imaginary frequencies

Standard orientation:

Center Number	Atomic Number	Atomic Type	Coordinates (Angstroms) X	Y	Z
1	6	0	-0.152619	0.814638	-1.129150
2	6	0	0.042460	-0.553228	-0.440613
3	6	0	-1.306950	-1.271536	-0.416892
4	6	0	-2.373673	-0.407476	0.233589
5	6	0	-2.416215	0.957331	-0.449875
6	1	0	-0.456809	0.666283	-2.172376
7	1	0	-1.617092	-1.511695	-1.439672
8	1	0	-2.171687	-0.302070	1.302977
9	1	0	-2.739763	0.831141	-1.493763
10	1	0	0.321931	-0.356261	0.601060
11	8	0	-1.128998	1.544098	-0.416538
12	6	0	-3.358905	1.911335	0.255333
13	1	0	-3.025014	2.076536	1.281269
14	1	0	-4.371404	1.509431	0.241375
15	8	0	-3.380765	3.143029	-0.454354
16	8	0	-3.642469	-1.028709	0.041362
17	8	0	-1.207279	-2.475139	0.330206
18	7	0	1.063981	1.603388	-1.192443
19	6	0	-2.614367	4.149654	0.041762
20	8	0	-2.055795	4.102648	1.096716
21	6	0	-2.585074	5.300962	-0.921418
22	1	0	-3.595975	5.543484	-1.247871
23	1	0	-2.115145	6.156628	-0.444505
24	1	0	-2.009890	5.004133	-1.800404
25	6	0	-4.161145	-1.741327	1.079835
26	8	0	-3.649850	-1.808809	2.156662
27	6	0	-5.441303	-2.405125	0.661843
28	1	0	-5.228936	-3.128482	-0.127058
29	1	0	-5.883768	-2.903277	1.519940
30	1	0	-6.127803	-1.663210	0.253190
31	6	0	-1.017691	-3.624834	-0.365501
32	8	0	-0.964163	-3.671031	-1.560871
33	6	0	-0.885570	-4.784678	0.575446
34	1	0	0.087213	-4.718486	1.066837
35	1	0	-1.653570	-4.729024	1.346632
36	1	0	-0.950404	-5.713953	0.016277
37	6	0	2.399427	-1.176805	-1.084062
38	8	0	3.077004	-1.475855	-2.037880
39	7	0	1.464684	1.958053	-0.074887
40	7	0	1.910562	2.324708	0.883732
41	7	0	1.022620	-1.403566	-1.076392
42	1	0	0.727671	-1.835734	-1.944342
43	7	0	2.882816	-0.646161	0.092745
44	6	0	4.204047	-0.237907	0.368240
45	6	0	5.225477	-0.229327	-0.583748
46	6	0	4.468953	0.204145	1.668088
47	6	0	6.490942	0.215010	-0.215320
48	1	0	5.029144	-0.574910	-1.586145
49	6	0	5.736915	0.639602	2.018660
50	1	0	3.668835	0.218790	2.401358
51	6	0	6.760004	0.647522	1.076647
52	1	0	7.278219	0.216767	-0.960001
53	1	0	5.921391	0.978678	3.030913
54	1	0	7.751536	0.987672	1.347496
55	1	0	2.278914	-0.696977	0.897913

Structure 14 (Z,Z) (M06-2X/6-311G(d,p), $CHCl_3$)

Energy (Hartrees): = -1613.2672268
No imaginary frequencies

Standard orientation:

Center Number	Atomic Number	Atomic Type	Coordinates (Angstroms) X	Y	Z
1	6	0	0.525891	-1.353224	-0.761570
2	6	0	-0.185179	-0.133096	-0.149513
3	6	0	0.683127	1.100795	-0.373537
4	6	0	2.091972	0.870567	0.144684
5	6	0	2.672102	-0.413651	-0.450130
6	1	0	0.609675	-1.234645	-1.847152
7	1	0	0.727158	1.346488	-1.439648
8	1	0	2.085307	0.817513	1.235845
9	1	0	2.784113	-0.290440	-1.536675
10	1	0	-0.289644	-0.305811	0.926983
11	8	0	1.801412	-1.497333	-0.171453
12	6	0	4.010917	-0.734771	0.165749
13	1	0	3.882014	-1.032846	1.209306
14	1	0	4.672665	0.133093	0.120653
15	8	0	4.571843	-1.814142	-0.583449
16	8	0	2.902223	1.962122	-0.287525
17	8	0	0.163263	2.221362	0.344984
18	7	0	-0.215015	-2.588936	-0.563374
19	6	0	5.762574	-2.265134	-0.151764
20	8	0	6.340032	-1.778831	0.783782
21	6	0	6.235784	-3.422616	-0.977190
22	1	0	6.278414	-3.131794	-2.028014
23	1	0	7.215987	-3.741917	-0.632085
24	1	0	5.520044	-4.241900	-0.886765
25	6	0	3.331131	2.853914	0.641764
26	8	0	3.099085	2.750978	1.812451
27	6	0	4.121763	3.945315	-0.011582
28	1	0	3.493456	4.460346	-0.740477
29	1	0	4.474301	4.643426	0.743390
30	1	0	4.966746	3.510237	-0.548071
31	6	0	-0.659775	3.072640	-0.299090
32	8	0	-1.023009	2.907260	-1.435136
33	6	0	-1.048901	4.210721	0.590112
34	1	0	-1.648780	3.820752	1.415354
35	1	0	-0.155779	4.669166	1.016681
36	1	0	-1.624350	4.938488	0.023806
37	6	0	-2.609391	-0.418717	-0.066510
38	8	0	-2.530569	-1.106384	0.936658
39	7	0	-0.303189	-2.922191	0.625219
40	7	0	-0.427713	-3.327282	1.659943
41	7	0	-1.498168	0.062097	-0.713966
42	1	0	-1.595671	0.736549	-1.461440
43	7	0	-3.789816	-0.045464	-0.664767
44	6	0	-5.097769	-0.372421	-0.271210
45	6	0	-5.409697	-1.086338	0.890681
46	6	0	-6.134846	0.064366	-1.105262
47	6	0	-6.742556	-1.348484	1.193640
48	1	0	-4.620832	-1.429720	1.540872
49	6	0	-7.457367	-0.204687	-0.787105
50	1	0	-5.892990	0.619104	-2.006414
51	6	0	-7.772777	-0.915723	0.367526
52	1	0	-6.970615	-1.903497	2.096694
53	1	0	-8.243384	0.143409	-1.447400
54	1	0	-8.805249	-1.128351	0.617204
55	1	0	-3.721519	0.497783	-1.514680

Structure 14 (Z,E) (M06-2X/6-311G(d,p), $CHCl_3$)

Energy (Hartrees): = -1613.2653296
No imaginary frequencies

Standard orientation:

Center Number	Atomic Number	Atomic Type	Coordinates (Angstroms) X	Y	Z
1	6	0	-1.044171	-1.421461	0.560416
2	6	0	0.018756	-0.668229	-0.255929
3	6	0	-0.079823	0.816147	0.083922
4	6	0	-1.489905	1.327136	-0.152468
5	6	0	-2.481827	0.456606	0.619271
6	1	0	-0.839795	-1.320649	1.631629
7	1	0	0.187434	0.981702	1.132285
8	1	0	-1.720602	1.324686	-1.219964
9	1	0	-2.294760	0.564046	1.697180
10	1	0	-0.208623	-0.811966	-1.317672
11	8	0	-2.319466	-0.899388	0.240740
12	6	0	-3.915462	0.837352	0.313618
13	1	0	-4.126568	0.700815	-0.747521
14	1	0	-4.100062	1.870488	0.605437
15	8	0	-4.780878	0.018463	1.098456
16	8	0	-1.582075	2.653665	0.366309
17	8	0	0.799763	1.575874	-0.746455
18	7	0	-1.052628	-2.851315	0.301067
19	6	0	-5.353747	-1.045128	0.491151
20	8	0	-5.304513	-1.245249	-0.690319
21	6	0	-6.042716	-1.916043	1.497543
22	1	0	-6.646002	-1.310008	2.173544
23	1	0	-6.658876	-2.650295	0.984464
24	1	0	-5.279783	-2.426267	2.090139
25	6	0	-1.540478	3.689548	-0.509609
26	8	0	-1.502050	3.549034	-1.698786
27	6	0	-1.545898	4.994179	0.226113
28	1	0	-0.614025	5.085967	0.788428
29	1	0	-1.633110	5.812934	-0.483624
30	1	0	-2.369642	5.015589	0.940558
31	6	0	2.013769	1.901802	-0.252645
32	8	0	2.395062	1.559013	0.836397
33	6	0	2.791808	2.712969	-1.239356
34	1	0	3.027968	2.082055	-2.099358
35	1	0	2.186479	3.547347	-1.595889
36	1	0	3.709635	3.066848	-0.776531
37	6	0	1.934788	-2.041307	-0.855640
38	8	0	1.304107	-2.624174	-1.721791
39	7	0	-1.380109	-3.137494	-0.858515
40	7	0	-1.680479	-3.510451	-1.868345
41	7	0	1.356594	-1.144775	0.001827
42	1	0	1.938463	-0.603353	0.628132
43	7	0	3.286727	-2.258803	-0.679294
44	6	0	4.154635	-1.414451	0.068150
45	6	0	4.103746	-1.422350	1.461433
46	6	0	5.056434	-0.579772	-0.591317
47	6	0	4.933191	-0.576207	2.189272
48	1	0	3.416152	-2.095982	1.961148
49	6	0	5.900518	0.245554	0.143093
50	1	0	5.087447	-0.583634	-1.675547
51	6	0	5.833589	0.256799	1.533329
52	1	0	4.883715	-0.578793	3.271726
53	1	0	6.603895	0.888769	-0.372891
54	1	0	6.484267	0.909290	2.103126
55	1	0	3.692812	-2.766472	-1.454481

Structure 14 (E,Z) (M06-2X/6-311G(d,p), $CHCl_3$)

Energy (Hartrees): = -1613.2620349
No imaginary frequencies

Standard orientation:

Center Number	Atomic Number	Atomic Type	Coordinates (Angstroms) X	Y	Z
1	6	0	-0.152426	0.816402	-1.158171
2	6	0	0.045435	-0.552519	-0.475856
3	6	0	-1.305549	-1.265997	-0.448970
4	6	0	-2.356964	-0.398151	0.221579
5	6	0	-2.407343	0.969552	-0.456244
6	1	0	-0.465469	0.677287	-2.198474
7	1	0	-1.624950	-1.496611	-1.469926
8	1	0	-2.133485	-0.290118	1.285752
9	1	0	-2.740145	0.851874	-1.497109
10	1	0	0.338150	-0.368489	0.563101
11	8	0	-1.113380	1.552057	-0.432763
12	6	0	-3.334815	1.920588	0.271266
13	1	0	-3.000413	2.063255	1.299630
14	1	0	-4.354023	1.536171	0.255642
15	8	0	-3.345870	3.167031	-0.421669
16	8	0	-3.629163	-1.019349	0.051220
17	8	0	-1.208064	-2.473946	0.296781
18	7	0	1.072297	1.602681	-1.224785
19	6	0	-2.566157	4.158898	0.065915
20	8	0	-1.997594	4.102294	1.120339
21	6	0	-2.525889	5.311928	-0.890183
22	1	0	-3.536359	5.577662	-1.201974
23	1	0	-2.033812	6.159605	-0.419734
24	1	0	-1.967745	5.007615	-1.778627
25	6	0	-4.156802	-1.696737	1.103511
26	8	0	-3.641820	-1.742191	2.184206
27	6	0	-5.443513	-2.352834	0.709420
28	1	0	-5.247173	-3.084006	-0.077177
29	1	0	-5.882338	-2.842866	1.574793
30	1	0	-6.130505	-1.607036	0.306441
31	6	0	-1.056908	-3.630488	-0.391774
32	8	0	-1.003794	-3.681249	-1.590095
33	6	0	-0.965444	-4.791146	0.547867
34	1	0	-0.019482	-4.725804	1.090248
35	1	0	-1.773775	-4.743035	1.278623
36	1	0	-1.003467	-5.720810	-0.014179
37	6	0	2.393781	-1.203828	-1.093476
38	8	0	3.104814	-1.529845	-2.022217
39	7	0	1.491797	1.947001	-0.113124
40	7	0	1.953137	2.306560	0.840937
41	7	0	1.022511	-1.390901	-1.135114
42	1	0	0.727383	-1.797567	-2.015653
43	7	0	2.854225	-0.663808	0.087318
44	6	0	4.171332	-0.265269	0.380685
45	6	0	5.161778	-0.095500	-0.591182
46	6	0	4.465234	0.020377	1.719259
47	6	0	6.425275	0.344370	-0.208552
48	1	0	4.947021	-0.312760	-1.625925
49	6	0	5.729644	0.458503	2.083322
50	1	0	3.691144	-0.098217	2.471335
51	6	0	6.722114	0.621676	1.120604
52	1	0	7.186026	0.470337	-0.970792
53	1	0	5.937740	0.674234	3.125038
54	1	0	7.710715	0.961225	1.404573
55	1	0	2.239441	-0.720027	0.887223

Structure 14 (Z,Z) (M06-2X/def2-TZVP, gas phase)

Energy (Hartrees): = -1613.4377789
No imaginary frequencies

Standard orientation:

Center Number	Atomic Number	Atomic Type	Coordinates (Angstroms) X	Y	Z
1	6	0	0.648399	-1.180123	-0.829584
2	6	0	-0.075297	0.002986	-0.165119
3	6	0	0.764833	1.261501	-0.348233
4	6	0	2.187319	1.046805	0.130075
5	6	0	2.773908	-0.207092	-0.518892
6	1	0	0.720122	-1.004400	-1.909386
7	1	0	0.782215	1.552171	-1.403249
8	1	0	2.209041	0.949347	1.217296
9	1	0	2.850512	-0.040302	-1.603954
10	1	0	-0.170200	-0.218258	0.902893
11	8	0	1.934666	-1.310820	-0.265758
12	6	0	4.143025	-0.553897	0.028603
13	1	0	4.079867	-0.777059	1.092978
14	1	0	4.839534	0.267348	-0.131937
15	8	0	4.641421	-1.675416	-0.687647
16	8	0	2.930082	2.196483	-0.270242
17	8	0	0.226012	2.341606	0.416134
18	7	0	-0.060473	-2.432333	-0.697231
19	6	0	4.619752	-2.876051	-0.060506
20	8	0	4.334779	-3.022703	1.090489
21	6	0	5.002058	-3.969559	-1.013342
22	1	0	5.897743	-3.692388	-1.566552
23	1	0	5.157617	-4.891483	-0.462269
24	1	0	4.192748	-4.101912	-1.732334
25	6	0	3.846056	2.689658	0.594813
26	8	0	4.109661	2.171488	1.640217
27	6	0	4.467644	3.941243	0.051525
28	1	0	3.694509	4.693169	-0.104461
29	1	0	5.213130	4.305950	0.750663
30	1	0	4.923611	3.732972	-0.916097
31	6	0	-0.624444	3.190525	-0.184326
32	8	0	-1.004208	3.062086	-1.318700
33	6	0	-1.034804	4.288922	0.747441
34	1	0	-1.590428	3.855094	1.579402
35	1	0	-0.151803	4.774949	1.159621
36	1	0	-1.655233	5.002580	0.215456
37	6	0	-2.491289	-0.314404	-0.066730
38	8	0	-2.390006	-1.046744	0.894895
39	7	0	-0.127098	-2.843064	0.470005
40	7	0	-0.230408	-3.313472	1.472408
41	7	0	-1.391827	0.203999	-0.714650
42	1	0	-1.508626	0.978874	-1.350049
43	7	0	-3.681697	0.088758	-0.622712
44	6	0	-4.983678	-0.266369	-0.232530
45	6	0	-5.273515	-0.997187	0.918709
46	6	0	-6.027626	0.162858	-1.053709
47	6	0	-6.597003	-1.284977	1.222940
48	1	0	-4.473875	-1.335674	1.556368
49	6	0	-7.341114	-0.131192	-0.734532
50	1	0	-5.802945	0.730542	-1.949941
51	6	0	-7.636071	-0.860095	0.409370
52	1	0	-6.812331	-1.854519	2.118114
53	1	0	-8.135584	0.210344	-1.385452
54	1	0	-8.661942	-1.093540	0.660878
55	1	0	-3.625317	0.639820	-1.463677

Structure 14 (Z,E) (M06-2X/def2-TZVP, gas phase)

Energy (Hartrees): = -1613.4367736
No imaginary frequencies

Standard orientation:

Center Number	Atomic Number	Atomic Type	Coordinates (Angstroms) X	Y	Z
1	6	0	-0.751406	-1.403239	0.456755
2	6	0	0.166549	-0.501916	-0.386129
3	6	0	-0.118597	0.950728	-0.023958
4	6	0	-1.593465	1.268413	-0.171307
5	6	0	-2.426337	0.252820	0.612025
6	1	0	-0.517021	-1.270523	1.519764
7	1	0	0.187508	1.146768	1.008290
8	1	0	-1.880114	1.256329	-1.224663
9	1	0	-2.208442	0.372616	1.684101
10	1	0	-0.075478	-0.670646	-1.440048
11	8	0	-2.094613	-1.054466	0.202924
12	6	0	-3.914235	0.425437	0.384359
13	1	0	-4.158474	0.267349	-0.665401
14	1	0	-4.236184	1.419430	0.689694
15	8	0	-4.604921	-0.510452	1.201641
16	8	0	-1.784777	2.574070	0.370349
17	8	0	0.594936	1.832020	-0.889468
18	7	0	-0.568764	-2.814015	0.203452
19	6	0	-5.111310	-1.610270	0.594524
20	6	0	-5.629897	-2.579967	1.614942
21	1	0	-6.222272	-2.061119	2.366219
22	1	0	-6.216335	-3.346417	1.118264
23	1	0	-4.777515	-3.039215	2.117288
24	6	0	-2.638332	3.405983	-0.268098
25	8	0	-3.290354	3.080321	-1.217152
26	6	0	-2.646265	4.757028	0.382297
27	1	0	-1.658202	5.207539	0.287643
28	1	0	-3.391240	5.383617	-0.097140
29	1	0	-2.860072	4.652401	1.445339
30	6	0	1.779165	2.313030	-0.465254
31	8	0	2.283109	2.009171	0.581560
32	6	0	2.366310	3.254418	-1.471922
33	1	0	2.538080	2.717014	-2.404627
34	1	0	1.660733	4.057879	-1.680072
35	1	0	3.300440	3.653273	-1.089814
36	6	0	2.204346	-1.651015	-1.036123
37	8	0	1.633283	-2.242420	-1.931312
38	7	0	-0.918973	-3.162791	-0.933419
39	7	0	-1.220809	-3.588782	-1.915109
40	7	0	1.560739	-0.799483	-0.180052
41	1	0	2.098237	-0.192356	0.422020
42	7	0	3.561879	-1.801481	-0.842181
43	6	0	4.396155	-1.106405	0.060087
44	6	0	4.134860	-1.112769	1.428090
45	6	0	5.519081	-0.438430	-0.420402
46	6	0	4.969243	-0.424750	2.295863
47	1	0	3.287286	-1.672714	1.802636
48	6	0	6.364692	0.222480	0.456295
49	1	0	5.720501	-0.439053	-1.484740
50	6	0	6.086771	0.242684	1.815944
51	1	0	4.753513	-0.428515	3.356346
52	1	0	7.238179	0.733676	0.072564
53	1	0	6.740751	0.769883	2.497533
54	1	0	3.997629	-2.288657	-1.609560
55	8	0	-5.132161	-1.769276	-0.589591

Structure 14 (E,Z) (M06-2X/def2-TZVP, gas phase)

Energy (Hartrees): = -1613.4309394
No imaginary frequencies

Standard orientation:

Center Number	Atomic Number	Atomic Type	Coordinates (Angstroms) X	Y	Z
1	6	0	-0.177314	0.726712	-1.144367
2	6	0	0.133540	-0.600760	-0.426191
3	6	0	-1.148141	-1.431389	-0.386501
4	6	0	-2.291598	-0.648816	0.230178
5	6	0	-2.442710	0.698715	-0.476057
6	1	0	-0.466062	0.523563	-2.181763
7	1	0	-1.424210	-1.728310	-1.402516
8	1	0	-2.114389	-0.493923	1.296434
9	1	0	-2.748066	0.520783	-1.517347
10	1	0	0.392646	-0.358194	0.609980
11	8	0	-1.210142	1.387963	-0.455140
12	6	0	-3.462308	1.592085	0.199893
13	1	0	-3.154373	1.813126	1.220992
14	1	0	-4.439854	1.113377	0.207644
15	8	0	-3.588072	2.790487	-0.552752
16	8	0	-3.469233	-1.427844	0.044299
17	8	0	-0.941517	-2.597360	0.396733
18	7	0	0.968277	1.612392	-1.235089
19	6	0	-2.966331	3.894255	-0.072547
20	8	0	-2.424470	3.943926	0.991644
21	6	0	-3.061509	5.019268	-1.059938
22	1	0	-4.086501	5.127517	-1.410097
23	1	0	-2.712292	5.936292	-0.596566
24	1	0	-2.437539	4.779991	-1.921671
25	6	0	-4.352330	-1.492412	1.068290
26	8	0	-4.227493	-0.871373	2.083418
27	6	0	-5.478967	-2.423947	0.737605
28	1	0	-5.082294	-3.421926	0.553466
29	1	0	-6.184496	-2.442708	1.561659
30	1	0	-5.970304	-2.092173	-0.176616
31	6	0	-0.666894	-3.748764	-0.262259
32	8	0	-0.634517	-3.833318	-1.456081
33	6	0	-0.406735	-4.863391	0.703463
34	1	0	0.541240	-4.671049	1.207128
35	1	0	-1.186482	-4.895410	1.462315
36	1	0	-0.350614	-5.802645	0.163070
37	6	0	2.541631	-1.100442	-1.025004
38	8	0	3.255700	-1.458292	-1.933063
39	7	0	1.338520	2.039191	-0.137422
40	7	0	1.747474	2.471907	0.805143
41	7	0	1.179720	-1.373320	-1.044694
42	1	0	0.924568	-1.877844	-1.883942
43	7	0	2.981884	-0.461980	0.108506
44	6	0	4.282771	-0.002734	0.386032
45	6	0	5.323911	-0.023602	-0.539853
46	6	0	4.506197	0.519888	1.660808
47	6	0	6.566677	0.473921	-0.171723
48	1	0	5.160463	-0.428741	-1.524422
49	6	0	5.751053	1.009846	2.011527
50	1	0	3.691855	0.550974	2.376146
51	6	0	6.793594	0.989180	1.095054
52	1	0	7.369883	0.452925	-0.897103
53	1	0	5.903478	1.411815	3.004645
54	1	0	7.768418	1.371385	1.365946
55	1	0	2.344523	-0.405342	0.884576

Structure 14 (Z,Z) (M06-2X/def2-TZVP, $CHCl_3$)

Energy (Hartrees): = -1613.4730947
No imaginary frequencies

Standard orientation:

Center Number	Atomic Number	Atomic Type	Coordinates (Angstroms) X	Y	Z
1	6	0	0.648157	-1.188201	-0.830843
2	6	0	-0.075898	-0.007001	-0.167575
3	6	0	0.759869	1.254154	-0.359247
4	6	0	2.179741	1.039112	0.128210
5	6	0	2.773439	-0.206332	-0.531355
6	1	0	0.704378	-1.030911	-1.912685
7	1	0	0.780421	1.535057	-1.416054
8	1	0	2.190816	0.928807	1.214100
9	1	0	2.844703	-0.036190	-1.614991
10	1	0	-0.160024	-0.219820	0.902339
11	8	0	1.937825	-1.319143	-0.278279
12	6	0	4.142921	-0.543554	0.017208
13	1	0	4.085504	-0.764306	1.081954
14	1	0	4.834965	0.280366	-0.151479
15	8	0	4.654534	-1.665152	-0.697794
16	8	0	2.925498	2.191713	-0.256363
17	8	0	0.211241	2.328399	0.401791
18	7	0	-0.057113	-2.446981	-0.661039
19	6	0	4.657239	-2.864830	-0.083725
20	8	0	4.365489	-3.025180	1.069444
21	6	0	5.070256	-3.944546	-1.034170
22	1	0	5.978647	-3.655990	-1.561846
23	1	0	5.221985	-4.873644	-0.492733
24	1	0	4.280579	-4.076647	-1.775930
25	6	0	3.811680	2.705769	0.623507
26	8	0	4.043986	2.204023	1.688760
27	6	0	4.443404	3.947150	0.080749
28	1	0	3.670906	4.690679	-0.118353
29	1	0	5.161986	4.335470	0.796283
30	1	0	4.937783	3.720819	-0.864684
31	6	0	-0.616087	3.194070	-0.210586
32	8	0	-0.947447	3.087136	-1.363500
33	6	0	-1.055724	4.271680	0.725881
34	1	0	-1.609851	3.820226	1.550132
35	1	0	-0.183540	4.771194	1.147733
36	1	0	-1.683938	4.983339	0.198884
37	6	0	-2.497984	-0.318428	-0.057915
38	8	0	-2.402965	-1.029107	0.928577
39	7	0	-0.114894	-2.824607	0.513545
40	7	0	-0.206795	-3.261678	1.533128
41	7	0	-1.398855	0.184856	-0.701842
42	1	0	-1.514225	0.857652	-1.446149
43	7	0	-3.685526	0.056198	-0.632565
44	6	0	-4.986813	-0.288356	-0.239028
45	6	0	-5.289204	-1.008180	0.917465
46	6	0	-6.027251	0.140394	-1.068108
47	6	0	-6.616422	-1.285613	1.220428
48	1	0	-4.498441	-1.345198	1.566869
49	6	0	-7.343883	-0.142959	-0.750835
50	1	0	-5.792077	0.700914	-1.966049
51	6	0	-7.649822	-0.861282	0.398743
52	1	0	-6.837764	-1.845898	2.120640
53	1	0	-8.133667	0.199338	-1.407983
54	1	0	-8.678692	-1.085845	0.648342
55	1	0	-3.629152	0.616290	-1.470827

Structure 14 (Z,E) (M06-2X/def2-TZVP, $CHCl_3$)

Energy (Hartrees): = -1613.4713098
No imaginary frequencies

Standard orientation:

Center Number	Atomic Number	Atomic Type	Coordinates (Angstroms) X	Y	Z
1	6	0	-0.752275	-1.426560	0.431772
2	6	0	0.168621	-0.525698	-0.405176
3	6	0	-0.108788	0.926272	-0.032443
4	6	0	-1.583032	1.248770	-0.175165
5	6	0	-2.414417	0.237920	0.615465
6	1	0	-0.508533	-1.321485	1.493966
7	1	0	0.199715	1.112021	1.000164
8	1	0	-1.872511	1.233916	-1.227260
9	1	0	-2.183216	0.347210	1.684499
10	1	0	-0.072336	-0.680082	-1.461138
11	8	0	-2.095289	-1.072874	0.191489
12	6	0	-3.899290	0.431381	0.398245
13	1	0	-4.156472	0.275580	-0.648456
14	1	0	-4.202259	1.430710	0.708220
15	8	0	-4.607878	-0.487553	1.226264
16	8	0	-1.771939	2.552521	0.370000
17	8	0	0.605231	1.804618	-0.899602
18	7	0	-0.586905	-2.839624	0.137598
19	6	0	-5.161120	-1.570142	0.644868
20	6	0	-5.750838	-2.480975	1.675660
21	1	0	-6.420602	-1.920286	2.327363
22	1	0	-6.284717	-3.290732	1.187202
23	1	0	-4.946450	-2.885904	2.291640
24	6	0	-2.565490	3.420848	-0.292791
25	8	0	-3.167399	3.130903	-1.289878
26	6	0	-2.576466	4.753807	0.384246
27	1	0	-1.582942	5.198619	0.306563
28	1	0	-3.308592	5.400282	-0.090126
29	1	0	-2.803832	4.630145	1.442887
30	6	0	1.778521	2.309671	-0.472973
31	8	0	2.274297	2.020735	0.585029
32	6	0	2.354970	3.253243	-1.476997
33	1	0	2.492287	2.731835	-2.424956
34	1	0	1.655269	4.072261	-1.647374
35	1	0	3.304838	3.636423	-1.116462
36	6	0	2.225771	-1.663482	-1.045526
37	8	0	1.664573	-2.256292	-1.955786
38	7	0	-0.937179	-3.148321	-1.005919
39	7	0	-1.244032	-3.532481	-2.004516
40	7	0	1.561768	-0.829544	-0.196087
41	1	0	2.082658	-0.251761	0.448174
42	7	0	3.575832	-1.811307	-0.836127
43	6	0	4.394544	-1.118126	0.085318
44	6	0	4.123217	-1.146908	1.451301
45	6	0	5.511757	-0.430387	-0.381296
46	6	0	4.945320	-0.461177	2.334287
47	1	0	3.280289	-1.720888	1.816529
48	6	0	6.342802	0.230705	0.510045
49	1	0	5.720885	-0.418423	-1.444402
50	6	0	6.056679	0.228093	1.869281
51	1	0	4.723483	-0.483044	3.393824
52	1	0	7.211854	0.759246	0.138511
53	1	0	6.700512	0.754034	2.562338
54	1	0	4.037516	-2.268789	-1.609351
55	8	0	-5.176115	-1.756491	-0.540731

Structure 14 (E,Z) (M06-2X/def2-TZVP, $CHCl_3$)

Energy (Hartrees): = -1613.4676889
No imaginary frequencies

Standard orientation:

Center Number	Atomic Number	Atomic Type	Coordinates (Angstroms) X	Y	Z
1	6	0	-0.179060	0.741656	-1.154585
2	6	0	0.136570	-0.585864	-0.441477
3	6	0	-1.143827	-1.418356	-0.413535
4	6	0	-2.281250	-0.637833	0.217893
5	6	0	-2.440206	0.711091	-0.484365
6	1	0	-0.470675	0.544067	-2.190579
7	1	0	-1.420543	-1.699716	-1.432955
8	1	0	-2.087910	-0.486277	1.281413
9	1	0	-2.742176	0.536953	-1.526178
10	1	0	0.401359	-0.357411	0.595528
11	8	0	-1.206220	1.405237	-0.458854
12	6	0	-3.463144	1.591551	0.200466
13	1	0	-3.168615	1.796142	1.228409
14	1	0	-4.441810	1.114453	0.185371
15	8	0	-3.583458	2.806505	-0.533904
16	8	0	-3.462578	-1.413836	0.041376
17	8	0	-0.939861	-2.592502	0.361267
18	7	0	0.970385	1.630198	-1.243521
19	6	0	-2.987652	3.908740	-0.036568
20	8	0	-2.469338	3.955390	1.045367
21	6	0	-3.062874	5.039581	-1.013665
22	1	0	-4.077789	5.143010	-1.395360
23	1	0	-2.737445	5.959290	-0.536878
24	1	0	-2.408764	4.811875	-1.857352
25	6	0	-4.324602	-1.515995	1.076809
26	8	0	-4.172811	-0.932171	2.114594
27	6	0	-5.453690	-2.436251	0.742371
28	1	0	-5.058915	-3.439059	0.573785
29	1	0	-6.169462	-2.448802	1.558649
30	1	0	-5.934508	-2.109429	-0.179697
31	6	0	-0.687421	-3.747604	-0.293059
32	8	0	-0.649949	-3.829846	-1.490715
33	6	0	-0.459363	-4.868159	0.667994
34	1	0	0.472418	-4.682952	1.205288
35	1	0	-1.265456	-4.904209	1.400080
36	1	0	-0.389914	-5.806307	0.125940
37	6	0	2.539700	-1.108581	-1.016381
38	8	0	3.283838	-1.505555	-1.894160
39	7	0	1.356660	2.042611	-0.148035
40	7	0	1.779476	2.465537	0.792687
41	7	0	1.183905	-1.343495	-1.080931
42	1	0	0.925514	-1.835255	-1.927241
43	7	0	2.965120	-0.445714	0.106630
44	6	0	4.269700	-0.006176	0.390135
45	6	0	5.260810	0.138262	-0.579996
46	6	0	4.546470	0.346720	1.712658
47	6	0	6.510413	0.619074	-0.211149
48	1	0	5.056834	-0.124578	-1.605163
49	6	0	5.796095	0.827488	2.063871
50	1	0	3.770953	0.244648	2.463982
51	6	0	6.790311	0.964358	1.103059
52	1	0	7.273399	0.725480	-0.972376
53	1	0	5.991624	1.095574	3.094492
54	1	0	7.768829	1.336656	1.376613
55	1	0	2.325364	-0.386106	0.883791

Structure 15 (Z,Z) (M06-2X/6-311G(d,p), gas phase)

Energy (Hartrees): = -1936.1742609
No imaginary frequencies

Standard orientation:

Center Number	Atomic Number	Atomic Type	Coordinates (Angstroms) X	Y	Z
1	6	0	0.934271	-1.306328	-0.791247
2	6	0	0.115006	-0.155618	-0.175967
3	6	0	0.843370	1.165366	-0.423049
4	6	0	2.278981	1.096807	0.067238
5	6	0	2.965537	-0.120275	-0.548951
6	1	0	0.982806	-1.186854	-1.881108
7	1	0	0.848341	1.407804	-1.492145
8	1	0	2.300661	1.048186	1.158873
9	1	0	3.012217	0.006197	-1.641333
10	1	0	0.033121	-0.327806	0.901648
11	8	0	2.233580	-1.286881	-0.234313
12	6	0	4.369469	-0.313889	-0.013062
13	1	0	4.340273	-0.494593	1.062656
14	1	0	4.978326	0.560591	-0.240329
15	8	0	4.953714	-1.426324	-0.679263
16	8	0	2.973653	2.258835	-0.383550
17	8	0	0.202814	2.225840	0.285068
18	7	0	0.341089	-2.607723	-0.577999
19	6	0	4.990115	-2.600226	0.004041
20	8	0	4.720648	-2.704563	1.162897
21	6	0	5.408443	-3.717853	-0.907200
22	1	0	6.250091	-3.408918	-1.525940
23	1	0	5.660479	-4.591977	-0.312811
24	1	0	4.570627	-3.951832	-1.567170
25	6	0	3.153774	3.274940	0.504905
26	8	0	2.805625	3.227092	1.645632
27	6	0	3.848580	4.427234	-0.162969
28	1	0	3.248724	4.775085	-1.004877
29	1	0	3.990840	5.226139	0.559417
30	1	0	4.810194	4.095642	-0.556690
31	6	0	-0.747782	2.937158	-0.351305
32	8	0	-1.096569	2.718946	-1.482217
33	6	0	-1.303091	3.999344	0.548392
34	1	0	-1.926203	3.514090	1.302977
35	1	0	-0.493746	4.512094	1.067427
36	1	0	-1.904190	4.691039	-0.035386
37	6	0	-2.350484	-0.438477	-0.058887
38	7	0	0.421401	-3.000148	0.597729
39	7	0	0.452348	-3.468491	1.611611
40	7	0	-1.215351	-0.082406	-0.731151
41	1	0	-1.324102	0.567696	-1.500394
42	7	0	-3.473140	-0.119603	-0.768147
43	6	0	-4.827374	-0.352328	-0.416398
44	6	0	-5.331763	-0.001929	0.833829
45	6	0	-5.674791	-0.883106	-1.386331
46	6	0	-6.677812	-0.202688	1.107436
47	1	0	-4.670089	0.414560	1.579504
48	6	0	-7.023714	-1.065317	-1.108517
49	1	0	-5.270739	-1.164083	-2.352881
50	6	0	-7.528617	-0.731556	0.141942
51	1	0	-7.064680	0.064400	2.083328
52	1	0	-7.675588	-1.479024	-1.868258
53	1	0	-8.578028	-0.881964	0.363227
54	1	0	-3.332454	0.139437	-1.735077
55	16	0	-2.341984	-1.172401	1.438350

Structure 15 (Z,E) (M06-2X/6-311G(d,p), gas phase)

Energy (Hartrees): = -1936.1805349
No imaginary frequencies

Standard orientation:

Center Number	Atomic Number	Atomic Type	Coordinates (Angstroms) X	Y	Z
1	6	0	-0.447062	-1.273356	0.230340
2	6	0	0.162445	-0.067159	-0.510509
3	6	0	-0.388016	1.214410	0.109093
4	6	0	-1.906767	1.199243	0.095486
5	6	0	-2.416878	-0.080067	0.757761
6	1	0	-0.142276	-1.253416	1.285235
7	1	0	-0.040884	1.321912	1.143036
8	1	0	-2.271812	1.279916	-0.931702
9	1	0	-2.133789	-0.073868	1.821072
10	1	0	-0.132200	-0.116808	-1.562523
11	8	0	-1.852620	-1.210581	0.121459
12	6	0	-3.922098	-0.209011	0.642380
13	1	0	-4.212851	-0.265641	-0.407925
14	1	0	-4.405705	0.637038	1.129394
15	8	0	-4.333745	-1.385942	1.326966
16	8	0	-2.381397	2.301335	0.867052
17	8	0	0.035998	2.347319	-0.639822
18	7	0	0.024074	-2.546528	-0.274619
19	6	0	-4.588279	-2.480612	0.563885
20	8	0	-4.636161	-2.464610	-0.629425
21	6	0	-4.791158	-3.685241	1.437215
22	1	0	-5.425614	-3.435725	2.286781
23	1	0	-5.224745	-4.488356	0.847516
24	1	0	-3.817953	-3.996907	1.821864
25	6	0	-2.808323	3.406613	0.195447
26	8	0	-2.872888	3.475202	-0.994328
27	6	0	-3.177759	4.489730	1.168091
28	1	0	-2.294394	4.774796	1.741517
29	1	0	-3.565495	5.345043	0.621822
30	1	0	-3.921941	4.115761	1.871990
31	6	0	1.168880	2.970508	-0.246516
32	8	0	1.810918	2.636506	0.711428
33	6	0	1.495309	4.097974	-1.179190
34	1	0	1.865939	3.669424	-2.113083
35	1	0	0.595684	4.667438	-1.410300
36	1	0	2.260737	4.727409	-0.733626
37	6	0	2.408774	-0.523623	-1.437602
38	7	0	-0.450490	-2.840872	-1.386045
39	7	0	-0.828515	-3.221067	-2.365378
40	7	0	1.600162	-0.082989	-0.444806
41	1	0	2.033009	0.271507	0.400232
42	7	0	3.722660	-0.629767	-1.092253
43	6	0	4.238492	-0.754285	0.228188
44	6	0	3.655967	-1.643650	1.134130
45	6	0	5.356235	-0.011111	0.595945
46	6	0	4.190994	-1.769346	2.409255
47	1	0	2.798910	-2.233315	0.824289
48	6	0	5.897768	-0.160592	1.867581
49	1	0	5.786109	0.683697	-0.115910
50	6	0	5.313266	-1.032596	2.778204
51	1	0	3.741004	-2.460722	3.111640
52	1	0	6.770423	0.416244	2.148992
53	1	0	5.731501	-1.141569	3.771330
54	1	0	4.330117	-0.860618	-1.864632
55	16	0	1.899338	-0.878468	-2.992939

Structure 15 (E,Z) (M06-2X/6-311G(d,p), gas phase)

Energy (Hartrees): = -1936.1716117
No imaginary frequencies

Standard orientation:

Center Number	Atomic Number	Atomic Type	Coordinates (Angstroms) X	Y	Z
1	6	0	-0.144503	0.800149	-0.992003
2	6	0	-0.048307	-0.573089	-0.288478
3	6	0	-1.424495	-1.234124	-0.332001
4	6	0	-2.488581	-0.321166	0.253761
5	6	0	-2.439179	1.033399	-0.449159
6	1	0	-0.386083	0.657911	-2.051899
7	1	0	-1.690993	-1.469271	-1.367984
8	1	0	-2.338688	-0.208133	1.330571
9	1	0	-2.702613	0.903509	-1.509106
10	1	0	0.197329	-0.388276	0.762606
11	8	0	-1.134344	1.570459	-0.343717
12	6	0	-3.385198	2.034639	0.182814
13	1	0	-3.110617	2.203110	1.225318
14	1	0	-4.410246	1.673405	0.109651
15	8	0	-3.310810	3.253751	-0.544461
16	8	0	-3.767836	-0.896809	0.001315
17	8	0	-1.416315	-2.435469	0.426891
18	7	0	1.101339	1.545878	-0.978651
19	6	0	-2.538572	4.237329	-0.012382
20	8	0	-2.058311	4.187739	1.080464
21	6	0	-2.387921	5.364869	-0.992180
22	1	0	-3.354502	5.621282	-1.424329
23	1	0	-1.945438	6.221047	-0.490540
24	1	0	-1.734555	5.032578	-1.801188
25	6	0	-4.371871	-1.568700	1.020691
26	8	0	-3.924442	-1.635548	2.125700
27	6	0	-5.652467	-2.189193	0.541669
28	1	0	-5.426218	-2.928025	-0.228735
29	1	0	-6.157891	-2.661022	1.379571
30	1	0	-6.287401	-1.426167	0.090809
31	6	0	-1.257369	-3.599594	-0.253220
32	8	0	-1.131202	-3.654477	-1.442684
33	6	0	-1.258721	-4.761448	0.694403
34	1	0	-0.320524	-4.752907	1.252492
35	1	0	-2.073975	-4.659015	1.410264
36	1	0	-1.339706	-5.686426	0.130315
37	6	0	2.282382	-1.251663	-0.873467
38	7	0	1.432686	1.903555	0.161878
39	7	0	1.826286	2.270418	1.142642
40	7	0	0.921646	-1.464232	-0.896458
41	1	0	0.617818	-1.923566	-1.747167
42	7	0	2.728682	-0.750257	0.316083
43	6	0	4.015280	-0.217015	0.577406
44	6	0	4.566708	0.739873	-0.272810
45	6	0	4.685307	-0.597773	1.734854
46	6	0	5.797712	1.297579	0.037459
47	1	0	4.026794	1.032511	-1.164932
48	6	0	5.912139	-0.021592	2.044911
49	1	0	4.249518	-1.349188	2.383968
50	6	0	6.474444	0.922045	1.195279
51	1	0	6.227948	2.037779	-0.626046
52	1	0	6.430021	-0.320236	2.948134
53	1	0	7.433338	1.365535	1.433016
54	1	0	2.161103	-0.943410	1.129214
55	16	0	3.236655	-1.640929	-2.170363

Structure 15 (Z,Z) (M06-2X/6-311G(d,p), $CHCl_3$)

Energy (Hartrees): = -1936.2129962
No imaginary frequencies

Standard orientation:

Center Number	Atomic Number	Atomic Type	Coordinates (Angstroms) X	Y	Z
1	6	0	0.798164	-1.242266	-0.775009
2	6	0	0.091993	-0.035758	-0.127074
3	6	0	0.922274	1.214408	-0.412555
4	6	0	2.354996	1.024218	0.056343
5	6	0	2.934855	-0.243531	-0.569191
6	1	0	0.821230	-1.120279	-1.863774
7	1	0	0.926357	1.430502	-1.486333
8	1	0	2.392592	0.968570	1.146675
9	1	0	2.985265	-0.121567	-1.660691
10	1	0	0.039239	-0.199896	0.952372
11	8	0	2.103575	-1.347364	-0.252057
12	6	0	4.314104	-0.558269	-0.027931
13	1	0	4.271744	-0.709056	1.051412
14	1	0	5.005373	0.248009	-0.270600
15	8	0	4.799515	-1.734335	-0.672897
16	8	0	3.127301	2.130712	-0.406190
17	8	0	0.393450	2.338142	0.287710
18	7	0	0.102061	-2.496577	-0.538222
19	6	0	4.687613	-2.904313	-0.002701
20	8	0	4.345175	-2.986239	1.143319
21	6	0	5.051808	-4.054572	-0.891483
22	1	0	6.003129	-3.861128	-1.388166
23	1	0	5.105025	-4.967410	-0.303158
24	1	0	4.284190	-4.156765	-1.661920
25	6	0	3.427405	3.116625	0.479158
26	8	0	3.143014	3.069016	1.641485
27	6	0	4.145448	4.234243	-0.212513
28	1	0	3.467672	4.697710	-0.932657
29	1	0	4.470051	4.968620	0.520332
30	1	0	5.000343	3.840447	-0.763763
31	6	0	-0.465570	3.148532	-0.365413
32	8	0	-0.828044	2.950953	-1.496065
33	6	0	-0.888916	4.285825	0.508626
34	1	0	-1.459946	3.885804	1.349436
35	1	0	-0.009422	4.787467	0.914790
36	1	0	-1.501411	4.978630	-0.062709
37	6	0	-2.385230	-0.261520	-0.017970
38	7	0	0.226367	-2.910741	0.621967
39	7	0	0.290622	-3.384480	1.632520
40	7	0	-1.246701	0.135996	-0.637021
41	1	0	-1.334835	0.677329	-1.490348
42	7	0	-3.491536	-0.036498	-0.773191
43	6	0	-4.846265	-0.290098	-0.433875
44	6	0	-5.408489	0.207094	0.740143
45	6	0	-5.633340	-0.991092	-1.344598
46	6	0	-6.753148	-0.019046	1.004160
47	1	0	-4.795139	0.765180	1.434339
48	6	0	-6.982413	-1.199606	-1.079397
49	1	0	-5.183097	-1.376205	-2.253262
50	6	0	-7.544817	-0.719799	0.098136
51	1	0	-7.185778	0.365479	1.920420
52	1	0	-7.589162	-1.746233	-1.791645
53	1	0	-8.594036	-0.888872	0.309189
54	1	0	-3.334184	0.179256	-1.750805
55	16	0	-2.409101	-0.964564	1.513433

Structure 15 (Z,E) (M06-2X/6-311G(d,p), $CHCl_3$)

Energy (Hartrees): = -1936.217831
No imaginary frequencies

Standard orientation:

Center Number	Atomic Number	Atomic Type	Coordinates (Angstroms) X	Y	Z
1	6	0	0.650676	-1.331272	-0.340030
2	6	0	-0.121507	-0.288747	0.490696
3	6	0	0.254075	1.104436	-0.010195
4	6	0	1.761210	1.291538	0.024245
5	6	0	2.438065	0.160557	-0.749323
6	1	0	0.339107	-1.277095	-1.389427
7	1	0	-0.101013	1.249569	-1.035515
8	1	0	2.114680	1.318949	1.057364
9	1	0	2.147163	0.218539	-1.807666
10	1	0	0.169331	-0.386438	1.539536
11	8	0	2.034026	-1.089016	-0.213583
12	6	0	3.945969	0.231140	-0.634391
13	1	0	4.252670	0.127859	0.406920
14	1	0	4.307570	1.174013	-1.043326
15	8	0	4.514188	-0.812380	-1.423967
16	8	0	2.075380	2.516523	-0.635682
17	8	0	-0.319378	2.102738	0.830071
18	7	0	0.373990	-2.696950	0.076737
19	6	0	4.928949	-1.927814	-0.781495
20	8	0	4.972653	-2.032502	0.412237
21	6	0	5.317663	-2.986478	-1.768836
22	1	0	5.973670	-2.567165	-2.532135
23	1	0	5.806955	-3.807716	-1.251063
24	1	0	4.413588	-3.348881	-2.263335
25	6	0	2.423221	3.586191	0.125212
26	8	0	2.534987	3.540437	1.316859
27	6	0	2.648554	4.788637	-0.739159
28	1	0	1.745071	4.999270	-1.313718
29	1	0	2.907720	5.640751	-0.116005
30	1	0	3.453609	4.581134	-1.446318
31	6	0	-1.500520	2.642796	0.458015
32	8	0	-2.088485	2.317579	-0.539695
33	6	0	-1.954038	3.661349	1.455109
34	1	0	-2.225830	3.143190	2.377920
35	1	0	-1.139252	4.347853	1.686574
36	1	0	-2.815896	4.196563	1.064641
37	6	0	-2.324489	-1.084579	1.304096
38	7	0	0.902415	-2.995334	1.156551
39	7	0	1.348187	-3.376259	2.108160
40	7	0	-1.547469	-0.482506	0.382745
41	1	0	-1.998634	-0.078867	-0.431731
42	7	0	-3.627265	-1.236804	0.968740
43	6	0	-4.228887	-0.954576	-0.290583
44	6	0	-3.739654	-1.548113	-1.455092
45	6	0	-5.332134	-0.106323	-0.342708
46	6	0	-4.349383	-1.272013	-2.673100
47	1	0	-2.897832	-2.229582	-1.394824
48	6	0	-5.948576	0.146894	-1.562787
49	1	0	-5.695826	0.349234	0.571142
50	6	0	-5.453999	-0.426652	-2.729762
51	1	0	-3.968943	-1.732089	-3.577360
52	1	0	-6.809386	0.803749	-1.601260
53	1	0	-5.931268	-0.220493	-3.680250
54	1	0	-4.224123	-1.566754	1.714880
55	16	0	-1.765642	-1.618093	2.810314

Structure 15 (E,Z) (M06-2X/6-311G(d,p), $CHCl_3$)

Energy (Hartrees): = -1936.2122043
No imaginary frequencies

Standard orientation:

Center Number	Atomic Number	Atomic Type	Coordinates (Angstroms) X	Y	Z
1	6	0	-0.253315	0.853984	-1.052546
2	6	0	-0.035715	-0.499867	-0.343014
3	6	0	-1.356145	-1.266463	-0.371552
4	6	0	-2.472497	-0.432976	0.234957
5	6	0	-2.537896	0.928214	-0.455953
6	1	0	-0.520737	0.687655	-2.101034
7	1	0	-1.616902	-1.520210	-1.403511
8	1	0	-2.310768	-0.311646	1.308390
9	1	0	-2.812486	0.790638	-1.510943
10	1	0	0.208902	-0.298510	0.704156
11	8	0	-1.268620	1.559205	-0.373933
12	6	0	-3.537749	1.844859	0.217991
13	1	0	-3.266235	1.999792	1.262653
14	1	0	-4.538803	1.421218	0.147231
15	8	0	-3.559965	3.090153	-0.475965
16	8	0	-3.709433	-1.101148	-0.000338
17	8	0	-1.247209	-2.461127	0.394530
18	7	0	0.943510	1.683130	-1.088108
19	6	0	-2.844617	4.109134	0.053097
20	8	0	-2.319900	4.064911	1.130693
21	6	0	-2.810864	5.271964	-0.891091
22	1	0	-3.816335	5.495586	-1.248438
23	1	0	-2.379527	6.135970	-0.391752
24	1	0	-2.198655	5.001044	-1.754275
25	6	0	-4.281643	-1.775322	1.031051
26	8	0	-3.829761	-1.790262	2.140443
27	6	0	-5.528131	-2.466600	0.572768
28	1	0	-5.279453	-3.166965	-0.226411
29	1	0	-5.981032	-2.993166	1.408791
30	1	0	-6.225131	-1.731664	0.166459
31	6	0	-1.020018	-3.619786	-0.270891
32	8	0	-0.892524	-3.676949	-1.463376
33	6	0	-0.945353	-4.769030	0.683361
34	1	0	-0.020226	-4.682194	1.257894
35	1	0	-1.780059	-4.729264	1.383770
36	1	0	-0.944604	-5.703928	0.128770
37	6	0	2.337564	-1.097353	-0.856632
38	7	0	1.307411	2.068972	0.029526
39	7	0	1.724934	2.469265	0.987497
40	7	0	0.997193	-1.297063	-0.975418
41	1	0	0.727792	-1.789615	-1.818735
42	7	0	2.729219	-0.511568	0.303290
43	6	0	4.036455	-0.030956	0.589527
44	6	0	4.598770	0.964751	-0.206816
45	6	0	4.712750	-0.508771	1.706136
46	6	0	5.854401	1.463853	0.109018
47	1	0	4.049346	1.334817	-1.064968
48	6	0	5.964846	0.008658	2.025186
49	1	0	4.260993	-1.285333	2.313295
50	6	0	6.540189	0.989059	1.225399
51	1	0	6.294916	2.234068	-0.512841
52	1	0	6.491672	-0.363948	2.895596
53	1	0	7.518010	1.385680	1.471185
54	1	0	2.112144	-0.601074	1.101365
55	16	0	3.387775	-1.592530	-2.070659

Structure 15 (Z,Z) (M06-2X/def2-TZVP, gas phase)

Energy (Hartrees): = -1936.3739915
No imaginary frequencies

Standard orientation:

Center Number	Atomic Number	Atomic Type	Coordinates (Angstroms) X	Y	Z
1	6	0	0.843354	-1.246641	-0.813568
2	6	0	0.035886	-0.103407	-0.176388
3	6	0	0.773686	1.213016	-0.407330
4	6	0	2.206547	1.127858	0.079172
5	6	0	2.887351	-0.090953	-0.544676
6	1	0	0.891113	-1.103783	-1.900091
7	1	0	0.776095	1.466762	-1.472235
8	1	0	2.235627	1.057773	1.168240
9	1	0	2.943992	0.056805	-1.633652
10	1	0	-0.039429	-0.295746	0.897435
11	8	0	2.139454	-1.252338	-0.261403
12	6	0	4.282924	-0.318617	-0.001547
13	1	0	4.245658	-0.524550	1.067430
14	1	0	4.911324	0.550869	-0.186706
15	8	0	4.863350	-1.411804	-0.700413
16	8	0	2.856492	2.321956	-0.349868
17	8	0	0.133653	2.263809	0.313540
18	7	0	0.238148	-2.546794	-0.629762
19	6	0	4.932225	-2.600224	-0.053883
20	8	0	4.655092	-2.750199	1.098544
21	6	0	5.403003	-3.675299	-0.988185
22	1	0	6.290945	-3.345139	-1.524880
23	1	0	5.607089	-4.580023	-0.424641
24	1	0	4.619976	-3.865129	-1.723119
25	6	0	3.727068	2.909032	0.503923
26	8	0	4.033168	2.436464	1.559278
27	6	0	4.239851	4.198145	-0.064297
28	1	0	3.407105	4.884794	-0.214742
29	1	0	4.964563	4.628464	0.619241
30	1	0	4.695287	4.015091	-1.037123
31	6	0	-0.797026	2.996457	-0.323730
32	8	0	-1.113406	2.819005	-1.470455
33	6	0	-1.384481	4.027954	0.588833
34	1	0	-1.975579	3.516561	1.350108
35	1	0	-0.593051	4.575148	1.097952
36	1	0	-2.017630	4.698650	0.017124
37	6	0	-2.428056	-0.374163	-0.043560
38	7	0	0.313419	-2.969048	0.532303
39	7	0	0.341952	-3.451965	1.534163
40	7	0	-1.296942	-0.009670	-0.713534
41	1	0	-1.406577	0.626010	-1.492307
42	7	0	-3.552909	-0.060563	-0.747641
43	1	0	-4.723250	0.430494	1.621623
44	1	0	-5.374558	-1.031467	-2.341074
45	1	0	-7.113355	0.096753	2.134468
46	1	0	-7.773124	-1.335292	-1.843734
47	1	0	-8.649284	-0.788943	0.407275
48	1	0	-3.416768	0.202990	-1.712487
49	16	0	-2.412955	-1.118948	1.439675
50	6	0	-5.394044	0.041094	0.871071
51	6	0	-6.737764	-0.150614	1.150087
52	6	0	-7.601234	-0.644324	0.181130
53	6	0	-7.111148	-0.949580	-1.079526
54	6	0	-5.765384	-0.774674	-1.363502
55	6	0	-4.903918	-0.282194	-0.389256

Structure 15 (Z,E) (M06-2X/def2-TZVP, gas phase)

Energy (Hartrees): = -1936.3786132
No imaginary frequencies

Standard orientation:

Center Number	Atomic Number	Atomic Type	Coordinates (Angstroms) X	Y	Z
1	6	0	0.427537	-1.280186	-0.211262
2	6	0	-0.226001	-0.095563	0.516972
3	6	0	0.313992	1.211132	-0.054662
4	6	0	1.829616	1.225491	-0.030795
5	6	0	2.371209	-0.036138	-0.705035
6	1	0	0.137102	-1.272278	-1.269402
7	1	0	-0.029480	1.349952	-1.084664
8	1	0	2.192617	1.285713	0.997234
9	1	0	2.085254	-0.018859	-1.767280
10	1	0	0.032045	-0.156410	1.577293
11	8	0	1.825545	-1.182309	-0.086584
12	6	0	3.877605	-0.149626	-0.597904
13	1	0	4.180562	-0.220912	0.445980
14	1	0	4.357914	0.710741	-1.060222
15	8	0	4.295035	-1.301675	-1.319001
16	8	0	2.239279	2.380712	-0.758648
17	8	0	-0.145691	2.298052	0.738463
18	7	0	-0.016509	-2.563779	0.291232
19	6	0	4.645111	-2.394131	-0.599827
20	8	0	4.741366	-2.407106	0.591167
21	6	0	4.886929	-3.563249	-1.508451
22	1	0	5.491973	-3.262814	-2.361853
23	1	0	5.370305	-4.359524	-0.951258
24	1	0	3.924742	-3.912143	-1.885693
25	6	0	3.288397	3.089050	-0.281137
26	8	0	3.934791	2.749585	0.666848
27	6	0	3.517241	4.319392	-1.106495
28	1	0	2.617074	4.933116	-1.104072
29	1	0	4.356832	4.873780	-0.699944
30	1	0	3.717490	4.032708	-2.138838
31	6	0	-1.272453	2.930236	0.348876
32	8	0	-1.861982	2.672323	-0.663697
33	6	0	-1.675781	3.959481	1.359198
34	1	0	-2.008242	3.439691	2.259309
35	1	0	-0.821666	4.577101	1.631228
36	1	0	-2.482821	4.564828	0.959465
37	6	0	-2.499929	-0.501811	1.397309
38	7	0	0.408412	-2.822064	1.427522
39	7	0	0.748794	-3.161594	2.430316
40	7	0	-1.658161	-0.134021	0.406166
41	1	0	-2.064888	0.178641	-0.465815
42	7	0	-3.808678	-0.583393	1.032765
43	6	0	-4.328345	-0.674865	-0.284432
44	6	0	-3.778618	-1.565848	-1.204687
45	6	0	-5.425696	0.100918	-0.638792
46	6	0	-4.320239	-1.659575	-2.477047
47	1	0	-2.940596	-2.186601	-0.909243
48	6	0	-5.975183	-0.015877	-1.907024
49	1	0	-5.836384	0.796807	0.081792
50	6	0	-5.420373	-0.889100	-2.831328
51	1	0	-3.892815	-2.354290	-3.188500
52	1	0	-6.832454	0.587309	-2.175916
53	1	0	-5.845275	-0.973494	-3.822719
54	1	0	-4.434662	-0.747434	1.805712
55	16	0	-2.039281	-0.796670	2.972634

Structure 15 (E,Z) (M06-2X/def2-TZVP, gas phase)

Energy (Hartrees): = -1936.368488
No imaginary frequencies

Standard orientation:

Center Number	Atomic Number	Atomic Type	Coordinates (Angstroms) X	Y	Z
1	6	0	-0.135826	0.777778	-0.983150
2	6	0	-0.014901	-0.586135	-0.271218
3	6	0	-1.378142	-1.270814	-0.318977
4	6	0	-2.466474	-0.373901	0.242583
5	6	0	-2.432800	0.978626	-0.462893
6	1	0	-0.370794	0.616517	-2.041364
7	1	0	-1.626243	-1.518907	-1.355548
8	1	0	-2.332371	-0.247152	1.319055
9	1	0	-2.686794	0.838264	-1.523424
10	1	0	0.223499	-0.391483	0.779214
11	8	0	-1.140785	1.536582	-0.353370
12	6	0	-3.401149	1.960712	0.160006
13	1	0	-3.128363	2.149804	1.198441
14	1	0	-4.414862	1.566549	0.102366
15	8	0	-3.373788	3.171728	-0.580556
16	8	0	-3.727028	-0.978248	-0.020586
17	8	0	-1.353643	-2.464531	0.448311
18	7	0	1.094063	1.547608	-0.979992
19	6	0	-2.687801	4.211123	-0.045264
20	8	0	-2.224181	4.205082	1.056242
21	6	0	-2.601499	5.342240	-1.025729
22	1	0	-3.572670	5.522090	-1.483111
23	1	0	-2.239302	6.231749	-0.520183
24	1	0	-1.905762	5.060748	-1.817248
25	6	0	-4.383174	-1.566227	1.012641
26	8	0	-3.988515	-1.553573	2.140044
27	6	0	-5.649731	-2.205542	0.527880
28	1	0	-5.414249	-2.947016	-0.235295
29	1	0	-6.161378	-2.670690	1.364124
30	1	0	-6.286619	-1.450898	0.066808
31	6	0	-1.186941	-3.633542	-0.216234
32	8	0	-1.088985	-3.705987	-1.407381
33	6	0	-1.137591	-4.782336	0.742742
34	1	0	-0.187082	-4.744274	1.276538
35	1	0	-1.935414	-4.694288	1.478286
36	1	0	-1.209092	-5.715490	0.193295
37	6	0	2.327052	-1.251529	-0.850051
38	7	0	1.432110	1.913382	0.151529
39	7	0	1.822145	2.285956	1.126459
40	7	0	0.967535	-1.462668	-0.872517
41	1	0	0.670568	-1.932789	-1.718504
42	7	0	2.778558	-0.731060	0.324979
43	6	0	4.046126	-0.156946	0.573535
44	6	0	4.619816	0.721797	-0.339490
45	6	0	4.674920	-0.413544	1.784985
46	6	0	5.828109	1.325319	-0.036878
47	1	0	4.114003	0.923208	-1.273680
48	6	0	5.879839	0.205709	2.083979
49	1	0	4.222770	-1.102286	2.488795
50	6	0	6.463680	1.071884	1.172638
51	1	0	6.271969	2.008058	-0.749733
52	1	0	6.363992	0.002663	3.030268
53	1	0	7.405995	1.550715	1.403063
54	1	0	2.193640	-0.859536	1.136356
55	16	0	3.271125	-1.680283	-2.133748

Structure 15 (Z,Z) (M06-2X/def2-TZVP, $CHCl_3$)

Energy (Hartrees): = -1936.4119175
No imaginary frequencies

Standard orientation:

Center Number	Atomic Number	Atomic Type	Coordinates (Angstroms) X	Y	Z
1	6	0	0.732539	-1.203442	-0.766713
2	6	0	0.013233	-0.007866	-0.119998
3	6	0	0.827752	1.253612	-0.396380
4	6	0	2.256829	1.076982	0.078284
5	6	0	2.852691	-0.182525	-0.553211
6	1	0	0.754880	-1.077367	-1.854333
7	1	0	0.830523	1.473628	-1.468012
8	1	0	2.287227	1.003693	1.166872
9	1	0	2.896259	-0.045503	-1.643004
10	1	0	-0.040996	-0.177944	0.957691
11	8	0	2.037168	-1.297887	-0.248289
12	6	0	4.238676	-0.487942	-0.028458
13	1	0	4.209449	-0.682861	1.042432
14	1	0	4.914586	0.340810	-0.234633
15	8	0	4.749294	-1.619659	-0.728249
16	8	0	2.983111	2.222523	-0.358706
17	8	0	0.269874	2.358713	0.307378
18	7	0	0.048585	-2.465082	-0.538461
19	6	0	4.770395	-2.806683	-0.089776
20	8	0	4.493469	-2.945506	1.069634
21	6	0	5.182741	-3.901469	-1.023119
22	1	0	6.088431	-3.618935	-1.558768
23	1	0	5.340015	-4.820576	-0.466437
24	1	0	4.391262	-4.048677	-1.760042
25	6	0	3.882491	2.771501	0.486833
26	8	0	4.134346	2.309466	1.565332
27	6	0	4.503633	3.991687	-0.113005
28	1	0	3.726372	4.717862	-0.352176
29	1	0	5.217255	4.418914	0.585141
30	1	0	5.004553	3.722096	-1.043733
31	6	0	-0.575587	3.174015	-0.351314
32	8	0	-0.890988	3.007594	-1.501123
33	6	0	-1.052912	4.278969	0.533290
34	1	0	-1.596960	3.848181	1.375207
35	1	0	-0.198225	4.823561	0.935118
36	1	0	-1.699284	4.945905	-0.029157
37	6	0	-2.460851	-0.235045	-0.001807
38	7	0	0.149677	-2.876837	0.621308
39	7	0	0.196658	-3.342048	1.631649
40	7	0	-1.323960	0.153262	-0.627165
41	1	0	-1.411807	0.664332	-1.496760
42	7	0	-3.567195	-0.017708	-0.756668
43	1	0	-4.858920	0.740336	1.474904
44	1	0	-5.273422	-1.292331	-2.264299
45	1	0	-7.245164	0.346200	1.955020
46	1	0	-7.674459	-1.658033	-1.805563
47	1	0	-8.665729	-0.854932	0.319456
48	1	0	-3.410854	0.188772	-1.734931
49	16	0	-2.480973	-0.921575	1.526663
50	6	0	-5.475415	0.204984	0.767638
51	6	0	-6.818705	-0.017749	1.028653
52	6	0	-7.617140	-0.687383	0.109538
53	6	0	-7.062467	-1.136723	-1.080400
54	6	0	-5.715642	-0.930449	-1.343374
55	6	0	-4.919924	-0.263638	-0.418190

Structure 15 (Z,E) (M06-2X/def2-TZVP, $CHCl_3$)

Energy (Hartrees): = -1936.4156339
No imaginary frequencies

Standard orientation:

Center Number	Atomic Number	Atomic Type	Coordinates (Angstroms) X	Y	Z
1	6	0	0.571705	-1.326958	-0.328509
2	6	0	-0.184869	-0.276886	0.501491
3	6	0	0.236666	1.114090	0.036834
4	6	0	1.744777	1.261014	0.087456
5	6	0	2.397411	0.124898	-0.700865
6	1	0	0.271386	-1.257351	-1.379413
7	1	0	-0.106893	1.290095	-0.986469
8	1	0	2.091606	1.247986	1.122297
9	1	0	2.109215	0.213023	-1.757837
10	1	0	0.082977	-0.404510	1.552949
11	8	0	1.956270	-1.119867	-0.190867
12	6	0	3.905674	0.142444	-0.586003
13	1	0	4.212396	-0.004456	0.448524
14	1	0	4.305469	1.084245	-0.958975
15	8	0	4.435393	-0.888397	-1.416032
16	8	0	2.056978	2.511231	-0.520485
17	8	0	-0.324012	2.100413	0.897013
18	7	0	0.261167	-2.689700	0.070855
19	6	0	4.910912	-2.002148	-0.824785
20	8	0	5.016272	-2.133860	0.363638
21	6	0	5.279873	-3.029411	-1.848931
22	1	0	5.884698	-2.579390	-2.635358
23	1	0	5.815231	-3.845761	-1.373118
24	1	0	4.364085	-3.409042	-2.305827
25	6	0	3.001798	3.286862	0.054746
26	8	0	3.640361	2.941295	1.010377
27	6	0	3.125583	4.594206	-0.659427
28	1	0	2.178995	5.131430	-0.587988
29	1	0	3.923189	5.180184	-0.212832
30	1	0	3.329317	4.417732	-1.715759
31	6	0	-1.472362	2.697426	0.521315
32	8	0	-2.041969	2.443274	-0.507455
33	6	0	-1.924327	3.681110	1.550722
34	1	0	-2.178521	3.138168	2.462918
35	1	0	-1.114948	4.370412	1.789448
36	1	0	-2.793299	4.220789	1.186503
37	6	0	-2.428969	-0.985538	1.274726
38	7	0	0.743618	-3.002989	1.164115
39	7	0	1.148418	-3.387606	2.126929
40	7	0	-1.612156	-0.421556	0.366444
41	1	0	-2.031716	0.014640	-0.445638
42	7	0	-3.735875	-1.068237	0.930500
43	6	0	-4.343187	-0.782554	-0.319042
44	6	0	-3.828477	-1.296162	-1.507442
45	6	0	-5.509960	-0.024783	-0.335705
46	6	0	-4.469965	-1.023933	-2.707121
47	1	0	-2.944316	-1.921773	-1.486397
48	6	0	-6.155515	0.224352	-1.537290
49	1	0	-5.902595	0.365672	0.595384
50	6	0	-5.633617	-0.266237	-2.727106
51	1	0	-4.067064	-1.424718	-3.628734
52	1	0	-7.064499	0.812291	-1.542971
53	1	0	-6.134835	-0.064615	-3.664990
54	1	0	-4.343670	-1.361179	1.681624
55	16	0	-1.923479	-1.545421	2.779430

Structure 15 (E,Z) (M06-2X/def2-TZVP, $CHCl_3$)

Energy (Hartrees): = -1936.4086464
No imaginary frequencies

Standard orientation:

Center Number	Atomic Number	Atomic Type	Coordinates (Angstroms) X	Y	Z
1	6	0	-0.202467	0.802000	-1.039145
2	6	0	0.007844	-0.546052	-0.320894
3	6	0	-1.312744	-1.308912	-0.355184
4	6	0	-2.435597	-0.469262	0.225228
5	6	0	-2.491299	0.885989	-0.475368
6	1	0	-0.454614	0.624040	-2.088992
7	1	0	-1.559183	-1.570777	-1.387627
8	1	0	-2.286165	-0.333206	1.297842
9	1	0	-2.755191	0.737393	-1.531159
10	1	0	0.248186	-0.339505	0.725614
11	8	0	-1.225229	1.514596	-0.387002
12	6	0	-3.499259	1.802092	0.180697
13	1	0	-3.226774	1.987634	1.219197
14	1	0	-4.492064	1.356329	0.131630
15	8	0	-3.556013	3.027218	-0.542898
16	8	0	-3.662419	-1.148241	-0.016557
17	8	0	-1.215383	-2.495241	0.421799
18	7	0	0.993344	1.631118	-1.068721
19	6	0	-2.935205	4.102926	-0.018096
20	8	0	-2.436484	4.117163	1.073718
21	6	0	-2.957020	5.249672	-0.978954
22	1	0	-3.956805	5.379681	-1.391319
23	1	0	-2.627971	6.155229	-0.477827
24	1	0	-2.280996	5.022234	-1.805386
25	6	0	-4.335067	-1.669936	1.035798
26	8	0	-3.972217	-1.554895	2.172797
27	6	0	-5.571500	-2.371763	0.574020
28	1	0	-5.317701	-3.100801	-0.195272
29	1	0	-6.052968	-2.858453	1.416754
30	1	0	-6.250567	-1.642258	0.129550
31	6	0	-1.008852	-3.665359	-0.223639
32	8	0	-0.899341	-3.746072	-1.416883
33	6	0	-0.929010	-4.800395	0.743878
34	1	0	0.007589	-4.716658	1.298502
35	1	0	-1.747859	-4.744270	1.460175
36	1	0	-0.948863	-5.742536	0.204313
37	6	0	2.380383	-1.149179	-0.836574
38	7	0	1.350592	2.019400	0.046140
39	7	0	1.753306	2.417455	1.005996
40	7	0	1.038897	-1.349163	-0.943278
41	1	0	0.769696	-1.839384	-1.786742
42	7	0	2.780982	-0.567457	0.320284
43	6	0	4.057981	-0.016853	0.590048
44	6	0	4.631236	0.890465	-0.295534
45	6	0	4.691637	-0.322247	1.787299
46	6	0	5.848998	1.471912	0.013578
47	1	0	4.116139	1.134234	-1.215448
48	6	0	5.906776	0.275940	2.095471
49	1	0	4.234451	-1.027338	2.471306
50	6	0	6.491820	1.167967	1.208810
51	1	0	6.292409	2.176518	-0.678620
52	1	0	6.398224	0.036713	3.029998
53	1	0	7.440928	1.630221	1.447868
54	1	0	2.163715	-0.643080	1.117558
55	16	0	3.410706	-1.651981	-2.051455

Structure 25 (min 1) (M06-2X/6-311G(d,p), gas phase)

Energy (Hartrees): = -2435.0854761
No imaginary frequencies

Standard orientation:

Center Number	Atomic Number	Atomic Type	Coordinates (Angstroms) X	Y	Z
1	6	0	-1.528877	-1.381818	0.784478
2	6	0	-0.568618	-0.320331	0.219420
3	6	0	-1.138634	1.079630	0.456361
4	6	0	-2.567023	1.179979	-0.046993
5	6	0	-3.399362	0.043268	0.542571
6	1	0	-1.583561	-1.294431	1.876951
7	1	0	-1.119433	1.327136	1.524620
8	1	0	-2.579092	1.142758	-1.139292
9	1	0	-3.444731	0.153542	1.636772
10	1	0	-0.462025	-0.487860	-0.856870
11	8	0	-2.806995	-1.196538	0.210936
12	6	0	-4.808617	0.029014	-0.014177
13	1	0	-4.783046	-0.126489	-1.094007
14	1	0	-5.314368	0.963363	0.226879
15	8	0	-5.531910	-1.022223	0.613371
16	8	0	-3.127432	2.411895	0.404584
17	8	0	-0.376810	2.053979	-0.256725
18	7	0	-1.083006	-2.735024	0.532676
19	6	0	-5.688864	-2.168785	-0.099095
20	8	0	-5.405254	-2.279121	-1.253934
21	6	0	-6.260212	-3.247367	0.775527
22	1	0	-7.096146	-2.859810	1.356830
23	1	0	-6.570043	-4.085618	0.157453
24	1	0	-5.484950	-3.570688	1.472914
25	6	0	-3.201538	3.439095	-0.487269
26	8	0	-2.878154	3.346295	-1.632244
27	6	0	-3.743235	4.668001	0.184966
28	1	0	-3.051467	4.985550	0.966691
29	1	0	-3.863918	5.455811	-0.553400
30	1	0	-4.697296	4.439721	0.660834
31	6	0	0.690060	2.619831	0.340698
32	8	0	1.064736	2.331844	1.448693
33	6	0	1.325787	3.626101	-0.569302
34	1	0	1.832245	3.087981	-1.373746
35	1	0	0.560060	4.258012	-1.018731
36	1	0	2.048540	4.214404	-0.009792
37	6	0	1.877208	-0.724493	0.152350
38	7	0	-1.151188	-3.059823	-0.664347
39	7	0	-1.186344	-3.472942	-1.701981
40	7	0	0.736971	-0.412018	0.826066
41	1	0	0.854298	0.133290	1.670821
42	7	0	2.996264	-0.340533	0.847786
43	1	0	6.989505	1.198466	-0.750731
44	1	0	6.618004	-3.013496	-0.004003
45	1	0	7.974838	-1.090677	-0.757659
46	1	0	2.876360	0.461634	1.457997
47	16	0	1.907624	-1.483680	-1.327686
48	6	0	5.102481	0.505226	-0.022995
49	6	0	6.414347	0.339115	-0.432942
50	6	0	6.952665	-0.940030	-0.433289
51	6	0	6.187431	-2.018659	-0.012256
52	6	0	4.871545	-1.857626	0.417398
53	6	0	4.318559	-0.570738	0.397304
54	17	0	4.414286	2.112479	-0.032084
55	6	0	4.072182	-3.042452	0.881082
56	1	0	3.330481	-2.752558	1.626305
57	1	0	3.532122	-3.483263	0.039943
58	1	0	4.730745	-3.798470	1.309747

Structure 25 (min 2) (M06-2X/6-311G(d,p), gas phase)

Energy (Hartrees): = -2435.0888337
No imaginary frequencies

Standard orientation:

Center Number	Atomic Number	Atomic Type	Coordinates (Angstroms) X	Y	Z
1	6	0	0.967418	-1.463002	-0.263864
2	6	0	0.211545	-0.397150	0.548783
3	6	0	0.619060	1.008240	0.104724
4	6	0	2.133139	1.175301	0.007470
5	6	0	2.747713	-0.002084	-0.746364
6	1	0	0.661964	-1.420054	-1.317434
7	1	0	0.188083	1.247783	-0.874676
8	1	0	2.560323	1.272253	1.009566
9	1	0	2.405107	0.024478	-1.791804
10	1	0	0.458045	-0.525148	1.606318
11	8	0	2.352654	-1.218093	-0.147151
12	6	0	4.262372	0.039923	-0.721538
13	1	0	4.622852	-0.036364	0.305594
14	1	0	4.617268	0.959112	-1.185795
15	8	0	4.754237	-1.048601	-1.493301
16	8	0	2.436555	2.336743	-0.771117
17	8	0	0.086426	1.899177	1.083407
18	7	0	0.676299	-2.815169	0.158476
19	6	0	5.190755	-2.138099	-0.808768
20	8	0	5.341252	-2.163848	0.375904
21	6	0	5.446283	-3.275709	-1.754847
22	1	0	6.018622	-2.930346	-2.615267
23	1	0	5.971452	-4.069345	-1.230400
24	1	0	4.484377	-3.644978	-2.115914
25	6	0	2.543220	3.528362	-0.134456
26	8	0	2.495507	3.644568	1.057238
27	6	0	2.675005	4.643466	-1.126300
28	1	0	1.685335	4.799877	-1.562652
29	1	0	3.001868	5.545588	-0.616086
30	1	0	3.362767	4.371431	-1.925572
31	6	0	-0.363271	3.107487	0.681051
32	8	0	-0.425123	3.440016	-0.471843
33	6	0	-0.722501	3.952496	1.864238
34	1	0	-1.240766	3.360836	2.617997
35	1	0	0.216715	4.308081	2.295850
36	1	0	-1.323556	4.798564	1.540347
37	6	0	-2.053384	-1.052189	1.335282
38	7	0	1.154689	-3.097981	1.269588
39	7	0	1.551247	-3.474417	2.243631
40	7	0	-1.217301	-0.530984	0.405407
41	1	0	-1.615880	-0.260696	-0.485369
42	7	0	-3.376055	-0.995475	0.997712
43	1	0	-5.223975	-1.252048	-3.189834
44	1	0	-4.401952	2.644129	-1.578706
45	1	0	-5.238374	1.223080	-3.421407
46	1	0	-3.984387	-1.543895	1.588295
47	16	0	-1.567789	-1.683636	2.802229
48	6	0	-4.361872	-1.188149	-1.232128
49	6	0	-4.860076	-0.615158	-2.394586
50	6	0	-4.862540	0.767084	-2.513692
51	6	0	-4.391267	1.565250	-1.477609
52	6	0	-3.915711	1.002319	-0.295894
53	6	0	-3.891691	-0.397704	-0.180814
54	17	0	-4.314818	-2.918682	-1.084439
55	6	0	-3.468615	1.861312	0.856843
56	1	0	-4.158264	1.751765	1.697480
57	1	0	-2.480150	1.567974	1.215223
58	1	0	-3.438655	2.910195	0.560059

Structure 25 (max 1) (M06-2X/6-311G(d,p), gas phase)

Energy (Hartrees): = -2435.0676817
Imaginary frequency -32.49

Standard orientation:

Center Number	Atomic Number	Atomic Type	Coordinates (Angstroms) X	Y	Z
1	6	0	-1.667021	-1.311793	0.753016
2	6	0	-0.438946	-0.791791	-0.010103
3	6	0	-0.395916	0.734028	0.059686
4	6	0	-1.718203	1.375418	-0.342600
5	6	0	-2.870298	0.684166	0.388128
6	1	0	-1.582623	-1.054422	1.816228
7	1	0	-0.164392	1.060426	1.081463
8	1	0	-1.842986	1.329120	-1.428232
9	1	0	-2.788446	0.906576	1.462592
10	1	0	-0.510749	-1.116926	-1.052822
11	8	0	-2.814361	-0.713542	0.187730
12	6	0	-4.219678	1.156628	-0.113254
13	1	0	-4.343770	0.891863	-1.164652
14	1	0	-4.310152	2.233267	0.025499
15	8	0	-5.233539	0.536917	0.667975
16	8	0	-1.744687	2.734254	0.102103
17	8	0	0.658179	1.137016	-0.807966
18	7	0	-1.804810	-2.751070	0.720248
19	6	0	-5.866735	-0.530566	0.115655
20	8	0	-5.733598	-0.867799	-1.022526
21	6	0	-6.734563	-1.204992	1.138721
22	1	0	-7.292108	-0.464313	1.710611
23	1	0	-7.404572	-1.900531	0.640706
24	1	0	-6.088082	-1.749539	1.829927
25	6	0	-1.210775	3.685512	-0.704163
26	8	0	-0.824758	3.464012	-1.816098
27	6	0	-1.141119	5.000673	0.010395
28	1	0	-0.303519	4.940862	0.709706
29	1	0	-0.965018	5.794581	-0.710582
30	1	0	-2.051332	5.181036	0.580284
31	6	0	1.447545	2.166356	-0.421802
32	8	0	1.328840	2.723800	0.635445
33	6	0	2.418405	2.510169	-1.506376
34	1	0	2.899703	1.605268	-1.877242
35	1	0	1.846575	2.962652	-2.320211
36	1	0	3.155953	3.210946	-1.123986
37	6	0	1.594568	-2.238389	0.021473
38	7	0	-2.137766	-3.188725	-0.392660
39	7	0	-2.451371	-3.697111	-1.337007
40	7	0	0.811308	-1.268383	0.534168
41	1	0	1.229649	-0.687926	1.250202
42	7	0	2.918808	-2.241356	0.601080
43	1	0	4.665184	1.857011	1.834055
44	1	0	5.732309	0.248943	-2.000117
45	1	0	5.911843	2.052106	-0.316601
46	1	0	2.879360	-2.494047	1.583170
47	16	0	1.138562	-3.308386	-1.135543
48	6	0	3.817205	-0.033954	1.301664
49	6	0	4.602303	1.086844	1.076871
50	6	0	5.288505	1.187388	-0.124108
51	6	0	5.180769	0.175564	-1.069290
52	6	0	4.391981	-0.952368	-0.850320
53	6	0	3.689242	-1.066371	0.363797
54	17	0	2.991319	-0.150528	2.847916
55	6	0	4.321681	-2.041237	-1.884267
56	1	0	4.201774	-3.015326	-1.410428
57	1	0	3.458028	-1.903595	-2.539301
58	1	0	5.222445	-2.033344	-2.498988

Structure 25 (min 1) (M06-2X/6-311G(d,p), $CHCl_3$)

Energy (Hartrees): = -2435.1230361
No imaginary frequencies

Standard orientation:

Center Number	Atomic Number	Atomic Type	Coordinates (Angstroms) X	Y	Z
1	6	0	-1.358700	-1.330046	0.770512
2	6	0	-0.508627	-0.185113	0.189449
3	6	0	-1.216751	1.140664	0.462439
4	6	0	-2.645801	1.106470	-0.048826
5	6	0	-3.370328	-0.111402	0.520940
6	1	0	-1.415481	-1.242069	1.861134
7	1	0	-1.229097	1.350244	1.537262
8	1	0	-2.655624	1.084318	-1.140890
9	1	0	-3.445480	-0.014264	1.613389
10	1	0	-0.416669	-0.331327	-0.890488
11	8	0	-2.646414	-1.286164	0.195505
12	6	0	-4.755307	-0.258168	-0.071724
13	1	0	-4.691040	-0.411738	-1.149617
14	1	0	-5.351344	0.626746	0.150010
15	8	0	-5.406723	-1.366182	0.547474
16	8	0	-3.312023	2.275102	0.426050
17	8	0	-0.555791	2.208731	-0.214399
18	7	0	-0.779589	-2.639892	0.515818
19	6	0	-5.446447	-2.531546	-0.137307
20	8	0	-5.077219	-2.651287	-1.271714
21	6	0	-6.014558	-3.622446	0.719207
22	1	0	-6.943095	-3.288275	1.183143
23	1	0	-6.185270	-4.510307	0.115462
24	1	0	-5.301948	-3.847504	1.515820
25	6	0	-3.541344	3.285185	-0.452589
26	8	0	-3.286134	3.214822	-1.620473
27	6	0	-4.143353	4.461361	0.252248
28	1	0	-3.424822	4.848920	0.977628
29	1	0	-4.391955	5.231821	-0.473245
30	1	0	-5.034627	4.150239	0.799119
31	6	0	0.378985	2.913138	0.457780
32	8	0	0.703788	2.664761	1.590222
33	6	0	0.920318	4.017613	-0.392864
34	1	0	1.331852	3.596281	-1.311679
35	1	0	0.104842	4.686970	-0.674233
36	1	0	1.685774	4.562276	0.154281
37	6	0	1.949794	-0.559654	0.158329
38	7	0	-0.872910	-2.991408	-0.667924
39	7	0	-0.920221	-3.415479	-1.701248
40	7	0	0.813327	-0.153860	0.767624
41	1	0	0.902823	0.357055	1.639237
42	7	0	3.062768	-0.348855	0.906904
43	1	0	6.906949	1.206649	-1.040702
44	1	0	6.786026	-2.911753	0.178699
45	1	0	8.003786	-1.014311	-0.839189
46	1	0	2.964004	0.196751	1.756779
47	16	0	1.999598	-1.265217	-1.370074
48	6	0	5.087384	0.518659	-0.139831
49	6	0	6.387675	0.367030	-0.597069
50	6	0	6.990356	-0.878508	-0.480747
51	6	0	6.304583	-1.944387	0.090705
52	6	0	5.000251	-1.794602	0.557056
53	6	0	4.387099	-0.543569	0.428603
54	17	0	4.308369	2.074916	-0.285215
55	6	0	4.249501	-2.935822	1.182258
56	1	0	3.941514	-2.687680	2.201008
57	1	0	3.344296	-3.159106	0.611628
58	1	0	4.870877	-3.830978	1.214343

Structure 25 (min 2) (M06-2X/6-311G(d,p), $CHCl_3$)

Energy (Hartrees): = -2435.1275939

No imaginary frequencies

Standard orientation:

Center Number	Atomic Number	Atomic Type	Coordinates (Angstroms) X	Y	Z
1	6	0	0.945304	-1.457155	-0.245203
2	6	0	0.196334	-0.386179	0.564551
3	6	0	0.606402	1.010256	0.096910
4	6	0	2.122654	1.164053	0.020241
5	6	0	2.742668	-0.018084	-0.725375
6	1	0	0.636391	-1.420973	-1.295782
7	1	0	0.183424	1.222772	-0.890438
8	1	0	2.537843	1.250822	1.027655
9	1	0	2.413477	0.004533	-1.773740
10	1	0	0.450987	-0.500340	1.620722
11	8	0	2.331715	-1.233839	-0.125807
12	6	0	4.255196	0.025356	-0.670179
13	1	0	4.598438	-0.066965	0.360708
14	1	0	4.617448	0.954597	-1.107790
15	8	0	4.775196	-1.041370	-1.461813
16	8	0	2.452007	2.323086	-0.752346
17	8	0	0.064975	1.923781	1.052629
18	7	0	0.642682	-2.807095	0.201433
19	6	0	5.222642	-2.142051	-0.816677
20	8	0	5.315377	-2.224240	0.376131
21	6	0	5.582729	-3.214610	-1.799758
22	1	0	6.232224	-2.807843	-2.575601
23	1	0	6.072622	-4.035391	-1.281966
24	1	0	4.668947	-3.572204	-2.279168
25	6	0	2.587357	3.508179	-0.114754
26	8	0	2.489566	3.626879	1.076372
27	6	0	2.842526	4.610609	-1.092817
28	1	0	1.925740	4.773637	-1.664419
29	1	0	3.110904	5.517800	-0.557231
30	1	0	3.630895	4.325194	-1.789618
31	6	0	-0.372141	3.126167	0.620762
32	8	0	-0.394310	3.439762	-0.540593
33	6	0	-0.777256	3.990951	1.771987
34	1	0	-1.333318	3.417350	2.513298
35	1	0	0.139521	4.354706	2.243713
36	1	0	-1.360258	4.834361	1.409333
37	6	0	-2.078595	-1.030573	1.336284
38	7	0	1.133795	-3.079190	1.305024
39	7	0	1.544403	-3.437995	2.280998
40	7	0	-1.232971	-0.525348	0.417242
41	1	0	-1.616378	-0.266245	-0.485863
42	7	0	-3.390521	-0.996968	0.991330
43	1	0	-5.197811	-1.287689	-3.217526
44	1	0	-4.534383	2.623816	-1.569581
45	1	0	-5.311443	1.184160	-3.424902
46	1	0	-4.020715	-1.479365	1.618633
47	16	0	-1.599251	-1.639782	2.834804
48	6	0	-4.346069	-1.205121	-1.251552
49	6	0	-4.859781	-0.646678	-2.413591
50	6	0	-4.917999	0.736413	-2.520275
51	6	0	-4.480508	1.545135	-1.477381
52	6	0	-3.980902	0.992723	-0.299615
53	6	0	-3.911149	-0.405652	-0.192335
54	17	0	-4.242773	-2.939308	-1.112396
55	6	0	-3.543906	1.857686	0.850457
56	1	0	-4.180932	1.686654	1.722290
57	1	0	-2.520915	1.624631	1.154230
58	1	0	-3.598511	2.911874	0.578043

Structure 25 (min 1) (M06-2X/ def2-TZVP, gas phase)

Energy (Hartrees): = -2435.2920199
No imaginary frequencies

Standard orientation:

Center Number	Atomic Number	Atomic Type	Coordinates (Angstroms) X	Y	Z
1	6	0	-1.406395	-1.336980	0.796520
2	6	0	-0.478643	-0.260537	0.213409
3	6	0	-1.091660	1.119649	0.440722
4	6	0	-2.517453	1.177761	-0.068098
5	6	0	-3.321706	0.017502	0.520106
6	1	0	-1.466437	-1.223533	1.885693
7	1	0	-1.084840	1.366727	1.507500
8	1	0	-2.532835	1.127949	-1.158646
9	1	0	-3.381064	0.145557	1.611443
10	1	0	-0.375152	-0.441666	-0.860170
11	8	0	-2.684584	-1.204473	0.218791
12	6	0	-4.724072	-0.060189	-0.046691
13	1	0	-4.690240	-0.244622	-1.119672
14	1	0	-5.265410	0.863765	0.149390
15	8	0	-5.420805	-1.105497	0.618177
16	8	0	-3.059493	2.420653	0.371332
17	8	0	-0.344289	2.107465	-0.265877
18	7	0	-0.920527	-2.682237	0.582506
19	6	0	-5.603151	-2.263974	-0.060021
20	8	0	-5.326634	-2.413278	-1.212670
21	6	0	-6.197291	-3.306885	0.840008
22	1	0	-7.060172	-2.901351	1.365809
23	1	0	-6.477039	-4.174543	0.251158
24	1	0	-5.453725	-3.589631	1.585950
25	6	0	-3.857334	3.101026	-0.484793
26	8	0	-4.186091	2.678458	-1.554345
27	6	0	-4.262916	4.418416	0.104611
28	1	0	-3.373709	5.007658	0.326485
29	1	0	-4.902686	4.945260	-0.595729
30	1	0	-4.787774	4.249354	1.044681
31	6	0	0.666440	2.730168	0.367387
32	8	0	0.989265	2.491537	1.502024
33	6	0	1.319813	3.733449	-0.530905
34	1	0	1.779307	3.205539	-1.367348
35	1	0	0.571117	4.413300	-0.935192
36	1	0	2.076114	4.278434	0.025179
37	6	0	1.969366	-0.643110	0.149460
38	7	0	-0.985462	-3.049701	-0.598534
39	7	0	-1.016239	-3.489013	-1.620341
40	7	0	0.832107	-0.299182	0.807046
41	1	0	0.942639	0.256141	1.644499
42	7	0	3.091260	-0.266327	0.838910
43	1	0	7.030733	1.288865	-0.855291
44	1	0	6.734402	-2.901055	0.000348
45	1	0	8.046624	-0.980686	-0.821945
46	1	0	2.979591	0.529652	1.455580
47	16	0	1.999113	-1.425136	-1.311565
48	6	0	5.166197	0.589691	-0.081691
49	6	0	6.472379	0.430955	-0.508188
50	6	0	7.028272	-0.837056	-0.485834
51	6	0	6.287854	-1.914863	-0.026812
52	6	0	4.979245	-1.762639	0.418398
53	6	0	4.409204	-0.486674	0.375282
54	17	0	4.454273	2.173925	-0.116372
55	6	0	4.202335	-2.938479	0.929303
56	1	0	3.572989	-2.656261	1.773395
57	1	0	3.542024	-3.322520	0.149725
58	1	0	4.877271	-3.733967	1.240922

Structure 25 (min 2) (M06-2X/ def2-TZVP, gas phase)

Energy (Hartrees): = -2435.2929242
No imaginary frequencies

Standard orientation:

Center Number	Atomic Number	Atomic Type	Coordinates (Angstroms) X	Y	Z
1	6	0	0.931484	-1.463692	-0.246757
2	6	0	0.186372	-0.389918	0.560718
3	6	0	0.621434	1.004220	0.115138
4	6	0	2.136642	1.148722	0.032586
5	6	0	2.747285	-0.041319	-0.703822
6	1	0	0.635915	-1.407990	-1.301852
7	1	0	0.205688	1.237440	-0.871607
8	1	0	2.552489	1.240415	1.039028
9	1	0	2.432022	-0.005295	-1.756734
10	1	0	0.423685	-0.525015	1.618750
11	8	0	2.317224	-1.249120	-0.119393
12	6	0	4.258329	-0.020872	-0.636252
13	1	0	4.587424	-0.156183	0.394220
14	1	0	4.635740	0.920361	-1.032951
15	8	0	4.766526	-1.067407	-1.451305
16	8	0	2.465726	2.300373	-0.743736
17	8	0	0.091860	1.911895	1.075872
18	7	0	0.610259	-2.812738	0.161464
19	6	0	5.313112	-2.137475	-0.826288
20	8	0	5.505447	-2.194349	0.351938
21	6	0	5.635524	-3.214467	-1.819034
22	1	0	6.195264	-2.798199	-2.655102
23	1	0	6.200999	-3.999956	-1.328112
24	1	0	4.701800	-3.619039	-2.210924
25	6	0	2.598458	3.491530	-0.118873
26	8	0	2.543508	3.621899	1.070955
27	6	0	2.784930	4.592223	-1.115694
28	1	0	1.819177	4.762863	-1.595065
29	1	0	3.108570	5.493589	-0.604850
30	1	0	3.498331	4.299364	-1.883515
31	6	0	-0.339206	3.119694	0.663522
32	8	0	-0.382146	3.448961	-0.491114
33	6	0	-0.715246	3.971903	1.833966
34	1	0	-1.263060	3.389187	2.572319
35	1	0	0.214226	4.313830	2.294072
36	1	0	-1.294648	4.825359	1.495021
37	6	0	-2.088288	-1.009449	1.334741
38	7	0	1.065976	-3.116704	1.272370
39	7	0	1.442765	-3.501383	2.245762
40	7	0	-1.241141	-0.502016	0.410207
41	1	0	-1.632823	-0.208937	-0.474753
42	7	0	-3.406894	-0.945182	0.989637
43	1	0	-5.272654	-1.290756	-3.174382
44	1	0	-4.411479	2.631728	-1.669963
45	1	0	-5.260524	1.173707	-3.472684
46	1	0	-4.013491	-1.499119	1.574947
47	16	0	-1.621174	-1.647957	2.796920
48	6	0	-4.402490	-1.185063	-1.225693
49	6	0	-4.898541	-0.637382	-2.398762
50	6	0	-4.887324	0.738240	-2.555053
51	6	0	-4.407851	1.556667	-1.542489
52	6	0	-3.935888	1.021216	-0.349537
53	6	0	-3.918582	-0.372778	-0.200131
54	17	0	-4.381947	-2.901108	-1.031257
55	6	0	-3.492781	1.908164	0.779722
56	1	0	-4.195983	1.835489	1.611907
57	1	0	-2.516397	1.614689	1.166402
58	1	0	-3.444565	2.945804	0.452606

Structure 25 (min 1) (M06-2X/ def2-TZVP, $CHCl_3$)

Energy (Hartrees): = -2435.3289242
No imaginary frequencies

Standard orientation:

Center Number	Atomic Number	Atomic Type	Coordinates (Angstroms) X	Y	Z
1	6	0	-1.288488	-1.294589	0.768414
2	6	0	-0.445984	-0.154601	0.175244
3	6	0	-1.159424	1.169652	0.434611
4	6	0	-2.590145	1.129160	-0.065992
5	6	0	-3.305015	-0.091907	0.513597
6	1	0	-1.341653	-1.192201	1.857255
7	1	0	-1.165104	1.387380	1.506610
8	1	0	-2.609054	1.095627	-1.156882
9	1	0	-3.371378	0.020875	1.604831
10	1	0	-0.353927	-0.315710	-0.901843
11	8	0	-2.575808	-1.262852	0.201361
12	6	0	-4.694112	-0.265644	-0.060679
13	1	0	-4.647014	-0.427364	-1.136545
14	1	0	-5.305947	0.608801	0.154631
15	8	0	-5.320314	-1.372029	0.583936
16	8	0	-3.215800	2.321653	0.401983
17	8	0	-0.493572	2.224653	-0.253650
18	7	0	-0.707303	-2.605869	0.532108
19	6	0	-5.392707	-2.539658	-0.085478
20	8	0	-5.053598	-2.675450	-1.228548
21	6	0	-5.953035	-3.615942	0.790747
22	1	0	-6.876157	-3.273881	1.257825
23	1	0	-6.131966	-4.512424	0.204608
24	1	0	-5.235605	-3.830120	1.584719
25	6	0	-4.052039	2.980373	-0.429601
26	8	0	-4.352826	2.569646	-1.516226
27	6	0	-4.534529	4.248853	0.197065
28	1	0	-3.679873	4.874687	0.455718
29	1	0	-5.190812	4.771323	-0.492295
30	1	0	-5.067801	4.016285	1.119812
31	6	0	0.407315	2.964146	0.420187
32	8	0	0.701271	2.759671	1.569978
33	6	0	0.958571	4.051351	-0.442854
34	1	0	1.348153	3.623773	-1.366696
35	1	0	0.151591	4.735561	-0.709904
36	1	0	1.738867	4.586192	0.090309
37	6	0	2.013776	-0.509791	0.147193
38	7	0	-0.784597	-2.972473	-0.644589
39	7	0	-0.820168	-3.401755	-1.671181
40	7	0	0.874092	-0.103747	0.748692
41	1	0	0.960017	0.416373	1.613244
42	7	0	3.123041	-0.272498	0.888370
43	1	0	6.973170	1.252501	-1.053235
44	1	0	6.832214	-2.851167	0.188545
45	1	0	8.059262	-0.968071	-0.836399
46	1	0	3.018848	0.285518	1.727646
47	16	0	2.070062	-1.242126	-1.358413
48	6	0	5.150579	0.579889	-0.154130
49	6	0	6.449680	0.418487	-0.606164
50	6	0	7.046484	-0.825669	-0.481540
51	6	0	6.355908	-1.883069	0.093534
52	6	0	5.054023	-1.725399	0.555908
53	6	0	4.447296	-0.474678	0.419719
54	17	0	4.383763	2.128228	-0.312689
55	6	0	4.302556	-2.859655	1.185539
56	1	0	4.005665	-2.611767	2.206564
57	1	0	3.391455	-3.075917	0.623699
58	1	0	4.917555	-3.757924	1.211720

Structure 25 (min 2) (M06-2X/ def2-TZVP, $CHCl_3$)

Energy (Hartrees): = -2435.3312857
No imaginary frequencies

Standard orientation:

Center Number	Atomic Number	Atomic Type	Coordinates (Angstroms) X	Y	Z
1	6	0	0.921667	-1.448120	-0.241045
2	6	0	0.183563	-0.373959	0.569701
3	6	0	0.612188	1.014783	0.101257
4	6	0	2.127960	1.154358	0.027438
5	6	0	2.743467	-0.036236	-0.706749
6	1	0	0.615319	-1.400937	-1.291277
7	1	0	0.195420	1.222224	-0.888959
8	1	0	2.539749	1.237957	1.035600
9	1	0	2.433431	-0.005335	-1.760020
10	1	0	0.436847	-0.495894	1.624624
11	8	0	2.307804	-1.245786	-0.119912
12	6	0	4.252939	-0.010506	-0.620242
13	1	0	4.572195	-0.130226	0.414290
14	1	0	4.633829	0.927600	-1.020556
15	8	0	4.783438	-1.058206	-1.426484
16	8	0	2.469587	2.305948	-0.744823
17	8	0	0.078319	1.939090	1.046287
18	7	0	0.601464	-2.797487	0.192488
19	6	0	5.292344	-2.143222	-0.809763
20	8	0	5.415023	-2.237327	0.380704
21	6	0	5.676572	-3.188689	-1.809398
22	1	0	6.297540	-2.750482	-2.590125
23	1	0	6.204145	-3.996505	-1.311281
24	1	0	4.770679	-3.574410	-2.279737
25	6	0	2.630179	3.489751	-0.118865
26	8	0	2.532874	3.621925	1.071048
27	6	0	2.924936	4.576729	-1.100671
28	1	0	2.035178	4.740167	-1.711201
29	1	0	3.181701	5.488813	-0.570345
30	1	0	3.736295	4.276454	-1.762931
31	6	0	-0.375591	3.129834	0.613062
32	8	0	-0.409559	3.439570	-0.549405
33	6	0	-0.796416	3.991638	1.758595
34	1	0	-1.362066	3.414232	2.488332
35	1	0	0.108502	4.356399	2.249438
36	1	0	-1.376684	4.833495	1.392052
37	6	0	-2.094648	-0.991643	1.342302
38	7	0	1.073024	-3.085045	1.296797
39	7	0	1.467333	-3.448611	2.272370
40	7	0	-1.244753	-0.495599	0.424152
41	1	0	-1.627092	-0.219857	-0.472642
42	7	0	-3.403264	-0.958196	0.992496
43	1	0	-5.198559	-1.346504	-3.205199
44	1	0	-4.573147	2.595833	-1.637080
45	1	0	-5.330155	1.115788	-3.463577
46	1	0	-4.030084	-1.437524	1.623437
47	16	0	-1.625720	-1.601131	2.835055
48	6	0	-4.352420	-1.219318	-1.242900
49	6	0	-4.867161	-0.687371	-2.414615
50	6	0	-4.936262	0.689689	-2.549956
51	6	0	-4.509466	1.520745	-1.524011
52	6	0	-4.009675	0.996713	-0.336464
53	6	0	-3.925175	-0.395558	-0.202019
54	17	0	-4.241052	-2.938774	-1.070845
55	6	0	-3.592953	1.889562	0.795861
56	1	0	-4.249481	1.746849	1.657086
57	1	0	-2.580264	1.661807	1.132139
58	1	0	-3.636681	2.935084	0.494868

Structure 33 (min 1) (M06-2X/6-311G(d,p), gas phase)

Energy (Hartrees): = -1317.3980826
No imaginary frequencies

Standard orientation:

Center Number	Atomic Number	Atomic Type	Coordinates (Angstroms) X	Y	Z
1	6	0	1.703565	-0.048233	-0.097890
2	7	0	2.839480	0.372833	-0.693708
3	1	0	2.803708	0.592368	-1.677305
4	7	0	0.586647	0.137126	-0.891552
5	1	0	-3.471027	1.548866	0.681593
6	1	0	-2.911386	-2.662187	0.055466
7	1	0	-4.351765	-0.779986	0.754659
8	1	0	0.648644	0.923679	-1.526458
9	16	0	1.663473	-0.716446	1.425694
10	6	0	-1.556572	0.923515	-0.038095
11	6	0	-2.858704	0.708431	0.383269
12	6	0	-3.338351	-0.592918	0.421534
13	6	0	-2.526188	-1.649303	0.030261
14	6	0	-1.220729	-1.439049	-0.407016
15	6	0	-0.728033	-0.128484	-0.429274
16	17	0	-0.939539	2.558041	-0.070531
17	6	0	-0.353779	-2.589559	-0.833136
18	1	0	0.262780	-2.314423	-1.690335
19	1	0	0.321745	-2.869231	-0.020912
20	1	0	-0.964862	-3.453169	-1.095891
21	6	0	4.145673	0.264460	-0.071607
22	1	0	4.880032	0.697963	-0.749146
23	1	0	4.156340	0.808200	0.873199
24	1	0	4.398210	-0.778529	0.131598

Structure 33 (min 2) (M06-2X/6-311G(d,p), gas phase)

Energy (Hartrees): = -1317.4029352
No imaginary frequencies

Standard orientation:

Center Number	Atomic Number	Atomic Type	Coordinates (Angstroms) X	Y	Z
1	6	0	-1.730877	0.123062	-0.129550
2	7	0	-1.612835	-0.535450	1.038962
3	1	0	-0.686092	-0.657267	1.422407
4	7	0	-0.558212	0.341973	-0.805589
5	1	0	3.674347	1.138869	0.752872
6	1	0	2.564891	-2.924037	-0.084915
7	1	0	4.241108	-1.282115	0.691775
8	1	0	-0.629007	1.026749	-1.544303
9	16	0	-3.190959	0.636146	-0.760747
10	6	0	1.683329	0.815778	0.039325
11	6	0	2.950929	0.404493	0.424089
12	6	0	3.256454	-0.948828	0.388073
13	6	0	2.311041	-1.870981	-0.043189
14	6	0	1.041623	-1.467475	-0.452946
15	6	0	0.721128	-0.100249	-0.396847
16	17	0	1.281643	2.507425	0.099260
17	6	0	0.033830	-2.460658	-0.964790
18	1	0	-0.732790	-2.667265	-0.213490
19	1	0	-0.481702	-2.065583	-1.841679
20	1	0	0.522409	-3.398397	-1.227420
21	6	0	-2.750165	-0.818077	1.895613
22	1	0	-2.396740	-1.395055	2.749094
23	1	0	-3.220030	0.105387	2.240995
24	1	0	-3.495396	-1.393894	1.346929

Structure 33 (max 1) (M06-2X/6-311G(d,p), gas phase)

Energy (Hartrees): = -1317.3860344
Imaginary frequency -73.53

Standard orientation:

Center Number	Atomic Number	Atomic Type	Coordinates (Angstroms) X	Y	Z
1	6	0	-1.733565	-0.517785	-0.037275
2	7	0	-2.476924	-0.046787	-1.038707
3	1	0	-2.020639	0.022216	-1.936563
4	7	0	-0.390473	-0.773955	-0.454487
5	1	0	4.067624	-0.223472	0.154931
6	1	0	1.644213	3.299432	0.374601
7	1	0	3.876297	2.248077	0.481370
8	1	0	-0.147624	-1.715490	-0.165999
9	16	0	-2.297331	-0.839050	1.482845
10	6	0	1.967521	-0.468062	-0.111489
11	6	0	3.109642	0.277823	0.116452
12	6	0	2.996290	1.646284	0.295623
13	6	0	1.740009	2.228538	0.235820
14	6	0	0.576486	1.495074	0.003333
15	6	0	0.682616	0.099010	-0.171536
16	17	0	2.160864	-2.195492	-0.331580
17	6	0	-0.727179	2.255266	-0.063423
18	1	0	-1.182012	2.193591	-1.054045
19	1	0	-1.454631	1.894656	0.665892
20	1	0	-0.537354	3.307083	0.147953
21	6	0	-3.863333	0.341560	-0.892458
22	1	0	-4.223069	0.715420	-1.849127
23	1	0	-4.463889	-0.513563	-0.575865
24	1	0	-3.955468	1.119168	-0.129752

Structure 33 (max 2) (M06-2X/6-311G(d,p), gas phase)

Energy (Hartrees): = -1317.3843777
Imaginary frequency -66.19

Standard orientation:

Center Number	Atomic Number	Atomic Type	Coordinates (Angstroms) X	Y	Z
1	6	0	1.591508	-0.707616	-0.092693
2	7	0	2.306884	-0.101180	-1.036866
3	1	0	1.823166	0.107388	-1.897881
4	7	0	0.275828	-1.048475	-0.553081
5	1	0	-2.127247	2.767978	0.515385
6	1	0	-4.214048	-0.942498	0.037928
7	1	0	-4.263749	1.493895	0.527549
8	1	0	0.125681	-2.017206	-0.300208
9	16	0	2.151685	-1.125503	1.404560
10	6	0	-0.938787	1.066241	0.038231
11	6	0	-2.143949	1.705645	0.310184
12	6	0	-3.327939	0.993538	0.315607
13	6	0	-3.294426	-0.368474	0.042550
14	6	0	-2.102155	-1.023691	-0.225779
15	6	0	-0.882784	-0.306658	-0.226297
16	17	0	0.479187	2.092590	0.039807
17	6	0	-2.103933	-2.500839	-0.523032
18	1	0	-1.625859	-2.714645	-1.483331
19	1	0	-1.574778	-3.068428	0.250699
20	1	0	-3.124949	-2.877381	-0.561179
21	6	0	3.617131	0.462279	-0.793086
22	1	0	3.974780	0.923245	-1.711664
23	1	0	3.560086	1.208343	0.005293
24	1	0	4.306801	-0.321637	-0.476752

Structure 33 (min 1) (M06-2X/6-311G(d,p), $CHCl_3$)

Energy (Hartrees): = -1317.423558
No imaginary frequencies

Standard orientation:

Center Number	Atomic Number	Atomic Type	Coordinates (Angstroms) X	Y	Z
1	6	0	1.701360	-0.094968	-0.110880
2	7	0	2.846828	0.221683	-0.729692
3	1	0	2.810776	0.487683	-1.704032
4	7	0	0.589682	0.011501	-0.893710
5	1	0	-3.387963	1.698244	0.639789
6	1	0	-3.067761	-2.552853	0.118498
7	1	0	-4.397213	-0.569486	0.762083
8	1	0	0.692954	0.468498	-1.793236
9	16	0	1.639773	-0.589907	1.501698
10	6	0	-1.505274	0.949487	-0.061715
11	6	0	-2.819210	0.819856	0.362927
12	6	0	-3.373489	-0.451107	0.427575
13	6	0	-2.624574	-1.564901	0.065998
14	6	0	-1.307112	-1.438208	-0.369901
15	6	0	-0.740108	-0.158842	-0.424178
16	17	0	-0.795181	2.544537	-0.137370
17	6	0	-0.497144	-2.640336	-0.764061
18	1	0	-0.038155	-2.501653	-1.745576
19	1	0	0.309650	-2.804594	-0.044621
20	1	0	-1.124095	-3.531845	-0.791853
21	6	0	4.156302	0.152032	-0.108401
22	1	0	4.885520	0.517551	-0.829697
23	1	0	4.192903	0.773703	0.786901
24	1	0	4.405021	-0.874237	0.169483

Structure 33 (min 2) (M06-2X/6-311G(d,p), $CHCl_3$)

Energy (Hartrees): = -1317.4273584
No imaginary frequencies

Standard orientation:

Center Number	Atomic Number	Atomic Type	Coordinates (Angstroms) X	Y	Z
1	6	0	-1.713941	0.083000	-0.133242
2	7	0	-1.569662	-0.410865	1.100817
3	1	0	-0.632123	-0.517705	1.466597
4	7	0	-0.571441	0.253381	-0.851068
5	1	0	3.613307	1.265167	0.733685
6	1	0	2.673154	-2.862388	0.006458
7	1	0	4.274706	-1.129316	0.740959
8	1	0	-0.674727	0.741561	-1.730280
9	16	0	-3.217688	0.461563	-0.812855
10	6	0	1.642150	0.841854	0.003542
11	6	0	2.920608	0.496232	0.416534
12	6	0	3.279941	-0.845221	0.419172
13	6	0	2.377556	-1.819225	0.008259
14	6	0	1.096021	-1.478947	-0.422182
15	6	0	0.726660	-0.124495	-0.419534
16	17	0	1.166610	2.519155	0.015102
17	6	0	0.118461	-2.523851	-0.883354
18	1	0	-0.685770	-2.657124	-0.153626
19	1	0	-0.348487	-2.229777	-1.825477
20	1	0	0.618282	-3.482702	-1.019556
21	6	0	-2.673335	-0.646316	2.014585
22	1	0	-2.261938	-1.067692	2.930307
23	1	0	-3.196710	0.283248	2.246518
24	1	0	-3.386059	-1.349718	1.582435

Structure 33 (max 1) (M06-2X/6-311G(d,p), $CHCl_3$)

Energy (Hartrees): = -1317.4084235
Imaginary frequency -70.93

Standard orientation:

Center Number	Atomic Number	Atomic Type	Coordinates (Angstroms) X	Y	Z
1	6	0	-1.733565	-0.517785	-0.037275
2	7	0	-2.476924	-0.046787	-1.038707
3	1	0	-2.020639	0.022216	-1.936563
4	7	0	-0.390473	-0.773955	-0.454487
5	1	0	4.067624	-0.223472	0.154931
6	1	0	1.644213	3.299432	0.374601
7	1	0	3.876297	2.248077	0.481370
8	1	0	-0.147624	-1.715490	-0.165999
9	16	0	-2.297331	-0.839050	1.482845
10	6	0	1.967521	-0.468062	-0.111489
11	6	0	3.109642	0.277823	0.116452
12	6	0	2.996290	1.646284	0.295623
13	6	0	1.740009	2.228538	0.235820
14	6	0	0.576486	1.495074	0.003333
15	6	0	0.682616	0.099010	-0.171536
16	17	0	2.160864	-2.195492	-0.331580
17	6	0	-0.727179	2.255266	-0.063423
18	1	0	-1.182012	2.193591	-1.054045
19	1	0	-1.454631	1.894656	0.665892
20	1	0	-0.537354	3.307083	0.147953
21	6	0	-3.863333	0.341560	-0.892458
22	1	0	-4.223069	0.715420	-1.849127
23	1	0	-4.463889	-0.513563	-0.575865
24	1	0	-3.955468	1.119168	-0.129752

Structure 33 (max 2) (M06-2X/6-311G(d,p), $CHCl_3$)

Energy (Hartrees): = -1317.407627
Imaginary frequency -69.18

Standard orientation:

Center Number	Atomic Number	Atomic Type	Coordinates (Angstroms) X	Y	Z
1	6	0	1.595366	-0.692767	-0.078932
2	7	0	2.310616	-0.133985	-1.038967
3	1	0	1.849394	-0.000705	-1.929941
4	7	0	0.280495	-1.052541	-0.515954
5	1	0	-2.157554	2.766510	0.478553
6	1	0	-4.215362	-0.964368	0.024464
7	1	0	-4.282683	1.478639	0.489693
8	1	0	0.136789	-2.024114	-0.264473
9	16	0	2.156901	-1.039297	1.452553
10	6	0	-0.951895	1.062851	0.032173
11	6	0	-2.162288	1.700967	0.286786
12	6	0	-3.341985	0.980506	0.291687
13	6	0	-3.298595	-0.385210	0.032962
14	6	0	-2.100571	-1.038301	-0.219007
15	6	0	-0.883628	-0.313459	-0.213924
16	17	0	0.464297	2.098295	0.023046
17	6	0	-2.093022	-2.515776	-0.509269
18	1	0	-1.604518	-2.732640	-1.464043
19	1	0	-1.567497	-3.078593	0.270325
20	1	0	-3.113059	-2.895285	-0.555961
21	6	0	3.646774	0.391282	-0.852530
22	1	0	3.982465	0.817751	-1.795306
23	1	0	3.643565	1.161309	-0.076183
24	1	0	4.326286	-0.405658	-0.544988
